# Epidemiological trends of hand osteoarthritis from 1990 to 2019: Estimates from the 2019 Global Burden of Disease study

**DOI:** 10.3389/fmed.2022.922321

**Published:** 2022-12-12

**Authors:** Junlai Wan, Xiaoyuan Qian, Zhiyi He, Ziqing Zhu, Peng Cheng, Anmin Chen

**Affiliations:** ^1^Department of Orthopedics, Tongji Hospital, Tongji Medical College, Huazhong University of Science and Technology, Wuhan, China; ^2^Department of Urology, Xiangyang Central Hospital, Affiliated Hospital of Hubei University of Arts and Science, Xiangyang, China

**Keywords:** Global Burden of Disease, hand osteoarthritis, incidence, age-standardized rates, disability-adjusted life years

## Abstract

**Background:**

Hand osteoarthritis (OA) is a chronic progressive disease characterized by disabling pain in the hand, with a high clinical burden. This study is designed to assess the epidemiological patterns of hand OA from 1990 to 2019 and analyze its secular trends based on sex, age, and socio-demographic index (SDI) at global, regional, and national levels.

**Methods:**

Data on the incidence and disability-adjusted life years (DALYs) of hand OA were extracted from the 2019 Global Burden of Disease (GBD), and their respective age-standardized rates (ASRs) were calculated. The estimated annual percentage changes (EAPCs) in ASR were calculated to assess the prevalent trends of the incidence and DALYs of hand OA over the recent three decades. The relationship between ASR and SDI was analyzed by Pearson's correlation analysis.

**Results:**

The incidence of hand OA increased from 371.30 million in 1990 to 676.02 million in 2019, increasing by 82.07%, whereas its age-standardized incidence rate (ASIR) decreased, with a downward trend [EAPC = −0.34; 95% confidence interval: −0.39–−0.28]. With the changes in age, the incidence of hand OA exhibited a unimodal distribution before 70 years of age, peaking at 50–54 years, while its incidence had an upward trend in the >70 years age groups. Overall, hand OA-related DALYs increased in the recent 30 years. Meanwhile, its annual age-standardized DALY rate decreased, with EAPCs of −0.35 (95% CI, −0.38 –−0.32). The DALYs increased with age. In 2019, the ASIR and age-standardized DALY rate were positively associated with the SDI regions. The incidence and DALYs presented predominance in female patients. The burden of hand OA over the recent three decades displayed obvious geographical diversity.

**Conclusion:**

The incident cases of hand OA increased globally from 1990 to 2019, while the ASIR and age-standardized DALY rate decreased. However, in many countries and regions, there was a rising trend of ASR related to incidence and DALYs. In addition, the prevalence revealed geographical, sex, and age diversity. Thus, governments and medical institutions should reallocate medical resources based on the epidemiological characteristics of hand OA.

## Introduction

Osteoarthritis (OA) is a common disability characterized by the pain and impairment of joint function caused by cartilage degradation and inflammation response (1–4). Patients with OA often present pain, stiffness, and limited joint motion, including reduced pinch and/or grip strength (5–8). To date, except for surgery, no other intervention can prevent, treat, or even inhibit the development of OA (9–11). Non-steroidal anti-inflammatory drugs (NSAIDs) are commonly used clinically for the relief from OA, but with limited efficacy and an increased clinical risk of gastrointestinal peptic ulcer and cardiovascular disease (12, 13). Of the common types of OA, that of the knee is the most common, followed by hand OA (14). Hand OA is a type of heterogeneous disease that generally involves multiple joints (15) and different types of joints, such as OA of the interdigital joint and/or first carpal metacarpal joint (16), that seriously affects the quality of life of the patient. As an important functional organ, hand OA is of great significance for a high quality of life (17). Thus, hand OA deserves more social and scientific attention.

The definition of hand OA depends largely on radiology and symptoms. According to radiological analysis, the prevalence of hand OA ranges from 21% of the US population to 92% of the Japanese population, which is higher than that of hip OA (1.0–45.0%) and knee OA (7.1–70.8%), based on statistical data from different countries (5). Similarly, the prevalence of symptomatic hand OA, which ranges from 3% of the population of Iran and China to 16% of the population of the United States, is higher than that reported for hip OA (0.9–7.4%) during the same period. By contrast, the prevalence of symptomatic hand OA has been estimated to be slightly lower than that of knee OA (5.4–24.2%) (5). The etiology of hand OA is somewhat different from OA of large joints, such as the knee and hip. In addition to excessive mechanical load and wear of joints, several characteristics of hand OA are unique. For example, the peak incidence period of hand OA is around menopause, and the early inflammatory phase often occurs before bone remodeling (5). In addition, the interventions of hand OA are mostly based on the judgment of experts instead of evidence due to the limitation of clinical trials (17).

A few studies have revealed that the burden of OA is based on the region and the country (18–22). The global disease burden of knee and hip OA has been reported from 1990 to 2017 (23). However, studies, especially on global, regional, and national burdens of hand OA and its relationship with age, sex, and socio-demographic index (SDI), are not yet available. Hence, there is a need for a systematic analysis of the epidemiological trends of hand OA based on up-to-date datasets.

Data of hundreds of diseases and injuries from 192 countries and territories were included in the Global Burden of Disease (GBD) study (24–28), which provided an opportunity to comprehensively evaluate the secular prevalence trend of different diseases and injuries, including hand OA, based on its epidemiological characteristics including geography, age, sex, and SDI (29). The incidence and disability-adjusted life years (DALYs) from 1990 to 2019 associated with hand OA, which were obtained from the 2019 GBD, were used to evaluate the burden of hand OA and the epidemiological trends of hand OA.

## Methods

### Study data

Data related to hand OA, including the annual incidence and DALYs, were obtained from the 2019 GBD study database using the Global Health Data Exchange (GHDx) query tool (http://ghdx.healthdata.org/gbd-results-tool). The GHDx is a constantly updated website reflecting global and regional epidemiological data. Its data are mainly obtained from the following aspects: published reports and systematic reviews, data collected by official and non-governmental websites, raw data not yet published, and data from GBD collaborators. To date, a total of 329 diseases in 192 countries and territories have been included in the GBD database to assess their global burden on human health. These 192 countries and territories were divided into 21 regions according to geographic proximity and epidemiological similarity. The sex and age of the patients and country SDI values were also collected to assess their impact on incidence and DALYs and their age-standardized rate (ASR). The value of SDI, ranging from 0 to 1, is a comprehensive index of social and demographic developments. Based on the order of their SDI value, 192 countries and regions worldwide are classified as low, low-middle, middle, high-middle, and high SDI. The information on national SDI values was acquired from GHDx (http://ghdx.healthdata.org/record/ihme-data/gbd-2019-socio-demographicindex-sdi-1950-2019).

### Statistical analysis

The ASR of incidence and DALYs were analyzed, and their estimated annual percentage changes (EAPCs) were calculated to describe the prevalence trends for hand OA incidence and DALY rates. The total years lived with disability and years of life lost were summed to evaluate the DALYs of hand OA. ASRs (per 100,000 persons) were calculated according to the age groups of the standard population using the following formula:


$$\begin{array}{l} \text{ASR}=\frac{\sum_{i=1}^{A}{a_{i}w}_{i}}{\sum_{i=\;1}^{A}a_{i}}\times 100,000 \end{array}$$

*a*_*i*_ is the incidence of the ith age group; *w*_*i*_ is the number of persons (or weight) in the same age subgroup *i* of the assigned reference standard population (30).

The estimated annual percentage changes are widely well-acknowledged to reflect ASRs by a regression model and to quantitatively calculate the average annual rate of ASR changes for a specified period (31). The natural logarithm of the rates is assessed using the regression line, i.e., f(x) = α + βx + ε, in which f(x) is ln (ASR) and x is the calendar year. The EAPC calculation formula, 100 × (exp(β)-1), and its 95% confidence intervals (CIs) can also be calculated based on the linear regression model (30, 31). All statistical data were analyzed by R (version 3.6.3), and a two-sided *P* of < 0.05 was deemed statistically significant.

## Results

### Changes in the global incidence of hand OA

**Figure 1:**
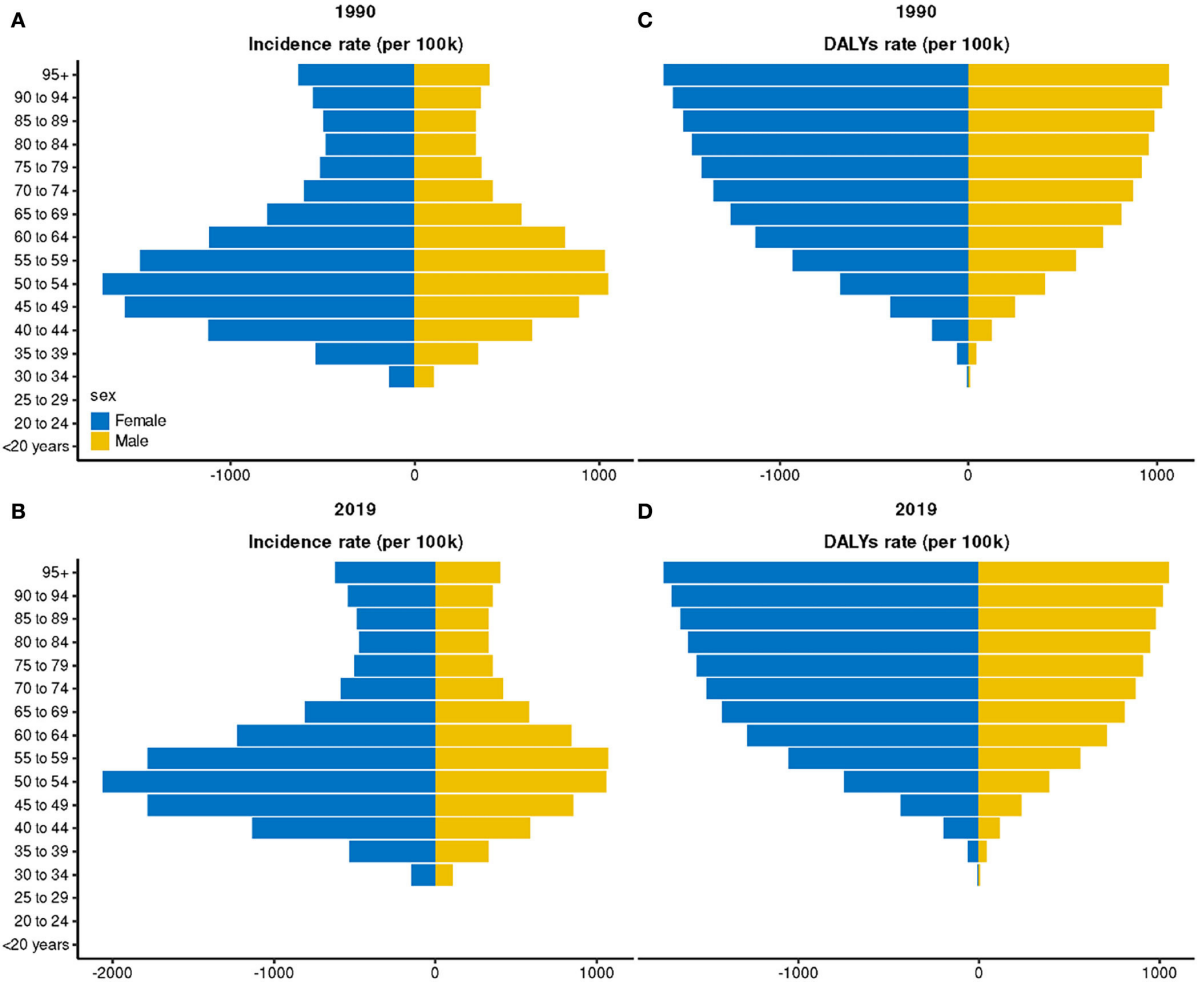
Incidence and disability-adjusted life year (DALY) rate of hand osteoarthritis in different sex and age groups. **(A)** Incidence rate in 1990; **(B)** Incidence rate in 2019; **(C)** DALY rate in 1990; and **(D)** DALY rate in 2019.

**Figure 2:**
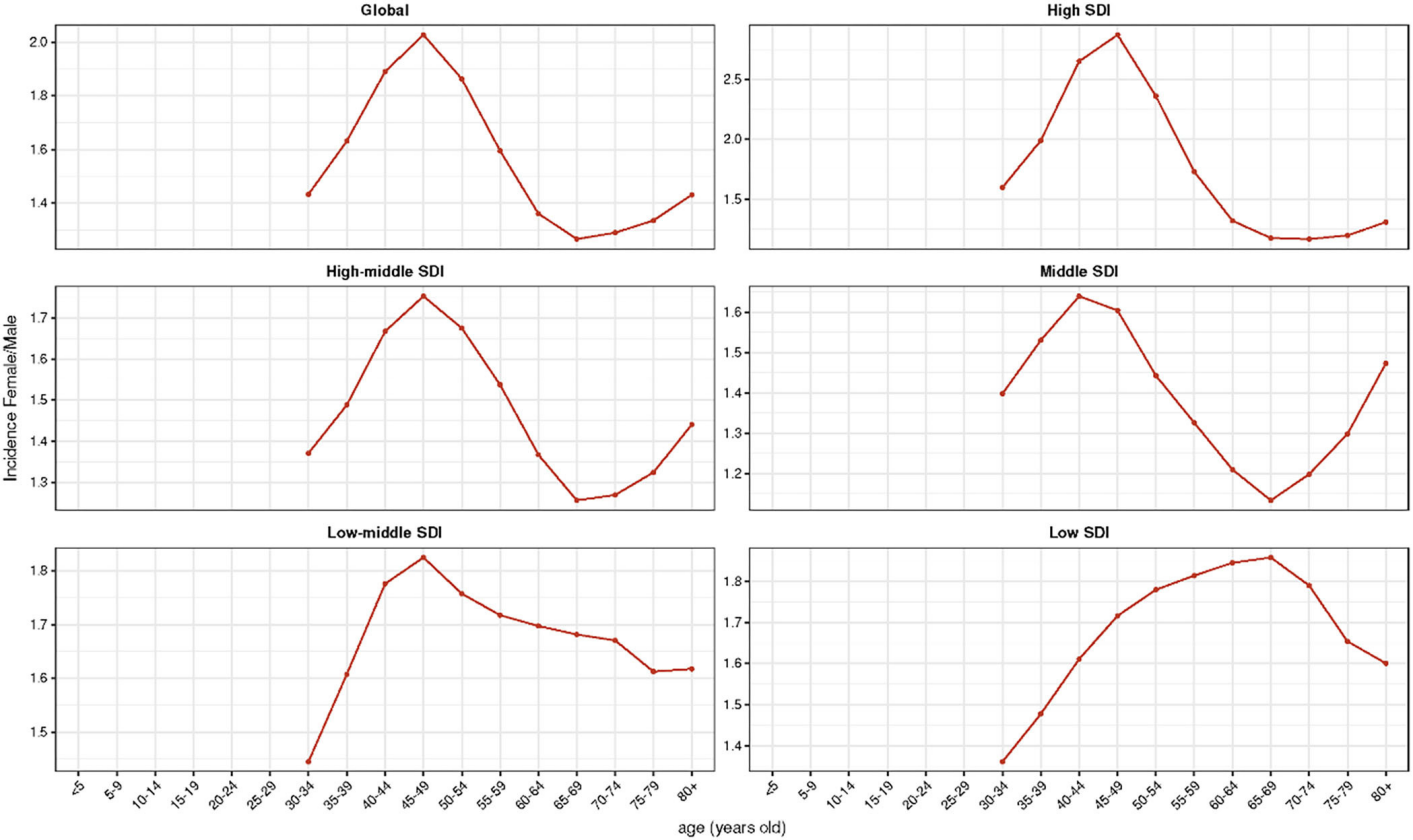
Female-to-male ratio of the incidence among different age groups and different socio-demographic index (SDI) quintiles in 2019.

The global incident cases of hand OA in 2019 reached 6.76 × 10^6^ [95% uncertainty interval (UI): 5.20 × 10^6^-8.84 × 10^6^], compared to 3.71 × 10^6^ (UI: 2.86 × 10^6^-4.84 × 10^6^) in 1990, with an 82.07% increase over these three decades (Table 1, Supplementary Figure 1A). While the ASIR exhibited a downward trend, decreasing by an average of 0.34% per year from 85.41/100,000 persons (95% UI, 65.91–111.44) in 1990 to 80.14/100,000 persons (95% UI: 61.79–104.84) in 2019 (EAPC = −0.34; 95% CI: −0.39 to −0.28; Table 1, Supplementary Figure 1B). People in the age group ranging from 40 to 59 years were found to be most affected (Supplementary Figure 2). Moreover, the incidence of hand OA was higher in female patients than in male patients, while the trend of their ASIR was correspondingly decreased, respectively (Table 1, Supplementary Figure 1B). Ignoring the factor of sex, before 70 years of age, the incidence of hand OA based on age was the highest in patients aged 50–54 years, but an upward trend was found in the >70 years age groups (Figures 1A,B, Supplementary Figures 3–5). A higher DALY rate was reported for female patients with hand OA than for male patients, which increased with age (Figures 1C,D). Globally, the incidence ratio of female and male patients presented a unimodal age distribution, peaking at 45–49 years of age, while this ratio increased slightly after 65 years (Figure 2).

**Table 1:** Incident cases and ASIR in 1990 and 2019 and its current trends from 1990 to 2019.

	**1990**		**2019**		**1990–2019 EAPC**
	**Incident cases**	**ASIR per 100,000**	**Incident cases**	**ASIR per 100,000**	
	No. *10^2(95%UI)^	**No. (95% UI)**	No. *10^2(95%UI)^	**No. (95% UI)**	**No. (95% CI)**
Overall	37,130.45 [28,555.15–483,70.01]	85.41 [65.91–111.44]	67,601.68 [52,002.6–88,423.18]	80.14 [61.79–104.84]	−0.34 [−0.39 to −0.28]
**Sex**					
Female	23,516.87 [18,158.76–30,543.68]	107.45 [82.48–139.53]	42,760.08 [32,803.28–55,817.9]	99.83 [76.72–129.4]	−0.34 [−0.39 to −0.28]
Male	13,613.58 [10,389.19–17,842.34]	63.29 [48.6–83.25]	24,841.6 [18,927.55–32,805.52]	60.04 [46.08–79.19]	−0.34 [−0.39 to −0.28]
**Socio–demographic index**					
High SDI	15,062.85 [11,655.81–19,403.53]	161.19 [123.78–209.78]	24,994.55 [18,992.6–32,950.37]	177.1 [136.44–232.41]	0.53 [0.41 to 0.66]
High–middle SDI	11,171.25 [8,568.66–14,622.87]	100.14 [76.72–130.6]	16,705.15 [12,574.14–21,896.79]	84.88 [64.09–110.47]	−1.01 [−1.19 to −0.84]
Middle SDI	5,575.74 [4,263.26–7,407.67]	45.91 [35.13–60.87]	13,813.81 [10,522.09–18,278.24]	50.42 [38.75–66.6]	−0.02 [−0.2 to 0.16]
Low–middle SDI	3,345.96 [2,541.34–4,479.31]	46.78 [35.86–62.59]	7,550.87 [5,760.5–10,045.71]	49.2 [37.82–65.54]	0.08 [0.04 to 0.13]
Low SDI	1,964.06 [1,492.89–2,594.42]	65.93 [50.46–86.57]	4,516.97 [3,428.84–5,951.67]	67 [51.32–87.82]	0.05 [0.05 to 0.06]
**Region**					
Andean Latin America	134.72 [101.77–179.28]	55.92 [42.44–74.26]	359.38 [281.7–459.03]	59.62 [46.46–76.7]	0.37 [0.3 to 0.45]
Australasia	353.87 [271.07–465.46]	163.37 [124.13–217.24]	647.4 [490.58–871.45]	171.72 [130.09–227.87]	−0.1 [−0.22 to 0.01]
Caribbean	111.68 [84.73–149.19]	41.01 [31.08–54.5]	213.1 [160.96–283.49]	41.15 [31.17–54.64]	0.01 [0.01 to 0.01]
Central Asia	531.81 [398.89–718.17]	104.67 [79.26–139.23]	956.53 [709.49–1,276.78]	103.42 [78.07–137.41]	0 [−0.04 to 0.05]
Central Europe	798.59 [604.25–1,074.05]	55.57 [42.11–74.68]	962.95 [725.49–1,295.1]	55.35 [41.97–74.62]	−0.02 [−0.02 to −0.01]
Central Latin America	797.77 [605.17–1,066.94]	78.88 [59.63–105.53]	2,272.23 [1,708.68–3,039.56]	88.9 [67.39–117.98]	0.57 [0.44 to 0.7]
Central Sub–Saharan Africa	248.93 [189.11–328.01]	82.85 [63.17–108.6]	636.49 [479.82–839.5]	82.46 [62.96–108.12]	−0.02 [−0.03 to −0.02]
East Asia	4,558.81 [3,407.63–6,165.56]	45.7 [34.55–61.43]	11,312.1 [8,401.22–15,264.36]	52.5 [39.5–70.24]	−0.26 [−0.64 to 0.12]
Eastern Europe	5,803.61 [4,436.46–7,561.45]	222.55 [171.27–284.79]	6,213.55 [4,738.48–8,026.34]	215.56 [164.82–278.62]	−0.82 [−1.11 to −0.53]
Eastern Sub–Saharan Africa	821.81 [623.36–1,079.42]	84.5 [64.36–110.6]	1,956.47 [1,476.78–2,575.82]	84.9 [64.75–111.01]	0.02 [0.02 to 0.02]
High–income Asia Pacific	2,971.07 [2,270.66–3,906.45]	140.96 [108.46–184.01]	4,632.74 [3,562.07–6,003.34]	162.72 [123.75–212.42]	1.4 [1.1 to 1.71]
High–income North America	7,360.11 [5,717.52–9,388.73]	245.34 [187.78–316.72]	13,312.66 [9,975.04–17,562.83]	275.59 [211.37–357.91]	0.45 [0.2 to 0.71]
North Africa and the Middle East	998.25 [754.66–1,336.31]	47.49 [36.11–62.98]	2,817.59 [2,129.17–3,772.41]	50.2 [38.25–66.43]	0.11 [0.08 to 0.14]
Oceania	5.81 [4.23–7.96]	16.22 [12.08–22.02]	14.29 [10.4–19.5]	16.25 [12.1–22.04]	0 [0 to 0]
South Asia	3,201.55 [2,402.52–4,313.65]	46.18 [35.29–61.86]	7,349.91 [5,590.98–9,894]	47 [35.89–62.86]	0.07 [0.06 to 0.07]
Southeast Asia	229.07 [165.57–311.81]	8.15 [6.13–10.79]	532.78 [384.84–729.17]	8.15 [6.05–10.78]	0 [0 to 0]
Southern Latin America	743.24 [561.28–979.86]	160.11 [120.97–211.46]	1,219.95 [923.16–1,615.98]	160.22 [121.05–211.51]	0 [0 to 0]
Southern Sub-Saharan Africa	344.8 [261.53–447.63]	102.95 [78.75–133.99]	716.13 [543.43–931.38]	103.55 [79.27–134.85]	0.03 [0.02 to 0.03]
Tropical Latin America	486.47 [363.97–648.23]	46.09 [35.2–61.02]	1,175.87 [891.6–1,559.3]	46.38 [35.43–61.38]	0.02 [0.02 to 0.02]
Western Europe	5,711.85 [4,379–7,553.19]	119.31 [90.98–156.87]	8,044.89 [6,035.81–10,582.64]	125.73 [93.92–163.78]	0.34 [0.28 to 0.4]
Western Sub-Saharan Africa	916.65 [696.53–1,197.37]	84.14 [64.25–109.97]	2,254.7 [1,698.46–2,964.85]	85.81 [65.31–111.93]	0.08 [0.07 to 0.09]

ASIR, age-standardized incidence rate; UI, uncertainty interval; CI, confidence interval; EAPCs, estimated annual percentage changes; SDI, socio-demographic index.

**Figure 3:**
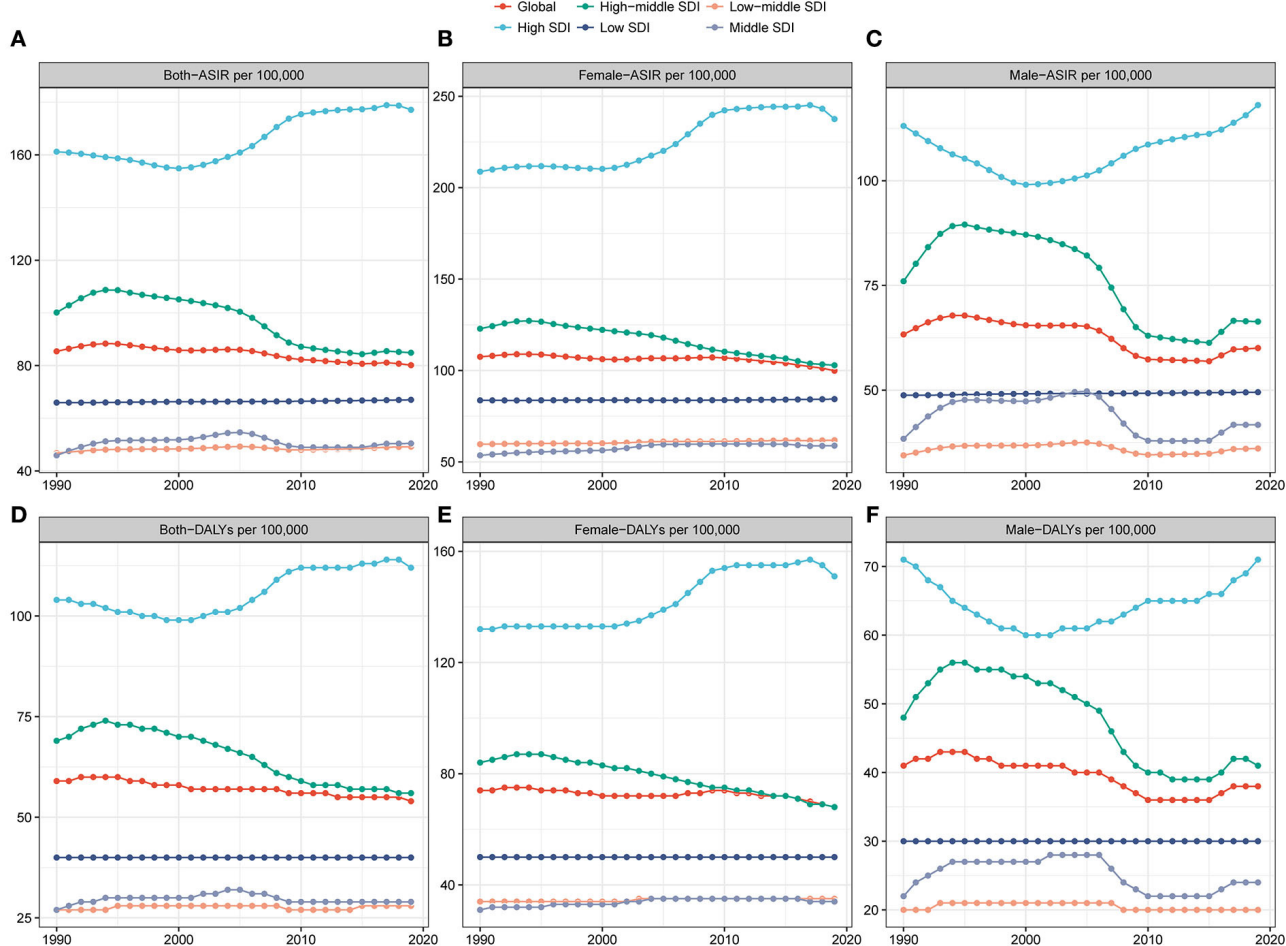
Change trends of age-standardized incidence rate (ASIR) and age-standardized disability-adjusted life year (DALY) rate among different genders and different socio-demographic index (SDI) quintiles. **(A–C)** ASIR; **(D–F)** age-standardized DALY rate.

**Figure 4:**
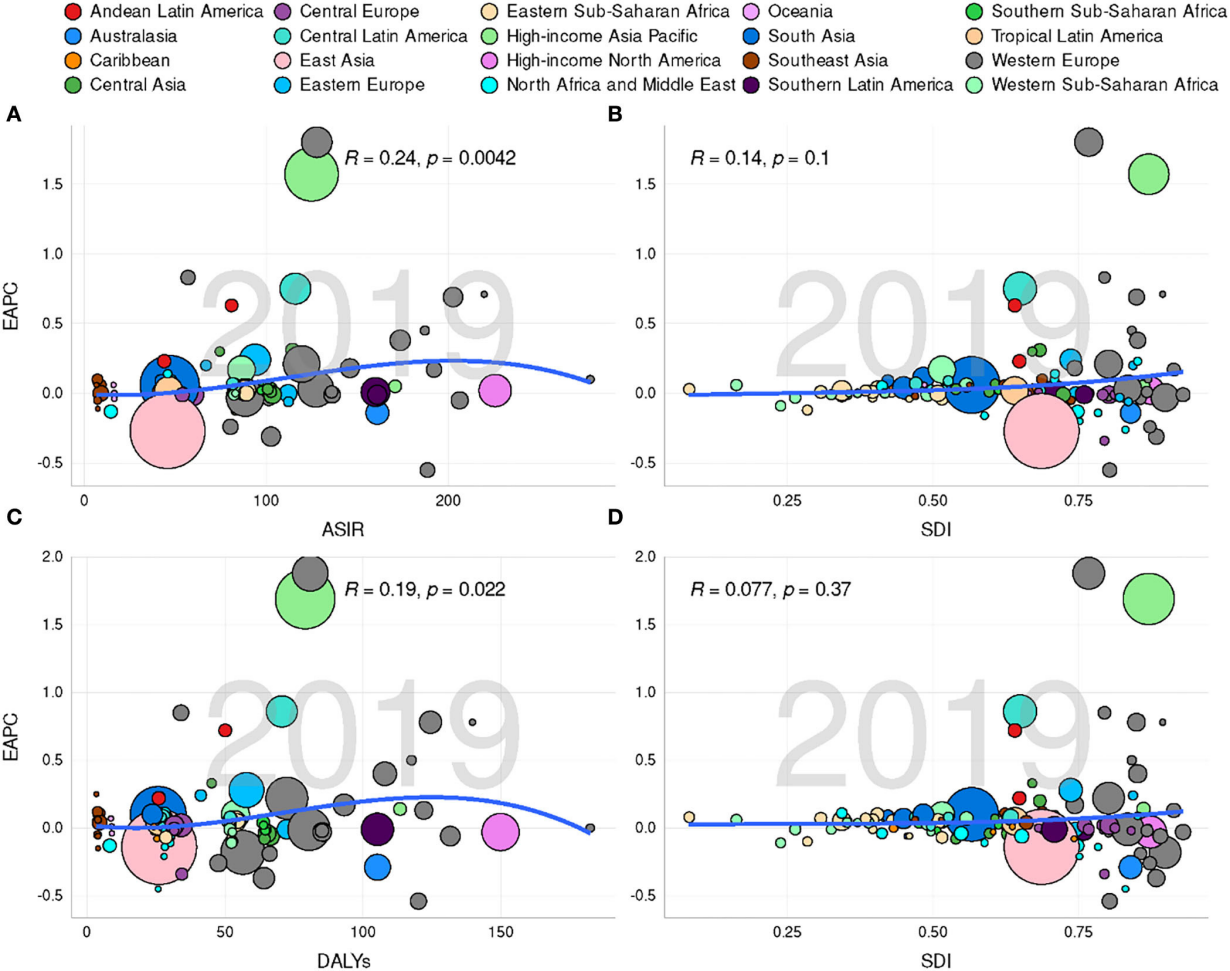
Correlation between estimated annual percentage changes (EAPCs) and hand osteoarthritis age-standardized rates [ASRs; incidence **(A)** and DALYs **(C)**] in 2019 and socio-demographic index [SDI; incidence **(B)** and DALYs **(D)**] in 2019. Circles represent different countries with their SDI values. The size of these circles reflects the number of hand osteoarthritis subjects. The *R*- and *P*-values were calculated by Pearson's correlation analysis.

Considering the SDI level, compared with regions having a relatively low ASIR, the high SDI region had a high ASIR (Table 1, Figures 3A–C). The high-middle SDI regions had a significant decline in ASIR (EAPC = −1.01, 95% CI: −1.19 –−0.84), whereas the middle SDI regions exhibited a relatively stable ASIR (EAPC = −0.02, 95% CI: −0.2 – 0.16). However, a slightly increasing trend was observed in the other three regions (Table 1). The incidence ratio of female and male patients presenting unimodal distribution was highest between 55 and 74 years in the low SDI region and between 45 and 49 years in the other four regions (Figure 2). Meanwhile, the ASIR increased with the increase in SDI (*R* = 0.33, *P* < 0.05; Supplementary Figure 6A). Moreover, the higher the ASIR, the greater the EAPC (*R* = 0.24, *P* = 0.004), suggesting that the increase in the incidence of hand OA was more rapid in regions or countries with high ASIR than in those with low ASIR (Figure 4A). However, the trend of ASIR (*R* = 0.14, *P* = 0.1) did not appear to be affected by the SDI of regions or countries (Figure 4B).

**Figure 5:**
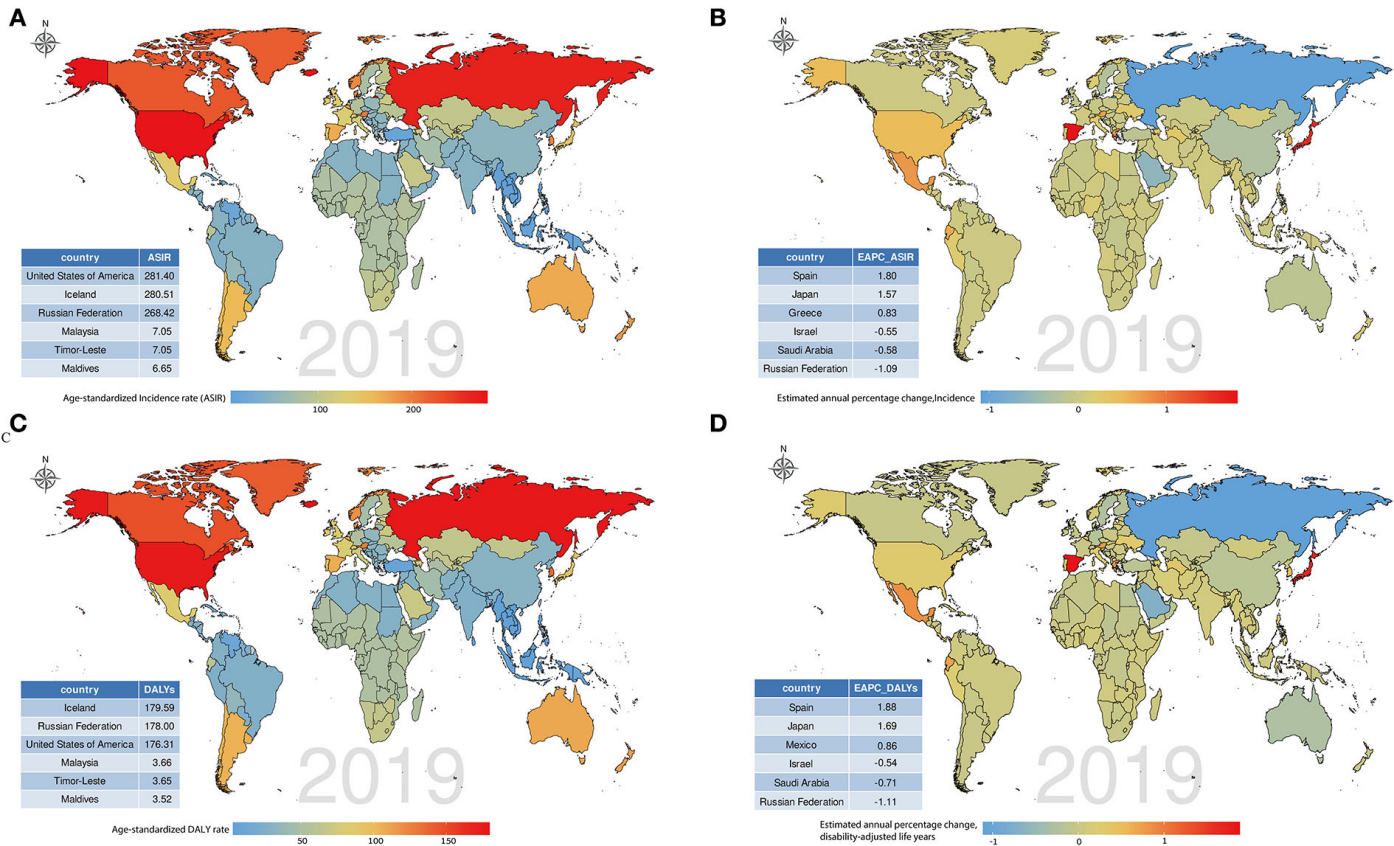
Global disease burdens of hand osteoarthritis and their estimated annual percentage changes (EAPCs) in 192 countries. **(A)** The age-standardized incidence rate (ASIR) of hand osteoarthritis in 2019; **(B)** The EAPCs of ASIR from 1990 to 2019; **(C)** The age-standardized disability-adjusted life year (DALY) rate of hand osteoarthritis in 2019; and **(D)** The EAPCs of DALYs from 1990 to 2019. Countries with extremums were annotated in the tabulation at the bottom left.

Regarding the findings in GBD regions and countries, the ASIR of hand OA exhibited an increasing trend in 88 countries and 12 regions, a decreasing trend in 75 countries and three regions, and remained unchanged in 29 countries and six regions. The USA (281.4 per 100,000 people), Iceland (280.51 per 100,000 people), and the Russian Federation (268.42 per 100,000 people) had the highest ASIR, whereas Maldives (6.65 per 100,000 people), Timor-Leste (7.05 per 100,000 people), and Malaysia (7.05 per 100,000 people) had the lowest ASIR. Spain (1.8), Japan (1.57), and Greece (0.83) were the three countries with the highest EAPCs, whereas the Russian Federation (−1.09), Saudi Arabia (−0.58), and Israel (−0.55) had the lowest EAPCs. The above findings are presented in Figures 5A,B, Supplementary Tables 1–3. Moreover, the incidence of hand OA displayed a unimodal age distribution in most countries (Supplementary Table 4).

### Changes in global disability-adjusted life years of hand OA

Globally, 2.33 × 10^9^ (95% UI, 1.15 × 10^9^-4.85 × 10^9^) DALYs were reported in 1990 and 4.45 × 10^9^ (95% UI, 2.20 × 10^9^-9.23 × 10^9^) in 2019, increasing by 91.35% in recent 30 years. The ASR of DALYs decreased from 58.85/100,000 persons (95% UI, 29.16–122.38) in 1990 to 53.87/100,000 persons (95% UI, 26.59–111.49) in 2019 (EAPC = −0.35, 95% CI, −0.38 to−0.32; Table 2, Supplementary Figure 1C). EAPC was closely associated with the ASR of DALYs (*R* = 0.19, *P* = 0.022; Figure 4C). Moreover, the ASR of DALYs was higher in female patients than that in male patients (Table 2). Globally, the incidence ratio of female and male patients presented a unimodal age distribution, peaking at 50–54 years of age, while this ratio had a slight decrease after 55 years (Supplementary Figure 10).

**Table 2:** DALYs and age-standardized DALY rate in 1990 and 2019 and its up-to-date trends from 1990 to 2019.

	**1990**		**2019**		**1990–2019 EAPC**
	**DALYs**	**Age–standardized DALYs rate**	**DALYs**	**Age-standardized DALYs rate**	
	No. *10^3^ (95% UI)	**per 100,000 No. (95% UI)**	No. *10^3^ (95% UI)	**per 100,000 No. (95% UI)**	**No. (95% CI)**
Overall	23,281.38 [11,472.67–48,520.15]	58.85 [29.16–122.38]	44,549.35 [21,984.23–92,341.84]	53.87 [26.59–111.49]	−0.35 [−0.38 to −0.32]
**Sex**					
Female	15,650.52 [7,762.19–32,399.98]	74.02 [36.81–153.1]	29,717.63 [14,846.19–61,321.81]	68 [33.93–140.24]	−0.35 [−0.38 to −0.32]
Male	7,630.86 [3,756.83–16,157.98]	41.08 [20.14–86.6]	14,831.73 [7,265.46–31,302.71]	37.93 [18.81–79.01]	−0.35 [−0.38 to −0.32]
**Socio-demographic index**					
High SDI	10,493.3 [5,233.34–21,884.12]	104.01 [51.91–217.96]	19,468.45 [9,704.71–40,787.69]	112.44 [56.48–234.4]	0.49 [0.36 to 0.62]
High-middle SDI	7,368.22 [3,659.36–15,252.26]	68.53 [34.07–141.88]	11,476.92 [5,702.11–23,924.18]	56.01 [27.86–117.1]	−1.07 [−1.22 to −0.93]
Middle SDI	2,812.51 [1,370.17–5,979.69]	27 [13.2–56.48]	7,565.44 [3,692.28–16,041.09]	29.47 [14.48–61.85]	0.03 [−0.12 to 0.19]
Low-middle SDI	1,626.16 [789.63–3,444.62]	26.87 [13.29–56.48]	3,858.5 [1,887.17–8,170.83]	27.8 [13.77–58.52]	0.04 [0 to 0.08]
Low SDI	974.62 [472.94–2,020.64]	39.82 [19.6–83.03]	2,167.35 [1,054.74–4,477.31]	39.93 [19.7–83.09]	0.01 [0.01 to 0.01]
**Region**					
Andean Latin America	70.19 [34.08–147.48]	33.73 [16.52–71.48]	205.85 [102.91–428.32]	36.32 [18.09–74.76]	0.42 [0.34 to 0.49]
Australasia	243.07 [122.56–514.93]	106.44 [53.25–224.56]	501.08 [248.55–1,035.49]	109.63 [55.14–229.46]	−0.24 [−0.37 to −0.11]
Caribbean	62.58 [30.67–130.47]	24.16 [11.86–50.25]	126.14 [61.95–260.85]	24.23 [11.88–50.17]	0.01 [0 to 0.01]
Central Asia	316.98 [155.41–661.18]	67.59 [33.07–142.05]	510.04 [250.4–1,070.76]	65.87 [32.21–137.65]	−0.06 [−0.11 to −0.01]
Central Europe	475.56 [231.39–996.11]	32.4 [15.86–68.31]	659.46 [323.71–1,383.94]	32.39 [15.91–67.75]	0 [0 to 0]
Central Latin America	407.4 [199.65–848.67]	47.57 [23.27–99.48]	1,300.43 [640.49–2,697.18]	53.94 [26.62–111.55]	0.61 [0.47 to 0.75]
Central Sub–Saharan Africa	125.67 [61.66–260.25]	51.98 [25.6–107.02]	304.75 [149.46–636.6]	52.74 [26.18–109.9]	0.05 [0.04 to 0.06]
East Asia	2,312.14 [1,125.87–4,889.55]	26.13 [12.91–54.58]	6,428.95 [3,138.16–13,399.66]	30.02 [14.7–62.64]	−0.14 [−0.48 to 0.21]
Eastern Europe	4,114.19 [2,065.73–8,411.96]	147.45 [74.54–301.81]	4,734.4 [2,361.8–9,854.74]	141.05 [70.38–293.25]	−0.81 [−1.08 to −0.55]
Eastern Sub–Saharan Africa	411.68 [201.59–857.8]	52.53 [25.96–108.86]	930.01 [455.63–1,936.24]	53.31 [26.44–110.46]	0.06 [0.06 to 0.07]
High–income Asia Pacific	1,832.06 [900.8–3,810.06]	89.36 [44.01–185.22]	4,048.28 [2,018.5–8,409.84]	105.1 [52.6–219.92]	1.52 [1.18 to 1.86]
High–income North America	5,341.12 [2,729.68–11,182.3]	162.16 [82.83–341.11]	10,244.26 [5,128.85–21,641.64]	173.29 [87.85–360.45]	0.24 [0.02 to 0.46]
North Africa and the Middle East	503.35 [246.69–1,066.81]	28.27 [13.87–59.8]	1,353.36 [658.01–2,838.53]	29.22 [14.32–61.61]	0.03 [0 to 0.06]
Oceania	2.58 [1.19–5.44]	8.77 [4.17–18.36]	6.21 [2.92–12.98]	8.72 [4.2–17.99]	−0.02 [−0.02 to −0.01]
South Asia	1,461.84 [711.22–3,089.64]	25.49 [12.65–53.68]	3,697.71 [1,822.88–7,673.07]	26.15 [13–54.95]	0.1 [0.1 to 0.1]
Southeast Asia	104.46 [47.47–219.91]	4.18 [1.92–8.63]	255.95 [115.97–535.14]	4.21 [1.93–8.67]	0.03 [0.02 to 0.03]
Southern Latin America	485.66 [241.26–1,015.51]	105.07 [52.27–219.66]	852.2 [421.91–1,767.29]	104.63 [51.98–217.69]	−0.01 [−0.02 to −0.01]
Southern Sub–Saharan Africa	187.77 [92.52–390.64]	66.11 [33–136.68]	390.24 [193.25–808.62]	66.08 [33.01–136.14]	0 [0 to 0]
Tropical Latin America	240.52 [115.99–495.28]	26.15 [12.68–53.77]	651.9 [315.98–1,342.9]	26.58 [12.93–54.73]	0.05 [0.04 to 0.05]
Western Europe	4,102.99 [2,038.5–8,601.38]	74.44 [37.11–155.51]	6,259.65 [3,076.68–12,738.8]	77.22 [37.99–158.06]	0.3 [0.23 to 0.36]
Western Sub–Saharan Africa	479.56 [235.86–1,007.73]	52.79 [26.06–110.19]	1,088.48 [534.39–2,269.79]	53.54 [26.39–111.38]	0.06 [0.04 to 0.07]

DALYs, disability-adjusted life years; UI, uncertainty interval; CI, confidence interval; EAPCs, estimated annual percentage changes.

Considering SDI, compared with other SDI regions, the high SDI region was accompanied by a higher ASR of DALYs (Table 2, Figures 3D–F). The high to middle SDI regions exhibited a significant downward trend of ASR (EAPC = −1.07, 95% CI: −1.22–−0.93), while the ASR trend of DALYs was stable in the middle SDI regions (EAPC = 0.03. 95% CI: −0.12–0.19), but a slightly increasing trend was seen in the other SDI regions (Table 2). DALYs were also associated with SDI (*R* = 0.32, *P* < 0.001; Supplementary Figure 6B). Furthermore, no significant correlation was recognized between the EAPC of ASR of DALYs and the value of SDI (*R* = 0.077, *P* = 0.37; Figure 4D). The DALYs increased with age in all SDI regions, especially after 30 years (Supplementary Figures 7–9). Except in the high-middle and low SDI regions, the DALY ratio of female and male patients presented a steep upward trend before 60 years, which decreased over 60 years. Interestingly, the DALY ratio of female and male patients showed a relatively increasing trend in the high-middle and low SDI regions (Supplementary Figure 10).

From the perspective of GBD regions and countries, 95 countries and 13 regions had an increasing trend of age-standardized DALY rate, and 81 countries and five regions had a decreasing trend. Meanwhile, 16 countries and three regions exhibited a stable trend of age-standardized DALY rate. Countries with high age-standardized DALY rates were Iceland (179.59/100,000 persons), the Russian Federation (178/100,000 persons), and the USA (176.31/100,000 persons), whereas those with the lowest rates were the Maldives (3.52/100,000 persons), Timor-Leste (3.65/100,000 persons), and Malaysia (3.66/100,000 persons). The three countries with the largest increasing trend in DALYs were Spain (1.88), Japan (1.69), and Mexico (0.86), whereas the three countries with the largest decreasing trend were the Russian Federation (−1.11), Saudi Arabia (−0.71), and Israel (−0.54; Figures 5C,D, Supplementary Tables 1, 2, 5). Similarly, when sex was not considered, DALYs rapidly increased with age in most countries (Supplementary Table 6).

## Discussion

Hand osteoarthritis is the most common type of OA and is still a major global public health issue requiring more attention. Only a few prior epidemiological studies on hand OA, based on individual regions or countries, have been mainly reported (32, 33), and its global epidemiological trend is not yet elucidated. In addition, unlike previous GBD studies that only focused on the burden of the knee and hip OA, GBD 2019 study estimated the burden of hand OA, which contributed to overall OA burden estimates (34). Here, the 2019 GBD study was used to demonstrate the global, regional, and country incidence and DALYs related to hand OA and to explore their corresponding up-to-date trends and survival patterns over the recent three decades. Furthermore, the disease burden of hand OA was also explored based on sex, age, and SDI.

In this study, we observed that although the incident cases and DALYs of hand OA increased in the recent 30 years, their corresponding ASRs exhibited a decreasing trend. This trend is likely accounted for by effective diagnosis and treatment strategies. Meanwhile, although the burden of hand OA decreased at the global level over time, the ASIR in a majority of regions and countries had an increasing trend; so was also the case with the ASR of DALYs. Therefore, diagnoses, preventive measures, management, and therapy of hand OA should be prioritized based on different regions and countries, especially the USA, Iceland, and the Russian Federation, where the ASIRs and the ASR of DALYs are relatively high. The hand OA burden frequently includes direct costs of some treatments, such as imaging tests, pharmacological treatment, and surgery, and indirect costs due to the loss of productivity and early retirement (35–37). The total healthcare costs of hand OA may be underestimated, and national expenses and personal out-of-pocket costs for patients with hand OA most likely exceed the direct medical costs, implying an even worse actual burden of hand OA. Thus, policymakers in those regions and countries with an increasing burden of hand OA should train more medical professionals (physiotherapists, rheumatologists, and orthopedic surgeons) and enact comprehensive programs including recommended educational, behavioral, physical, psychosocial, mind-body, and pharmacologic interventions.

Consistent with previous studies (32, 38), the hand OA burden was higher in female patients than in male patients. Patients aged between 40 and 60 years were found to have a higher incidence of hand OA, especially female patients, and this trend eventually decreased in the elderly population. Another study observed a similar pattern (39). This incidence trend in female patients may be caused by structural or inflammatory changes during menopause or even possible changes in pain sensitivity (39, 40). Nonetheless, the cause of the occurrence of this phenomenon in male patients remains unclear. Therefore, this female group with hand OA should be chosen as the target population for the prevention, management, and treatment.

The estimated annual percentage change was positively associated with ASIR, indicating that the increase in hand OA was more rapid in regions or countries with high ASIR than in those with low ASIR. The ASIR also increased with the value of SDI. As reported, the USA, Iceland, and the Russian Federation had the highest ASIR; this differential distribution was caused by racial differences and genetic, environmental, anatomical, and biomechanical features (41).

Although DALYs increased at the global level in 2019, the ASR of DALYs also decreased year after year. A downward trend of age-standardized DALYs in hand OA from 1990 to 2019 coincided with early diagnosis and advanced treatment guidelines (5, 42, 43). Moreover, a distinct positive correlation was also observed between variations of the age-standard DALY rate and the baseline of the age-standardized DALY rate in 1990. This changing trend in 1990 is more significant in countries with higher age-standardized DALY rates. These findings could be considered because countries with higher age-standardized DALY rates had lower trends of hand OA. Therefore, preventive strategies are the top priority due to their role in public health, and efforts based on the population-level registration and disease surveillance systems to monitor burden trends in hand OA and its risk factors are necessary.

Despite high-quality assessment of the disease burden of hand OA in GBD studies, several shortcomings are inevitable. First, only limited information about hand OA could be extracted from the 2019 GBD database study, and data on mortality and risk factors of hand OA could not be obtained. The changing trend of mortality due to hand OA over the recent three decades and its risk factors are not yet determined. Second, the data were collected from different countries and territories, with varying diagnoses, screening standards, and the monitoring system of hand OA; therefore, the quality of the data included was uneven and, consequently, influenced the accuracy of the calculated results. Scarce data of some areas or some years obscured the real situation of a country. Third, specific types of hand OA are still unknown; the epidemiological trend of each type of hand OA needs to be further examined.

## Conclusion

In brief, according to this study, there was a decline in the global disease burden of hand OA from 1990 to 2019. However, due to the global population explosion and the aging tendency of the general population in recent decades, the incidence rate of patients with hand OA has risen rapidly. Thus, hand OA remains a globally prevalent public health concern and continues to take a huge toll on healthy lives. The prevalence characteristic of hand OA revealed preponderance in elderly female patients and high SDI regions. Hence, enhancing public awareness of the modifiable aetiological factors and allocating medical resources based on epidemiological variations of hand OA are essential to mitigate the global burden of hand OA.

## Data availability statement

The original contributions presented in the study are included in the article/Supplementary material, further inquiries can be directed to the corresponding authors.

## Author contributions

AC and PC conceptualized and designed the current study. JW, XQ, ZH, ZZ, PC, and AC were involved in data analysis and interpretation. ZH and ZZ contributed to the collection of relevant literature. JW and XQ downloaded data from GHDx and participated in the drafting and revising critically for the important intellectual content of the article and made significant contributions to the completion of the final version of this article. All authors listed are responsible for the reliability of the data sources, the accuracy of the data collation process and analysis, and had no doubts about authorship, and approved of publishing the final version.
