# Supplementary material for: Epidemiological trends of hand osteoarthritis from 1990 to 2019: Estimates from the 2019 Global Burden of Disease study
Source: Front Med (Lausanne). 2022 Dec 12;9:922321. doi: 10.3389/fmed.2022.922321 (PMC9790907; doi:10.3389/fmed.2022.922321)
Supplement: Supplementary file 1 [file Data_Sheet_1.pdf]

Epidemiological Trends of Hand Osteoarthritis at the Global, Regional and National Levels: Findings from the 2019 Global Burden of Disease Study

Author names: Junlai Wan[1†], Xiaoyuan Qian[2†], Zhiyi He[1], Ziqing Zhu[1], Peng Cheng[1*], Anmin Chen[1*]

[1]Author affiliation
Department of Orthopedics, Tongji Hospital, Tongji Medical College, Huazhong University of Science and Technology, Wuhan 430030, China
[2]Author Affiliation
Department of Urology, Tongji Hospital, Tongji Medical College, Huazhong University of Science and Technology, Wuhan 430030, China

*Correspondence:
Anmin Chen, E-mail: anminchen@hust.edu.cn
Address: Department of Orthopedics, Tongji Hospital, 1095 Jiefang Avenue, Wuhan, Hubei Province, China.
Peng Cheng, E-mail: chengpeng2015@tjh.tjmu.edu.cn
Address: Department of Orthopedics, Tongji Hospital, 1095 Jiefang Avenue, Wuhan, Hubei Province, China.

† Junlai Wan and Xiaoyuan Qian contributed equally to this work.

Supplementary Tables

Supplementary Table S1 Three countries with the largest and lowest number of incidence and DALYs.

Supplementary Table S2. Three regions with the largest and lowest number of incidence and DALYs.

Supplementary Table S3. The incident cases and age-standardized incidence rate of hand osteoarthritis in 1990 and 2019, and its temporal trends from 1990 to 2019.

Supplementary Table S4. Age distribution of incidence (per 100,000) for hand osteoarthritis in different countries in 2019.

Supplementary Table S5. The DALYs and age-standardized DALYs rate of hand osteoarthritis in 1990 and 2019, and its temporal trends from 1990 to 2019.

Supplementary Table S6. Age distribution of DALYs rate (per 100,000) for hand osteoarthritis in different countries in 2019.

Supplementary Figures

Supplementary figure 1. The incident cases (A), age-standardized incidence (B), and age-standardized DALYs (C) rates of hand osteoarthritis between 1990 and 2019 both males and females.

Supplementary figure 2 The proportion of different ages in incidence of hand osteoarthritis by years.

Supplementary figure 3. Distribution of different ages in incidence of hand osteoarthritis in global population.

Supplementary figure 4. Distribution of different ages in incidence of hand osteoarthritis in female subjects.

Supplementary figure 5. Distribution of different ages in incidence of hand osteoarthritis in male subjects.

Supplementary figure 6. The age standardized incidence (A) and DALY (B) rates of hand osteoarthritis per 100,000 population among regions based on SDI in 2019.

Supplementary figure 7. Distribution of different ages in DALYs of hand osteoarthritis in global level.

Supplementary figure 8. Distribution of different ages in DALYs of hand osteoarthritis in females.

Supplementary Figure 9. Distribution of different ages in DALYs of hand osteoarthritis in males.

Supplementary figure 10. The ratio of female to male DALYs among different age groups in global. SDI, socio-demographic index.

Supplementary Tables

Supplementary Table S1 Three countries with the largest and lowest number of incidence and DALYs.

Supplementary Table S1 Three countries with the largest and lowest number of incidence and DALY.

Measures	Top three countries			Bottom three countries		
2019ASR (per 100,000 people)						
ASIR	United States of America 281.4	Iceland 280.51	Russian Federation 268.42	Maldives 6.65	Timor-Leste 7.05	Malaysia 7.05
Age Standardized DALY Rate	Iceland 179.59	Russian Federation 178	United States of America 176.31	Maldives 3.52	Timor-Leste 3.65	Malaysia 3.66
1990-2019 increase times						
Incidence(cases)	United Arab Emirates 918.55	Qatar 730.65	Bahrain 496.38	Latvia -16.59	Estonia -4.71	Bulgaria -4.48
DALY(Year)	United Arab Emirates 898.62	Qatar 779.49	Bahrain 503.57	Latvia 1.13	Ukraine 5.35	Bulgaria 10.42
EAPC						
Incidence	Spain 1.80	Japan 1.57	Greece 0.83	Russian Federation -1.09	Saudi Arabia -0.58	Israel -0.55
DALY	Spain 1.88	Japan 1.69	Mexico 0.86	Russian Federation -1.11	Saudi Arabia -0.71	Israel -0.54

ASIR: age-standardized incidence; DALYs: disability adjusted life-years; EAPCs: estimated annual percentage changes.

Supplementary Table S2. Three regions with the largest and lowest number of incidence and DALYs.

Supplementary Table S2 Three regions with the largest and lowest number of incidence and DALYs.

Measure	Top three regions			Bottom three regions		
2019ASR (per 100,000 people)						
ASIR	High-income North America 275.59	Eastern Europe 215.56	High SDI 177.1	Southeast Asia 8.15	Oceania 16.25	Caribbean 41.15
Age Standardized DALY Rate	High-income North America 173.29	Eastern Europe 141.05	High SDI 112.44	Southeast Asia 4.21	Oceania 8.72	Caribbean 24.23
1990-2019 increase in the number of cases/years (-fold)						
Incidence(cases)	Central Latin America 1.85	North Africa and Middle East 1.82	Andean Latin America 1.67	Eastern Europe 0.07	Central Europe 0.21	Western Europe 0.41
DALY(Year)	Central Latin America 2.19	Andean Latin America 1.93	East Asia 1.78	Eastern Europe 0.15	Central Europe 0.39	Western Europe 0.53
EAPCs						
Incidence	High-income Asia Pacific 1.4	Central Latin America 0.57	High SDI 0.53	High-middle SDI -1.01	Eastern Europe -0.82	East Asia -0.26
DALY	High-income Asia Pacific 1.52	Central Latin America 0.61	High SDI 0.49	High-middle SDI -1.07	Eastern Europe -0.81	Overall -0.35

Supplementary Table S3. The incident cases and age-standardized incidence rate of hand osteoarthritis in 1990 and 2019, and its temporal trends from 1990 to 2019.

Supplementary Table S3. The incident cases and age-standardized incidence rate of Osteoarthritis hand in 1990 and 2019, and its temporal trends from 1990 to 2019.

Nation	Sex	Incident cases No. (95% UI)		change absolute number (%)	ASIR per 100,000 No. (95% UI)		1990-2019 EAPC No. (95% CI)
		1990	2019		1990	2019	
Afghanistan	Both	37.48 [27.58-50.57]	93.96 [67.64-129.25]	150.69	48.33 [36.24-64.47]	48.05 [36.06-64.16]	-0.02 [-0.06 to 0.02]
Albania	Both	12.82 [9.61-17.27]	19.84 [14.88-27.34]	54.76	53.11 [40.05-71.93]	53.33 [40.1-72.31]	0.01 [0.01 to 0.01]
Algeria	Both	68.12 [51.49-92.05]	195.42 [145.77-262.57]	186.88	48.24 [36.18-64.5]	47.72 [35.83-63.72]	-0.04 [-0.05 to -0.04]
American Samoa	Both	0.04 [0.03-0.06]	0.09 [0.06-0.12]	125	16.17 [12.01-21.91]	16.46 [12.19-22.42]	0.07 [0.06 to 0.08]
Andorra	Both	1.32 [1.01-1.71]	2.99 [2.18-3.98]	126.52	219.56 [164.56-287.21]	217.34 [162.73-285.01]	0.71 [0.46 to 0.96]
Angola	Both	46.23 [34.85-60.95]	140.11 [105.29-185.37]	203.07	81.44 [62.14-106.78]	83.05 [63.42-108.91]	0.07 [0.07 to 0.07]
Antigua and Barbuda	Both	0.2 [0.16-0.26]	0.46 [0.34-0.61]	130	41.66 [31.53-55.23]	41.37 [31.32-54.93]	-0.03 [-0.04 to -0.02]
Argentina	Both	509.79 [384.17-673.11]	788.57 [595.12-1035.14]	54.69	159.83 [120.76-211.05]	160.15 [121-211.36]	0.01 [0 to 0.01]
Armenia	Both	30.93 [23.06-41.81]	39.51 [29.51-53.3]	27.74	102.2 [77.18-135.49]	103.01 [77.78-136.86]	0.02 [0.02 to 0.03]
Australia	Both	291.47 [224.26-383.92]	539.18 [408.58-724.89]	84.99	160.86 [121.75-214.68]	170.1 [128.92-227.38]	-0.14 [-0.28 to 0]
Austria	Both	188.8 [142.99-247.26]	263.77 [195.47-352.14]	39.71	202.43 [152.85-265.47]	202.65 [153.17-266.04]	0.69 [0.46 to 0.91]
Azerbaijan	Both	64.21 [47.32-86.42]	136.98 [100.35-182.76]	113.33	114.24 [85.73-150.13]	113.46 [84.95-149.21]	0.31 [0.11 to 0.51]
Bahamas	Both	0.75 [0.57-1.01]	1.85 [1.38-2.48]	146.67	41.65 [31.51-55.2]	41.52 [31.44-55.06]	-0.01 [-0.01 to -0.01]
Bahrain	Both	1.38 [1.01-1.84]	8.23 [5.98-11.26]	496.38	44.28 [33.17-59.23]	41.9 [31.37-56.41]	-0.2 [-0.23 to -0.18]
Bangladesh	Both	231.93 [173.83-309.47]	610.8 [456.9-820.1]	163.36	41.29 [31.17-55.13]	42.48 [32.08-56.75]	0.11 [0.09 to 0.13]
Barbados	Both	1.01 [0.77-1.32]	1.8 [1.37-2.39]	78.22	41.99 [31.78-55.58]	41.54 [31.46-55.1]	-0.03 [-0.04 to -0.03]
Belarus	Both	137.16 [102.76-182.38]	153.13 [115.35-203.67]	11.64	111.88 [84.86-146.13]	111.39 [84.49-145.22]	0 [-0.01 to 0]
Belgium	Both	214.95 [160.95-280.22]	277.14 [203.82-365.69]	28.93	173.4 [130.01-228.03]	173.3 [129.9-227.7]	0.38 [0.26 to 0.51]
Belize	Both	0.4 [0.3-0.53]	1.37 [1.02-1.85]	242.5	40.25 [30.5-53.63]	40.72 [30.88-54.16]	0.04 [0.04 to 0.05]
Benin	Both	19.84 [15.2-26]	56.25 [42.58-74.14]	183.52	82.83 [63.13-108.59]	82.46 [62.91-108.15]	-0.01 [-0.02 to -0.01]

Bermuda	Both	0.27 [0.21-0.36]	0.44 [0.33-0.58]	62.96	41.51 [31.4-55.03]	41.1 [31.12-54.54]	-0.03 [-0.04 to -0.02]
Bhutan	Both	1.32 [0.99-1.76]	2.7 [2.03-3.6]	104.55	42.23 [32.06-55.97]	41.91 [31.71-55.76]	-0.03 [-0.04 to -0.01]
Bolivia (Plurinational State of)	Both	22.98 [17.36-30.58]	58.81 [44.4-78.49]	155.92	59.44 [45.18-79.56]	58.9 [44.8-78.84]	-0.03 [-0.03 to -0.02]
Bosnia and Herzegovina	Both	25.35 [18.75-34.85]	27.46 [20.62-37.59]	8.32	53.63 [40.51-72.74]	53.44 [40.28-72.49]	-0.01 [-0.01 to -0.01]
Botswana	Both	7.11 [5.42-9.25]	20.04 [15.14-25.87]	181.86	97.37 [74.63-125.9]	97.27 [74.55-126.02]	0 [0 to 0.01]
Brazil	Both	476.26 [356.24-634.33]	1150.96 [873.7-1526.19]	141.67	46.23 [35.3-61.2]	46.51 [35.53-61.55]	0.02 [0.02 to 0.02]
Brunei Darussalam	Both	2.52 [1.92-3.31]	7.95 [5.97-10.7]	215.48	157.47 [120-208.89]	164.21 [124.75-217.78]	0.18 [0.16 to 0.2]
Bulgaria	Both	62.03 [46.14-84.55]	59.25 [44.62-80.17]	-4.48	53.4 [40.22-72.44]	53.46 [40.33-72.5]	0 [0 to 0.01]
Burkina Faso	Both	43.53 [33.13-57.24]	105.7 [79.91-138.6]	142.82	83.05 [63.34-108.92]	83.27 [63.53-109.17]	0.01 [0.01 to 0.01]
Burundi	Both	23.86 [18.3-31.29]	53.32 [40.39-70.16]	123.47	83.25 [63.47-109.09]	80.73 [61.58-105.85]	-0.12 [-0.12 to -0.11]
Cabo Verde	Both	1.63 [1.26-2.15]	4.13 [3.15-5.44]	153.37	85.27 [64.92-111.73]	82.23 [62.86-107.78]	-0.13 [-0.15 to -0.12]
Cambodia	Both	3.77 [2.64-5.25]	9.71 [6.9-13.63]	157.56	7.58 [5.56-10.22]	7.54 [5.54-10.13]	-0.02 [-0.03 to -0.01]
Cameroon	Both	47.58 [36.12-62.68]	140.11 [106.29-184.22]	194.47	82.11 [62.66-107.66]	81.94 [62.55-107.43]	-0.01 [-0.01 to 0]
Canada	Both	672 [507.52-883.37]	1132.89 [829-1529.83]	68.58	225.53 [168.4-298.91]	226.8 [169.36-300.5]	0.02 [0.02 to 0.02]
Central African Republic	Both	13.02 [9.86-17.21]	26.93 [20.19-35.61]	106.84	82.91 [63.29-108.64]	82.42 [62.9-108.09]	-0.03 [-0.03 to -0.02]
Chad	Both	26.63 [20.32-34.83]	62.95 [47.59-82.82]	136.39	82.92 [63.18-108.68]	81.03 [61.81-106.28]	-0.09 [-0.1 to -0.08]
Chile	Both	179.83 [136.05-236.66]	364.65 [272.13-486.67]	102.77	160.79 [121.43-212.4]	160.2 [121.03-211.62]	-0.01 [-0.01 to 0]
China	Both	4384.51 [3271.47-5925.27]	10979.65 [8151.05-14824.96]	150.42	45.66 [34.52-61.37]	52.71 [39.69-70.52]	-0.27 [-0.67 to 0.13]
Colombia	Both	98.02 [73.93-131.82]	247.58 [185.56-332.42]	152.58	46.18 [34.95-61.71]	46.74 [35.34-62.46]	0.05 [0.04 to 0.05]
Comoros	Both	2.12 [1.61-2.78]	4.85 [3.68-6.38]	128.77	82.24 [62.74-107.85]	82.27 [62.85-107.89]	-0.01 [-0.02 to 0]
Congo	Both	11.01 [8.4-14.47]	32.52 [24.34-42.98]	195.37	83.2 [63.56-109.1]	81.58 [62.2-107.04]	-0.08 [-0.09 to -0.08]
Costa Rica	Both	9.06 [6.89-12.13]	24.63 [18.46-33.13]	171.85	46.15 [34.93-61.67]	46.66 [35.29-62.36]	0.04 [0.04 to 0.04]
Cote d'Ivoire	Both	49.71 [37.55-65.45]	132.78 [100.08-174.76]	167.11	80.32 [61.27-105.3]	80.38 [61.33-105.43]	0 [-0.01 to 0.01]
Croatia	Both	36.99 [27.25-48.86]	35.5 [26.71-48.48]	-4.03	58.35 [43.91-77.1]	53.45 [40.28-72.49]	-0.34 [-0.43 to -0.25]
Cuba	Both	41.85 [31.52-55.91]	71.32 [53.13-96.23]	70.42	40.81 [30.91-54.27]	41.04 [31.04-54.5]	0.02 [0.02 to 0.02]
Cyprus	Both	14.85 [11.27-19.4]	32.13 [24.27-41.86]	116.36	186.93 [139.71-245.76]	186.33 [139.18-244.68]	0.45 [0.3 to 0.6]

Czechia	Both	68.17 [51.35-90.9]	87.46 [66.11-117.33]	28.3	53.68 [40.52-72.85]	53.35 [40.21-72.32]	-0.02 [-0.02 to -0.02]
Democratic People's Republic of Korea	Both	90 [65.84-123.06]	157.82 [117.83-216.03]	75.36	46.28 [34.77-62.19]	46.51 [34.93-62.42]	0.02 [0.01 to 0.02]
Democratic Republic of the Congo	Both	171.55 [130.11-226.62]	419.43 [316.96-552.71]	144.49	83.18 [63.35-109.03]	82.31 [62.86-107.93]	-0.04 [-0.05 to -0.04]
Denmark	Both	129.8 [99.73-165.86]	147.03 [110.13-196.77]	13.27	206.04 [158.05-264.71]	184.8 [138.19-244.75]	-0.05 [-0.15 to 0.05]
Djibouti	Both	1.91 [1.43-2.51]	7.75 [5.81-10.26]	305.76	80.81 [61.64-105.94]	80.15 [61.16-105.16]	-0.04 [-0.05 to -0.03]
Dominica	Both	0.25 [0.19-0.32]	0.34 [0.25-0.45]	36	42.13 [31.79-55.78]	40.29 [30.49-53.74]	-0.17 [-0.2 to -0.14]
Dominican Republic	Both	17.69 [13.33-23.7]	41.31 [31.2-55.34]	133.52	40.91 [31.01-54.37]	40.8 [30.88-54.25]	-0.02 [-0.03 to -0.02]
Ecuador	Both	51.22 [38.55-67.81]	151.27 [121.87-184.61]	195.33	80.82 [60.79-106.73]	92.4 [74.22-112.66]	0.63 [0.51 to 0.75]
Egypt	Both	175.61 [130.9-236.62]	382.55 [284.05-514.86]	117.84	47.71 [35.83-63.67]	46.83 [35.09-62.59]	-0.06 [-0.07 to -0.06]
El Salvador	Both	15.12 [11.43-20.2]	27.91 [21.09-37.33]	84.59	46.65 [35.27-62.33]	47.68 [35.95-63.73]	0.08 [0.08 to 0.08]
Equatorial Guinea	Both	2.08 [1.58-2.76]	6.06 [4.59-7.96]	191.35	83.73 [63.87-109.8]	83.91 [64.02-109.95]	0 [-0.02 to 0.02]
Eritrea	Both	12.81 [9.62-16.97]	34.29 [25.77-45.24]	167.68	83.54 [63.82-109.5]	82.86 [63.34-108.56]	-0.03 [-0.04 to -0.02]
Estonia	Both	21.43 [16.1-28.24]	20.42 [15.6-27.09]	-4.71	112.04 [85.02-146.24]	110.1 [83.49-143.48]	-0.06 [-0.07 to -0.05]
Eswatini	Both	4.06 [3.06-5.27]	7.77 [5.91-10.04]	91.38	97.32 [74.69-125.79]	97.77 [75.02-126.67]	0.02 [0.01 to 0.02]
Ethiopia	Both	231.52 [174.47-304.05]	502.37 [378.87-661.38]	116.99	88.26 [67.06-115.78]	88.37 [67.18-115.98]	0.02 [0.01 to 0.02]
Fiji	Both	0.75 [0.54-1.02]	1.39 [1-1.94]	85.33	16.41 [12.19-22.32]	16.46 [12.21-22.34]	0 [0 to 0.01]
Finland	Both	63.79 [48.76-84.41]	78.58 [59.6-105.47]	23.19	101.32 [76.88-134.49]	99.83 [75.8-132.45]	-0.04 [-0.05 to -0.02]
France	Both	857.52 [652.33-1139.54]	1167.97 [890.54-1571.06]	36.2	127.07 [95.95-168.77]	127.29 [96-169.01]	0.03 [0.02 to 0.04]
Gabon	Both	5.04 [3.84-6.62]	11.43 [8.65-15.1]	126.79	82.6 [63.05-108.26]	82.29 [62.78-107.87]	-0.02 [-0.03 to 0]
Gambia	Both	3.9 [2.96-5.12]	10.46 [7.93-13.74]	168.21	80.04 [60.98-105]	81.76 [62.33-107.23]	0.09 [0.07 to 0.1]
Germany	Both	953.51 [719.36-1276.31]	1152.29 [881.88-1541.49]	20.85	88.22 [66.51-117.2]	87.24 [67.22-115.95]	-0.03 [-0.04 to -0.03]
Ghana	Both	69.52 [52.84-91.39]	184.79 [140-242.2]	165.81	82.03 [62.61-107.56]	83.29 [63.59-109.2]	0.06 [0.05 to 0.07]
Greece	Both	77.2 [58.37-104.98]	93.82 [70.93-126.92]	21.53	56.89 [43.36-76.13]	56.92 [43.37-76.2]	0.83 [0.49 to 1.17]
Greenland	Both	1.11 [0.83-1.46]	1.72 [1.23-2.35]	54.95	213.8 [159.55-283.34]	220.88 [165.16-292.93]	0.13 [0.12 to 0.14]
Grenada	Both	0.25 [0.19-0.33]	0.49 [0.36-0.65]	96	41.78 [31.6-55.37]	40.58 [30.67-54.04]	-0.1 [-0.11 to -0.1]
Guam	Both	0.15 [0.11-0.21]	0.31 [0.23-0.43]	106.67	16.14 [12.01-21.97]	16.31 [12.09-22.07]	0.03 [0.02 to 0.05]

Guatemala	Both	20.62 [15.43-27.69]	58.41 [44.4-78.28]	183.27	46.06 [34.82-61.6]	47.18 [35.62-63.06]	0.09 [0.09 to 0.09]
Guinea	Both	30.95 [23.71-40.47]	57.51 [43.83-75.27]	85.82	82.09 [62.64-107.68]	82.2 [62.69-107.79]	-0.01 [-0.01 to 0]
Guinea-Bissau	Both	4.33 [3.3-5.69]	8.79 [6.65-11.56]	103	82.92 [63.21-108.68]	83 [63.36-108.82]	0 [0 to 0]
Guyana	Both	1.87 [1.41-2.51]	2.98 [2.22-3.99]	59.36	40.97 [31.02-54.43]	41.16 [31.18-54.66]	0.01 [0.01 to 0.02]
Haiti	Both	15.86 [11.91-21.23]	36.74 [27.42-49.32]	131.65	40.94 [31.02-54.43]	41.5 [31.48-55.05]	0.05 [0.04 to 0.05]
Honduras	Both	11.25 [8.5-15.1]	32.84 [24.71-44.04]	191.91	46.17 [34.93-61.7]	46.62 [35.26-62.33]	0.03 [0.02 to 0.04]
Hungary	Both	72.25 [54.31-97.82]	81.12 [60.92-108.76]	12.28	53.74 [40.53-72.95]	53.61 [40.46-72.75]	-0.01 [-0.01 to -0.01]
Iceland	Both	6.82 [5.51-8.46]	12.27 [9.22-15.97]	79.91	277.81 [222.77-348.84]	280.51 [209.43-363.33]	0.1 [-0.89 to 1.1]
India	Both	2621.58 [1967.34-3529.07]	5977.16 [4538.71-8033.57]	128	46.82 [35.72-62.74]	47.6 [36.33-63.56]	0.06 [0.06 to 0.07]
Indonesia	Both	98.55 [71.28-135.52]	214.42 [155.76-296.29]	117.57	9 [6.79-11.94]	9 [6.8-11.92]	0 [0 to 0]
Iran (Islamic Republic of)	Both	238.34 [180.17-315.18]	685.06 [517.4-913.82]	187.43	73.95 [56.12-97.61]	75.64 [57.34-100.02]	0.08 [-0.08 to 0.24]
Iraq	Both	44.66 [33.36-59.91]	147.57 [109.71-199.54]	230.43	47.73 [35.85-63.63]	47.69 [35.83-63.62]	0.01 [0 to 0.01]
Ireland	Both	47.87 [36.51-63.09]	88.25 [66.13-117.29]	84.35	135.71 [102.19-180]	136.11 [102.52-180.41]	0.01 [0.01 to 0.01]
Israel	Both	81.66 [62.29-104.94]	182.03 [137.32-236.94]	122.91	188.36 [140.34-246.45]	187.87 [140.06-245.73]	-0.55 [-0.8 to -0.3]
Italy	Both	893.11 [678.47-1190.62]	1161.61 [876.69-1574.87]	30.06	119.26 [90.53-158.26]	118.29 [89.84-158.04]	0.21 [-0.03 to 0.46]
Jamaica	Both	6.74 [5.15-8.89]	12.28 [9.32-16.29]	82.2	41.12 [31.15-54.59]	40.89 [31-54.36]	-0.02 [-0.02 to -0.01]
Japan	Both	2117.77 [1608.42-2814.79]	2827.13 [2168.38-3662.13]	33.5	124.73 [95.67-165.14]	144.81 [109.46-188.98]	1.57 [1.21 to 1.94]
Jordan	Both	8.74 [6.46-11.85]	43.57 [32.13-59.28]	398.51	47.04 [35.43-62.94]	46.34 [34.77-61.94]	-0.05 [-0.07 to -0.02]
Kazakhstan	Both	144.9 [108.8-194.57]	204.39 [153.13-272.25]	41.06	103.23 [77.88-137.02]	102.79 [77.65-136.47]	-0.01 [-0.02 to 0]
Kenya	Both	93.44 [70.91-122.38]	277.33 [208.86-365.58]	196.8	88.97 [67.68-116.96]	89.12 [67.9-117.09]	0 [0 to 0.01]
Kuwait	Both	4.84 [3.49-6.51]	22.7 [16.39-31.02]	369.01	42.2 [31.74-56.69]	45.02 [33.83-60.31]	0.23 [0.19 to 0.27]
Kyrgyzstan	Both	32.07 [24.33-43.09]	58.71 [43.58-78.21]	83.07	102.44 [77.4-135.94]	102.32 [77.32-135.89]	0.01 [0 to 0.01]
Lao People's Democratic Republic	Both	1.62 [1.13-2.27]	3.6 [2.53-5]	122.22	7.33 [5.38-9.81]	7.21 [5.29-9.67]	-0.05 [-0.06 to -0.05]
Latvia	Both	37.25 [27.89-49.3]	31.07 [23.46-41.37]	-16.59	112.22 [85.15-146.52]	110.97 [84.16-144.68]	-0.03 [-0.04 to -0.03]
Lebanon	Both	12.36 [9.21-16.58]	26.12 [19.69-34.81]	111.33	48.22 [36.22-64.38]	49.65 [37.22-66.5]	0.1 [0.1 to 0.11]
Lesotho	Both	11.08 [8.48-14.42]	15.97 [12.2-20.88]	44.13	97 [74.4-125.46]	97.54 [74.83-126.5]	0.03 [0.02 to 0.03]

Liberia	Both	10.06 [7.7-13.16]	25.97 [19.41-34.41]	158.15	80.74 [61.56-105.89]	80.85 [61.64-106.1]	0 [0 to 0]
Libya	Both	10.51 [7.81-14.15]	33.88 [24.98-45.77]	222.36	45.71 [34.32-61.16]	47.34 [35.51-63.15]	0.14 [0.11 to 0.16]
Lithuania	Both	28.8 [21.79-39.07]	28.28 [21.32-38.32]	-1.81	66.79 [50.94-89.46]	66.16 [50.46-88.6]	0.2 [0.05 to 0.34]
Luxembourg	Both	6.57 [4.94-8.76]	11.97 [8.92-16.04]	82.19	135.78 [102.21-180.25]	135.22 [101.85-179.29]	-0.01 [-0.02 to -0.01]
Madagascar	Both	52.12 [39.76-68.3]	136.36 [102.4-180.14]	161.63	81.89 [62.38-107.34]	82.06 [62.62-107.59]	0.01 [0 to 0.01]
Malawi	Both	41.28 [31.45-54.09]	82.48 [62.55-108.21]	99.81	82.46 [62.96-108.14]	82.62 [63.12-108.35]	0.02 [0.02 to 0.03]
Malaysia	Both	7.58 [5.35-10.47]	20.12 [14.3-27.95]	165.44	7.14 [5.22-9.62]	7.05 [5.16-9.5]	-0.05 [-0.05 to -0.04]
Maldives	Both	0.07 [0.05-0.1]	0.26 [0.19-0.36]	271.43	6.59 [4.79-8.9]	6.65 [4.84-8.95]	0.06 [0.01 to 0.12]
Mali	Both	41.63 [31.76-54.57]	92.38 [70.12-121.29]	121.91	81.97 [62.52-107.49]	81.39 [62.05-106.8]	-0.03 [-0.04 to -0.03]
Malta	Both	5.8 [4.38-7.65]	8.49 [6.42-11.41]	46.38	136.89 [103.02-181.61]	135.55 [102.07-179.83]	-0.03 [-0.04 to -0.03]
Marshall Islands	Both	0.03 [0.02-0.04]	0.07 [0.05-0.1]	133.33	16.28 [12.1-22.05]	16.25 [12.08-22.21]	-0.01 [-0.01 to 0]
Mauritania	Both	9.65 [7.39-12.63]	21.19 [16.13-27.92]	119.59	82.23 [62.76-107.81]	82.16 [62.63-107.74]	0 [-0.01 to 0.01]
Mauritius	Both	0.58 [0.41-0.8]	1.26 [0.91-1.71]	117.24	7.36 [5.42-9.86]	7.29 [5.37-9.77]	-0.04 [-0.04 to -0.03]
Mexico	Both	604.7 [454.24-806.62]	1773.52 [1327.86-2369.34]	193.29	115.82 [86.64-153.93]	135.26 [101.86-178.7]	0.75 [0.6 to 0.91]
Micronesia (Federated States of)	Both	0.08 [0.06-0.11]	0.14 [0.1-0.19]	75	16.31 [12.13-22.25]	16.48 [12.2-22.43]	0.03 [0.02 to 0.04]
Mongolia	Both	11.98 [9.09-15.86]	34.38 [25.32-46.09]	186.98	100.46 [75.92-133.27]	102.34 [77.3-135.9]	0.07 [0.06 to 0.08]
Montenegro	Both	3.5 [2.62-4.79]	4.67 [3.5-6.37]	33.43	53.56 [40.4-72.65]	53.41 [40.25-72.43]	-0.01 [-0.01 to -0.01]
Morocco	Both	78.29 [58.64-105.31]	177.8 [132.24-238.55]	127.1	48.27 [36.23-64.42]	47.98 [36.04-64.04]	-0.03 [-0.03 to -0.03]
Mozambique	Both	65.49 [49.62-86.4]	127.47 [96.28-167.7]	94.64	82.66 [63.04-108.42]	83.15 [63.5-108.99]	0.01 [0.01 to 0.02]
Myanmar	Both	18.05 [12.77-25.24]	37.21 [26.55-52.2]	106.15	7.31 [5.38-9.79]	7.48 [5.5-10.06]	0.08 [0.08 to 0.08]
Namibia	Both	8.01 [6.12-10.36]	17.82 [13.5-23.05]	122.47	96.92 [74.27-125.34]	97.53 [74.74-126.26]	0.02 [0.02 to 0.03]
Nepal	Both	49.07 [36.4-65.51]	107.16 [80.45-143.39]	118.38	42.19 [31.97-56.22]	43.37 [32.86-57.76]	0.09 [0.08 to 0.1]
Netherlands	Both	179.99 [147.29-219.56]	247.03 [187.56-338.38]	37.25	102.44 [83.7-124.93]	97.78 [74.21-130.92]	-0.31 [-0.57 to -0.05]
New Zealand	Both	62.4 [47.7-81.1]	108.22 [81.39-144.24]	73.43	176.12 [133.4-231.2]	180.45 [136.38-237.37]	0.09 [0.08 to 0.09]
Nicaragua	Both	8.62 [6.51-11.62]	24.74 [18.5-33.15]	187.01	46.45 [35.15-62.07]	46.84 [35.39-62.57]	0.03 [0.03 to 0.04]
Niger	Both	32.47 [24.53-42.78]	87.14 [66.11-114.54]	168.37	80.99 [61.73-106.17]	82.49 [62.9-108.1]	0.06 [0.05 to 0.06]

Nigeria	Both	459.18 [347.44-602.06]	1100.45 [822.1-1453.15]	139.66	86.2 [65.54-112.95]	90.05 [68.57-118.52]	0.17 [0.15 to 0.19]
North Macedonia	Both	10.92 [8.2-14.85]	16.27 [12.16-22.24]	48.99	53.32 [40.11-72.32]	53.2 [40.06-72.11]	-0.01 [-0.01 to -0.01]
Northern Mariana Islands	Both	0.04 [0.03-0.06]	0.1 [0.07-0.14]	150	15.33 [11.42-20.68]	16.19 [12.04-22.07]	0.17 [0.14 to 0.19]
Norway	Both	94.3 [72.6-121.7]	139.02 [106.69-182.8]	47.42	191.97 [145.86-252.32]	191.23 [145.29-251.52]	0.17 [0.08 to 0.26]
Oman	Both	4.61 [3.39-6.17]	15.18 [10.93-20.46]	229.28	43.76 [32.95-58.6]	42.55 [31.98-57.01]	-0.14 [-0.21 to -0.07]
Pakistan	Both	297.65 [226.89-399.06]	652.1 [487.14-874.3]	119.08	46.5 [35.49-62.29]	47.11 [36.01-62.97]	0.04 [0.04 to 0.04]
Palestine	Both	4.88 [3.68-6.56]	15.61 [11.61-21.11]	219.88	49.9 [37.34-66.75]	47.73 [35.94-63.72]	-0.16 [-0.18 to -0.15]
Panama	Both	7.64 [5.79-10.22]	19.57 [14.68-26.14]	156.15	45.77 [34.63-61.15]	46.07 [34.87-61.56]	0.02 [0.02 to 0.02]
Papua New Guinea	Both	3.61 [2.63-4.95]	9.93 [7.19-13.66]	175.07	16.19 [12.06-21.95]	16.18 [12.04-21.94]	-0.01 [-0.01 to 0]
Paraguay	Both	10.21 [7.73-13.7]	24.91 [18.8-33.2]	143.98	40.82 [30.9-54.27]	40.8 [30.88-54.27]	0 [0 to 0]
Peru	Both	60.53 [45.77-81.18]	149.3 [112.31-199.82]	146.65	43.81 [32.99-58.52]	44.15 [33.22-59]	0.23 [0.1 to 0.35]
Philippines	Both	30.04 [21.92-41.08]	76.31 [55.71-104.4]	154.03	8.94 [6.74-11.87]	9.06 [6.83-11.99]	0.06 [0.05 to 0.06]
Poland	Both	252.96 [193.52-336.82]	344.27 [261.75-457.21]	36.1	59.51 [45.37-79.41]	59.27 [45.22-79.16]	-0.01 [-0.02 to -0.01]
Portugal	Both	175.43 [132.06-235.3]	235.47 [177.11-314.99]	34.22	145.82 [109.68-195.6]	145.57 [109.47-195.24]	0.18 [0.12 to 0.25]
Puerto Rico	Both	14.74 [11.09-19.61]	22.06 [16.58-29.1]	49.66	41.61 [31.54-55.18]	41.66 [31.56-55.22]	0 [-0.01 to 0.01]
Qatar	Both	1.24 [0.88-1.71]	10.3 [7.29-14.22]	730.65	39.48 [29.91-52.89]	37.63 [28.19-50.68]	-0.26 [-0.29 to -0.22]
Republic of Korea	Both	800.58 [603.97-1043.62]	1662.25 [1242.33-2193.67]	107.63	200.8 [152.26-260.34]	196.59 [149.15-256.03]	0.58 [0.36 to 0.79]
Republic of Moldova	Both	51.25 [38.72-67.49]	57.8 [43.55-76.3]	12.78	111.81 [84.82-145.69]	110.87 [84.11-144.5]	-0.02 [-0.03 to -0.02]
Romania	Both	147.58 [110.59-202.7]	158.95 [119.82-214.38]	7.7	53.5 [40.34-72.57]	53.46 [40.33-72.51]	0 [0 to 0]
Russian Federation	Both	4905.24 [3756.42-6358.96]	5309.17 [4054.88-6801.65]	8.23	287.81 [221.26-368.56]	268.42 [204.66-347.13]	-1.09 [-1.41 to -0.76]
Rwanda	Both	30.07 [23.02-39.51]	69.01 [52.59-90.91]	129.5	83.54 [63.67-109.45]	83.69 [63.93-109.68]	0.01 [0 to 0.02]
Saint Lucia	Both	0.36 [0.27-0.48]	0.92 [0.69-1.23]	155.56	41.68 [31.54-55.2]	40.95 [31.01-54.43]	-0.06 [-0.07 to -0.06]
Saint Vincent and the Grenadines	Both	0.28 [0.22-0.37]	0.55 [0.42-0.73]	96.43	41.42 [31.33-54.92]	40.24 [30.52-53.6]	-0.1 [-0.11 to -0.09]
Samoa	Both	0.15 [0.11-0.21]	0.26 [0.19-0.36]	73.33	16.42 [12.2-22.32]	16.27 [12.06-22.06]	-0.04 [-0.04 to -0.04]
Sao Tome and Principe	Both	0.55 [0.42-0.72]	1.21 [0.91-1.6]	120	82.52 [62.94-108.2]	81.76 [62.43-107.22]	-0.04 [-0.05 to -0.03]
Saudi Arabia	Both	102.47 [75.85-135.86]	427 [311.32-574.16]	316.71	106.69 [80.45-139.09]	107.25 [80.77-139.97]	-0.58 [-0.81 to -0.34]

Senegal	Both	32.7 [24.96-42.99]	78.45 [59.74-103.09]	139.91	81.98 [62.51-107.48]	82.43 [62.88-108.06]	0.02 [0.02 to 0.02]
Serbia	Both	62.6 [46.43-85.63]	67.28 [50.3-91.1]	7.48	53.43 [40.24-72.49]	53.35 [40.18-72.36]	0 [0 to 0]
Seychelles	Both	0.04 [0.03-0.05]	0.08 [0.06-0.12]	100	7.35 [5.4-9.85]	7.14 [5.25-9.55]	-0.11 [-0.13 to -0.1]
Sierra Leone	Both	18.15 [13.87-23.84]	39.75 [30.15-52.18]	119.01	81.59 [62.23-107.01]	81.32 [62-106.68]	-0.02 [-0.03 to -0.01]
Singapore	Both	50.19 [38.32-66.66]	135.41 [102.98-179.06]	169.79	170.63 [129.63-226.53]	160.46 [122.17-211.42]	0.05 [-0.03 to 0.14]
Slovakia	Both	30.63 [23.14-41.06]	43.21 [32.52-58.32]	41.07	53.67 [40.45-72.86]	53.51 [40.36-72.57]	-0.01 [-0.01 to -0.01]
Slovenia	Both	12.8 [9.66-17.34]	17.66 [13.33-23.99]	37.97	53.77 [40.66-72.94]	53.28 [40.13-72.22]	-0.03 [-0.03 to -0.03]
Solomon Islands	Both	0.26 [0.19-0.36]	0.64 [0.46-0.88]	146.15	15.97 [11.91-21.75]	16.27 [12.08-22.15]	0.06 [0.06 to 0.06]
Somalia	Both	33.51 [25.12-44.23]	85.48 [63.85-112.91]	155.09	82.37 [62.9-108]	83.06 [63.51-108.85]	0.03 [0.02 to 0.04]
South Africa	Both	262.89 [199.31-340.92]	557.28 [421.01-726.49]	111.98	104.92 [80.12-137.24]	104.92 [80.12-137.23]	0 [0 to 0.01]
South Sudan	Both	24.05 [18.34-31.58]	46.6 [34.8-61.71]	93.76	79.4 [60.61-104.14]	81.47 [62.15-106.89]	0.09 [0.08 to 0.09]
Spain	Both	580.28 [440.11-764.05]	1202.84 [742.64-1959.33]	107.29	127.62 [96.91-167.26]	168.99 [105.07-275.48]	1.8 [1.42 to 2.19]
Sri Lanka	Both	8.56 [6.02-11.89]	18.63 [13.45-25.48]	117.64	7.09 [5.18-9.53]	7.39 [5.44-9.92]	0.15 [0.14 to 0.16]
Sudan	Both	52.61 [39.24-70.76]	119.58 [88.69-160.55]	127.3	47.44 [35.64-63.39]	47.11 [35.41-63.03]	-0.04 [-0.05 to -0.02]
Suriname	Both	1.19 [0.89-1.59]	2.66 [1.99-3.54]	123.53	41.09 [31.1-54.66]	41.16 [31.16-54.67]	0 [-0.01 to 0]
Sweden	Both	92 [69.91-121.95]	116.65 [88.39-155.42]	26.79	80.22 [60.97-106.88]	79.55 [60.5-105.9]	-0.24 [-0.65 to 0.17]
Switzerland	Both	116.91 [88.21-154.95]	175.26 [130.69-235.47]	49.91	136.04 [102.41-180.54]	135.6 [102.14-179.85]	-0.01 [-0.01 to -0.01]
Syrian Arab Republic	Both	30.54 [22.89-41.04]	73 [53.67-98.79]	139.03	47.37 [35.55-63.26]	47.85 [35.96-63.88]	0.02 [0 to 0.04]
Tajikistan	Both	30.47 [23.12-40.82]	74.26 [54.84-99.01]	143.72	100.67 [76.18-133.53]	100.93 [76.23-134.02]	0.03 [0.02 to 0.03]
Thailand	Both	28.71 [20.46-39.61]	74.72 [54.38-101.59]	160.26	7.16 [5.36-9.73]	7.31 [5.37-9.82]	0.1 [0.08 to 0.12]
Timor-Leste	Both	0.26 [0.18-0.36]	0.59 [0.42-0.81]	126.92	7.05 [5.17-9.47]	7.05 [5.18-9.51]	0.01 [0 to 0.01]
Togo	Both	14.63 [11.1-19.23]	44.65 [33.68-58.86]	205.19	83 [63.31-108.82]	83.2 [63.5-109.14]	0 [0 to 0.01]
Tonga	Both	0.1 [0.07-0.14]	0.14 [0.1-0.19]	40	16.62 [12.32-22.62]	16.58 [12.28-22.46]	-0.02 [-0.02 to -0.01]
Trinidad and Tobago	Both	3.7 [2.81-4.95]	7.36 [5.56-9.74]	98.92	40.96 [31-54.41]	40.73 [30.84-54.16]	-0.02 [-0.02 to -0.01]
Tunisia	Both	27.07 [20.4-36.37]	65.86 [49.26-88.21]	143.3	47.84 [35.91-63.84]	48.23 [36.23-64.33]	0.03 [0.02 to 0.03]
Turkey	Both	58.99 [43.61-79.68]	135.78 [101.05-183.8]	130.17	14.51 [10.82-19.61]	14.52 [10.83-19.63]	-0.13 [-0.3 to 0.05]

Turkmenistan	Both	16.18 [12.94-21.06]	39.1 [29-52.44]	141.66	74.31 [59.73-95.34]	80.24 [60.74-107.03]	0.3 [0.22 to 0.39]
Uganda	Both	66.97 [51.19-88.18]	166.9 [126.52-219.1]	149.22	82.04 [62.61-107.56]	83.14 [63.49-109]	0.04 [0.03 to 0.05]
Ukraine	Both	622.48 [473.35-846.66]	613.68 [467.86-825.64]	-1.41	93.66 [71.95-123.94]	93.56 [71.72-123.95]	0.24 [0.09 to 0.4]
United Arab Emirates	Both	4.69 [3.31-6.44]	47.77 [33.36-66.36]	918.55	39.56 [29.79-53.03]	37.63 [28.32-50.63]	-0.13 [-0.15 to -0.1]
United Kingdom	Both	923.63 [710.53-1226.57]	1239.8 [948.48-1681.29]	34.23	129.59 [98.49-172.1]	133.38 [101.72-177.72]	-0.06 [-0.39 to 0.27]
United Republic of Tanzania	Both	110.62 [84.61-144.98]	277.96 [210.18-365.33]	151.27	82.37 [62.82-107.96]	82.38 [62.86-108]	-0.01 [-0.01 to 0]
United States of America	Both	6686.83 [5209.1-8522.8]	12177.84 [9172.32-16019.87]	82.12	247.51 [189.48-318.79]	281.4 [216.27-364.83]	0.5 [0.22 to 0.79]
United States Virgin Islands	Both	0.43 [0.32-0.58]	0.64 [0.48-0.85]	48.84	41.42 [31.4-54.97]	41.71 [31.58-55.3]	0.02 [0.02 to 0.02]
Uruguay	Both	53.59 [40.52-71.13]	66.67 [50.59-88.26]	24.41	160.45 [121.21-211.77]	161.01 [121.62-212.49]	0.01 [0.01 to 0.01]
Uzbekistan	Both	126.33 [96.01-170.18]	309.56 [228.37-413.05]	145.04	101.61 [76.73-134.79]	101.94 [77.04-135.39]	0.02 [0.02 to 0.03]
Vanuatu	Both	0.12 [0.09-0.17]	0.31 [0.23-0.43]	158.33	15.96 [11.9-21.66]	16.19 [12.06-22.01]	0.06 [0.05 to 0.07]
Venezuela	Both	22.74 [16.8-31.07]	63.03 [47.03-86.08]	177.18	20.36 [15.24-27.73]	20.4 [15.28-27.78]	-0.03 [-0.05 to -0.01]
VietNam	Both	30.93 [22.1-42.45]	75.16 [53.78-104.66]	143	7.57 [5.56-10.13]	7.44 [5.49-9.95]	-0.07 [-0.07 to -0.07]
Yemen	Both	30.15 [22.48-40.42]	87.8 [65.24-117.02]	191.21	47.5 [35.81-63.4]	48.06 [36.03-64.14]	0.05 [0.04 to 0.05]
Zambia	Both	31.43 [23.87-41.52]	82.71 [62.15-109.2]	163.16	81.68 [62.26-107.04]	81.64 [62.31-107.12]	0 [-0.01 to 0.01]
Zimbabwe	Both	51.64 [39.46-67.29]	97.24 [73.24-125.94]	88.3	96.45 [73.96-124.71]	97.74 [74.9-126.64]	0.05 [0.04 to 0.06]
Afghanistan	Male	10.76 [7.8-15.05]	29.13 [20.13-41.57]	170.72	29 [21.46-39.78]	29 [21.46-39.78]	0 [0 to 0]
Albania	Male	6.14 [4.52-8.38]	8.52 [6.31-11.78]	38.76	48.07 [35.92-65.22]	48.07 [35.92-65.22]	0 [0 to 0]
Algeria	Male	20.26 [14.84-27.8]	59.81 [43.74-82.09]	195.21	29 [21.46-39.78]	29 [21.46-39.78]	0 [0 to 0]
American Samoa	Male	0.02 [0.01-0.02]	0.03 [0.02-0.04]	50	11.63 [8.56-15.76]	11.63 [8.56-15.76]	0 [0 to 0]
Andorra	Male	0.87 [0.66-1.12]	1.92 [1.39-2.56]	120.69	269.38 [200.55-351.69]	269.38 [200.55-351.69]	1.13 [0.76 to 1.5]
Angola	Male	18.58 [13.96-24.73]	50.73 [38.01-67.38]	173.04	63.23 [48.09-83.63]	63.23 [48.09-83.63]	0 [0 to 0]
Antigua and Barbuda	Male	0.06 [0.04-0.08]	0.14 [0.1-0.19]	133.33	26.98 [20.13-36.38]	26.98 [20.13-36.38]	0 [0 to 0]
Argentina	Male	127.13 [95.34-171.99]	196.49 [148.56-264.84]	54.56	83.05 [62.52-112.17]	83.05 [62.52-112.17]	0 [0 to 0]
Armenia	Male	9.27 [6.93-12.63]	11.46 [8.62-15.38]	23.62	65.64 [49.82-87.15]	65.64 [49.82-87.15]	0 [0 to 0]
Australia	Male	75.28 [57.03-100.91]	133.09 [100.83-181.44]	76.79	83.05 [62.52-112.17]	83.05 [62.52-112.17]	0 [0 to 0]

Austria	Male	108.61 [81.72-141.75]	155.49 [114.37-207.29]	43.16	243.74 [183.93-316.09]	243.74 [183.93-316.09]	1.09 [0.73 to 1.45]
Azerbaijan	Male	14.96 [11-20.58]	32.57 [23.91-44.35]	117.71	58.13 [43.59-78.19]	58.13 [43.59-78.19]	0.35 [0.13 to 0.58]
Bahamas	Male	0.23 [0.17-0.31]	0.56 [0.41-0.77]	143.48	26.98 [20.13-36.38]	26.98 [20.13-36.38]	0 [0 to 0]
Bahrain	Male	0.6 [0.42-0.83]	3.92 [2.76-5.5]	553.33	29 [21.46-39.78]	29 [21.46-39.78]	0 [0 to 0]
Bangladesh	Male	87.85 [63.94-117.73]	206.57 [151.67-283.12]	135.14	28.81 [21.3-39.3]	28.81 [21.3-39.3]	0 [0 to 0]
Barbados	Male	0.29 [0.22-0.39]	0.54 [0.4-0.74]	86.21	26.98 [20.13-36.38]	26.98 [20.13-36.38]	0 [0 to 0]
Belarus	Male	41.11 [30.89-55.23]	46.63 [34.86-62.06]	13.43	76.3 [57.89-101.15]	76.3 [57.89-101.15]	0 [0 to 0]
Belgium	Male	112.6 [83.53-146.52]	146.07 [107.25-192.83]	29.72	185.04 [137.94-241.65]	185.04 [137.94-241.65]	0.69 [0.47 to 0.92]
Belize	Male	0.14 [0.1-0.19]	0.45 [0.33-0.61]	221.43	26.98 [20.13-36.38]	26.98 [20.13-36.38]	0 [0 to 0]
Benin	Male	7.14 [5.49-9.48]	20.97 [15.81-27.73]	193.7	63.23 [48.09-83.63]	63.23 [48.09-83.63]	0 [0 to 0]
Bermuda	Male	0.08 [0.06-0.11]	0.14 [0.1-0.19]	75	26.98 [20.13-36.38]	26.98 [20.13-36.38]	0 [0 to 0]
Bhutan	Male	0.47 [0.34-0.64]	0.97 [0.71-1.31]	106.38	28.81 [21.3-39.3]	28.81 [21.3-39.3]	0 [0 to 0]
Bolivia (Plurinational State of)	Male	5.98 [4.43-8.15]	15.76 [11.67-21.53]	163.55	32.38 [23.91-43.67]	32.38 [23.91-43.67]	0 [0 to 0]
Bosnia and Herzegovina	Male	10.92 [7.96-14.87]	11.69 [8.69-15.97]	7.05	48.07 [35.92-65.22]	48.07 [35.92-65.22]	0 [0 to 0]
Botswana	Male	2.83 [2.15-3.71]	8.36 [6.28-11]	195.41	82.01 [62.18-108.33]	82.01 [62.18-108.33]	0 [0 to 0]
Brazil	Male	151.58 [111.42-204.5]	353.97 [261.89-481.39]	133.52	30.61 [22.83-41.03]	30.61 [22.83-41.03]	0 [0 to 0]
Brunei Darussalam	Male	0.4 [0.3-0.54]	1.16 [0.86-1.56]	190	52.77 [40.02-71.34]	52.77 [40.02-71.34]	0 [0 to 0]
Bulgaria	Male	26.72 [19.87-36.32]	24.99 [18.45-33.72]	-6.47	48.07 [35.92-65.22]	48.07 [35.92-65.22]	0 [0 to 0]
Burkina Faso	Male	15.26 [11.59-20.34]	37.41 [28.26-49.52]	145.15	63.23 [48.09-83.63]	63.23 [48.09-83.63]	0 [0 to 0]
Burundi	Male	8.53 [6.51-11.26]	22.23 [16.76-29.45]	160.61	63.23 [48.09-83.63]	63.23 [48.09-83.63]	0 [0 to 0]
Cabo Verde	Male	0.49 [0.38-0.65]	1.6 [1.2-2.12]	226.53	63.23 [48.09-83.63]	63.23 [48.09-83.63]	0 [0 to 0]
Cambodia	Male	0.81 [0.53-1.17]	2.18 [1.42-3.13]	169.14	3.82 [2.6-5.38]	3.82 [2.6-5.38]	0 [0 to 0]
Cameroon	Male	18.06 [13.7-23.99]	54.41 [41.09-71.76]	201.27	63.23 [48.09-83.63]	63.23 [48.09-83.63]	0 [0 to 0]
Canada	Male	240.19 [182-319.62]	413.42 [300.71-567.97]	72.12	162.77 [121.3-219.16]	162.77 [121.3-219.16]	0 [0 to 0]
Central African Republic	Male	4.76 [3.58-6.31]	10.33 [7.66-13.82]	117.02	63.23 [48.09-83.63]	63.23 [48.09-83.63]	0 [0 to 0]
Chad	Male	9.51 [7.29-12.62]	24.97 [18.92-33.13]	162.57	63.23 [48.09-83.63]	63.23 [48.09-83.63]	0 [0 to 0]

Chile	Male	43.49 [32.7-58.09]	91.29 [68.24-124.95]	109.91	83.05 [62.52-112.17]	83.05 [62.52-112.17]	0 [0 to 0]
China	Male	2215.64 [1639.56-3049.93]	5588.29 [4123.26-7743.92]	152.22	45.13 [33.7-61.13]	53.18 [39.92-72.42]	-0.96 [-1.63 to -0.29]
Colombia	Male	36.7 [27.4-49.61]	86.04 [64.53-116.48]	134.44	34.92 [26.18-47.08]	34.92 [26.18-47.08]	0 [0 to 0]
Comoros	Male	0.79 [0.6-1.05]	1.85 [1.4-2.46]	134.18	63.23 [48.09-83.63]	63.23 [48.09-83.63]	0 [0 to 0]
Congo	Male	3.95 [2.99-5.26]	12.98 [9.64-17.34]	228.61	63.23 [48.09-83.63]	63.23 [48.09-83.63]	0 [0 to 0]
Costa Rica	Male	3.4 [2.54-4.59]	8.64 [6.47-11.74]	154.12	34.92 [26.18-47.08]	34.92 [26.18-47.08]	0 [0 to 0]
Cote d'Ivoire	Male	21.31 [16.03-28.12]	57 [42.61-75.76]	167.48	63.23 [48.09-83.63]	63.23 [48.09-83.63]	0 [0 to 0]
Croatia	Male	16.01 [11.74-21.5]	14.93 [11.14-20.34]	-6.75	53.93 [40.09-71.23]	48.07 [35.92-65.22]	-0.45 [-0.57 to -0.32]
Cuba	Male	13.71 [10.24-18.61]	22.56 [16.66-30.93]	64.55	26.98 [20.13-36.38]	26.98 [20.13-36.38]	0 [0 to 0]
Cyprus	Male	8.27 [6.21-10.76]	17.56 [13.27-22.8]	112.33	213.52 [159.56-279.01]	213.52 [159.56-279.01]	0.81 [0.54 to 1.07]
Czechia	Male	28.29 [20.97-37.76]	37.88 [28.28-50.92]	33.9	48.07 [35.92-65.22]	48.07 [35.92-65.22]	0 [0 to 0]
Democratic People's Republic of Korea	Male	40.56 [29.41-56.57]	76.85 [56.39-106.17]	89.47	47.32 [35.44-64.81]	47.32 [35.44-64.81]	0 [0 to 0]
Democratic Republic of the Congo	Male	60.76 [46.12-80.94]	161.98 [121.82-215.12]	166.59	63.23 [48.09-83.63]	63.23 [48.09-83.63]	0 [0 to 0]
Denmark	Male	54.65 [40.88-70.76]	62 [46.38-82.83]	13.45	176.52 [132.22-228.89]	155.62 [117.38-204.18]	0.24 [0.05 to 0.43]
Djibouti	Male	0.81 [0.6-1.07]	3.29 [2.46-4.39]	306.17	63.23 [48.09-83.63]	63.23 [48.09-83.63]	0 [0 to 0]
Dominica	Male	0.07 [0.05-0.09]	0.12 [0.09-0.16]	71.43	26.98 [20.13-36.38]	26.98 [20.13-36.38]	0 [0 to 0]
Dominican Republic	Male	5.73 [4.24-7.83]	13.53 [10.07-18.46]	136.13	26.98 [20.13-36.38]	26.98 [20.13-36.38]	0 [0 to 0]
Ecuador	Male	11.75 [8.75-16.03]	32.27 [25.09-42.06]	174.64	37.81 [28.34-51.2]	40.83 [31.68-53.49]	0.37 [0.29 to 0.44]
Egypt	Male	54.11 [39.39-74.16]	124.08 [91.02-170.46]	129.31	29 [21.46-39.78]	29 [21.46-39.78]	0 [0 to 0]
El Salvador	Male	5.35 [4.02-7.2]	8.67 [6.53-11.68]	62.06	34.92 [26.18-47.08]	34.92 [26.18-47.08]	0 [0 to 0]
Equatorial Guinea	Male	0.71 [0.53-0.95]	2.15 [1.61-2.84]	202.82	63.23 [48.09-83.63]	63.23 [48.09-83.63]	0 [0 to 0]
Eritrea	Male	4.62 [3.45-6.17]	13.09 [9.73-17.52]	183.33	63.23 [48.09-83.63]	63.23 [48.09-83.63]	0 [0 to 0]
Estonia	Male	6.4 [4.81-8.54]	6.5 [4.9-8.62]	1.56	76.3 [57.89-101.15]	76.3 [57.89-101.15]	0 [0 to 0]
Eswatini	Male	1.62 [1.22-2.14]	3.13 [2.36-4.12]	93.21	82.01 [62.18-108.33]	82.01 [62.18-108.33]	0 [0 to 0]
Ethiopia	Male	92.6 [69.39-123.55]	200.01 [150.22-263.96]	115.99	68.95 [51.83-90.95]	68.95 [51.83-90.95]	0 [0 to 0]
Fiji	Male	0.26 [0.18-0.36]	0.48 [0.33-0.68]	84.62	11.63 [8.56-15.76]	11.63 [8.56-15.76]	0 [0 to 0]

Finland	Male	12.71 [9.53-17.1]	17.51 [12.97-23.71]	37.77	42.76 [32.03-57.53]	42.76 [32.03-57.53]	0 [0 to 0]
France	Male	301.13 [230.35-405.54]	411.07 [314.51-563.12]	36.51	91.83 [70.25-123.6]	91.83 [70.25-123.6]	0.05 [0.04 to 0.07]
Gabon	Male	1.89 [1.44-2.51]	4.3 [3.24-5.71]	127.51	63.23 [48.09-83.63]	63.23 [48.09-83.63]	0 [0 to 0]
Gambia	Male	1.68 [1.27-2.23]	4.06 [3.08-5.37]	141.67	63.23 [48.09-83.63]	63.23 [48.09-83.63]	0 [0 to 0]
Germany	Male	244.15 [181.6-332.3]	318.34 [239.1-428.97]	30.39	47.79 [36.18-64.08]	47.68 [36.27-63.49]	-0.01 [-0.01 to -0.01]
Ghana	Male	26.76 [20.15-35.59]	65.93 [49.54-87.4]	146.38	63.23 [48.09-83.63]	63.23 [48.09-83.63]	0 [0 to 0]
Greece	Male	22.8 [16.86-31.71]	27.7 [20.69-38.21]	21.49	34.57 [25.76-47.75]	34.57 [25.76-47.75]	1.2 [0.7 to 1.69]
Greenland	Male	0.47 [0.35-0.62]	0.7 [0.49-0.98]	48.94	162.77 [121.3-219.16]	162.77 [121.3-219.16]	0 [0 to 0]
Grenada	Male	0.07 [0.06-0.1]	0.16 [0.12-0.22]	128.57	26.98 [20.13-36.38]	26.98 [20.13-36.38]	0 [0 to 0]
Guam	Male	0.06 [0.04-0.08]	0.11 [0.08-0.16]	83.33	11.63 [8.56-15.76]	11.63 [8.56-15.76]	0 [0 to 0]
Guatemala	Male	7.73 [5.77-10.46]	19.48 [14.66-26.31]	152.01	34.92 [26.18-47.08]	34.92 [26.18-47.08]	0 [0 to 0]
Guinea	Male	11.59 [8.84-15.42]	21.22 [16.22-28.2]	83.09	63.23 [48.09-83.63]	63.23 [48.09-83.63]	0 [0 to 0]
Guinea-Bissau	Male	1.54 [1.18-2.05]	3.18 [2.4-4.19]	106.49	63.23 [48.09-83.63]	63.23 [48.09-83.63]	0 [0 to 0]
Guyana	Male	0.61 [0.45-0.82]	0.94 [0.69-1.29]	54.1	26.98 [20.13-36.38]	26.98 [20.13-36.38]	0 [0 to 0]
Haiti	Male	5.13 [3.78-7.02]	11.19 [8.24-15.33]	118.13	26.98 [20.13-36.38]	26.98 [20.13-36.38]	0 [0 to 0]
Honduras	Male	4.21 [3.15-5.69]	11.61 [8.69-15.72]	175.77	34.92 [26.18-47.08]	34.92 [26.18-47.08]	0 [0 to 0]
Hungary	Male	29.34 [21.68-39.58]	33.23 [24.72-44.52]	13.26	48.07 [35.92-65.22]	48.07 [35.92-65.22]	0 [0 to 0]
Iceland	Male	3.22 [2.6-3.93]	5.65 [4.14-7.47]	75.47	261.06 [209.13-321.77]	253.57 [185.28-331.57]	-0.11 [-1.19 to 0.98]
India	Male	951.8 [698.63-1303.99]	2035.78 [1502.27-2780.61]	113.89	32.45 [24.35-42.85]	32.45 [24.35-42.85]	0 [0 to 0]
Indonesia	Male	27.31 [17.74-39.12]	59.42 [38.56-85.85]	117.58	5.09 [3.46-7]	5.09 [3.46-7]	0 [0 to 0]
Iran (Islamic Republic of)	Male	64.32 [48.01-86.68]	177.58 [132.29-238.16]	176.09	38.73 [29.34-51.86]	38.97 [29.45-52.04]	-0.02 [-0.13 to 0.09]
Iraq	Male	13.92 [10.14-18.99]	45.84 [33.19-63.24]	229.31	29 [21.46-39.78]	29 [21.46-39.78]	0 [0 to 0]
Ireland	Male	19.32 [14.6-25.74]	35.21 [26.31-47.11]	82.25	110.27 [82.68-146.57]	110.27 [82.68-146.57]	0 [0 to 0]
Israel	Male	33.48 [25.31-43.46]	76.52 [57.72-100.18]	128.55	161.51 [120.46-213.24]	161.51 [120.46-213.24]	-0.67 [-0.98 to -0.36]
Italy	Male	326.54 [245.33-441.7]	409.07 [307.66-557.86]	25.27	91.78 [69.24-122.14]	86.34 [65.24-115.92]	0.09 [-0.19 to 0.38]
Jamaica	Male	2.13 [1.59-2.9]	3.96 [2.96-5.4]	85.92	26.98 [20.13-36.38]	26.98 [20.13-36.38]	0 [0 to 0]

Japan	Male	425.11 [316.62-577.64]	574.42 [431.16-776.53]	35.12	52.9 [39.91-71.31]	52.88 [39.9-71.28]	-0.13 [-0.18 to -0.08]
Jordan	Male	2.82 [2.03-3.91]	14.89 [10.7-20.69]	428.01	29 [21.46-39.78]	29 [21.46-39.78]	0 [0 to 0]
Kazakhstan	Male	41.5 [30.9-55.87]	59.66 [44.46-79.65]	43.76	65.64 [49.82-87.15]	65.64 [49.82-87.15]	0 [0 to 0]
Kenya	Male	36.34 [27.32-48.06]	107.67 [80.2-142.38]	196.29	68.95 [51.83-90.95]	68.95 [51.83-90.95]	0 [0 to 0]
Kuwait	Male	2.23 [1.58-3.08]	8.31 [5.86-11.69]	272.65	29 [21.46-39.78]	29 [21.46-39.78]	0 [0 to 0]
Kyrgyzstan	Male	9.45 [7.08-12.67]	17.55 [13.11-23.45]	85.71	65.64 [49.82-87.15]	65.64 [49.82-87.15]	0 [0 to 0]
Lao People's Democratic Republic	Male	0.4 [0.26-0.58]	0.93 [0.61-1.35]	132.5	3.82 [2.6-5.38]	3.82 [2.6-5.38]	0 [0 to 0]
Latvia	Male	11 [8.26-14.66]	9.49 [7.14-12.64]	-13.73	76.3 [57.89-101.15]	76.3 [57.89-101.15]	0 [0 to 0]
Lebanon	Male	3.61 [2.64-5.02]	7 [5.18-9.58]	93.91	29 [21.46-39.78]	29 [21.46-39.78]	0 [0 to 0]
Lesotho	Male	4.6 [3.49-6.09]	6.6 [5.01-8.63]	43.48	82.01 [62.18-108.33]	82.01 [62.18-108.33]	0 [0 to 0]
Liberia	Male	4.13 [3.15-5.47]	10.73 [7.92-14.37]	159.81	63.23 [48.09-83.63]	63.23 [48.09-83.63]	0 [0 to 0]
Libya	Male	3.78 [2.76-5.19]	10.81 [7.79-15.01]	185.98	29 [21.46-39.78]	29 [21.46-39.78]	0 [0 to 0]
Lithuania	Male	7.73 [5.64-10.59]	7.67 [5.62-10.56]	-0.78	41.27 [30.54-56.05]	41.27 [30.54-56.05]	0.2 [0.08 to 0.33]
Luxembourg	Male	2.62 [1.96-3.5]	4.97 [3.69-6.68]	89.69	110.27 [82.68-146.57]	110.27 [82.68-146.57]	0 [0 to 0]
Madagascar	Male	20.04 [15.33-26.63]	52.36 [39.23-69.53]	161.28	63.23 [48.09-83.63]	63.23 [48.09-83.63]	0 [0 to 0]
Malawi	Male	15.49 [11.77-20.48]	31.06 [23.21-41.36]	100.52	63.23 [48.09-83.63]	63.23 [48.09-83.63]	0 [0 to 0]
Malaysia	Male	2.02 [1.32-2.87]	5.51 [3.67-7.83]	172.77	3.82 [2.6-5.38]	3.82 [2.6-5.38]	0 [0 to 0]
Maldives	Male	0.02 [0.01-0.03]	0.09 [0.06-0.13]	350	3.82 [2.6-5.38]	3.82 [2.6-5.38]	0 [0 to 0]
Mali	Male	15.84 [12.05-21.06]	36.3 [27.54-48.12]	129.17	63.23 [48.09-83.63]	63.23 [48.09-83.63]	0 [0 to 0]
Malta	Male	2.21 [1.68-2.93]	3.49 [2.64-4.71]	57.92	110.27 [82.68-146.57]	110.27 [82.68-146.57]	0 [0 to 0]
Marshall Islands	Male	0.01 [0.01-0.02]	0.02 [0.02-0.03]	100	11.63 [8.56-15.76]	11.63 [8.56-15.76]	0 [0 to 0]
Mauritania	Male	3.64 [2.79-4.84]	7.95 [6.08-10.54]	118.41	63.23 [48.09-83.63]	63.23 [48.09-83.63]	0 [0 to 0]
Mauritius	Male	0.14 [0.09-0.2]	0.31 [0.2-0.44]	121.43	3.82 [2.6-5.38]	3.82 [2.6-5.38]	0 [0 to 0]
Mexico	Male	258.21 [193.58-345.71]	732.03 [551.03-977.15]	183.5	100.65 [76.11-134.46]	117.38 [88.79-155.99]	0.75 [0.59 to 0.92]
Micronesia (Federated States of)	Male	0.03 [0.02-0.04]	0.05 [0.03-0.07]	66.67	11.63 [8.56-15.76]	11.63 [8.56-15.76]	0 [0 to 0]
Mongolia	Male	3.87 [2.92-5.17]	10.34 [7.68-13.88]	167.18	65.64 [49.82-87.15]	65.64 [49.82-87.15]	0 [0 to 0]

Montenegro	Male	1.49 [1.1-2.04]	2 [1.49-2.73]	34.23	48.07 [35.92-65.22]	48.07 [35.92-65.22]	0 [0 to 0]
Morocco	Male	23.25 [17.06-31.76]	53.16 [38.94-73.09]	128.65	29 [21.46-39.78]	29 [21.46-39.78]	0 [0 to 0]
Mozambique	Male	23.81 [17.99-31.58]	45.82 [34.45-60.6]	92.44	63.23 [48.09-83.63]	63.23 [48.09-83.63]	0 [0 to 0]
Myanmar	Male	4.49 [2.91-6.44]	8.44 [5.57-12.14]	87.97	3.82 [2.6-5.38]	3.82 [2.6-5.38]	0 [0 to 0]
Namibia	Male	3.3 [2.52-4.32]	7.14 [5.43-9.37]	116.36	82.01 [62.18-108.33]	82.01 [62.18-108.33]	0 [0 to 0]
Nepal	Male	17.08 [12.4-23.39]	33.13 [24.28-45.43]	93.97	28.81 [21.3-39.3]	28.81 [21.3-39.3]	0 [0 to 0]
Netherlands	Male	65.49 [53.68-80.87]	99.39 [73.65-136.14]	51.76	76.25 [62.73-94.56]	79.29 [59.18-106.39]	-0.05 [-0.21 to 0.1]
New Zealand	Male	15.78 [11.95-20.74]	26.63 [20.01-35.92]	68.76	89.84 [68.17-119.42]	89.84 [68.17-119.42]	0 [0 to 0]
Nicaragua	Male	3.14 [2.34-4.24]	8.64 [6.46-11.7]	175.16	34.92 [26.18-47.08]	34.92 [26.18-47.08]	0 [0 to 0]
Niger	Male	13.19 [9.95-17.51]	32.26 [24.52-42.93]	144.58	63.23 [48.09-83.63]	63.23 [48.09-83.63]	0 [0 to 0]
Nigeria	Male	210.59 [156.71-281.54]	398.32 [296.63-529.18]	89.14	68.95 [51.83-90.95]	68.95 [51.83-90.95]	0 [0 to 0]
North Macedonia	Male	4.83 [3.58-6.56]	7.35 [5.43-10]	52.17	48.07 [35.92-65.22]	48.07 [35.92-65.22]	0 [0 to 0]
Northern Mariana Islands	Male	0.02 [0.01-0.03]	0.04 [0.02-0.05]	100	11.63 [8.56-15.76]	11.63 [8.56-15.76]	0 [0 to 0]
Norway	Male	31.7 [24.58-41.52]	48.52 [36.5-65.58]	53.06	129.89 [99.06-171.86]	130.19 [99.33-172.28]	0.14 [0.07 to 0.21]
Oman	Male	2.04 [1.45-2.82]	7.36 [5.14-10.25]	260.78	29 [21.46-39.78]	29 [21.46-39.78]	0 [0 to 0]
Pakistan	Male	110.13 [82.11-148.84]	229.64 [169.01-314.87]	108.52	32.45 [24.35-42.85]	32.45 [24.35-42.85]	0 [0 to 0]
Palestine	Male	1.3 [0.95-1.78]	4.81 [3.47-6.67]	270	29 [21.46-39.78]	29 [21.46-39.78]	0 [0 to 0]
Panama	Male	2.98 [2.24-4.03]	7.35 [5.5-9.9]	146.64	34.92 [26.18-47.08]	34.92 [26.18-47.08]	0 [0 to 0]
Papua New Guinea	Male	1.32 [0.93-1.84]	3.61 [2.52-5.09]	173.48	11.63 [8.56-15.76]	11.63 [8.56-15.76]	0 [0 to 0]
Paraguay	Male	3.36 [2.48-4.58]	8.17 [6.07-11.14]	143.15	26.98 [20.13-36.38]	26.98 [20.13-36.38]	0 [0 to 0]
Peru	Male	19.16 [14.17-26.12]	45.91 [34.23-62.68]	139.61	28.07 [20.95-38.16]	28.07 [20.95-38.16]	0.18 [0.07 to 0.29]
Philippines	Male	8.45 [5.52-12.25]	20.75 [13.54-29.75]	145.56	5.09 [3.46-7]	5.09 [3.46-7]	0 [0 to 0]
Poland	Male	103.16 [78.7-136.46]	142.1 [107.31-188.2]	37.75	53.01 [40.53-70.2]	53.01 [40.53-70.2]	0 [0 to 0]
Portugal	Male	44.19 [33.05-60.36]	60.11 [45.17-81.3]	36.03	76.86 [58-104.14]	76.86 [58-104.14]	-0.3 [-0.39 to -0.2]
Puerto Rico	Male	4.46 [3.34-6.05]	6.55 [4.83-8.86]	46.86	26.98 [20.13-36.38]	26.98 [20.13-36.38]	0 [0 to 0]
Qatar	Male	0.72 [0.5-1.01]	6.18 [4.32-8.61]	758.33	29 [21.46-39.78]	29 [21.46-39.78]	0 [0 to 0]

Republic of Korea	Male	104.2 [76.34-140.48]	261.08 [190.86-360.09]	150.56	59.5 [44.51-80.18]	59.5 [44.51-80.18]	0.05 [-0.25 to 0.34]
Republic of Moldova	Male	15.7 [11.86-20.95]	18.18 [13.74-24.19]	15.8	76.3 [57.89-101.15]	76.3 [57.89-101.15]	0 [0 to 0]
Romania	Male	63.08 [46.85-86.11]	67.36 [50.22-90.97]	6.79	48.07 [35.92-65.22]	48.07 [35.92-65.22]	0 [0 to 0]
Russian Federation	Male	1827.04 [1382.08-2362.17]	1963.02 [1492.41-2527.72]	7.44	235.26 [177.86-302.44]	218.93 [167.26-281.21]	-1.56 [-2.05 to -1.06]
Rwanda	Male	10.58 [8.09-14.1]	24.24 [18.31-32.08]	129.11	63.23 [48.09-83.63]	63.23 [48.09-83.63]	0 [0 to 0]
Saint Lucia	Male	0.11 [0.08-0.15]	0.29 [0.22-0.4]	163.64	26.98 [20.13-36.38]	26.98 [20.13-36.38]	0 [0 to 0]
Saint Vincent and the Grenadines	Male	0.09 [0.06-0.12]	0.19 [0.14-0.26]	111.11	26.98 [20.13-36.38]	26.98 [20.13-36.38]	0 [0 to 0]
Samoa	Male	0.05 [0.04-0.07]	0.09 [0.07-0.13]	80	11.63 [8.56-15.76]	11.63 [8.56-15.76]	0 [0 to 0]
Sao Tome and Principe	Male	0.2 [0.15-0.27]	0.48 [0.36-0.63]	140	63.23 [48.09-83.63]	63.23 [48.09-83.63]	0 [0 to 0]
Saudi Arabia	Male	54.21 [39.68-73.07]	221.75 [160.33-300.54]	309.06	86.73 [65.24-114.89]	86.73 [65.24-114.89]	-0.71 [-0.99 to -0.43]
Senegal	Male	12.39 [9.49-16.48]	29.16 [22.21-38.76]	135.35	63.23 [48.09-83.63]	63.23 [48.09-83.63]	0 [0 to 0]
Serbia	Male	27.07 [19.98-36.91]	28.87 [21.45-39.03]	6.65	48.07 [35.92-65.22]	48.07 [35.92-65.22]	0 [0 to 0]
Seychelles	Male	0.01 [0.01-0.01]	0.02 [0.01-0.03]	100	3.82 [2.6-5.38]	3.82 [2.6-5.38]	0 [0 to 0]
Sierra Leone	Male	7.07 [5.42-9.38]	15.88 [12.02-21.05]	124.61	63.23 [48.09-83.63]	63.23 [48.09-83.63]	0 [0 to 0]
Singapore	Male	6.85 [5.15-9.18]	23.11 [17.4-31.41]	237.37	52.77 [40.02-71.34]	52.77 [40.02-71.34]	0 [0 to 0]
Slovakia	Male	12.67 [9.51-16.92]	18.39 [13.66-24.68]	45.15	48.07 [35.92-65.22]	48.07 [35.92-65.22]	0 [0 to 0]
Slovenia	Male	5.3 [3.91-7.22]	7.71 [5.72-10.45]	45.47	48.07 [35.92-65.22]	48.07 [35.92-65.22]	0 [0 to 0]
Solomon Islands	Male	0.1 [0.07-0.14]	0.22 [0.15-0.31]	120	11.63 [8.56-15.76]	11.63 [8.56-15.76]	0 [0 to 0]
Somalia	Male	12.8 [9.54-17.1]	32.55 [24.09-43.77]	154.3	63.23 [48.09-83.63]	63.23 [48.09-83.63]	0 [0 to 0]
South Africa	Male	104.7 [78.86-137.44]	222.41 [167.53-292.61]	112.43	87.91 [66.74-115.32]	87.91 [66.74-115.32]	0 [0 to 0]
South Sudan	Male	10.69 [8.15-14.18]	17.93 [13.4-23.84]	67.73	63.23 [48.09-83.63]	63.23 [48.09-83.63]	0 [0 to 0]
Spain	Male	181.71 [135.63-244.61]	362.14 [233.18-547.14]	99.3	82.02 [61.73-110.03]	103.08 [67.06-154.41]	1.74 [1.34 to 2.15]
Sri Lanka	Male	2.3 [1.52-3.28]	4.36 [2.94-6.33]	89.57	3.82 [2.6-5.38]	3.82 [2.6-5.38]	0 [0 to 0]
Sudan	Male	16.41 [12.09-22.43]	37.49 [27.23-51.42]	128.46	29 [21.46-39.78]	29 [21.46-39.78]	0 [0 to 0]
Suriname	Male	0.38 [0.28-0.51]	0.84 [0.61-1.15]	121.05	26.98 [20.13-36.38]	26.98 [20.13-36.38]	0 [0 to 0]
Sweden	Male	36.38 [27.37-47.79]	47.5 [35.64-62.97]	30.57	65.99 [49.83-87.88]	65.3 [49.37-86.88]	-0.28 [-0.74 to 0.19]

Switzerland	Male	46.02 [34.63-61.28]	71.45 [53.25-96.53]	55.26	110.27 [82.68-146.57]	110.27 [82.68-146.57]	0 [0 to 0]
Syrian Arab Republic	Male	9.63 [7.08-13.16]	21.56 [15.56-30.01]	123.88	29 [21.46-39.78]	29 [21.46-39.78]	0 [0 to 0]
Tajikistan	Male	9.67 [7.29-12.99]	23.49 [17.51-31.42]	142.92	65.64 [49.82-87.15]	65.64 [49.82-87.15]	0 [0 to 0]
Thailand	Male	7.26 [4.74-10.41]	17.97 [11.9-26.11]	147.52	3.81 [2.56-5.37]	3.82 [2.6-5.38]	0.01 [0.01 to 0.01]
Timor-Leste	Male	0.07 [0.05-0.11]	0.16 [0.11-0.23]	128.57	3.82 [2.6-5.38]	3.82 [2.6-5.38]	0 [0 to 0]
Togo	Male	5.19 [3.93-6.88]	16.07 [11.95-21.46]	209.63	63.23 [48.09-83.63]	63.23 [48.09-83.63]	0 [0 to 0]
Tonga	Male	0.03 [0.02-0.05]	0.05 [0.03-0.06]	66.67	11.63 [8.56-15.76]	11.63 [8.56-15.76]	0 [0 to 0]
Trinidad and Tobago	Male	1.2 [0.89-1.63]	2.41 [1.79-3.31]	100.83	26.98 [20.13-36.38]	26.98 [20.13-36.38]	0 [0 to 0]
Tunisia	Male	8.27 [6.04-11.44]	19.36 [14.21-26.68]	134.1	29 [21.46-39.78]	29 [21.46-39.78]	0 [0 to 0]
Turkey	Male	15.42 [10.38-22.31]	35.11 [23.98-49.78]	127.69	7.7 [5.36-10.89]	7.7 [5.36-10.89]	-0.21 [-0.51 to 0.08]
Turkmenistan	Male	7.69 [6.09-9.83]	17.57 [13.08-23.65]	128.48	74.18 [58.89-93.83]	73.79 [56.04-97.91]	-0.02 [-0.03 to -0.01]
Uganda	Male	25.66 [19.58-34.12]	60.35 [45.36-79.86]	135.19	63.23 [48.09-83.63]	63.23 [48.09-83.63]	0 [0 to 0]
Ukraine	Male	165.15 [123-222.04]	165.77 [124.3-220.78]	0.38	58.69 [44.68-77.03]	58.97 [44.89-77.36]	0.26 [0.1 to 0.41]
United Arab Emirates	Male	2.82 [1.91-3.95]	29.73 [20.23-41.74]	954.26	29 [21.46-39.78]	29 [21.46-39.78]	0 [0 to 0]
United Kingdom	Male	391.16 [297.84-520.75]	494.24 [375.32-668.46]	26.35	114.75 [86.57-152.14]	108.23 [81.63-143.66]	-0.43 [-0.61 to -0.24]
United Republic of Tanzania	Male	41.26 [31.44-54.8]	103.66 [78.34-137.49]	151.24	63.23 [48.09-83.63]	63.23 [48.09-83.63]	0 [0 to 0]
United States of America	Male	2608.22 [1999.54-3358.03]	4431.89 [3360.93-5905.6]	69.92	200.8 [152.23-261.02]	204.08 [157.47-267.25]	0.24 [-0.26 to 0.74]
United States Virgin Islands	Male	0.13 [0.1-0.18]	0.19 [0.14-0.26]	46.15	26.98 [20.13-36.38]	26.98 [20.13-36.38]	0 [0 to 0]
Uruguay	Male	13.47 [10.13-18.37]	16.56 [12.5-22.55]	22.94	83.05 [62.52-112.17]	83.05 [62.52-112.17]	0 [0 to 0]
Uzbekistan	Male	38.8 [29.12-52.14]	94.11 [69.88-125.79]	142.55	65.64 [49.82-87.15]	65.64 [49.82-87.15]	0 [0 to 0]
Vanuatu	Male	0.05 [0.03-0.07]	0.12 [0.08-0.16]	140	11.63 [8.56-15.76]	11.63 [8.56-15.76]	0 [0 to 0]
Venezuela	Male	6.98 [4.99-9.7]	19.02 [13.46-26.39]	172.49	12.88 [9.36-17.9]	12.88 [9.36-17.9]	-0.07 [-0.11 to -0.03]
VietNam	Male	6.77 [4.43-9.65]	17.57 [11.49-25.37]	159.53	3.82 [2.6-5.38]	3.82 [2.6-5.38]	0 [0 to 0]
Yemen	Male	9.48 [6.9-12.9]	26.89 [19.49-36.64]	183.65	29 [21.46-39.78]	29 [21.46-39.78]	0 [0 to 0]
Zambia	Male	12.13 [9.24-16.13]	33.12 [24.7-44.39]	173.04	63.23 [48.09-83.63]	63.23 [48.09-83.63]	0 [0 to 0]
Zimbabwe	Male	21.76 [16.58-28.66]	38.69 [29.16-51.09]	77.8	82.01 [62.18-108.33]	82.01 [62.18-108.33]	0 [0 to 0]

Afghanistan	Female	26.72 [19.66-35.75]	64.83 [46.59-88.07]	142.63	66.8 [49.71-88.07]	48.05 [36.06-64.16]	0 [0 to 0]
Albania	Female	6.69 [5.07-9.09]	11.31 [8.5-15.57]	69.06	58.15 [44.01-78.74]	53.33 [40.1-72.31]	0 [0 to 0]
Algeria	Female	47.86 [35.6-63.61]	135.61 [100.56-181.21]	183.35	66.8 [49.71-88.07]	47.72 [35.83-63.72]	0 [0 to 0]
American Samoa	Female	0.03 [0.02-0.04]	0.06 [0.04-0.08]	100	21.08 [15.62-28.72]	16.46 [12.19-22.42]	0 [0 to 0]
Andorra	Female	0.45 [0.34-0.59]	1.07 [0.78-1.43]	137.78	161.21 [119.81-213.68]	217.34 [162.73-285.01]	0 [0 to 0]
Angola	Female	27.64 [20.89-36.48]	89.38 [67.21-118.13]	223.37	100.24 [76.12-130.42]	83.05 [63.42-108.91]	0 [0 to 0]
Antigua and Barbuda	Female	0.14 [0.11-0.18]	0.32 [0.24-0.42]	128.57	54.39 [40.98-71.57]	41.37 [31.32-54.93]	0 [0 to 0]
Argentina	Female	382.67 [287.53-502.69]	592.09 [446.12-776.63]	54.73	232.56 [174.38-306.32]	160.15 [121-211.36]	0 [0 to 0]
Armenia	Female	21.66 [15.88-29.32]	28.05 [20.74-37.73]	29.5	134.75 [100.77-179.14]	103.01 [77.78-136.86]	0 [0 to 0]
Australia	Female	216.19 [165.34-282.51]	406.09 [304.23-546.38]	87.84	240.35 [180.87-316.28]	170.1 [128.92-227.38]	-0.26 [-0.45 to -0.08]
Austria	Female	80.19 [60.7-106.56]	108.29 [80.75-145.26]	35.04	161.21 [119.81-213.68]	202.65 [153.17-266.04]	0 [0 to 0]
Azerbaijan	Female	49.25 [36.21-66.53]	104.41 [75.47-139.88]	112	163.86 [121.76-215.02]	113.46 [84.95-149.21]	0.31 [0.12 to 0.51]
Bahamas	Female	0.52 [0.4-0.7]	1.29 [0.96-1.71]	148.08	54.39 [40.98-71.57]	41.52 [31.44-55.06]	0 [0 to 0]
Bahrain	Female	0.78 [0.58-1.03]	4.32 [3.14-5.87]	453.85	66.8 [49.71-88.07]	41.9 [31.37-56.41]	0 [0 to 0]
Bangladesh	Female	144.08 [108.01-191.8]	404.23 [303.21-539.83]	180.56	56.21 [42.33-74.43]	42.48 [32.08-56.75]	0 [0 to 0]
Barbados	Female	0.72 [0.54-0.93]	1.26 [0.95-1.67]	75	54.39 [40.98-71.57]	41.54 [31.46-55.1]	0 [0 to 0]
Belarus	Female	96.05 [72.5-128.62]	106.51 [79.98-142.41]	10.89	140.74 [105.93-185.35]	111.39 [84.49-145.22]	0 [0 to 0]
Belgium	Female	102.35 [76.62-136.28]	131.07 [97.83-175.4]	28.06	161.21 [119.81-213.68]	173.3 [129.9-227.7]	0 [0 to 0]
Belize	Female	0.26 [0.2-0.34]	0.92 [0.68-1.24]	253.85	54.39 [40.98-71.57]	40.72 [30.88-54.16]	0 [0 to 0]
Benin	Female	12.7 [9.72-16.67]	35.28 [26.7-46.51]	177.8	100.24 [76.12-130.42]	82.46 [62.91-108.15]	0 [0 to 0]
Bermuda	Female	0.19 [0.14-0.25]	0.3 [0.23-0.4]	57.89	54.39 [40.98-71.57]	41.1 [31.12-54.54]	0 [0 to 0]
Bhutan	Female	0.85 [0.64-1.13]	1.73 [1.31-2.29]	103.53	56.21 [42.33-74.43]	41.91 [31.71-55.76]	0 [0 to 0]
Bolivia (Plurinational State of)	Female	16.99 [12.8-22.51]	43.05 [32.63-57.45]	153.38	84.46 [64.22-113.23]	58.9 [44.8-78.84]	0 [0 to 0]
Bosnia and Herzegovina	Female	14.43 [10.79-19.76]	15.77 [11.77-21.73]	9.29	58.15 [44.01-78.74]	53.44 [40.28-72.49]	0 [0 to 0]
Botswana	Female	4.29 [3.28-5.49]	11.69 [8.83-15.02]	172.49	110.4 [84.37-142]	97.27 [74.55-126.02]	0 [0 to 0]
Brazil	Female	324.68 [245.65-429.29]	796.99 [603.75-1052.64]	145.47	60.56 [46.06-79.76]	46.51 [35.53-61.55]	0 [0 to 0]

Brunei Darussalam	Female	2.13 [1.6-2.8]	6.79 [5.07-9.14]	218.78	283.43 [213.8-376.63]	164.21 [124.75-217.78]	0 [0 to 0]
Bulgaria	Female	35.31 [26.43-48.38]	34.26 [25.55-46.48]	-2.97	58.15 [44.01-78.74]	53.46 [40.33-72.5]	0 [0 to 0]
Burkina Faso	Female	28.27 [21.5-37.2]	68.3 [51.65-89.93]	141.6	100.24 [76.12-130.42]	83.27 [63.53-109.17]	0 [0 to 0]
Burundi	Female	15.33 [11.73-20.12]	31.09 [23.61-40.98]	102.8	100.24 [76.12-130.42]	80.73 [61.58-105.85]	0 [0 to 0]
Cabo Verde	Female	1.14 [0.88-1.5]	2.54 [1.94-3.31]	122.81	100.24 [76.12-130.42]	82.23 [62.86-107.78]	0 [0 to 0]
Cambodia	Female	2.96 [2.03-4.19]	7.53 [5.26-10.62]	154.39	10.37 [7.49-14.19]	7.54 [5.54-10.13]	0 [0 to 0]
Cameroon	Female	29.51 [22.37-38.89]	85.7 [64.95-112.66]	190.41	100.24 [76.12-130.42]	81.94 [62.55-107.43]	0 [0 to 0]
Canada	Female	431.81 [326.24-569]	719.47 [530.54-963.81]	66.62	288.53 [214.35-383.77]	226.8 [169.36-300.5]	0 [0 to 0]
Central African Republic	Female	8.27 [6.22-10.91]	16.6 [12.47-21.99]	100.73	100.24 [76.12-130.42]	82.42 [62.9-108.09]	0 [0 to 0]
Chad	Female	17.12 [13.06-22.52]	37.98 [28.61-49.97]	121.85	100.24 [76.12-130.42]	81.03 [61.81-106.28]	0 [0 to 0]
Chile	Female	136.34 [103.1-178.94]	273.36 [203.07-362.89]	100.5	232.56 [174.38-306.32]	160.2 [121.03-211.62]	0 [0 to 0]
China	Female	2168.87 [1620.84-2903.28]	5391.36 [4015.1-7302.04]	148.58	46.08 [34.87-61.5]	52.71 [39.69-70.52]	0.49 [0.29 to 0.69]
Colombia	Female	61.32 [46.03-82.12]	161.54 [120.61-217.16]	163.44	57.04 [42.87-76.75]	46.74 [35.34-62.46]	0 [0 to 0]
Comoros	Female	1.32 [1.01-1.74]	3 [2.27-3.93]	127.27	100.24 [76.12-130.42]	82.27 [62.85-107.89]	0 [0 to 0]
Congo	Female	7.06 [5.36-9.23]	19.54 [14.7-25.8]	176.77	100.24 [76.12-130.42]	81.58 [62.2-107.04]	0 [0 to 0]
Costa Rica	Female	5.65 [4.28-7.52]	15.99 [11.97-21.52]	183.01	57.04 [42.87-76.75]	46.66 [35.29-62.36]	0 [0 to 0]
Cote d'Ivoire	Female	28.4 [21.4-37.55]	75.78 [57.47-99.64]	166.83	100.24 [76.12-130.42]	80.38 [61.33-105.43]	0 [0 to 0]
Croatia	Female	20.98 [15.39-27.75]	20.57 [15.4-28.2]	-1.95	61.57 [46.38-80.89]	53.45 [40.28-72.49]	-0.22 [-0.28 to -0.16]
Cuba	Female	28.15 [21.24-37.26]	48.75 [36.62-65.19]	73.18	54.39 [40.98-71.57]	41.04 [31.04-54.5]	0 [0 to 0]
Cyprus	Female	6.58 [4.96-8.73]	14.57 [10.9-19.36]	121.43	161.21 [119.81-213.68]	186.33 [139.18-244.68]	0 [0 to 0]
Czechia	Female	39.88 [30.15-53.54]	49.59 [37.37-66.44]	24.35	58.15 [44.01-78.74]	53.35 [40.21-72.32]	0 [0 to 0]
Democratic People's Republic of Korea	Female	49.44 [36.29-66.84]	80.97 [60.26-110.53]	63.77	46.21 [34.65-62.26]	46.51 [34.93-62.42]	0 [0 to 0]
Democratic Republic of the Congo	Female	110.79 [83.81-146.02]	257.46 [194.44-340.06]	132.39	100.24 [76.12-130.42]	82.31 [62.86-107.93]	0 [0 to 0]
Denmark	Female	75.15 [58.02-95.42]	85.04 [63.13-114.8]	13.16	235.43 [181.27-302.41]	184.8 [138.19-244.75]	-0.28 [-0.35 to -0.21]
Djibouti	Female	1.1 [0.83-1.45]	4.46 [3.34-5.89]	305.45	100.24 [76.12-130.42]	80.15 [61.16-105.16]	0 [0 to 0]
Dominica	Female	0.18 [0.13-0.23]	0.22 [0.17-0.3]	22.22	54.39 [40.98-71.57]	40.29 [30.49-53.74]	0 [0 to 0]

Dominican Republic	Female	11.96 [9.04-15.94]	27.78 [20.92-36.88]	132.27	54.39 [40.98-71.57]	40.8 [30.88-54.25]	0 [0 to 0]
Ecuador	Female	39.46 [29.51-51.79]	119 [95.9-143.53]	201.57	123.24 [92.61-163.64]	92.4 [74.22-112.66]	0.66 [0.52 to 0.79]
Egypt	Female	121.51 [90.05-163.14]	258.47 [190.83-348.02]	112.72	66.8 [49.71-88.07]	46.83 [35.09-62.59]	0 [0 to 0]
El Salvador	Female	9.77 [7.35-13.12]	19.23 [14.49-25.86]	96.83	57.04 [42.87-76.75]	47.68 [35.95-63.73]	0 [0 to 0]
Equatorial Guinea	Female	1.37 [1.04-1.81]	3.91 [2.96-5.15]	185.4	100.24 [76.12-130.42]	83.91 [64.02-109.95]	0 [0 to 0]
Eritrea	Female	8.19 [6.15-10.85]	21.2 [15.99-28.02]	158.85	100.24 [76.12-130.42]	82.86 [63.34-108.56]	0 [0 to 0]
Estonia	Female	15.03 [11.31-20.03]	13.92 [10.6-18.55]	-7.39	140.74 [105.93-185.35]	110.1 [83.49-143.48]	0 [0 to 0]
Eswatini	Female	2.44 [1.84-3.15]	4.64 [3.55-5.95]	90.16	110.4 [84.37-142]	97.77 [75.02-126.67]	0 [0 to 0]
Ethiopia	Female	138.92 [104.57-182.8]	302.36 [227.71-397.91]	117.65	108.46 [82.87-142.7]	88.37 [67.18-115.98]	0 [0 to 0]
Fiji	Female	0.49 [0.35-0.68]	0.91 [0.66-1.26]	85.71	21.08 [15.62-28.72]	16.46 [12.21-22.34]	0 [0 to 0]
Finland	Female	51.08 [39.02-67.83]	61.07 [46.43-82.13]	19.56	157.31 [118.32-208.56]	99.83 [75.8-132.45]	0.01 [-0.01 to 0.02]
France	Female	556.39 [417.9-735.79]	756.9 [568.11-1010.04]	36.04	161.21 [119.81-213.68]	127.29 [96-169.01]	0 [0 to 0]
Gabon	Female	3.15 [2.39-4.16]	7.13 [5.37-9.42]	126.35	100.24 [76.12-130.42]	82.29 [62.78-107.87]	0 [0 to 0]
Gambia	Female	2.22 [1.68-2.9]	6.4 [4.85-8.41]	188.29	100.24 [76.12-130.42]	81.76 [62.33-107.23]	0 [0 to 0]
Germany	Female	709.36 [535.78-945.77]	833.95 [637.46-1116.31]	17.56	127.13 [95.32-169.31]	87.24 [67.22-115.95]	-0.01 [-0.01 to -0.01]
Ghana	Female	42.76 [32.47-56.27]	118.86 [89.92-156.46]	177.97	100.24 [76.12-130.42]	83.29 [63.59-109.2]	0 [0 to 0]
Greece	Female	54.4 [40.82-73.27]	66.13 [50.07-88.35]	21.56	77.98 [58.98-103.51]	56.92 [43.37-76.2]	0.68 [0.4 to 0.96]
Greenland	Female	0.64 [0.48-0.85]	1.02 [0.73-1.4]	59.38	288.53 [214.35-383.77]	220.88 [165.16-292.93]	0 [0 to 0]
Grenada	Female	0.18 [0.14-0.24]	0.32 [0.24-0.43]	77.78	54.39 [40.98-71.57]	40.58 [30.67-54.04]	0 [0 to 0]
Guam	Female	0.1 [0.07-0.13]	0.2 [0.15-0.27]	100	21.08 [15.62-28.72]	16.31 [12.09-22.07]	0 [0 to 0]
Guatemala	Female	12.89 [9.61-17.35]	38.93 [29.26-52.06]	202.02	57.04 [42.87-76.75]	47.18 [35.62-63.06]	0 [0 to 0]
Guinea	Female	19.37 [14.79-25.36]	36.29 [27.56-47.72]	87.35	100.24 [76.12-130.42]	82.2 [62.69-107.79]	0 [0 to 0]
Guinea-Bissau	Female	2.79 [2.12-3.67]	5.61 [4.25-7.38]	101.08	100.24 [76.12-130.42]	83 [63.36-108.82]	0 [0 to 0]
Guyana	Female	1.26 [0.95-1.69]	2.04 [1.52-2.71]	61.9	54.39 [40.98-71.57]	41.16 [31.18-54.66]	0 [0 to 0]
Haiti	Female	10.73 [8.06-14.32]	25.55 [19.14-34.31]	138.12	54.39 [40.98-71.57]	41.5 [31.48-55.05]	0 [0 to 0]
Honduras	Female	7.04 [5.29-9.44]	21.23 [15.9-28.47]	201.56	57.04 [42.87-76.75]	46.62 [35.26-62.33]	0 [0 to 0]

Hungary	Female	42.9 [32.23-58.69]	47.89 [35.94-64.5]	11.63	58.15 [44.01-78.74]	53.61 [40.46-72.75]	0 [0 to 0]
Iceland	Female	3.6 [2.88-4.58]	6.62 [5.01-8.53]	83.89	294.75 [234.01-381.69]	280.51 [209.43-363.33]	0.28 [-0.63 to 1.19]
India	Female	1669.77 [1252.71-2234.69]	3941.38 [3001.23-5275.05]	136.04	62.63 [47.77-83.63]	47.6 [36.33-63.56]	0 [0 to 0]
Indonesia	Female	71.24 [51.9-97.75]	155 [112.8-211.68]	117.57	12.59 [9.4-16.66]	9 [6.8-11.92]	0 [0 to 0]
Iran (Islamic Republic of)	Female	174.02 [130.84-231.05]	507.48 [383.03-673.14]	191.62	111.88 [83.78-149.33]	75.64 [57.34-100.02]	0.03 [-0.13 to 0.2]
Iraq	Female	30.74 [22.99-41.05]	101.73 [75.27-136.97]	230.94	66.8 [49.71-88.07]	47.69 [35.83-63.62]	0 [0 to 0]
Ireland	Female	28.56 [21.72-37.86]	53.03 [39.58-70.69]	85.68	161.21 [119.81-213.68]	136.11 [102.52-180.41]	0 [0 to 0]
Israel	Female	48.18 [36.71-61.81]	105.51 [79.3-136.79]	118.99	213.47 [159.6-278.69]	187.87 [140.06-245.73]	-0.44 [-0.64 to -0.23]
Italy	Female	566.57 [433.14-753.13]	752.54 [574.98-1013.15]	32.82	144.74 [109.48-190.78]	118.29 [89.84-158.04]	0.3 [0.08 to 0.52]
Jamaica	Female	4.6 [3.51-6.01]	8.32 [6.26-10.96]	80.87	54.39 [40.98-71.57]	40.89 [31-54.36]	0 [0 to 0]
Japan	Female	1692.66 [1270.89-2237.41]	2252.71 [1734.73-2919.88]	33.09	193.86 [146.38-255.45]	144.81 [109.46-188.98]	2 [1.55 to 2.44]
Jordan	Female	5.92 [4.36-8]	28.68 [21-38.71]	384.46	66.8 [49.71-88.07]	46.34 [34.77-61.94]	0 [0 to 0]
Kazakhstan	Female	103.41 [77.17-138.48]	144.73 [107.49-192.82]	39.96	134.75 [100.77-179.14]	102.79 [77.65-136.47]	0 [0 to 0]
Kenya	Female	57.1 [43.23-74.8]	169.67 [127.73-222.87]	197.15	108.46 [82.87-142.7]	89.12 [67.9-117.09]	0 [0 to 0]
Kuwait	Female	2.61 [1.92-3.45]	14.39 [10.36-19.37]	451.34	66.8 [49.71-88.07]	45.02 [33.83-60.31]	0 [0 to 0]
Kyrgyzstan	Female	22.62 [16.91-30.24]	41.16 [30.33-55.03]	81.96	134.75 [100.77-179.14]	102.32 [77.32-135.89]	0 [0 to 0]
Lao People's Democratic Republic	Female	1.22 [0.84-1.73]	2.67 [1.85-3.79]	118.85	10.37 [7.49-14.19]	7.21 [5.29-9.67]	0 [0 to 0]
Latvia	Female	26.25 [19.74-35.09]	21.58 [16.29-28.87]	-17.79	140.74 [105.93-185.35]	110.97 [84.16-144.68]	0 [0 to 0]
Lebanon	Female	8.75 [6.52-11.6]	19.12 [14.3-25.21]	118.51	66.8 [49.71-88.07]	49.65 [37.22-66.5]	0 [0 to 0]
Lesotho	Female	6.48 [4.98-8.27]	9.37 [7.19-12.01]	44.6	110.4 [84.37-142]	97.54 [74.83-126.5]	0 [0 to 0]
Liberia	Female	5.93 [4.55-7.76]	15.24 [11.34-20.14]	157	100.24 [76.12-130.42]	80.85 [61.64-106.1]	0 [0 to 0]
Libya	Female	6.73 [5.03-8.96]	23.06 [16.88-31.13]	242.64	66.8 [49.71-88.07]	47.34 [35.51-63.15]	0 [0 to 0]
Lithuania	Female	21.07 [16.01-28.55]	20.61 [15.73-27.78]	-2.18	86.95 [66.32-116.87]	66.16 [50.46-88.6]	0.24 [0.09 to 0.39]
Luxembourg	Female	3.95 [2.95-5.26]	7.01 [5.21-9.34]	77.47	161.21 [119.81-213.68]	135.22 [101.85-179.29]	0 [0 to 0]
Madagascar	Female	32.08 [24.31-42.25]	84 [62.9-111.09]	161.85	100.24 [76.12-130.42]	82.06 [62.62-107.59]	0 [0 to 0]
Malawi	Female	25.79 [19.64-33.87]	51.43 [39.24-67.42]	99.42	100.24 [76.12-130.42]	82.62 [63.12-108.35]	0 [0 to 0]

Malaysia	Female	5.56 [3.84-7.77]	14.61 [10.21-20.49]	162.77	10.37 [7.49-14.19]	7.05 [5.16-9.5]	0 [0 to 0]
Maldives	Female	0.05 [0.03-0.07]	0.17 [0.12-0.24]	240	10.37 [7.49-14.19]	6.65 [4.84-8.95]	0 [0 to 0]
Mali	Female	25.79 [19.68-33.87]	56.08 [42.54-73.72]	117.45	100.24 [76.12-130.42]	81.39 [62.05-106.8]	0 [0 to 0]
Malta	Female	3.58 [2.68-4.72]	5 [3.8-6.66]	39.66	161.21 [119.81-213.68]	135.55 [102.07-179.83]	0 [0 to 0]
Marshall Islands	Female	0.02 [0.01-0.03]	0.05 [0.03-0.06]	150	21.08 [15.62-28.72]	16.25 [12.08-22.21]	0 [0 to 0]
Mauritania	Female	6.01 [4.62-7.86]	13.24 [10.02-17.43]	120.3	100.24 [76.12-130.42]	82.16 [62.63-107.74]	0 [0 to 0]
Mauritius	Female	0.44 [0.31-0.6]	0.95 [0.68-1.32]	115.91	10.37 [7.49-14.19]	7.29 [5.37-9.77]	0 [0 to 0]
Mexico	Female	346.5 [259.18-463.91]	1041.49 [775.68-1389.75]	200.57	130.09 [97.28-174.43]	135.26 [101.86-178.7]	0.73 [0.58 to 0.89]
Micronesia (Federated States of)	Female	0.05 [0.04-0.07]	0.09 [0.06-0.12]	80	21.08 [15.62-28.72]	16.48 [12.2-22.43]	0 [0 to 0]
Mongolia	Female	8.11 [6.12-10.78]	24.04 [17.54-32.12]	196.42	134.75 [100.77-179.14]	102.34 [77.3-135.9]	0 [0 to 0]
Montenegro	Female	2.01 [1.51-2.76]	2.67 [2.01-3.65]	32.84	58.15 [44.01-78.74]	53.41 [40.25-72.43]	0 [0 to 0]
Morocco	Female	55.03 [41.02-73.2]	124.65 [92.48-165.91]	126.51	66.8 [49.71-88.07]	47.98 [36.04-64.04]	0 [0 to 0]
Mozambique	Female	41.69 [31.45-54.97]	81.65 [61.77-107.43]	95.85	100.24 [76.12-130.42]	83.15 [63.5-108.99]	0 [0 to 0]
Myanmar	Female	13.57 [9.44-19.09]	28.76 [20.09-40.35]	111.94	10.37 [7.49-14.19]	7.48 [5.5-10.06]	0 [0 to 0]
Namibia	Female	4.71 [3.61-6.02]	10.68 [8.11-13.74]	126.75	110.4 [84.37-142]	97.53 [74.74-126.26]	0 [0 to 0]
Nepal	Female	31.99 [23.94-42.67]	74.03 [55.7-98.3]	131.42	56.21 [42.33-74.43]	43.37 [32.86-57.76]	0 [0 to 0]
Netherlands	Female	114.5 [92.96-138.59]	147.64 [112.59-200.91]	28.94	127.99 [104.35-155.2]	97.78 [74.21-130.92]	-0.46 [-0.79 to -0.14]
New Zealand	Female	46.62 [35.45-60.71]	81.58 [61.17-108.36]	74.99	262.18 [197.02-347.8]	180.45 [136.38-237.37]	0 [0 to 0]
Nicaragua	Female	5.48 [4.13-7.32]	16.1 [12.04-21.64]	193.8	57.04 [42.87-76.75]	46.84 [35.39-62.57]	0 [0 to 0]
Niger	Female	19.28 [14.54-25.36]	54.88 [41.38-72.5]	184.65	100.24 [76.12-130.42]	82.49 [62.9-108.1]	0 [0 to 0]
Nigeria	Female	248.59 [190.96-325.51]	702.12 [526.6-928.82]	182.44	108.46 [82.87-142.7]	90.05 [68.57-118.52]	0 [0 to 0]
North Macedonia	Female	6.1 [4.55-8.34]	8.93 [6.71-12.22]	46.39	58.15 [44.01-78.74]	53.2 [40.06-72.11]	0 [0 to 0]
Northern Mariana Islands	Female	0.02 [0.02-0.03]	0.06 [0.04-0.09]	200	21.08 [15.62-28.72]	16.19 [12.04-22.07]	0 [0 to 0]
Norway	Female	62.6 [48.49-79.77]	90.5 [69.03-118.53]	44.57	254.48 [192.74-331.98]	191.23 [145.29-251.52]	0.22 [0.11 to 0.32]
Oman	Female	2.56 [1.92-3.41]	7.82 [5.78-10.35]	205.47	66.8 [49.71-88.07]	42.55 [31.98-57.01]	0 [0 to 0]
Pakistan	Female	187.53 [141.35-250.32]	422.46 [317.07-564.85]	125.28	62.63 [47.77-83.63]	47.11 [36.01-62.97]	0 [0 to 0]

Palestine	Female	3.58 [2.68-4.74]	10.8 [7.95-14.55]	201.68	66.8 [49.71-88.07]	47.73 [35.94-63.72]	0 [0 to 0]
Panama	Female	4.66 [3.51-6.23]	12.22 [9.2-16.48]	162.23	57.04 [42.87-76.75]	46.07 [34.87-61.56]	0 [0 to 0]
Papua New Guinea	Female	2.29 [1.65-3.15]	6.32 [4.51-8.72]	175.98	21.08 [15.62-28.72]	16.18 [12.04-21.94]	0 [0 to 0]
Paraguay	Female	6.85 [5.2-9.07]	16.74 [12.64-22.1]	144.38	54.39 [40.98-71.57]	40.8 [30.88-54.27]	0 [0 to 0]
Peru	Female	41.36 [30.94-55.3]	103.39 [77.05-138.35]	149.98	59.24 [44.16-78.79]	44.15 [33.22-59]	0.21 [0.08 to 0.35]
Philippines	Female	21.59 [15.77-29.76]	55.56 [40.68-75.11]	157.34	12.59 [9.4-16.66]	9.06 [6.83-11.99]	0 [0 to 0]
Poland	Female	149.8 [114.15-200.92]	202.18 [151.93-269.5]	34.97	64.49 [48.92-86.4]	59.27 [45.22-79.16]	0 [0 to 0]
Portugal	Female	131.24 [98.57-176.11]	175.36 [131.88-234.21]	33.62	207.97 [156.67-276.89]	145.57 [109.47-195.24]	0.34 [0.23 to 0.45]
Puerto Rico	Female	10.28 [7.8-13.51]	15.51 [11.64-20.49]	50.88	54.39 [40.98-71.57]	41.66 [31.56-55.22]	0 [0 to 0]
Qatar	Female	0.52 [0.37-0.69]	4.12 [2.94-5.56]	692.31	66.8 [49.71-88.07]	37.63 [28.19-50.68]	0 [0 to 0]
Republic of Korea	Female	696.38 [525.75-908.3]	1401.16 [1039.79-1854.39]	101.21	340.81 [258.39-445.56]	196.59 [149.15-256.03]	0.71 [0.44 to 0.98]
Republic of Moldova	Female	35.55 [26.76-47.32]	39.62 [29.78-52.74]	11.45	140.74 [105.93-185.35]	110.87 [84.11-144.5]	0 [0 to 0]
Romania	Female	84.5 [63.29-115.99]	91.59 [68.72-124.12]	8.39	58.15 [44.01-78.74]	53.46 [40.33-72.51]	0 [0 to 0]
Russian Federation	Female	3078.2 [2359.67-3989.97]	3346.15 [2570.61-4299.38]	8.7	330.69 [254.52-424.62]	268.42 [204.66-347.13]	-0.78 [-1 to -0.55]
Rwanda	Female	19.49 [14.84-25.45]	44.77 [34-58.89]	129.71	100.24 [76.12-130.42]	83.69 [63.93-109.68]	0 [0 to 0]
Saint Lucia	Female	0.25 [0.19-0.33]	0.62 [0.47-0.83]	148	54.39 [40.98-71.57]	40.95 [31.01-54.43]	0 [0 to 0]
Saint Vincent and the Grenadines	Female	0.19 [0.15-0.26]	0.36 [0.27-0.48]	89.47	54.39 [40.98-71.57]	40.24 [30.52-53.6]	0 [0 to 0]
Samoa	Female	0.1 [0.07-0.14]	0.17 [0.12-0.23]	70	21.08 [15.62-28.72]	16.27 [12.06-22.06]	0 [0 to 0]
Sao Tome and Principe	Female	0.35 [0.26-0.45]	0.73 [0.55-0.97]	108.57	100.24 [76.12-130.42]	81.76 [62.43-107.22]	0 [0 to 0]
Saudi Arabia	Female	48.27 [35.92-62.82]	205.25 [149.21-270.23]	325.21	138.88 [103.64-181.42]	107.25 [80.77-139.97]	-0.47 [-0.66 to -0.28]
Senegal	Female	20.3 [15.43-26.72]	49.29 [37.42-64.8]	142.81	100.24 [76.12-130.42]	82.43 [62.88-108.06]	0 [0 to 0]
Serbia	Female	35.53 [26.42-49.11]	38.41 [28.59-52.61]	8.11	58.15 [44.01-78.74]	53.35 [40.18-72.36]	0 [0 to 0]
Seychelles	Female	0.03 [0.02-0.04]	0.06 [0.04-0.09]	100	10.37 [7.49-14.19]	7.14 [5.25-9.55]	0 [0 to 0]
Sierra Leone	Female	11.08 [8.47-14.53]	23.88 [18.07-31.39]	115.52	100.24 [76.12-130.42]	81.32 [62-106.68]	0 [0 to 0]
Singapore	Female	43.34 [32.81-57.29]	112.3 [85-149.5]	159.11	282.18 [213.42-373.66]	160.46 [122.17-211.42]	0.39 [0.26 to 0.52]
Slovakia	Female	17.96 [13.58-24.21]	24.82 [18.68-33.54]	38.2	58.15 [44.01-78.74]	53.51 [40.36-72.57]	0 [0 to 0]

Slovenia	Female	7.5 [5.64-10.28]	9.95 [7.51-13.57]	32.67	58.15 [44.01-78.74]	53.28 [40.13-72.22]	0 [0 to 0]
Solomon Islands	Female	0.16 [0.11-0.22]	0.42 [0.3-0.58]	162.5	21.08 [15.62-28.72]	16.27 [12.08-22.15]	0 [0 to 0]
Somalia	Female	20.71 [15.51-27.43]	52.93 [39.64-69.87]	155.58	100.24 [76.12-130.42]	83.06 [63.51-108.85]	0 [0 to 0]
South Africa	Female	158.19 [120.8-204.7]	334.87 [256.49-437.12]	111.69	118.93 [90.73-154.88]	104.92 [80.12-137.23]	0 [0 to 0]
South Sudan	Female	13.36 [10.11-17.45]	28.67 [21.33-38.07]	114.6	100.24 [76.12-130.42]	81.47 [62.15-106.89]	0 [0 to 0]
Spain	Female	398.56 [302.54-522.16]	840.7 [508.81-1421.41]	110.93	170.99 [129.98-224.05]	168.99 [105.07-275.48]	1.88 [1.5 to 2.27]
Sri Lanka	Female	6.26 [4.33-8.83]	14.27 [10.15-19.71]	127.96	10.37 [7.49-14.19]	7.39 [5.44-9.92]	0 [0 to 0]
Sudan	Female	36.2 [27.02-48.49]	82.09 [60.47-109.46]	126.77	66.8 [49.71-88.07]	47.11 [35.41-63.03]	0 [0 to 0]
Suriname	Female	0.81 [0.61-1.07]	1.83 [1.37-2.42]	125.93	54.39 [40.98-71.57]	41.16 [31.16-54.67]	0 [0 to 0]
Sweden	Female	55.62 [42.36-73.99]	69.15 [52.39-92.86]	24.33	93.9 [71.48-125.02]	79.55 [60.5-105.9]	-0.19 [-0.57 to 0.18]
Switzerland	Female	70.89 [53.39-94.17]	103.81 [77.6-138.7]	46.44	161.21 [119.81-213.68]	135.6 [102.14-179.85]	0 [0 to 0]
Syrian Arab Republic	Female	20.91 [15.56-28]	51.44 [37.74-69.11]	146.01	66.8 [49.71-88.07]	47.85 [35.96-63.88]	0 [0 to 0]
Tajikistan	Female	20.8 [15.56-27.83]	50.77 [37.11-67.84]	144.09	134.75 [100.77-179.14]	100.93 [76.23-134.02]	0 [0 to 0]
Thailand	Female	21.45 [15.35-30.14]	56.75 [40.91-78.31]	164.57	10.08 [7.35-13.84]	7.31 [5.37-9.82]	0.14 [0.11 to 0.17]
Timor-Leste	Female	0.19 [0.13-0.27]	0.43 [0.3-0.59]	126.32	10.37 [7.49-14.19]	7.05 [5.18-9.51]	0 [0 to 0]
Togo	Female	9.43 [7.14-12.44]	28.57 [21.6-37.65]	202.97	100.24 [76.12-130.42]	83.2 [63.5-109.14]	0 [0 to 0]
Tonga	Female	0.07 [0.05-0.09]	0.09 [0.07-0.12]	28.57	21.08 [15.62-28.72]	16.58 [12.28-22.46]	0 [0 to 0]
Trinidad and Tobago	Female	2.5 [1.9-3.3]	4.95 [3.74-6.52]	98	54.39 [40.98-71.57]	40.73 [30.84-54.16]	0 [0 to 0]
Tunisia	Female	18.8 [13.99-25.01]	46.5 [34.59-61.56]	147.34	66.8 [49.71-88.07]	48.23 [36.23-64.33]	0 [0 to 0]
Turkey	Female	43.57 [31.81-59.59]	100.67 [73.88-137.58]	131.05	21.02 [15.56-28.6]	14.52 [10.83-19.63]	-0.1 [-0.25 to 0.04]
Turkmenistan	Female	8.49 [6.67-11.24]	21.53 [15.9-29.1]	153.59	73.69 [58.58-96.78]	80.24 [60.74-107.03]	0.57 [0.41 to 0.74]
Uganda	Female	41.31 [31.39-54.44]	106.55 [80.83-140.28]	157.93	100.24 [76.12-130.42]	83.14 [63.49-109]	0 [0 to 0]
Ukraine	Female	457.33 [346.67-630.74]	447.91 [340.78-609.78]	-2.06	120.74 [91.86-160.09]	93.56 [71.72-123.95]	0.25 [0.09 to 0.4]
United Arab Emirates	Female	1.87 [1.36-2.49]	18.05 [12.65-24.38]	865.24	66.8 [49.71-88.07]	37.63 [28.32-50.63]	0 [0 to 0]
United Kingdom	Female	532.47 [407.53-710.08]	745.56 [573.97-1002.32]	40.02	143.28 [108.72-191.52]	133.38 [101.72-177.72]	0.22 [-0.23 to 0.67]
United Republic of Tanzania	Female	69.37 [52.79-91.25]	174.31 [131.53-229.78]	151.28	100.24 [76.12-130.42]	82.38 [62.86-108]	0 [0 to 0]

United States of America	Female	4078.61 [3164.52-5185.12]	7745.95 [5895.22-10101.08]	89.92	293.2 [223.86-375.63]	281.4 [216.27-364.83]	0.66 [0.48 to 0.83]
United States Virgin Islands	Female	0.3 [0.22-0.4]	0.45 [0.33-0.6]	50	54.39 [40.98-71.57]	41.71 [31.58-55.3]	0 [0 to 0]
Uruguay	Female	40.11 [30.11-52.98]	50.11 [37.74-65.99]	24.93	232.56 [174.38-306.32]	161.01 [121.62-212.49]	0 [0 to 0]
Uzbekistan	Female	87.53 [65.85-117.55]	215.44 [158.04-287.8]	146.13	134.75 [100.77-179.14]	101.94 [77.04-135.39]	0 [0 to 0]
Vanuatu	Female	0.08 [0.06-0.1]	0.2 [0.15-0.27]	150	21.08 [15.62-28.72]	16.19 [12.06-22.01]	0 [0 to 0]
Venezuela (Bolivarian Republic of)	Female	15.76 [11.66-21.21]	44.01 [32.88-59.58]	179.25	27.35 [20.34-37.11]	20.4 [15.28-27.78]	-0.02 [-0.03 to -0.01]
VietNam	Female	24.16 [17.26-33.51]	57.59 [40.76-80.41]	138.37	10.37 [7.49-14.19]	7.44 [5.49-9.95]	0 [0 to 0]
Yemen	Female	20.67 [15.54-27.49]	60.9 [45.44-80.72]	194.63	66.8 [49.71-88.07]	48.06 [36.03-64.14]	0 [0 to 0]
Zambia	Female	19.3 [14.49-25.55]	49.59 [37.49-65.25]	156.94	100.24 [76.12-130.42]	81.64 [62.31-107.12]	0 [0 to 0]
Zimbabwe	Female	29.88 [22.72-38.42]	58.56 [44.51-75.27]	95.98	110.4 [84.37-142]	97.74 [74.9-126.64]	0 [0 to 0]

ASIR: age-standardized incidence; EAPCs: estimated annual percentage changes, CI: confidence interval; UI: Uncertainty interval.

Supplementary Table S4. Age distribution of incidence (per 100,000) for hand osteoarthritis in different countries in 2019.

Supplementary Table S4. Age distribution of incidence (per 100,000) for osteoarthritis-hand in different countries in 2019.

country	sex	<5	5 to 9	10 to 14	15 to 19	20 to 24	25 to 29	30 to 34	35 to 39	40 to 44	45 to 49	50 to 54	55 to 59	60 to 64	65 to 69	70 to 74	75 to 79	80 to 84	85 to 89	90 to 94	95+
Afghanistan	Male	0	0	0	0	0	0	13.23	39.99	67.53	89.71	97.52	90.43	74.27	58.1	47.85	48.18	51.44	57.42	66.95	80.68
Afghanistan	Female	0	0	0	0	0	0	19.49	68.7	143.36	223.28	259.91	238.92	185.34	132.9	98.16	86.22	87.37	100.92	122.47	147.21
Albania	Male	0	0	0	0	0	0	15.41	53.02	101.71	147.09	173.29	171.44	140.88	104.54	82.34	77.11	74.78	76.32	84.47	98.65
Albania	Female	0	0	0	0	0	0	16.31	56.34	110.27	165.54	205.19	212.5	184.66	146.16	117.36	108.03	106.04	113.45	131.59	155.62
Algeria	Male	0	0	0	0	0	0	13.23	39.99	67.53	89.71	97.52	90.43	74.27	58.1	47.85	48.18	51.44	57.42	66.95	80.68
Algeria	Female	0	0	0	0	0	0	19.49	68.7	143.36	223.28	259.91	238.92	185.34	132.9	98.16	86.22	87.37	100.92	122.47	147.21
American Samoa	Male	0	0	0	0	0	0	4.36	10.65	14.87	22.35	32.17	39.38	40.35	37.6	36.39	40.75	45.47	52.85	63.7	79.31
American Samoa	Female	0	0	0	0	0	0	7.73	22.12	35.76	50.64	63.5	68.03	63.37	56.62	54.66	62.3	70.48	83.47	104.52	131.83
Andorra	Male	0	0	0	0	0	0	122.97	358.34	589.86	919.3	1267.93	1130.56	632.57	287.42	138.44	83.62	64.98	59.61	63.32	73.99
Andorra	Female	0	0	0	0	0	0	52.68	171.66	338.66	551.11	691.52	638.6	445.89	271.49	173.11	128.06	108.63	105.53	113.91	129.92
Angola	Male	0	0	0	0	0	0	28.81	100.47	179.18	219.94	215.43	182.85	136.22	98.79	79.04	76.57	76.51	79.01	86.75	100.22
Angola	Female	0	0	0	0	0	0	37.57	140.34	267.59	343.87	351.39	311.02	238.73	176.47	138.2	122.79	119.35	126.6	141.38	162.55
Antigua and Barbuda	Male	0	0	0	0	0	0	9.64	30.84	54.33	75.84	89.95	89.88	76.32	61.7	54.81	59.52	65.62	73.19	84.05	100.02
Antigua and Barbuda	Female	0	0	0	0	0	0	15.17	54.74	110.24	163.85	192.97	189.52	159.44	124.96	104.56	102.52	105.99	119.89	141.44	165.86
Argentina	Male	0	0	0	0	0	0	28.21	89.98	163.3	241.06	310.76	332.12	274.99	189.13	125.51	95.53	82	77.84	82.59	93.46
Argentina	Female	0	0	0	0	0	0	67.37	270.3	610.21	940.34	1040.86	828.82	486.5	253.62	148.16	110.62	99.69	104.1	117.8	136.04
Armenia	Male	0	0	0	0	0	0	22.44	79.13	150.54	206.35	231.16	224.19	188.63	144.76	108.49	90.96	81.68	79.72	86.4	100.81
Armenia	Female	0	0	0	0	0	0	33.29	135.15	304.33	468.78	541.1	504.41	383.85	254.29	167.89	127.79	114.46	119.59	136.6	159.85
Australia	Male	0	0	0	0	0	0	28.21	89.98	163.3	241.06	310.76	332.12	274.99	189.13	125.51	95.53	82	77.84	82.59	93.46
Australia	Female	0	0	0	0	0	0	64.77	271.28	655.49	1060.91	1188.98	920.98	512.04	253.67	145.18	107.42	96.24	100.48	112.89	130.19
Austria	Male	0	0	0	0	0	0	110.01	319.3	527.48	810.36	1119.99	1031.47	606.59	289.78	143.73	88.61	68.43	61.8	64.74	74.17
Austria	Female	0	0	0	0	0	0	52.68	171.66	338.66	551.11	691.52	638.6	445.89	271.49	173.11	128.06	108.63	105.53	113.91	129.92
Azerbaijan	Male	0	0	0	0	0	0	19.93	69.26	129.63	177.74	201.92	199.27	169.93	132.8	102.62	87.66	78.78	77.62	86.32	103.09
Azerbaijan	Female	0	0	0	0	0	0	40.23	166.78	384.2	595.1	682.08	616.4	441.36	274.88	173.56	127.56	112.19	116.21	131.79	152.57
Bahamas	Male	0	0	0	0	0	0	9.64	30.84	54.33	75.84	89.95	89.88	76.32	61.7	54.81	59.52	65.62	73.19	84.05	100.02
Bahamas	Female	0	0	0	0	0	0	15.17	54.74	110.24	163.85	192.97	189.52	159.44	124.96	104.56	102.52	105.99	119.89	141.44	165.86
Bahrain	Male	0	0	0	0	0	0	13.23	39.99	67.53	89.71	97.52	90.43	74.27	58.1	47.85	48.18	51.44	57.42	66.95	80.68

Bahrain	Female	0	0	0	0	0	0	19.49	68.7	143.36	223.28	259.91	238.92	185.34	132.9	98.16	86.22	87.37	100.92	122.47	147.21
Bangladesh	Male	0	0	0	0	0	0	10.3	32.23	56.19	78.75	94.37	96.7	84.7	69.74	62.26	66.26	70.58	75.56	84.16	99.57
Bangladesh	Female	0	0	0	0	0	0	16.31	57.17	111.43	161.42	191.83	195.03	170.6	138.37	116.81	114.17	116.75	126.39	142.86	165.84
Barbados	Male	0	0	0	0	0	0	9.64	30.84	54.33	75.84	89.95	89.88	76.32	61.7	54.81	59.52	65.62	73.19	84.05	100.02
Barbados	Female	0	0	0	0	0	0	15.17	54.74	110.24	163.85	192.97	189.52	159.44	124.96	104.56	102.52	105.99	119.89	141.44	165.86
Belarus	Male	0	0	0	0	0	0	31.46	105	190.52	254.31	275.2	252.28	197.24	140.34	99.62	82	75.4	77.3	85.72	99.94
Belarus	Female	0	0	0	0	0	0	44.02	164.39	334.35	480.97	546.03	509.01	387.03	258.05	169.54	128.51	114.26	118.43	135.14	156.66
Belgium	Male	0	0	0	0	0	0	82.53	231.88	374.83	573.57	801.51	802.69	536.04	279.81	145.94	91.46	71.43	65.8	70.45	82.36
Belgium	Female	0	0	0	0	0	0	52.68	171.66	338.66	551.11	691.52	638.6	445.89	271.49	173.11	128.06	108.63	105.53	113.91	129.92
Belize	Male	0	0	0	0	0	0	9.64	30.84	54.33	75.84	89.95	89.88	76.32	61.7	54.81	59.52	65.62	73.19	84.05	100.02
Belize	Female	0	0	0	0	0	0	15.17	54.74	110.24	163.85	192.97	189.52	159.44	124.96	104.56	102.52	105.99	119.89	141.44	165.86
Benin	Male	0	0	0	0	0	0	28.81	100.47	179.18	219.94	215.43	182.85	136.22	98.79	79.04	76.57	76.51	79.01	86.75	100.22
Benin	Female	0	0	0	0	0	0	37.57	140.34	267.59	343.87	351.39	311.02	238.73	176.47	138.2	122.79	119.35	126.6	141.38	162.55
Bermuda	Male	0	0	0	0	0	0	9.64	30.84	54.33	75.84	89.95	89.88	76.32	61.7	54.81	59.52	65.62	73.19	84.05	100.02
Bermuda	Female	0	0	0	0	0	0	15.17	54.74	110.24	163.85	192.97	189.52	159.44	124.96	104.56	102.52	105.99	119.89	141.44	165.86
Bhutan	Male	0	0	0	0	0	0	10.3	32.23	56.19	78.75	94.37	96.7	84.7	69.74	62.26	66.26	70.58	75.56	84.16	99.57
Bhutan	Female	0	0	0	0	0	0	16.31	57.17	111.43	161.42	191.83	195.03	170.6	138.37	116.81	114.17	116.75	126.39	142.86	165.84
Bolivia (Plurinational State of)	Male	0	0	0	0	0	0	10.95	36.11	67.23	96.89	113.42	109.51	90.34	71.57	61.3	62.28	64.9	71.09	84.17	103.94
Bolivia (Plurinational State of)	Female	0	0	0	0	0	0	21.87	82.82	181.28	283.45	331.74	305.64	234.26	169.6	129.27	112.91	108.99	118.83	140.65	168.52
Bosnia and Herzegovina	Male	0	0	0	0	0	0	15.41	53.02	101.71	147.09	173.29	171.44	140.88	104.54	82.34	77.11	74.78	76.32	84.47	98.65
Bosnia and Herzegovina	Female	0	0	0	0	0	0	16.31	56.34	110.27	165.54	205.19	212.5	184.66	146.16	117.36	108.03	106.04	113.45	131.59	155.62
Botswana	Male	0	0	0	0	0	0	49.38	164.05	267	295.79	263.29	205.58	142.41	97.55	76.13	73.35	74.27	77.94	85.3	97.85
Botswana	Female	0	0	0	0	0	0	55.57	195.99	335.88	383.13	355.75	299.25	226.68	167.8	131.69	118.01	114.99	123.73	139.65	161.55
Brazil	Male	0	0	0	0	0	0	10.48	33.16	58.49	80.62	94.59	96.53	86.7	76.49	74.68	85.87	95.7	107.14	123.07	143.23
Brazil	Female	0	0	0	0	0	0	15.89	58.09	118.54	171.76	197.35	198.13	178	155.41	143.57	149.73	158.75	173.77	196.3	225.64
Brunei Darussalam	Male	0	0	0	0	0	0	14.15	42.89	77.43	124.64	180.63	220.76	213.1	168.41	121.21	92.91	77.85	73.03	78.46	91.37
Brunei Darussalam	Female	0	0	0	0	0	0	75.79	329.86	785.05	1218.5	1293.48	970.21	540.33	263.33	140.06	96.18	85.35	92.21	107.9	127.52
Bulgaria	Male	0	0	0	0	0	0	15.41	53.02	101.71	147.09	173.29	171.44	140.88	104.54	82.34	77.11	74.78	76.32	84.47	98.65
Bulgaria	Female	0	0	0	0	0	0	16.31	56.34	110.27	165.54	205.19	212.5	184.66	146.16	117.36	108.03	106.04	113.45	131.59	155.62
Burkina Faso	Male	0	0	0	0	0	0	28.81	100.47	179.18	219.94	215.43	182.85	136.22	98.79	79.04	76.57	76.51	79.01	86.75	100.22

Burkina Faso	Female	0	0	0	0	0	0	37.57	140.34	267.59	343.87	351.39	311.02	238.73	176.47	138.2	122.79	119.35	126.6	141.38	162.55
Burundi	Male	0	0	0	0	0	0	28.81	100.47	179.18	219.94	215.43	182.85	136.22	98.79	79.04	76.57	76.51	79.01	86.75	100.22
Burundi	Female	0	0	0	0	0	0	37.57	140.34	267.59	343.87	351.39	311.02	238.73	176.47	138.2	122.79	119.35	126.6	141.38	162.55
Cabo Verde	Male	0	0	0	0	0	0	28.81	100.47	179.18	219.94	215.43	182.85	136.22	98.79	79.04	76.57	76.51	79.01	86.75	100.22
Cabo Verde	Female	0	0	0	0	0	0	37.57	140.34	267.59	343.87	351.39	311.02	238.73	176.47	138.2	122.79	119.35	126.6	141.38	162.55
Cambodia	Male	0	0	0	0	0	0	1.91	4.29	5.37	6.75	8.71	10.32	10.91	11.3	12.27	16.11	20.37	27.04	37.19	52.19
Cambodia	Female	0	0	0	0	0	0	4.04	10.17	13.98	20	26.9	31.14	32.38	31.57	32.36	40.1	49.06	62.43	82.34	109.8
Cameroon	Male	0	0	0	0	0	0	28.81	100.47	179.18	219.94	215.43	182.85	136.22	98.79	79.04	76.57	76.51	79.01	86.75	100.22
Cameroon	Female	0	0	0	0	0	0	37.57	140.34	267.59	343.87	351.39	311.02	238.73	176.47	138.2	122.79	119.35	126.6	141.38	162.55
Canada	Male	0	0	0	0	0	0	83.35	197.72	291.19	476.33	704.77	757.55	515.3	238.82	118.49	81.45	69.13	65.9	70.63	81.87
Canada	Female	0	0	0	0	0	0	120.45	352.35	696.56	1216.94	1447.62	1055.06	491.98	190.46	97.47	75.62	74.51	84.34	98.26	115
Central African Republic	Male	0	0	0	0	0	0	28.81	100.47	179.18	219.94	215.43	182.85	136.22	98.79	79.04	76.57	76.51	79.01	86.75	100.22
Central African Republic	Female	0	0	0	0	0	0	37.57	140.34	267.59	343.87	351.39	311.02	238.73	176.47	138.2	122.79	119.35	126.6	141.38	162.55
Chad	Male	0	0	0	0	0	0	28.81	100.47	179.18	219.94	215.43	182.85	136.22	98.79	79.04	76.57	76.51	79.01	86.75	100.22
Chad	Female	0	0	0	0	0	0	37.57	140.34	267.59	343.87	351.39	311.02	238.73	176.47	138.2	122.79	119.35	126.6	141.38	162.55
Chile	Male	0	0	0	0	0	0	28.21	89.98	163.3	241.06	310.76	332.12	274.99	189.13	125.51	95.53	82	77.84	82.59	93.46
Chile	Female	0	0	0	0	0	0	67.37	270.3	610.21	940.34	1040.86	828.82	486.5	253.62	148.16	110.62	99.69	104.1	117.8	136.04
China	Male	0	0	0	0	0	0	12.75	42.66	82.42	129.87	177.17	208.23	198.53	162.86	131.59	117.69	110.32	110.46	117.67	131.18
China	Female	0	0	0	0	0	0	16.65	58.76	112.93	154.78	168.42	161.69	139.67	119.36	112.76	121.05	130.74	145.46	164.7	189.92
Colombia	Male	0	0	0	0	0	0	10.8	37.46	73.64	108.58	126	119	96.28	74.93	63.16	63.51	65.71	71.53	83.61	102.71
Colombia	Female	0	0	0	0	0	0	13.91	51.29	111.96	178.35	215.15	206.04	167.22	129.49	104.94	98.92	100.33	112.98	137.22	165.48
Comoros	Male	0	0	0	0	0	0	28.81	100.47	179.18	219.94	215.43	182.85	136.22	98.79	79.04	76.57	76.51	79.01	86.75	100.22
Comoros	Female	0	0	0	0	0	0	37.57	140.34	267.59	343.87	351.39	311.02	238.73	176.47	138.2	122.79	119.35	126.6	141.38	162.55
Congo	Male	0	0	0	0	0	0	28.81	100.47	179.18	219.94	215.43	182.85	136.22	98.79	79.04	76.57	76.51	79.01	86.75	100.22
Congo	Female	0	0	0	0	0	0	37.57	140.34	267.59	343.87	351.39	311.02	238.73	176.47	138.2	122.79	119.35	126.6	141.38	162.55
Costa Rica	Male	0	0	0	0	0	0	10.8	37.46	73.64	108.58	126	119	96.28	74.93	63.16	63.51	65.71	71.53	83.61	102.71
Costa Rica	Female	0	0	0	0	0	0	13.91	51.29	111.96	178.35	215.15	206.04	167.22	129.49	104.94	98.92	100.33	112.98	137.22	165.48
Côte d'Ivoire	Male	0	0	0	0	0	0	28.81	100.47	179.18	219.94	215.43	182.85	136.22	98.79	79.04	76.57	76.51	79.01	86.75	100.22
Côte d'Ivoire	Female	0	0	0	0	0	0	37.57	140.34	267.59	343.87	351.39	311.02	238.73	176.47	138.2	122.79	119.35	126.6	141.38	162.55
Croatia	Male	0	0	0	0	0	0	15.41	53.02	101.71	147.09	173.29	171.44	140.88	104.54	82.34	77.11	74.78	76.32	84.47	98.65

Croatia	Female	0	0	0	0	0	0	16.31	56.34	110.27	165.54	205.19	212.5	184.66	146.16	117.36	108.03	106.04	113.45	131.59	155.62
Cuba	Male	0	0	0	0	0	0	9.64	30.84	54.33	75.84	89.95	89.88	76.32	61.7	54.81	59.52	65.62	73.19	84.05	100.02
Cuba	Female	0	0	0	0	0	0	15.17	54.74	110.24	163.85	192.97	189.52	159.44	124.96	104.56	102.52	105.99	119.89	141.44	165.86
Cyprus	Male	0	0	0	0	0	0	96.36	273.88	443.54	680.24	957.53	923.86	576.32	285.2	144.96	91.1	70.33	63.75	67.65	78.18
Cyprus	Female	0	0	0	0	0	0	52.68	171.66	338.66	551.11	691.52	638.6	445.89	271.49	173.11	128.06	108.63	105.53	113.91	129.92
Czechia	Male	0	0	0	0	0	0	15.41	53.02	101.71	147.09	173.29	171.44	140.88	104.54	82.34	77.11	74.78	76.32	84.47	98.65
Czechia	Female	0	0	0	0	0	0	16.31	56.34	110.27	165.54	205.19	212.5	184.66	146.16	117.36	108.03	106.04	113.45	131.59	155.62
Democratic People's Republic of Korea	Male	0	0	0	0	0	0	12.04	37.39	67.59	109.27	167.86	212.48	195.79	139.72	94.9	76.98	69.93	68.86	75.05	87.97
Democratic People's Republic of Korea	Female	0	0	0	0	0	0	16.47	56.38	105.66	144.64	158.52	148.37	121.15	94.77	81.04	83.01	88.08	99.19	117.35	140.3
Democratic Republic of the Congo	Male	0	0	0	0	0	0	28.81	100.47	179.18	219.94	215.43	182.85	136.22	98.79	79.04	76.57	76.51	79.01	86.75	100.22
Democratic Republic of the Congo	Female	0	0	0	0	0	0	37.57	140.34	267.59	343.87	351.39	311.02	238.73	176.47	138.2	122.79	119.35	126.6	141.38	162.55
Denmark	Male	0	0	0	0	0	0	69.65	191.48	304.64	464.28	656.52	675.61	473.65	264.17	143.54	92.59	73.88	68.26	72.37	84.69
Denmark	Female	0	0	0	0	0	0	70.72	238.01	480.7	784.26	965.43	833.45	524.75	292.4	176.52	125.82	104.93	101.5	108.43	122.37
Djibouti	Male	0	0	0	0	0	0	28.81	100.47	179.18	219.94	215.43	182.85	136.22	98.79	79.04	76.57	76.51	79.01	86.75	100.22
Djibouti	Female	0	0	0	0	0	0	37.57	140.34	267.59	343.87	351.39	311.02	238.73	176.47	138.2	122.79	119.35	126.6	141.38	162.55
Dominica	Male	0	0	0	0	0	0	9.64	30.84	54.33	75.84	89.95	89.88	76.32	61.7	54.81	59.52	65.62	73.19	84.05	100.02
Dominica	Female	0	0	0	0	0	0	15.17	54.74	110.24	163.85	192.97	189.52	159.44	124.96	104.56	102.52	105.99	119.89	141.44	165.86
Dominican Republic	Male	0	0	0	0	0	0	9.64	30.84	54.33	75.84	89.95	89.88	76.32	61.7	54.81	59.52	65.62	73.19	84.05	100.02
Dominican Republic	Female	0	0	0	0	0	0	15.17	54.74	110.24	163.85	192.97	189.52	159.44	124.96	104.56	102.52	105.99	119.89	141.44	165.86
Ecuador	Male	0	0	0	0	0	0	15.3	47.86	89.31	131.81	151.56	139.47	106.56	78.01	64.52	63	64.85	71.74	84.69	103.51
Ecuador	Female	0	0	0	0	0	0	43.74	143.52	313.36	545.17	649.55	519.13	316	192	135.33	113.22	106.59	114.48	131.92	155.99
Egypt	Male	0	0	0	0	0	0	13.23	39.99	67.53	89.71	97.52	90.43	74.27	58.1	47.85	48.18	51.44	57.42	66.95	80.68
Egypt	Female	0	0	0	0	0	0	19.49	68.7	143.36	223.28	259.91	238.92	185.34	132.9	98.16	86.22	87.37	100.92	122.47	147.21
El Salvador	Male	0	0	0	0	0	0	10.8	37.46	73.64	108.58	126	119	96.28	74.93	63.16	63.51	65.71	71.53	83.61	102.71
El Salvador	Female	0	0	0	0	0	0	13.91	51.29	111.96	178.35	215.15	206.04	167.22	129.49	104.94	98.92	100.33	112.98	137.22	165.48
Equatorial Guinea	Male	0	0	0	0	0	0	28.81	100.47	179.18	219.94	215.43	182.85	136.22	98.79	79.04	76.57	76.51	79.01	86.75	100.22
Equatorial Guinea	Female	0	0	0	0	0	0	37.57	140.34	267.59	343.87	351.39	311.02	238.73	176.47	138.2	122.79	119.35	126.6	141.38	162.55
Eritrea	Male	0	0	0	0	0	0	28.81	100.47	179.18	219.94	215.43	182.85	136.22	98.79	79.04	76.57	76.51	79.01	86.75	100.22
Eritrea	Female	0	0	0	0	0	0	37.57	140.34	267.59	343.87	351.39	311.02	238.73	176.47	138.2	122.79	119.35	126.6	141.38	162.55
Estonia	Male	0	0	0	0	0	0	31.46	105	190.52	254.31	275.2	252.28	197.24	140.34	99.62	82	75.4	77.3	85.72	99.94

Estonia	Female	0	0	0	0	0	0	44.02	164.39	334.35	480.97	546.03	509.01	387.03	258.05	169.54	128.51	114.26	118.43	135.14	156.66
Eswatini	Male	0	0	0	0	0	0	49.38	164.05	267	295.79	263.29	205.58	142.41	97.55	76.13	73.35	74.27	77.94	85.3	97.85
Eswatini	Female	0	0	0	0	0	0	55.57	195.99	335.88	383.13	355.75	299.25	226.68	167.8	131.69	118.01	114.99	123.73	139.65	161.55
Ethiopia	Male	0	0	0	0	0	0	26.21	97.44	184.04	230.17	226.79	200.14	160.41	128.29	112.49	112.8	114.94	119.9	129.05	143.74
Ethiopia	Female	0	0	0	0	0	0	33.11	132.58	270.09	358.41	369.18	334.56	275.93	225.16	191.76	180.79	177.96	186.89	206.64	236.58
Fiji	Male	0	0	0	0	0	0	4.36	10.65	14.87	22.35	32.17	39.38	40.35	37.6	36.39	40.75	45.47	52.85	63.7	79.31
Fiji	Female	0	0	0	0	0	0	7.73	22.12	35.76	50.64	63.5	68.03	63.37	56.62	54.66	62.3	70.48	83.47	104.52	131.83
Finland	Male	0	0	0	0	0	0	17.03	43.94	65.81	101.39	149.64	176.99	160.51	119.89	84.49	68.42	61.92	62.87	72.32	90.4
Finland	Female	0	0	0	0	0	0	51.19	167.23	328.76	531.83	671.1	626.85	441.1	268.74	171.61	127.98	108.53	105.16	113.17	128.34
France	Male	0	0	0	0	0	0	39.21	105.07	164.35	248.83	352.12	393.49	318.92	204.46	124.55	87.65	73.28	69.82	75.41	88.72
France	Female	0	0	0	0	0	0	52.68	171.66	338.66	551.11	691.52	638.6	445.89	271.49	173.11	128.06	108.63	105.53	113.91	129.92
Gabon	Male	0	0	0	0	0	0	28.81	100.47	179.18	219.94	215.43	182.85	136.22	98.79	79.04	76.57	76.51	79.01	86.75	100.22
Gabon	Female	0	0	0	0	0	0	37.57	140.34	267.59	343.87	351.39	311.02	238.73	176.47	138.2	122.79	119.35	126.6	141.38	162.55
Gambia	Male	0	0	0	0	0	0	28.81	100.47	179.18	219.94	215.43	182.85	136.22	98.79	79.04	76.57	76.51	79.01	86.75	100.22
Gambia	Female	0	0	0	0	0	0	37.57	140.34	267.59	343.87	351.39	311.02	238.73	176.47	138.2	122.79	119.35	126.6	141.38	162.55
Germany	Male	0	0	0	0	0	0	19.18	49.74	74.93	114.37	167.86	197.41	177.59	131.94	92.41	73.86	65.48	64.86	72.96	89.08
Germany	Female	0	0	0	0	0	0	41.57	131.89	254.31	406.24	518.11	506.1	379.26	246.34	164.74	126.07	108.22	105.91	115.88	133.55
Ghana	Male	0	0	0	0	0	0	28.81	100.47	179.18	219.94	215.43	182.85	136.22	98.79	79.04	76.57	76.51	79.01	86.75	100.22
Ghana	Female	0	0	0	0	0	0	37.57	140.34	267.59	343.87	351.39	311.02	238.73	176.47	138.2	122.79	119.35	126.6	141.38	162.55
Greece	Male	0	0	0	0	0	0	13.45	34.01	50.63	79.71	117.65	140.88	132.34	101.77	73.84	62.67	58.35	59.65	69.54	86.85
Greece	Female	0	0	0	0	0	0	25.04	77.39	143.02	226.06	296.21	305.41	251.34	183.85	135.22	112.26	101.86	103.94	117.61	138.86
Greenland	Male	0	0	0	0	0	0	83.35	197.72	291.19	476.33	704.77	757.55	515.3	238.82	118.49	81.45	69.13	65.9	70.63	81.87
Greenland	Female	0	0	0	0	0	0	120.45	352.35	696.56	1216.94	1447.62	1055.06	491.98	190.46	97.47	75.62	74.51	84.34	98.26	115
Grenada	Male	0	0	0	0	0	0	9.64	30.84	54.33	75.84	89.95	89.88	76.32	61.7	54.81	59.52	65.62	73.19	84.05	100.02
Grenada	Female	0	0	0	0	0	0	15.17	54.74	110.24	163.85	192.97	189.52	159.44	124.96	104.56	102.52	105.99	119.89	141.44	165.86
Guam	Male	0	0	0	0	0	0	4.36	10.65	14.87	22.35	32.17	39.38	40.35	37.6	36.39	40.75	45.47	52.85	63.7	79.31
Guam	Female	0	0	0	0	0	0	7.73	22.12	35.76	50.64	63.5	68.03	63.37	56.62	54.66	62.3	70.48	83.47	104.52	131.83
Guatemala	Male	0	0	0	0	0	0	10.8	37.46	73.64	108.58	126	119	96.28	74.93	63.16	63.51	65.71	71.53	83.61	102.71
Guatemala	Female	0	0	0	0	0	0	13.91	51.29	111.96	178.35	215.15	206.04	167.22	129.49	104.94	98.92	100.33	112.98	137.22	165.48
Guinea	Male	0	0	0	0	0	0	28.81	100.47	179.18	219.94	215.43	182.85	136.22	98.79	79.04	76.57	76.51	79.01	86.75	100.22

Guinea	Female	0	0	0	0	0	0	37.57	140.34	267.59	343.87	351.39	311.02	238.73	176.47	138.2	122.79	119.35	126.6	141.38	162.55
Guinea-Bissau	Male	0	0	0	0	0	0	28.81	100.47	179.18	219.94	215.43	182.85	136.22	98.79	79.04	76.57	76.51	79.01	86.75	100.22
Guinea-Bissau	Female	0	0	0	0	0	0	37.57	140.34	267.59	343.87	351.39	311.02	238.73	176.47	138.2	122.79	119.35	126.6	141.38	162.55
Guyana	Male	0	0	0	0	0	0	9.64	30.84	54.33	75.84	89.95	89.88	76.32	61.7	54.81	59.52	65.62	73.19	84.05	100.02
Guyana	Female	0	0	0	0	0	0	15.17	54.74	110.24	163.85	192.97	189.52	159.44	124.96	104.56	102.52	105.99	119.89	141.44	165.86
Haiti	Male	0	0	0	0	0	0	9.64	30.84	54.33	75.84	89.95	89.88	76.32	61.7	54.81	59.52	65.62	73.19	84.05	100.02
Haiti	Female	0	0	0	0	0	0	15.17	54.74	110.24	163.85	192.97	189.52	159.44	124.96	104.56	102.52	105.99	119.89	141.44	165.86
Honduras	Male	0	0	0	0	0	0	10.8	37.46	73.64	108.58	126	119	96.28	74.93	63.16	63.51	65.71	71.53	83.61	102.71
Honduras	Female	0	0	0	0	0	0	13.91	51.29	111.96	178.35	215.15	206.04	167.22	129.49	104.94	98.92	100.33	112.98	137.22	165.48
Hungary	Male	0	0	0	0	0	0	15.41	53.02	101.71	147.09	173.29	171.44	140.88	104.54	82.34	77.11	74.78	76.32	84.47	98.65
Hungary	Female	0	0	0	0	0	0	16.31	56.34	110.27	165.54	205.19	212.5	184.66	146.16	117.36	108.03	106.04	113.45	131.59	155.62
Iceland	Male	0	0	0	0	0	0	115.57	285.15	394.68	689.79	1283.07	1323.37	742.22	316.13	146.96	88.86	67.22	60.43	64.2	75.57
Iceland	Female	0	0	0	0	0	0	112.82	381.6	674.8	1108.1	1521.19	1264.66	667.72	318.25	173.3	117.18	94.23	89.31	96.48	110.11
India	Male	0	0	0	0	0	0	11.13	34.93	60.97	83.99	99.31	102.63	93.61	84.03	82.44	92.44	100.51	110.31	124.02	142.61
India	Female	0	0	0	0	0	0	16.59	59.73	119.43	172.22	200.57	205.07	186.79	165.59	156.9	162.77	169.99	184.31	205.92	235.88
Indonesia	Male	0	0	0	0	0	0	2.63	5.71	7.03	8.91	11.51	12.89	13.23	14.45	16.92	23.6	30.73	40.03	52.48	69.89
Indonesia	Female	0	0	0	0	0	0	4.82	12.16	16.8	22.89	30.38	35.56	37.21	37.92	41.9	55.72	70.17	88.49	111.91	140.17
Iran (Islamic Republic of)	Male	0	0	0	0	0	0	15.74	52.41	92.14	118.33	126.26	117.91	97.98	79.94	72.04	77.86	83.87	91.4	102.52	119.23
Iran (Islamic Republic of)	Female	0	0	0	0	0	0	28.82	119.31	269.51	400.23	435.22	380.16	286.47	210.87	166.18	148.52	144.69	155.67	176.78	203.30
Irap	Male	0	0	0	0	0	0	13.23	39.99	67.53	89.71	97.52	90.43	74.27	58.1	47.85	48.18	51.44	57.42	66.95	80.68
Irap	Female	0	0	0	0	0	0	19.49	68.7	143.36	223.28	259.91	238.92	185.34	132.9	98.16	86.22	87.37	100.92	122.47	147.21
Ireland	Male	0	0	0	0	0	0	48.22	130.26	204.24	307.1	435.53	476.09	370.14	226.87	131.55	89.41	72.78	68.3	73.15	86.4
Ireland	Female	0	0	0	0	0	0	52.68	171.66	338.66	551.11	691.52	638.6	445.89	271.49	173.11	128.06	108.63	105.53	113.91	129.92
Israel	Male	0	0	0	0	0	0	71.91	199.14	318.61	482.35	681.33	705.82	492.45	268.31	144.22	92.96	72.98	66.82	71.54	84.9
Israel	Female	0	0	0	0	0	0	69.57	234.09	476.05	781.98	964.55	833.57	527.79	295.17	177.02	127.35	105.81	101.32	109.87	124.24
Italy	Male	0	0	0	0	0	0	27.48	92.8	171.35	244.72	304.2	322.08	280.23	216.81	163.09	133.31	117.52	113.62	118.35	131.12
Italy	Female	0	0	0	0	0	0	37.27	146.32	320.77	494.58	579.2	544.32	427.93	314.49	235.25	191.88	168.32	162.55	171.09	188.34
Jamaica	Male	0	0	0	0	0	0	9.64	30.84	54.33	75.84	89.95	89.88	76.32	61.7	54.81	59.52	65.62	73.19	84.05	100.02
Jamaica	Female	0	0	0	0	0	0	15.17	54.74	110.24	163.85	192.97	189.52	159.44	124.96	104.56	102.52	105.99	119.89	141.44	165.86
Japan	Male	0	0	0	0	0	0	12.57	41.62	80.18	124.3	167.13	198.14	198.58	175.35	149.05	132.59	119.53	115.19	120.88	135.06

Japan	Female	0	0	0	0	0	0	52.48	248.2	623.62	951.41	1005.7	821.13	548.08	336.78	219.23	168.16	147.91	148.94	164.11	188.54
Jordan	Male	0	0	0	0	0	0	13.23	39.99	67.53	89.71	97.52	90.43	74.27	58.1	47.85	48.18	51.44	57.42	66.95	80.68
Jordan	Female	0	0	0	0	0	0	19.49	68.7	143.36	223.28	259.91	238.92	185.34	132.9	98.16	86.22	87.37	100.92	122.47	147.21
Kazakhstan	Male	0	0	0	0	0	0	22.44	79.13	150.54	206.35	231.16	224.19	188.63	144.76	108.49	90.96	81.68	79.72	86.4	100.81
Kazakhstan	Female	0	0	0	0	0	0	33.29	135.15	304.33	468.78	541.1	504.41	383.85	254.29	167.89	127.79	114.46	119.59	136.6	159.85
Kenya	Male	0	0	0	0	0	0	26.21	97.44	184.04	230.17	226.79	200.14	160.41	128.29	112.49	112.8	114.94	119.9	129.05	143.74
Kenya	Female	0	0	0	0	0	0	33.11	132.58	270.09	358.41	369.18	334.56	275.93	225.16	191.76	180.79	177.96	186.89	206.64	236.58
Kuwait	Male	0	0	0	0	0	0	13.23	39.99	67.53	89.71	97.52	90.43	74.27	58.1	47.85	48.18	51.44	57.42	66.95	80.68
Kuwait	Female	0	0	0	0	0	0	19.49	68.7	143.36	223.28	259.91	238.92	185.34	132.9	98.16	86.22	87.37	100.92	122.47	147.21
Kyrgyzstan	Male	0	0	0	0	0	0	22.44	79.13	150.54	206.35	231.16	224.19	188.63	144.76	108.49	90.96	81.68	79.72	86.4	100.81
Kyrgyzstan	Female	0	0	0	0	0	0	33.29	135.15	304.33	468.78	541.1	504.41	383.85	254.29	167.89	127.79	114.46	119.59	136.6	159.85
Lao People's Democratic Republic	Male	0	0	0	0	0	0	1.91	4.29	5.37	6.75	8.71	10.32	10.91	11.3	12.27	16.11	20.37	27.04	37.19	52.19
Lao People's Democratic Republic	Female	0	0	0	0	0	0	4.04	10.17	13.98	20	26.9	31.14	32.38	31.57	32.36	40.1	49.06	62.43	82.34	109.8
Latvia	Male	0	0	0	0	0	0	31.46	105	190.52	254.31	275.2	252.28	197.24	140.34	99.62	82	75.4	77.3	85.72	99.94
Latvia	Female	0	0	0	0	0	0	44.02	164.39	334.35	480.97	546.03	509.01	387.03	258.05	169.54	128.51	114.26	118.43	135.14	156.66
Lebanon	Male	0	0	0	0	0	0	13.23	39.99	67.53	89.71	97.52	90.43	74.27	58.1	47.85	48.18	51.44	57.42	66.95	80.68
Lebanon	Female	0	0	0	0	0	0	19.49	68.7	143.36	223.28	259.91	238.92	185.34	132.9	98.16	86.22	87.37	100.92	122.47	147.21
Lesotho	Male	0	0	0	0	0	0	49.38	164.05	267	295.79	263.29	205.58	142.41	97.55	76.13	73.35	74.27	77.94	85.3	97.85
Lesotho	Female	0	0	0	0	0	0	55.57	195.99	335.88	383.13	355.75	299.25	226.68	167.8	131.69	118.01	114.99	123.73	139.65	161.55
Liberia	Male	0	0	0	0	0	0	28.81	100.47	179.18	219.94	215.43	182.85	136.22	98.79	79.04	76.57	76.51	79.01	86.75	100.22
Liberia	Female	0	0	0	0	0	0	37.57	140.34	267.59	343.87	351.39	311.02	238.73	176.47	138.2	122.79	119.35	126.6	141.38	162.55
Libya	Male	0	0	0	0	0	0	13.23	39.99	67.53	89.71	97.52	90.43	74.27	58.1	47.85	48.18	51.44	57.42	66.95	80.68
Libya	Female	0	0	0	0	0	0	19.49	68.7	143.36	223.28	259.91	238.92	185.34	132.9	98.16	86.22	87.37	100.92	122.47	147.21
Lithuania	Male	0	0	0	0	0	0	16.81	52.78	89.83	121.07	139.3	138.49	118.88	93.46	74.34	67.53	65.76	70.24	80.43	95.42
Lithuania	Female	0	0	0	0	0	0	27.82	96.7	185.87	266.63	312.98	313.06	265.23	199.24	143.99	116.31	107.53	115.42	135.57	161.51
Luxembourg	Male	0	0	0	0	0	0	48.22	130.26	204.24	307.1	435.53	476.09	370.14	226.87	131.55	89.41	72.78	68.3	73.15	86.4
Luxembourg	Female	0	0	0	0	0	0	52.68	171.66	338.66	551.11	691.52	638.6	445.89	271.49	173.11	128.06	108.63	105.53	113.91	129.92
Madagascar	Male	0	0	0	0	0	0	28.81	100.47	179.18	219.94	215.43	182.85	136.22	98.79	79.04	76.57	76.51	79.01	86.75	100.22
Madagascar	Female	0	0	0	0	0	0	37.57	140.34	267.59	343.87	351.39	311.02	238.73	176.47	138.2	122.79	119.35	126.6	141.38	162.55
Malawi	Male	0	0	0	0	0	0	28.81	100.47	179.18	219.94	215.43	182.85	136.22	98.79	79.04	76.57	76.51	79.01	86.75	100.22

Malawi	Female	0	0	0	0	0	0	37.57	140.34	267.59	343.87	351.39	311.02	238.73	176.47	138.2	122.79	119.35	126.6	141.38	162.55
Malaysia	Male	0	0	0	0	0	0	1.91	4.29	5.37	6.75	8.71	10.32	10.91	11.3	12.27	16.11	20.37	27.04	37.19	52.19
Malaysia	Female	0	0	0	0	0	0	4.04	10.17	13.98	20	26.9	31.14	32.38	31.57	32.36	40.1	49.06	62.43	82.34	109.8
Maldives	Male	0	0	0	0	0	0	1.91	4.29	5.37	6.75	8.71	10.32	10.91	11.3	12.27	16.11	20.37	27.04	37.19	52.19
Maldives	Female	0	0	0	0	0	0	4.04	10.17	13.98	20	26.9	31.14	32.38	31.57	32.36	40.1	49.06	62.43	82.34	109.8
Mali	Male	0	0	0	0	0	0	28.81	100.47	179.18	219.94	215.43	182.85	136.22	98.79	79.04	76.57	76.51	79.01	86.75	100.22
Mali	Female	0	0	0	0	0	0	37.57	140.34	267.59	343.87	351.39	311.02	238.73	176.47	138.2	122.79	119.35	126.6	141.38	162.55
Malta	Male	0	0	0	0	0	0	48.22	130.26	204.24	307.1	435.53	476.09	370.14	226.87	131.55	89.41	72.78	68.3	73.15	86.4
Malta	Female	0	0	0	0	0	0	52.68	171.66	338.66	551.11	691.52	638.6	445.89	271.49	173.11	128.06	108.63	105.53	113.91	129.92
Marshall Islands	Male	0	0	0	0	0	0	4.36	10.65	14.87	22.35	32.17	39.38	40.35	37.6	36.39	40.75	45.47	52.85	63.7	79.31
Marshall Islands	Female	0	0	0	0	0	0	7.73	22.12	35.76	50.64	63.5	68.03	63.37	56.62	54.66	62.3	70.48	83.47	104.52	131.83
Mauritania	Male	0	0	0	0	0	0	28.81	100.47	179.18	219.94	215.43	182.85	136.22	98.79	79.04	76.57	76.51	79.01	86.75	100.22
Mauritania	Female	0	0	0	0	0	0	37.57	140.34	267.59	343.87	351.39	311.02	238.73	176.47	138.2	122.79	119.35	126.6	141.38	162.55
Mauritius	Male	0	0	0	0	0	0	1.91	4.29	5.37	6.75	8.71	10.32	10.91	11.3	12.27	16.11	20.37	27.04	37.19	52.19
Mauritius	Female	0	0	0	0	0	0	4.04	10.17	13.98	20	26.9	31.14	32.38	31.57	32.36	40.1	49.06	62.43	82.34	109.8
Mexico	Male	0	0	0	0	0	0	28.58	128.09	317.72	487.27	490.31	366.17	234.81	156.14	120.97	111.71	109.57	113.81	125.01	143.07
Mexico	Female	0	0	0	0	0	0	28.8	134.87	360.94	599.05	650.02	516.73	346.54	237.39	184.98	168.14	164.08	173.25	195.67	226.35
Micronesia (Federated States of)	Male	0	0	0	0	0	0	4.36	10.65	14.87	22.35	32.17	39.38	40.35	37.6	36.39	40.75	45.47	52.85	63.7	79.31
Micronesia (Federated States of)	Female	0	0	0	0	0	0	7.73	22.12	35.76	50.64	63.5	68.03	63.37	56.62	54.66	62.3	70.48	83.47	104.52	131.83
Mongolia	Male	0	0	0	0	0	0	22.44	79.13	150.54	206.35	231.16	224.19	188.63	144.76	108.49	90.96	81.68	79.72	86.4	100.81
Mongolia	Female	0	0	0	0	0	0	33.29	135.15	304.33	468.78	541.1	504.41	383.85	254.29	167.89	127.79	114.46	119.59	136.6	159.85
Montenegro	Male	0	0	0	0	0	0	15.41	53.02	101.71	147.09	173.29	171.44	140.88	104.54	82.34	77.11	74.78	76.32	84.47	98.65
Montenegro	Female	0	0	0	0	0	0	16.31	56.34	110.27	165.54	205.19	212.5	184.66	146.16	117.36	108.03	106.04	113.45	131.59	155.62
Morocco	Male	0	0	0	0	0	0	13.23	39.99	67.53	89.71	97.52	90.43	74.27	58.1	47.85	48.18	51.44	57.42	66.95	80.68
Morocco	Female	0	0	0	0	0	0	19.49	68.7	143.36	223.28	259.91	238.92	185.34	132.9	98.16	86.22	87.37	100.92	122.47	147.21
Mozambique	Male	0	0	0	0	0	0	28.81	100.47	179.18	219.94	215.43	182.85	136.22	98.79	79.04	76.57	76.51	79.01	86.75	100.22
Mozambique	Female	0	0	0	0	0	0	37.57	140.34	267.59	343.87	351.39	311.02	238.73	176.47	138.2	122.79	119.35	126.6	141.38	162.55
Myanmar	Male	0	0	0	0	0	0	1.91	4.29	5.37	6.75	8.71	10.32	10.91	11.3	12.27	16.11	20.37	27.04	37.19	52.19
Myanmar	Female	0	0	0	0	0	0	4.04	10.17	13.98	20	26.9	31.14	32.38	31.57	32.36	40.1	49.06	62.43	82.34	109.8
Namibia	Male	0	0	0	0	0	0	49.38	164.05	267	295.79	263.29	205.58	142.41	97.55	76.13	73.35	74.27	77.94	85.3	97.85

Namibia	Female	0	0	0	0	0	0	55.57	195.99	335.88	383.13	355.75	299.25	226.68	167.8	131.69	118.01	114.99	123.73	139.65	161.55
Nepal	Male	0	0	0	0	0	0	10.3	32.23	56.19	78.75	94.37	96.7	84.7	69.74	62.26	66.26	70.58	75.56	84.16	99.57
Nepal	Female	0	0	0	0	0	0	16.31	57.17	111.43	161.42	191.83	195.03	170.6	138.37	116.81	114.17	116.75	126.39	142.86	165.84
Netherlands	Male	0	0	0	0	0	0	36.02	95.73	145.28	213.17	295.24	326.79	270.26	180.01	113.65	83.97	72.02	69.6	74.95	88.42
Netherlands	Female	0	0	0	0	0	0	38.96	123.32	234.18	369.41	468.25	454.21	341.72	227.98	157.36	124.13	109.02	108.72	119.82	138.05
New Zealand	Male	0	0	0	0	0	0	24.78	90.63	182.4	266.44	319.45	326.56	283.86	224.17	173.61	145.92	129.43	123.98	127.57	139.94
New Zealand	Female	0	0	0	0	0	0	55	263.59	681.97	1068.45	1146.46	917.82	583.63	349.34	227.7	177.85	159.21	155.95	165.22	186.35
Nicaragua	Male	0	0	0	0	0	0	10.8	37.46	73.64	108.58	126	119	96.28	74.93	63.16	63.51	65.71	71.53	83.61	102.71
Nicaragua	Female	0	0	0	0	0	0	13.91	51.29	111.96	178.35	215.15	206.04	167.22	129.49	104.94	98.92	100.33	112.98	137.22	165.48
Niger	Male	0	0	0	0	0	0	28.81	100.47	179.18	219.94	215.43	182.85	136.22	98.79	79.04	76.57	76.51	79.01	86.75	100.22
Niger	Female	0	0	0	0	0	0	37.57	140.34	267.59	343.87	351.39	311.02	238.73	176.47	138.2	122.79	119.35	126.6	141.38	162.55
Nigeria	Male	0	0	0	0	0	0	26.21	97.44	184.04	230.17	226.79	200.14	160.41	128.29	112.49	112.8	114.94	119.9	129.05	143.74
Nigeria	Female	0	0	0	0	0	0	33.11	132.58	270.09	358.41	369.18	334.56	275.93	225.16	191.76	180.79	177.96	186.89	206.64	236.58
North Macedonia	Male	0	0	0	0	0	0	15.41	53.02	101.71	147.09	173.29	171.44	140.88	104.54	82.34	77.11	74.78	76.32	84.47	98.65
North Macedonia	Female	0	0	0	0	0	0	16.31	56.34	110.27	165.54	205.19	212.5	184.66	146.16	117.36	108.03	106.04	113.45	131.59	155.62
Northern Mariana Islands	Male	0	0	0	0	0	0	4.36	10.65	14.87	22.35	32.17	39.38	40.35	37.6	36.39	40.75	45.47	52.85	63.7	79.31
Northern Mariana Islands	Female	0	0	0	0	0	0	7.73	22.12	35.76	50.64	63.5	68.03	63.37	56.62	54.66	62.3	70.48	83.47	104.52	131.83
Norway	Male	0	0	0	0	0	0	40.54	142.56	274.72	397.95	488.15	502.46	410.03	286.8	195.85	146	121.17	112.31	114.99	126.2
Norway	Female	0	0	0	0	0	0	62.41	267.81	634.43	972.69	1075.11	912.91	623.84	395.15	263.51	199.41	165.72	154.12	158.49	172.84
Oman	Male	0	0	0	0	0	0	13.23	39.99	67.53	89.71	97.52	90.43	74.27	58.1	47.85	48.18	51.44	57.42	66.95	80.68
Oman	Female	0	0	0	0	0	0	19.49	68.7	143.36	223.28	259.91	238.92	185.34	132.9	98.16	86.22	87.37	100.92	122.47	147.21
Pakistan	Male	0	0	0	0	0	0	11.13	34.93	60.97	83.99	99.31	102.63	93.61	84.03	82.44	92.44	100.51	110.31	124.02	142.61
Pakistan	Female	0	0	0	0	0	0	16.59	59.73	119.43	172.22	200.57	205.07	186.79	165.59	156.9	162.77	169.99	184.31	205.92	235.88
Palestine	Male	0	0	0	0	0	0	13.23	39.99	67.53	89.71	97.52	90.43	74.27	58.1	47.85	48.18	51.44	57.42	66.95	80.68
Palestine	Female	0	0	0	0	0	0	19.49	68.7	143.36	223.28	259.91	238.92	185.34	132.9	98.16	86.22	87.37	100.92	122.47	147.21
Panama	Male	0	0	0	0	0	0	10.8	37.46	73.64	108.58	126	119	96.28	74.93	63.16	63.51	65.71	71.53	83.61	102.71
Panama	Female	0	0	0	0	0	0	13.91	51.29	111.96	178.35	215.15	206.04	167.22	129.49	104.94	98.92	100.33	112.98	137.22	165.48
Papua New Guinea	Male	0	0	0	0	0	0	4.36	10.65	14.87	22.35	32.17	39.38	40.35	37.6	36.39	40.75	45.47	52.85	63.7	79.31
Papua New Guinea	Female	0	0	0	0	0	0	7.73	22.12	35.76	50.64	63.5	68.03	63.37	56.62	54.66	62.3	70.48	83.47	104.52	131.83
Paraguay	Male	0	0	0	0	0	0	9.64	30.84	54.33	75.84	89.95	89.88	76.32	61.7	54.81	59.52	65.62	73.19	84.05	100.02

Paraguay	Female	0	0	0	0	0	0	15.17	54.74	110.24	163.85	192.97	189.52	159.44	124.96	104.56	102.52	105.99	119.89	141.44	165.86
Peru	Male	0	0	0	0	0	0	9.25	30.22	55.73	81.73	97.25	94.82	79.99	65.36	56.88	57.74	61.15	68.5	81.85	102.35
Peru	Female	0	0	0	0	0	0	15.39	55.57	117.4	183.53	220.15	213.15	175.68	136.07	108.78	100.51	101.37	113.28	135.05	164.01
Philippines	Male	0	0	0	0	0	0	2.63	5.71	7.03	8.91	11.51	12.89	13.23	14.45	16.92	23.6	30.73	40.03	52.48	69.89
Philippines	Female	0	0	0	0	0	0	4.82	12.16	16.8	22.89	30.38	35.56	37.21	37.92	41.9	55.72	70.17	88.49	111.91	140.17
Poland	Male	0	0	0	0	0	0	15.81	55.74	109.54	156.11	178.19	176.44	153.94	128.02	113.4	113.08	113.5	117.37	125.84	141.79
Poland	Female	0	0	0	0	0	0	16.38	59.49	120.63	176.4	210.68	218.96	201	175.97	158.08	157.31	159.41	168.49	187.9	214.76
Portugal	Male	0	0	0	0	0	0	32.73	86.64	132.6	199.57	286.34	327.12	274.67	184.38	117.26	85.59	72.09	68.21	73.68	86.89
Portugal	Female	0	0	0	0	0	0	68.09	228.94	464.35	758.21	931.14	810.78	520.86	292.66	175.66	126.65	105.26	100.85	109.54	126.13
Puerto Rico	Male	0	0	0	0	0	0	9.64	30.84	54.33	75.84	89.95	89.88	76.32	61.7	54.81	59.52	65.62	73.19	84.05	100.02
Puerto Rico	Female	0	0	0	0	0	0	15.17	54.74	110.24	163.85	192.97	189.52	159.44	124.96	104.56	102.52	105.99	119.89	141.44	165.86
Qatar	Male	0	0	0	0	0	0	13.23	39.99	67.53	89.71	97.52	90.43	74.27	58.1	47.85	48.18	51.44	57.42	66.95	80.68
Qatar	Female	0	0	0	0	0	0	19.49	68.7	143.36	223.28	259.91	238.92	185.34	132.9	98.16	86.22	87.37	100.92	122.47	147.21
Republic of Korea	Male	0	0	0	0	0	0	15.59	48.7	90.12	145.81	209.38	251.16	237.99	183.95	127.61	94.58	78.2	73.15	78.02	90.38
Republic of Korea	Female	0	0	0	0	0	0	93.1	409.36	997.06	1542.7	1575.62	1100.14	563.16	258.82	132.98	90.13	79.43	85.59	99.97	118.05
Republic of Moldova	Male	0	0	0	0	0	0	31.46	105	190.52	254.31	275.2	252.28	197.24	140.34	99.62	82	75.4	77.3	85.72	99.94
Republic of Moldova	Female	0	0	0	0	0	0	44.02	164.39	334.35	480.97	546.03	509.01	387.03	258.05	169.54	128.51	114.26	118.43	135.14	156.66
Romania	Male	0	0	0	0	0	0	15.41	53.02	101.71	147.09	173.29	171.44	140.88	104.54	82.34	77.11	74.78	76.32	84.47	98.65
Romania	Female	0	0	0	0	0	0	16.31	56.34	110.27	165.54	205.19	212.5	184.66	146.16	117.36	108.03	106.04	113.45	131.59	155.62
Russian Federation	Male	0	0	0	0	0	0	68.06	317.61	717.11	932.85	835.05	602.01	383.29	243.61	168.74	132.22	115.5	111.61	116.25	128.38
Russian Federation	Female	0	0	0	0	0	0	73.26	361.38	903.42	1307.87	1283.71	977.22	625.51	384.12	253.31	191.71	163.55	156.28	165.4	184.94
Rwanda	Male	0	0	0	0	0	0	28.81	100.47	179.18	219.94	215.43	182.85	136.22	98.79	79.04	76.57	76.51	79.01	86.75	100.22
Rwanda	Female	0	0	0	0	0	0	37.57	140.34	267.59	343.87	351.39	311.02	238.73	176.47	138.2	122.79	119.35	126.6	141.38	162.55
Saint Lucia	Male	0	0	0	0	0	0	9.64	30.84	54.33	75.84	89.95	89.88	76.32	61.7	54.81	59.52	65.62	73.19	84.05	100.02
Saint Lucia	Female	0	0	0	0	0	0	15.17	54.74	110.24	163.85	192.97	189.52	159.44	124.96	104.56	102.52	105.99	119.89	141.44	165.86
Saint Vincent and the Grenadines	Male	0	0	0	0	0	0	9.64	30.84	54.33	75.84	89.95	89.88	76.32	61.7	54.81	59.52	65.62	73.19	84.05	100.02
Saint Vincent and the Grenadines	Female	0	0	0	0	0	0	15.17	54.74	110.24	163.85	192.97	189.52	159.44	124.96	104.56	102.52	105.99	119.89	141.44	165.86
Samoa	Male	0	0	0	0	0	0	4.36	10.65	14.87	22.35	32.17	39.38	40.35	37.6	36.39	40.75	45.47	52.85	63.7	79.31
Samoa	Female	0	0	0	0	0	0	7.73	22.12	35.76	50.64	63.5	68.03	63.37	56.62	54.66	62.3	70.48	83.47	104.52	131.83
Sao Tome and Principe	Male	0	0	0	0	0	0	28.81	100.47	179.18	219.94	215.43	182.85	136.22	98.79	79.04	76.57	76.51	79.01	86.75	100.22

Sao Tome and Principe	Female	0	0	0	0	0	0	37.57	140.34	267.59	343.87	351.39	311.02	238.73	176.47	138.2	122.79	119.35	126.6	141.38	162.55
Saudi Arabia	Male	0	0	0	0	0	0	39.47	142.83	280.38	356.91	318.18	227.59	142.82	90.11	65.91	60.45	60.03	63.2	70.32	81.07
Saudi Arabia	Female	0	0	0	0	0	0	40.22	154.42	357.09	566.59	607.03	467.09	290.75	175.5	116.34	93.79	90.63	101.5	119.86	140.53
Senegal	Male	0	0	0	0	0	0	28.81	100.47	179.18	219.94	215.43	182.85	136.22	98.79	79.04	76.57	76.51	79.01	86.75	100.22
Senegal	Female	0	0	0	0	0	0	37.57	140.34	267.59	343.87	351.39	311.02	238.73	176.47	138.2	122.79	119.35	126.6	141.38	162.55
Serbia	Male	0	0	0	0	0	0	15.41	53.02	101.71	147.09	173.29	171.44	140.88	104.54	82.34	77.11	74.78	76.32	84.47	98.65
Serbia	Female	0	0	0	0	0	0	16.31	56.34	110.27	165.54	205.19	212.5	184.66	146.16	117.36	108.03	106.04	113.45	131.59	155.62
Seychelles	Male	0	0	0	0	0	0	1.91	4.29	5.37	6.75	8.71	10.32	10.91	11.3	12.27	16.11	20.37	27.04	37.19	52.19
Seychelles	Female	0	0	0	0	0	0	4.04	10.17	13.98	20	26.9	31.14	32.38	31.57	32.36	40.1	49.06	62.43	82.34	109.8
Sierra Leone	Male	0	0	0	0	0	0	28.81	100.47	179.18	219.94	215.43	182.85	136.22	98.79	79.04	76.57	76.51	79.01	86.75	100.22
Sierra Leone	Female	0	0	0	0	0	0	37.57	140.34	267.59	343.87	351.39	311.02	238.73	176.47	138.2	122.79	119.35	126.6	141.38	162.55
Singapore	Male	0	0	0	0	0	0	14.15	42.89	77.43	124.64	180.63	220.76	213.1	168.41	121.21	92.91	77.85	73.03	78.46	91.37
Singapore	Female	0	0	0	0	0	0	75.36	326.83	782.08	1209.69	1286.55	971.01	541.29	262.75	138.85	95.89	84.63	90.81	105.78	123.81
Slovakia	Male	0	0	0	0	0	0	15.41	53.02	101.71	147.09	173.29	171.44	140.88	104.54	82.34	77.11	74.78	76.32	84.47	98.65
Slovakia	Female	0	0	0	0	0	0	16.31	56.34	110.27	165.54	205.19	212.5	184.66	146.16	117.36	108.03	106.04	113.45	131.59	155.62
Slovenia	Male	0	0	0	0	0	0	15.41	53.02	101.71	147.09	173.29	171.44	140.88	104.54	82.34	77.11	74.78	76.32	84.47	98.65
Slovenia	Female	0	0	0	0	0	0	16.31	56.34	110.27	165.54	205.19	212.5	184.66	146.16	117.36	108.03	106.04	113.45	131.59	155.62
Solomon Islands	Male	0	0	0	0	0	0	4.36	10.65	14.87	22.35	32.17	39.38	40.35	37.6	36.39	40.75	45.47	52.85	63.7	79.31
Solomon Islands	Female	0	0	0	0	0	0	7.73	22.12	35.76	50.64	63.5	68.03	63.37	56.62	54.66	62.3	70.48	83.47	104.52	131.83
Somalia	Male	0	0	0	0	0	0	28.81	100.47	179.18	219.94	215.43	182.85	136.22	98.79	79.04	76.57	76.51	79.01	86.75	100.22
Somalia	Female	0	0	0	0	0	0	37.57	140.34	267.59	343.87	351.39	311.02	238.73	176.47	138.2	122.79	119.35	126.6	141.38	162.55
South Africa	Male	0	0	0	0	0	0	40.6	150.74	271.06	313.02	280.77	228.61	173.92	131.89	110.76	109.97	112.23	118.64	129.33	146.01
South Africa	Female	0	0	0	0	0	0	45.29	176.88	334.6	407.58	387.27	331.86	266.59	216.74	186.18	176.08	174.25	183.25	201.92	227.07
South Sudan	Male	0	0	0	0	0	0	28.81	100.47	179.18	219.94	215.43	182.85	136.22	98.79	79.04	76.57	76.51	79.01	86.75	100.22
South Sudan	Female	0	0	0	0	0	0	37.57	140.34	267.59	343.87	351.39	311.02	238.73	176.47	138.2	122.79	119.35	126.6	141.38	162.55
Spain	Male	0	0	0	0	0	0	36.38	101.59	168.72	274.83	422.11	476.44	370.63	228.36	134.64	92.73	75.51	70.18	74.41	86.98
Spain	Female	0	0	0	0	0	0	56.67	198.47	455.18	881.55	1194.82	1009.22	572.28	288.49	170.08	123.58	102.22	97.14	105.68	121.37
Sri Lanka	Male	0	0	0	0	0	0	1.91	4.29	5.37	6.75	8.71	10.32	10.91	11.3	12.27	16.11	20.37	27.04	37.19	52.19
Sri Lanka	Female	0	0	0	0	0	0	4.04	10.17	13.98	20	26.9	31.14	32.38	31.57	32.36	40.1	49.06	62.43	82.34	109.8
Sudan	Male	0	0	0	0	0	0	13.23	39.99	67.53	89.71	97.52	90.43	74.27	58.1	47.85	48.18	51.44	57.42	66.95	80.68

Sudan	Female	0	0	0	0	0	0	19.49	68.7	143.36	223.28	259.91	238.92	185.34	132.9	98.16	86.22	87.37	100.92	122.47	147.21
Suriname	Male	0	0	0	0	0	0	9.64	30.84	54.33	75.84	89.95	89.88	76.32	61.7	54.81	59.52	65.62	73.19	84.05	100.02
Suriname	Female	0	0	0	0	0	0	15.17	54.74	110.24	163.85	192.97	189.52	159.44	124.96	104.56	102.52	105.99	119.89	141.44	165.86
Sweden	Male	0	0	0	0	0	0	20.76	66.66	120.47	173.81	221.35	243.1	221.17	177.02	138.73	120.46	110.67	108.9	116.05	129.26
Sweden	Female	0	0	0	0	0	0	24.46	89.82	184.69	274.7	332.71	340.11	292.87	235.71	193.03	171.71	159.99	160.2	172.2	193.51
Switzerland	Male	0	0	0	0	0	0	48.22	130.26	204.24	307.1	435.53	476.09	370.14	226.87	131.55	89.41	72.78	68.3	73.15	86.4
Switzerland	Female	0	0	0	0	0	0	52.68	171.66	338.66	551.11	691.52	638.6	445.89	271.49	173.11	128.06	108.63	105.53	113.91	129.92
Syrian Arab Republic	Male	0	0	0	0	0	0	13.23	39.99	67.53	89.71	97.52	90.43	74.27	58.1	47.85	48.18	51.44	57.42	66.95	80.68
Syrian Arab Republic	Female	0	0	0	0	0	0	19.49	68.7	143.36	223.28	259.91	238.92	185.34	132.9	98.16	86.22	87.37	100.92	122.47	147.21
Tajikistan	Male	0	0	0	0	0	0	22.44	79.13	150.54	206.35	231.16	224.19	188.63	144.76	108.49	90.96	81.68	79.72	86.4	100.81
Tajikistan	Female	0	0	0	0	0	0	33.29	135.15	304.33	468.78	541.1	504.41	383.85	254.29	167.89	127.79	114.46	119.59	136.6	159.85
Thailand	Male	0	0	0	0	0	0	1.91	4.29	5.37	6.75	8.71	10.32	10.91	11.3	12.27	16.11	20.37	27.04	37.19	52.19
Thailand	Female	0	0	0	0	0	0	4.04	10.17	13.98	20	26.9	31.14	32.38	31.57	32.36	40.1	49.06	62.43	82.34	109.8
Timor-Leste	Male	0	0	0	0	0	0	1.91	4.29	5.37	6.75	8.71	10.32	10.91	11.3	12.27	16.11	20.37	27.04	37.19	52.19
Timor-Leste	Female	0	0	0	0	0	0	4.04	10.17	13.98	20	26.9	31.14	32.38	31.57	32.36	40.1	49.06	62.43	82.34	109.8
Togo	Male	0	0	0	0	0	0	28.81	100.47	179.18	219.94	215.43	182.85	136.22	98.79	79.04	76.57	76.51	79.01	86.75	100.22
Togo	Female	0	0	0	0	0	0	37.57	140.34	267.59	343.87	351.39	311.02	238.73	176.47	138.2	122.79	119.35	126.6	141.38	162.55
Tonga	Male	0	0	0	0	0	0	4.36	10.65	14.87	22.35	32.17	39.38	40.35	37.6	36.39	40.75	45.47	52.85	63.7	79.31
Tonga	Female	0	0	0	0	0	0	7.73	22.12	35.76	50.64	63.5	68.03	63.37	56.62	54.66	62.3	70.48	83.47	104.52	131.83
Trinidad and Tobago	Male	0	0	0	0	0	0	9.64	30.84	54.33	75.84	89.95	89.88	76.32	61.7	54.81	59.52	65.62	73.19	84.05	100.02
Trinidad and Tobago	Female	0	0	0	0	0	0	15.17	54.74	110.24	163.85	192.97	189.52	159.44	124.96	104.56	102.52	105.99	119.89	141.44	165.86
Tunisia	Male	0	0	0	0	0	0	13.23	39.99	67.53	89.71	97.52	90.43	74.27	58.1	47.85	48.18	51.44	57.42	66.95	80.68
Tunisia	Female	0	0	0	0	0	0	19.49	68.7	143.36	223.28	259.91	238.92	185.34	132.9	98.16	86.22	87.37	100.92	122.47	147.21
Turkey	Male	0	0	0	0	0	0	3.2	8.27	12.73	18.32	22.51	24.13	23.32	20.67	19.06	22.03	26.74	35.84	48.71	66.39
Turkey	Female	0	0	0	0	0	0	5.66	17.13	33.34	55.29	71.59	74.6	67	56.57	49.46	51.82	58.58	73.81	99.66	133.66
Turkmenistan	Male	0	0	0	0	0	0	25.52	90.91	175.28	240.8	264.11	248.43	204.19	154.43	114.31	93.24	81.6	79.64	87.39	101.63
Turkmenistan	Female	0	0	0	0	0	0	21.49	81.14	172.8	265.51	321.68	322.17	268.83	199.06	142.23	114.37	106.37	115.2	135.54	162.14
Uganda	Male	0	0	0	0	0	0	28.81	100.47	179.18	219.94	215.43	182.85	136.22	98.79	79.04	76.57	76.51	79.01	86.75	100.22
Uganda	Female	0	0	0	0	0	0	37.57	140.34	267.59	343.87	351.39	311.02	238.73	176.47	138.2	122.79	119.35	126.6	141.38	162.55
Ukraine	Male	0	0	0	0	0	0	20.51	71.44	134.1	178.59	193.63	188.18	164.24	135.6	115.4	110.52	108.37	113.21	125.38	142.24

Ukraine	Female	0	0	0	0	0	0	31.23	124.67	267.76	387.62	435.29	419.43	350.76	274.97	218.5	188.16	173.1	174.93	191.11	217.42
United Arab Emirates	Male	0	0	0	0	0	0	13.23	39.99	67.53	89.71	97.52	90.43	74.27	58.1	47.85	48.18	51.44	57.42	66.95	80.68
United Arab Emirates	Female	0	0	0	0	0	0	19.49	68.7	143.36	223.28	259.91	238.92	185.34	132.9	98.16	86.22	87.37	100.92	122.47	147.21
United Kingdom	Male	0	0	0	0	0	0	37.43	121.68	221	320.5	401.56	416.39	342.51	243.7	169.76	131.25	113.04	106.66	109.63	121.54
United Kingdom	Female	0	0	0	0	0	0	42.26	161.53	346.26	532.15	624.72	581.07	444.06	313.58	228.17	184.55	162.32	155.74	163.32	179.3
United Republic of Tanzania	Male	0	0	0	0	0	0	28.81	100.47	179.18	219.94	215.43	182.85	136.22	98.79	79.04	76.57	76.51	79.01	86.75	100.22
United Republic of Tanzania	Female	0	0	0	0	0	0	37.57	140.34	267.59	343.87	351.39	311.02	238.73	176.47	138.2	122.79	119.35	126.6	141.38	162.55
United States of America	Male	0	0	0	0	0	0	99.88	226.82	327.44	555.06	862.96	973.04	708.33	368.52	197.23	133.92	109.99	102.53	104.9	115.14
United States of America	Female	0	0	0	0	0	0	147.26	395.34	738.69	1367.86	1794.59	1456.74	772.01	334.32	173.86	125.8	112.78	115.78	127.54	145.44
United States Virgin Islands	Male	0	0	0	0	0	0	9.64	30.84	54.33	75.84	89.95	89.88	76.32	61.7	54.81	59.52	65.62	73.19	84.05	100.02
United States Virgin Islands	Female	0	0	0	0	0	0	15.17	54.74	110.24	163.85	192.97	189.52	159.44	124.96	104.56	102.52	105.99	119.89	141.44	165.86
Uruguay	Male	0	0	0	0	0	0	28.21	89.98	163.3	241.06	310.76	332.12	274.99	189.13	125.51	95.53	82	77.84	82.59	93.46
Uruguay	Female	0	0	0	0	0	0	67.37	270.3	610.21	940.34	1040.86	828.82	486.5	253.62	148.16	110.62	99.69	104.1	117.8	136.04
Uzbekistan	Male	0	0	0	0	0	0	22.44	79.13	150.54	206.35	231.16	224.19	188.63	144.76	108.49	90.96	81.68	79.72	86.4	100.81
Uzbekistan	Female	0	0	0	0	0	0	33.29	135.15	304.33	468.78	541.1	504.41	383.85	254.29	167.89	127.79	114.46	119.59	136.6	159.85
Vanuatu	Male	0	0	0	0	0	0	4.36	10.65	14.87	22.35	32.17	39.38	40.35	37.6	36.39	40.75	45.47	52.85	63.7	79.31
Vanuatu	Female	0	0	0	0	0	0	7.73	22.12	35.76	50.64	63.5	68.03	63.37	56.62	54.66	62.3	70.48	83.47	104.52	131.83
Venezuela	Male	0	0	0	0	0	0	3.79	11.03	19.8	31.93	41.55	44.12	40.74	35.61	33.08	36.15	41.12	51.15	66.35	87.94
Venezuela	Female	0	0	0	0	0	0	6.34	21.49	43.93	71.2	91.65	97.04	88.44	75.45	67.57	71.31	78.33	94.02	120.82	155.41
Viet Nam	Male	0	0	0	0	0	0	1.91	4.29	5.37	6.75	8.71	10.32	10.91	11.3	12.27	16.11	20.37	27.04	37.19	52.19
Viet Nam	Female	0	0	0	0	0	0	4.04	10.17	13.98	20	26.9	31.14	32.38	31.57	32.36	40.1	49.06	62.43	82.34	109.8
Yemen	Male	0	0	0	0	0	0	13.23	39.99	67.53	89.71	97.52	90.43	74.27	58.1	47.85	48.18	51.44	57.42	66.95	80.68
Yemen	Female	0	0	0	0	0	0	19.49	68.7	143.36	223.28	259.91	238.92	185.34	132.9	98.16	86.22	87.37	100.92	122.47	147.21
Zambia	Male	0	0	0	0	0	0	28.81	100.47	179.18	219.94	215.43	182.85	136.22	98.79	79.04	76.57	76.51	79.01	86.75	100.22
Zambia	Female	0	0	0	0	0	0	37.57	140.34	267.59	343.87	351.39	311.02	238.73	176.47	138.2	122.79	119.35	126.6	141.38	162.55
Zimbabwe	Male	0	0	0	0	0	0	49.38	164.05	267	295.79	263.29	205.58	142.41	97.55	76.13	73.35	74.27	77.94	85.3	97.85
Zimbabwe	Female	0	0	0	0	0	0	55.57	195.99	335.88	383.13	355.75	299.25	226.68	167.8	131.69	118.01	114.99	123.73	139.65	161.55

Supplementary Table S5. The DALYs and age-standardized DALYs rate of hand osteoarthritis in 1990 and 2019, and its temporal trends from 1990 to 2019.

Supplementary Table S5. The DALYs cases and age-standardized DALYs rate of hand osteoarthritis in 1990 and 2019, and its temporal trends from 1990 to 2019.

Nation	Sex	DALYs cases No. (95% UI)		change absolute number (%)	DALYs per 100,000 No. (95% UI)		1990-2019 EAPC No. (95% CI)
		1990	2019		1990	2019	
Afghanistan	Both	20.55 [9.96-43.75]	38.79 [19.03-80.26]	88.76	28.06 [13.85-59.57]	28.79 [14.25-60.53]	0.11 [0.09 to 0.13]
Albania	Both	6.7 [3.3-14.21]	13.36 [6.51-27.9]	99.4	31.65 [15.46-66.8]	31.66 [15.37-66.95]	0 [0 to 0.01]
Algeria	Both	36.84 [17.8-77.78]	101.54 [49.68-213.09]	175.62	29.16 [14.26-61.47]	28.77 [14.12-60.19]	-0.06 [-0.07 to -0.05]
American Samoa	Both	0.02 [0.01-0.04]	0.04 [0.02-0.09]	100	8.79 [4.2-18.47]	8.84 [4.27-18.42]	0.03 [0.02 to 0.03]
Andorra	Both	0.8 [0.4-1.66]	1.96 [0.98-4.13]	145	139.66 [70.47-292.77]	139.19 [69.86-293.69]	0.78 [0.51 to 1.04]
Angola	Both	21.78 [10.66-45.22]	65.5 [31.91-136.27]	200.73	51.79 [25.79-107.57]	52.94 [26.08-109.49]	0.08 [0.08 to 0.08]
Antigua and Barbuda	Both	0.13 [0.06-0.27]	0.25 [0.12-0.53]	92.31	24.92 [12.25-52.16]	24.31 [11.78-49.85]	-0.09 [-0.09 to -0.08]
Argentina	Both	339.7 [170.03-709.92]	549.78 [271.8-1141.54]	61.84	105.2 [52.72-219.98]	104.86 [52.01-218.47]	-0.01 [-0.02 to 0]
Armenia	Both	17.87 [8.77-37.88]	26.76 [13.14-56]	49.75	64.58 [31.46-135.8]	64.49 [31.75-134.35]	-0.01 [-0.02 to -0.01]
Australia	Both	200.41 [99.71-422.66]	418.93 [208.25-870.93]	109.04	105.22 [52.6-221.89]	109.07 [54.94-227.69]	-0.29 [-0.45 to -0.13]
Austria	Both	136.34 [68.36-289.68]	202.84 [100.85-433.78]	48.78	124.42 [62.77-259.45]	127.1 [63.29-267.09]	0.78 [0.55 to 1.01]
Azerbaijan	Both	37.99 [18.65-80.11]	73.22 [36.08-155.08]	92.73	74.99 [37.03-155.63]	72.32 [35.63-150.62]	0.2 [0.01 to 0.4]
Bahamas	Both	0.39 [0.19-0.82]	1 [0.48-2.11]	156.41	24.98 [12.3-52.15]	24.67 [11.96-51.66]	-0.06 [-0.06 to -0.05]
Bahrain	Both	0.56 [0.27-1.19]	3.38 [1.63-7.05]	503.57	27.86 [13.58-59.15]	26.26 [12.91-55.22]	-0.21 [-0.24 to -0.18]
Bangladesh	Both	110.97 [53.87-234.67]	320.32 [153.99-659.36]	188.65	23.6 [11.59-48.95]	24.23 [11.77-49.91]	0.1 [0.08 to 0.12]
Barbados	Both	0.69 [0.34-1.42]	1.19 [0.58-2.44]	72.46	25.19 [12.43-52.05]	24.59 [12.09-50.75]	-0.08 [-0.08 to -0.07]
Belarus	Both	93.93 [46.32-198.23]	112.13 [55.77-236.31]	19.38	72.32 [35.9-151.65]	71.86 [35.94-149.68]	-0.01 [-0.02 to 0]
Belgium	Both	156.69 [77.47-332.17]	218.39 [108.37-459.93]	39.38	107.88 [53.33-226.03]	108.04 [53.65-225]	0.4 [0.27 to 0.52]
Belize	Both	0.22 [0.11-0.46]	0.68 [0.33-1.42]	209.09	23.97 [11.65-50.35]	23.76 [11.62-48.74]	-0.03 [-0.04 to -0.02]
Benin	Both	10.56 [5.16-21.9]	27.43 [13.31-56.63]	159.75	52.05 [25.64-108.32]	52.64 [25.71-109.36]	0.04 [0.03 to 0.05]
Bermuda	Both	0.16 [0.08-0.33]	0.31 [0.15-0.64]	93.75	25.05 [12.33-51.9]	24.66 [12.1-51.44]	-0.05 [-0.06 to -0.05]
Bhutan	Both	0.61 [0.3-1.28]	1.38 [0.68-2.88]	126.23	24.81 [12.19-51.5]	24.3 [11.97-50.79]	-0.06 [-0.08 to -0.04]
Bolivia(Plurinational State of)	Both	11.7 [5.7-24.49]	32.18 [15.69-65.63]	175.04	35.98 [17.54-75.45]	35.68 [17.43-73.72]	-0.02 [-0.03 to -0.02]
Bosnia and Herzegovina	Both	13.38 [6.52-28.33]	18.29 [8.95-38.3]	36.7	31.71 [15.47-66.91]	31.43 [15.29-65.52]	-0.03 [-0.04 to -0.03]
Botswana	Both	3.87 [1.92-8.11]	9.75 [4.82-20.12]	151.94	64.28 [31.98-133.7]	63.74 [31.7-131.63]	-0.02 [-0.02 to -0.01]

Brazil	Both	235.12 [113.52-483.84]	638.3 [309.39-1314.76]	171.48	26.2 [12.71-53.84]	26.64 [12.95-54.83]	0.05 [0.04 to 0.05]
Brunei Darussalam	Both	1.11 [0.56-2.35]	3.81 [1.92-7.91]	243.24	107.52 [54.52-223.8]	111.05 [55.79-230.76]	0.11 [0.1 to 0.13]
Bulgaria	Both	38.98 [18.9-82.05]	43.04 [21.16-90.94]	10.42	31.52 [15.26-66.87]	31.67 [15.69-66.64]	0.01 [0.01 to 0.02]
Burkina Faso	Both	23.64 [11.59-48.72]	52.03 [25.42-107.31]	120.09	52.06 [25.6-107.96]	53.06 [26.11-109.74]	0.07 [0.06 to 0.08]
Burundi	Both	12.55 [6.15-25.97]	25.41 [12.38-52.87]	102.47	52.72 [25.93-109.28]	51.32 [25.17-107.19]	-0.1 [-0.1 to -0.09]
Cabo Verde	Both	1.17 [0.58-2.44]	2.36 [1.16-4.91]	101.71	54.1 [26.3-112.76]	53.39 [26.29-111.65]	-0.05 [-0.06 to -0.03]
Cambodia	Both	1.7 [0.76-3.67]	4.63 [2.06-9.94]	172.35	3.9 [1.77-8.2]	3.96 [1.8-8.35]	0.06 [0.05 to 0.07]
Cameroon	Both	24.5 [11.98-50.52]	67.04 [32.59-138.7]	173.63	51.8 [25.64-107.6]	52.1 [25.79-108.9]	0.03 [0.02 to 0.03]
Canada	Both	472.01 [235.32-985.73]	942.23 [470.28-1979.45]	99.62	149.82 [74.64-311.37]	148.46 [74-310.61]	-0.03 [-0.04 to -0.03]
Central African Republic	Both	6.45 [3.18-13.46]	12.44 [6.14-25.88]	92.87	52.37 [26.16-108.76]	52.8 [26.2-109.12]	0.03 [0.03 to 0.03]
Chad	Both	14.86 [7.26-31.1]	30.57 [14.92-63.74]	105.72	52.26 [25.49-108.79]	50.84 [24.93-106.09]	-0.11 [-0.11 to -0.1]
Chile	Both	106 [52.44-222.64]	249.66 [124.32-521.11]	135.53	104.35 [51.71-218.24]	103.87 [51.52-215.21]	-0.02 [-0.02 to -0.01]
China	Both	2222.54 [1081.57-4697.46]	6230.67 [3040.33-12983.9]	180.34	26.08 [12.91-54.45]	30.12 [14.75-62.79]	-0.14 [-0.5 to 0.22]
Colombia	Both	48.74 [23.36-103]	147.76 [71.05-311.44]	203.16	27.23 [13.19-58.02]	27.74 [13.35-58.42]	0.08 [0.07 to 0.08]
Comoros	Both	1.18 [0.58-2.43]	2.67 [1.31-5.54]	126.27	52.13 [25.63-107.66]	52.87 [26.04-109.48]	0.05 [0.04 to 0.06]
Congo	Both	5.85 [2.87-12.2]	15.12 [7.37-30.95]	158.46	52.95 [26.07-110.86]	52.06 [25.73-107.7]	-0.06 [-0.07 to -0.06]
Costa Rica	Both	4.77 [2.3-9.96]	14.38 [6.97-30.43]	201.47	27.27 [13.23-56.86]	27.58 [13.37-58.23]	0.04 [0.04 to 0.04]
Cote d'Ivoire	Both	22.49 [10.92-46.62]	60.5 [29.4-125.48]	169.01	50.45 [24.83-104.57]	51.27 [25.12-106.08]	0.04 [0.03 to 0.06]
Croatia	Both	22.13 [10.62-46.08]	25.57 [12.71-53.5]	15.54	34.3 [16.55-70.93]	31.42 [15.63-66.05]	-0.34 [-0.42 to -0.25]
Cuba	Both	24.37 [11.92-51.16]	45.52 [22.24-94.77]	86.79	23.97 [11.72-50.34]	24.21 [11.77-50.45]	0.03 [0.03 to 0.03]
Cyprus	Both	9.61 [4.75-20.26]	22.15 [10.98-47.13]	130.49	117.54 [58.2-247.37]	117.57 [58.46-247.33]	0.5 [0.34 to 0.66]
Czechia	Both	42.42 [20.6-89.41]	61.05 [30.21-127.17]	43.92	31.5 [15.36-66.87]	31.24 [15.41-66.64]	-0.03 [-0.04 to -0.03]
Democratic People's Republic of Korea	Both	45.18 [21.89-95.44]	88.77 [43.26-186.19]	96.48	27.29 [13.25-57.44]	27.27 [13.29-57.28]	0 [0 to 0]
Democratic Republic of the Congo	Both	87.51 [43.09-180.45]	203.02 [100.21-426.58]	132	51.84 [25.55-106.35]	52.72 [26.18-110.37]	0.06 [0.04 to 0.07]
Denmark	Both	98.12 [49.36-206.1]	120.92 [59.53-257.08]	23.24	131.62 [66.17-275.08]	117.35 [57.9-246.68]	-0.06 [-0.16 to 0.04]
Djibouti	Both	0.82 [0.4-1.69]	3.48 [1.68-7.16]	324.39	51.91 [25.55-107.28]	51.14 [25.09-106.37]	-0.06 [-0.06 to -0.05]
Dominica	Both	0.17 [0.08-0.35]	0.21 [0.1-0.44]	23.53	25.24 [12.37-52.64]	23.8 [11.62-50.07]	-0.24 [-0.25 to -0.22]
Dominican Republic	Both	9.06 [4.44-18.84]	22.72 [11.15-46.56]	150.77	24.1 [11.89-49.78]	24.2 [11.86-49.43]	0 [-0.01 to 0]

Ecuador	Both	27.18 [13.45-57.54]	89.56 [44.84-185.32]	229.51	49.99 [24.49-105.18]	58.25 [29.14-120.27]	0.72 [0.58 to 0.85]
Egypt	Both	88.21 [42.87-186.1]	190.25 [92.42-400.66]	115.68	29.11 [14.36-61.08]	27.9 [13.71-59.19]	-0.15 [-0.15 to -0.15]
El Salvador	Both	8.16 [3.9-17.23]	16.42 [8.01-34.71]	101.23	27.54 [13.18-58.03]	27.96 [13.6-59.22]	0.06 [0.06 to 0.06]
Equatorial Guinea	Both	1.08 [0.54-2.25]	2.79 [1.36-5.83]	158.33	52.5 [26.04-109.36]	53.39 [26.44-111.39]	0.05 [0.04 to 0.07]
Eritrea	Both	5.77 [2.83-11.91]	15.65 [7.67-32.66]	171.23	53.68 [26.52-111.76]	53.44 [26.59-110.27]	-0.01 [-0.01 to 0]
Estonia	Both	14.72 [7.23-30.8]	16.99 [8.38-35.81]	15.42	72.33 [35.79-150.17]	71.17 [34.94-148.99]	-0.04 [-0.05 to -0.04]
Eswatini	Both	2.05 [1.02-4.25]	3.96 [1.99-8.18]	93.17	64.42 [32.21-133.37]	64.17 [32.17-132.73]	-0.01 [-0.02 to -0.01]
Ethiopia	Both	111.29 [55.2-233.19]	236.78 [117.39-497.34]	112.76	53.72 [26.66-111.79]	54.18 [26.84-113.45]	0.05 [0.03 to 0.07]
Fiji	Both	0.32 [0.15-0.69]	0.67 [0.31-1.43]	109.38	8.87 [4.25-18.71]	8.93 [4.27-18.88]	0.01 [0 to 0.02]
Finland	Both	45.61 [22.79-95.73]	68.02 [33.68-141.28]	49.13	66.13 [33-138.53]	62.76 [30.89-130.99]	-0.19 [-0.2 to -0.18]
France	Both	628.88 [312.08-1332.56]	959.18 [477.35-2013.12]	52.52	80.4 [39.99-168.03]	79.81 [39.96-168.54]	-0.01 [-0.01 to 0]
Gabon	Both	3 [1.49-6.25]	5.89 [2.88-12.23]	96.33	53.04 [26.36-111.4]	52.43 [26.13-108.76]	-0.05 [-0.05 to -0.04]
Gambia	Both	1.91 [0.93-3.98]	5.21 [2.58-10.72]	172.77	51.29 [25.34-107.47]	52.04 [25.9-106.96]	0.06 [0.05 to 0.06]
Germany	Both	688.58 [340.18-1445.93]	915 [458.59-1889.13]	32.88	56.4 [27.68-118.26]	53.64 [27.05-110.65]	-0.18 [-0.19 to -0.17]
Ghana	Both	34.82 [17.07-73.04]	92.08 [44.9-192.72]	164.45	52.31 [25.89-108.88]	53.38 [26.43-111.01]	0.07 [0.06 to 0.08]
Greece	Both	50.72 [25-104.4]	71.1 [35.61-146.07]	40.18	33.97 [16.89-69.76]	33.82 [16.8-69.61]	0.85 [0.49 to 1.2]
Greenland	Both	0.53 [0.27-1.11]	1.06 [0.52-2.22]	100	142.97 [71.16-296.24]	141.6 [70.74-293.47]	-0.03 [-0.05 to -0.01]
Grenada	Both	0.17 [0.08-0.35]	0.28 [0.13-0.58]	64.71	25 [12.26-51.74]	24.18 [11.85-50.41]	-0.1 [-0.12 to -0.08]
Guam	Both	0.07 [0.03-0.14]	0.17 [0.08-0.36]	142.86	8.89 [4.3-18.49]	8.95 [4.31-18.63]	0.03 [0.01 to 0.04]
Guatemala	Both	10.15 [4.94-21.25]	30.54 [14.95-63.27]	200.89	26.89 [13.03-55.91]	27.53 [13.43-57.55]	0.08 [0.08 to 0.09]
Guinea	Both	17.49 [8.51-36.44]	29.69 [14.52-61.79]	69.75	51.81 [25.28-107.09]	51.75 [25.58-107.68]	-0.01 [-0.02 to -0.01]
Guinea-Bissau	Both	2.19 [1.07-4.52]	4.09 [1.98-8.4]	86.76	52.09 [25.73-107.84]	52.93 [25.95-109.02]	0.05 [0.04 to 0.05]
Guyana	Both	0.92 [0.45-1.92]	1.55 [0.76-3.26]	68.48	23.92 [11.83-48.99]	24.07 [11.82-49.86]	0.02 [0.01 to 0.03]
Haiti	Both	7.89 [3.84-16.44]	17.32 [8.35-36.45]	119.52	24.12 [11.79-50]	24.07 [11.8-49.81]	0 [-0.01 to 0]
Honduras	Both	5.67 [2.71-11.98]	16.86 [8.13-35.05]	197.35	27.19 [13.01-57.96]	27.34 [13.24-56.93]	0.01 [0 to 0.02]
Hungary	Both	45.47 [22.37-95.61]	57.18 [27.87-121.65]	25.75	31.47 [15.45-66.29]	31.59 [15.5-66.84]	0.02 [0.02 to 0.03]
Iceland	Both	4.91 [2.47-10.17]	9.3 [4.61-19.22]	89.41	182.36 [91.56-374.93]	179.59 [88.5-373.71]	0 [-1.02 to 1.03]
India	Both	1180.67 [572.5-2496.49]	3024.43 [1495.86-6330.35]	156.16	25.73 [12.81-53.82]	26.41 [13.17-55.04]	0.1 [0.1 to 0.11]
Indonesia	Both	43.54 [19.62-92.92]	98.59 [45.2-210.13]	126.44	4.52 [2.12-9.39]	4.57 [2.09-9.42]	0.04 [0.04 to 0.04]
Iran (Islamic Republic of)	Both	121.23 [59.78-257.02]	353.65 [174.48-741.52]	191.72	44.69 [21.99-93.74]	46.17 [22.73-96.78]	0.14 [0 to 0.28]

Iraq	Both	22.81 [11.04-48.77]	70.7 [34.37-148.45]	209.95	28.89 [14.15-61.7]	29.07 [14.2-60.89]	0.02 [0.02 to 0.03]
Ireland	Both	33.3 [16.6-70.47]	60.5 [30.03-127.46]	81.68	85.08 [42.38-179.18]	84.5 [41.9-177.88]	-0.02 [-0.02 to -0.02]
Israel	Both	55.99 [27.67-118.49]	130.56 [64.6-274.97]	133.18	120.05 [59.55-254.15]	119.73 [59.09-252.38]	-0.54 [-0.79 to -0.29]
Italy	Both	619.36 [304.82-1289.48]	905.54 [453.32-1885.97]	46.21	72.37 [35.76-151.35]	71.95 [35.69-151.16]	0.22 [-0.02 to 0.46]
Jamaica	Both	4.21 [2.06-8.64]	7.24 [3.58-15.18]	71.97	24.51 [11.96-50.33]	24.07 [11.9-50.43]	-0.06 [-0.07 to -0.05]
Japan	Both	1353 [660.28-2811.59]	2727.85 [1346.35-5696.49]	101.61	79 [38.66-164.18]	93.39 [46.08-197.07]	1.69 [1.29 to 2.09]
Jordan	Both	4.11 [2-8.7]	20.53 [9.96-42.87]	399.51	28.79 [13.94-60.52]	28.44 [13.82-60.14]	-0.03 [-0.03 to -0.02]
Kazakhstan	Both	84.27 [41.61-177.76]	116.03 [57.29-245.98]	37.69	66.26 [32.57-139.28]	65.11 [31.91-137.41]	-0.06 [-0.06 to -0.05]
Kenya	Both	46.47 [23.19-97.32]	130.06 [64.55-271.11]	179.88	54.36 [27.03-113.22]	55.04 [27.34-115.33]	0.05 [0.05 to 0.06]
Kuwait	Both	1.86 [0.89-3.84]	8.7 [4.2-17.98]	367.74	26.48 [12.91-56.63]	26.92 [13.18-57.2]	0.09 [0.05 to 0.13]
Kyrgyzstan	Both	19.9 [9.8-41.68]	31.06 [15.25-65.31]	56.08	65.83 [32.38-137.57]	64.81 [31.95-136.44]	-0.04 [-0.05 to -0.03]
Lao People's Democratic Republic	Both	0.75 [0.34-1.59]	1.63 [0.72-3.44]	117.33	3.81 [1.73-7.95]	3.75 [1.7-7.88]	-0.03 [-0.04 to -0.02]
Latvia	Both	25.66 [12.75-53.75]	25.95 [12.82-54.58]	1.13	72.04 [35.84-150.28]	71.49 [35.1-150.86]	-0.02 [-0.02 to -0.01]
Lebanon	Both	6.83 [3.33-14.69]	15.47 [7.62-32.67]	126.5	29.13 [14.23-62.36]	29.97 [14.79-63.21]	0.1 [0.09 to 0.11]
Lesotho	Both	6.54 [3.24-13.52]	8.55 [4.26-17.77]	30.73	64.56 [32.13-132.54]	64.07 [32.18-132.47]	-0.02 [-0.03 to -0.02]
Liberia	Both	5.66 [2.77-11.77]	11.62 [5.72-23.61]	105.3	50.37 [24.82-105.01]	50.83 [25.12-104.06]	0.04 [0.03 to 0.04]
Libya	Both	5.44 [2.67-11.54]	15.74 [7.72-33.53]	189.34	28.33 [13.98-59.57]	28.65 [14.09-60.84]	0.06 [0.05 to 0.06]
Lithuania	Both	18.56 [9.24-39.24]	22.02 [11.02-45.6]	18.64	41.18 [20.51-87.34]	41.19 [20.4-85.6]	0.24 [0.09 to 0.39]
Luxembourg	Both	4.55 [2.26-9.6]	8.05 [4-17.07]	76.92	85.76 [42.65-178.95]	83.96 [41.47-177.29]	-0.07 [-0.07 to -0.07]
Madagascar	Both	27.4 [13.48-57.12]	63.45 [31.05-131.69]	131.57	51.5 [25.48-107.07]	52.27 [25.71-108.35]	0.06 [0.05 to 0.06]
Malawi	Both	21.1 [10.23-44.08]	40.45 [19.91-83.91]	91.71	52.21 [25.83-108.7]	52.99 [26.19-109.8]	0.07 [0.06 to 0.08]
Malaysia	Both	3.4 [1.52-7.33]	9.78 [4.33-20.79]	187.65	3.7 [1.68-7.68]	3.66 [1.64-7.68]	-0.05 [-0.07 to -0.04]
Maldives	Both	0.03 [0.01-0.06]	0.11 [0.05-0.24]	266.67	3.33 [1.49-6.98]	3.52 [1.61-7.46]	0.25 [0.21 to 0.28]
Mali	Both	22.29 [10.92-46.57]	46.99 [22.8-98.5]	110.81	51.7 [25.35-108.14]	51.5 [25.21-107]	-0.01 [-0.01 to 0]
Malta	Both	3.65 [1.81-7.73]	7.05 [3.49-14.77]	93.15	85.68 [42.52-181.42]	84.55 [41.78-177.66]	-0.04 [-0.05 to -0.04]
Marshall Islands	Both	0.01 [0.01-0.03]	0.03 [0.01-0.06]	200	8.92 [4.26-18.8]	8.65 [4.13-18.1]	-0.11 [-0.13 to -0.1]
Mauritania	Both	5.36 [2.63-11.2]	11.38 [5.59-23.81]	112.31	52.6 [25.98-108.98]	52.14 [25.85-109.12]	-0.03 [-0.04 to -0.02]
Mauritius	Both	0.28 [0.12-0.59]	0.66 [0.3-1.4]	135.71	3.86 [1.73-8.07]	3.8 [1.73-8.04]	-0.06 [-0.07 to -0.05]
Mexico	Both	310.69 [154.58-647.26]	1017.07 [506.01-2096.82]	227.36	70.54 [34.94-147.12]	84.34 [42.02-174.2]	0.86 [0.68 to 1.03]
Micronesia (Federated States of)	Both	0.04 [0.02-0.08]	0.06 [0.03-0.13]	50	8.92 [4.22-18.64]	8.99 [4.35-18.55]	0.03 [0.02 to 0.04]

Mongolia	Both	6.65 [3.27-14.02]	16.21 [7.98-33.7]	143.76	63.66 [31.35-133.97]	64.81 [31.65-135.56]	0.07 [0.06 to 0.08]
Montenegro	Both	2 [0.97-4.25]	3.05 [1.49-6.33]	52.5	31.69 [15.5-66.8]	31.54 [15.51-66.15]	-0.02 [-0.02 to -0.02]
Morocco	Both	40.82 [20.18-86.74]	95.3 [46.49-200.24]	133.46	29.28 [14.59-61.7]	29.08 [14.31-61.08]	-0.04 [-0.05 to -0.03]
Mozambique	Both	33.16 [16.15-68.96]	61.6 [30.18-127.98]	85.77	51.87 [25.46-107.3]	52.75 [26.44-108.4]	0.07 [0.06 to 0.07]
Myanmar	Both	8.47 [3.78-17.8]	17.98 [7.88-38.64]	112.28	3.8 [1.71-8.06]	3.9 [1.75-8.21]	0.1 [0.09 to 0.1]
Namibia	Both	4.7 [2.35-9.7]	9.58 [4.77-19.91]	103.83	63.89 [31.66-131.74]	64.17 [31.75-131.99]	0.02 [0.01 to 0.02]
Nepal	Both	23 [11.16-49.49]	55.84 [27.31-117.46]	142.78	24.09 [11.78-50.78]	24.78 [11.93-51.92]	0.09 [0.08 to 0.1]
Netherlands	Both	122.89 [62.74-248.82]	188.09 [94.14-400.61]	53.06	64.03 [32.49-130.19]	60.04 [29.79-127.79]	-0.37 [-0.59 to -0.15]
New Zealand	Both	42.66 [21.23-87.79]	82.15 [40.81-170.3]	92.57	112.55 [55.93-235.14]	112.58 [56.1-236.6]	0 [0 to 0]
Nicaragua	Both	4.26 [2.06-8.98]	12.62 [6.14-26.94]	196.24	27.51 [13.26-57.7]	27.72 [13.46-59.17]	0.03 [0.02 to 0.03]
Niger	Both	15.5 [7.56-32.24]	43.78 [21.52-91.53]	182.45	51.53 [25.48-107.54]	52.36 [26.02-109.38]	0.06 [0.06 to 0.07]
Nigeria	Both	242.52 [120.15-511.65]	520.75 [258.2-1091.99]	114.72	53.73 [26.61-112.09]	55.05 [27.36-115.11]	0.1 [0.07 to 0.13]
North Macedonia	Both	5.98 [2.89-12.57]	10.05 [4.93-21.35]	68.06	31.42 [15.36-65.66]	31.36 [15.38-66.77]	0 [0 to 0]
Northern Mariana Islands	Both	0.02 [0.01-0.03]	0.05 [0.02-0.1]	150	8.41 [4.04-17.45]	8.79 [4.2-18.6]	0.15 [0.13 to 0.17]
Norway	Both	74.64 [37.41-155.65]	105.16 [52.8-218.55]	40.89	122.01 [61.19-252.8]	119.7 [59.91-249.15]	0.13 [0.03 to 0.23]
Oman	Both	2 [0.97-4.23]	5.74 [2.77-12.1]	187	28.11 [14.04-58.8]	27.28 [13.2-57.78]	-0.14 [-0.16 to -0.11]
Pakistan	Both	146.58 [71.91-309.05]	295.74 [143.63-625.43]	101.76	25.68 [12.59-54.13]	26.2 [12.8-55.13]	0.07 [0.07 to 0.07]
Palestine	Both	2.63 [1.29-5.63]	7.43 [3.56-15.7]	182.51	30.11 [14.72-63.88]	29.26 [14.33-61.86]	-0.11 [-0.12 to -0.09]
Panama	Both	4.07 [1.98-8.48]	11.36 [5.53-23.89]	179.12	27.1 [13.17-56.66]	27.25 [13.24-57.43]	0.02 [0.02 to 0.02]
Papua New Guinea	Both	1.6 [0.75-3.34]	4.15 [1.94-8.77]	159.38	8.73 [4.2-18.22]	8.64 [4.2-17.89]	-0.03 [-0.04 to -0.03]
Paraguay	Both	5.4 [2.64-11.44]	13.6 [6.67-28.14]	151.85	24.21 [11.95-50.99]	24.16 [11.91-49.9]	-0.01 [-0.01 to -0.01]
Peru	Both	31.31 [15.25-65.88]	84.11 [41.78-174.9]	168.64	25.91 [12.83-54.45]	26.06 [12.95-54.36]	0.22 [0.09 to 0.34]
Philippines	Both	13.28 [5.99-28.31]	35.62 [16.19-75.45]	168.22	4.52 [2.09-9.37]	4.64 [2.15-9.51]	0.11 [0.1 to 0.11]
Poland	Both	146.77 [73.2-306.84]	227.98 [113.79-477.52]	55.33	33.97 [16.97-71.14]	34.13 [16.93-71.64]	0.02 [0.01 to 0.02]
Portugal	Both	126.84 [62.98-265.35]	195.5 [97.28-409.13]	54.13	93.12 [46.07-194.14]	92.43 [45.55-191.6]	0.17 [0.1 to 0.23]
Puerto Rico	Both	8.79 [4.3-18.35]	16.15 [7.95-33.53]	83.73	24.5 [11.98-51.19]	24.56 [11.98-51.54]	0.01 [0 to 0.01]
Qatar	Both	0.39 [0.19-0.82]	3.43 [1.62-7.07]	779.49	25.73 [12.6-53.71]	22.78 [11.21-47.38]	-0.45 [-0.46 to -0.43]
Republic of Korea	Both	451.52 [229.29-934.61]	1228.65 [612.73-2565.18]	172.11	143.48 [72.48-293.95]	135.2 [68.07-281.23]	0.44 [0.21 to 0.66]
Republic of Moldova	Both	31.61 [15.68-66.67]	40.56 [20.11-84.75]	28.31	71.01 [35.15-148.23]	70.96 [35.12-147.66]	-0.01 [-0.01 to 0]
Romania	Both	88.75 [42.97-186.69]	111.58 [55.05-238.17]	25.72	31.59 [15.42-67.15]	31.75 [15.54-67.4]	0.02 [0.02 to 0.03]

Russian Federation	Both	3520.51 [1779.42-7196.48]	4085.65 [2052.95-8520.28]	16.05	195.22 [98.83-399.42]	178 [89.5-370.48]	-1.11 [-1.42 to -0.8]
Rwanda	Both	15.7 [7.76-32.55]	34.46 [16.74-72.55]	119.49	52.85 [26.31-109.76]	53.54 [26.15-111.61]	0.06 [0.05 to 0.07]
Saint Lucia	Both	0.21 [0.1-0.44]	0.53 [0.26-1.08]	152.38	24.7 [12.19-51.38]	24.05 [11.77-49.19]	-0.09 [-0.09 to -0.08]
Saint Vincent and the Grenadines	Both	0.17 [0.09-0.36]	0.32 [0.16-0.67]	88.24	24.77 [12.22-51.59]	23.54 [11.47-48.86]	-0.17 [-0.18 to -0.17]
Samoa	Both	0.08 [0.04-0.16]	0.13 [0.06-0.27]	62.5	8.96 [4.28-18.81]	8.86 [4.22-18.46]	-0.04 [-0.05 to -0.04]
Sao Tome and Principe	Both	0.34 [0.17-0.71]	0.6 [0.29-1.25]	76.47	52.62 [25.81-109.16]	52.24 [25.75-108.66]	-0.02 [-0.03 to -0.02]
Saudi Arabia	Both	46.01 [22.91-93.46]	162.07 [81.04-326.56]	252.25	70.25 [35.42-145.38]	69.58 [35.23-144.03]	-0.71 [-0.98 to -0.44]
Senegal	Both	17.29 [8.45-35.93]	41.26 [20.27-86.42]	138.64	51.64 [25.49-107.5]	52.23 [25.59-109.1]	0.04 [0.03 to 0.04]
Serbia	Both	36.69 [17.86-78.02]	47.37 [23.25-101.1]	29.11	31.55 [15.49-66.6]	31.38 [15.26-66.5]	-0.01 [-0.01 to -0.01]
Seychelles	Both	0.02 [0.01-0.05]	0.04 [0.02-0.09]	100	3.91 [1.75-8.18]	3.76 [1.73-7.85]	-0.15 [-0.17 to -0.13]
Sierra Leone	Both	9.98 [4.92-20.6]	19.64 [9.53-40.57]	96.79	51.46 [25.5-107.12]	51.72 [25.41-107.09]	0.02 [0.01 to 0.03]
Singapore	Both	26.44 [13.26-55.04]	87.98 [43.87-184.93]	232.75	113.41 [56.64-235.67]	108.11 [53.74-226.75]	0.14 [0.04 to 0.23]
Slovakia	Both	18.61 [9.2-39.57]	28.38 [13.89-59.36]	52.5	31.6 [15.58-66.9]	31.63 [15.38-66.77]	0 [0 to 0.01]
Slovenia	Both	7.69 [3.76-16.31]	12.57 [6.17-26.25]	63.46	31.53 [15.49-66.62]	31.43 [15.42-65.34]	-0.02 [-0.03 to -0.02]
Solomon Islands	Both	0.11 [0.05-0.24]	0.27 [0.13-0.56]	145.45	8.55 [4.07-17.93]	8.76 [4.22-18.03]	0.08 [0.08 to 0.09]
Somalia	Both	14.64 [7.11-30.37]	38.06 [18.67-79.15]	159.97	52.34 [25.99-109.01]	53.43 [26.58-111.58]	0.08 [0.07 to 0.09]
South Africa	Both	142.96 [70.83-298.74]	309.78 [154.39-643.76]	116.69	66.72 [33.34-138.51]	66.45 [33.21-137.17]	-0.01 [-0.02 to -0.01]
South Sudan	Both	12.22 [5.97-25.12]	21.67 [10.49-44.18]	77.33	49.59 [24.21-101.98]	50.3 [24.83-103.29]	0.05 [0.04 to 0.06]
Spain	Both	425.82 [212.66-896.15]	918.21 [369.86-2242.03]	115.63	80.83 [40.11-168.71]	106.59 [42.69-258.3]	1.88 [1.46 to 2.29]
Sri Lanka	Both	3.93 [1.7-8.18]	9.73 [4.31-20.95]	147.58	3.66 [1.61-7.54]	3.85 [1.74-8.23]	0.18 [0.16 to 0.19]
Sudan	Both	26.84 [13.03-56.77]	55.27 [26.89-115.97]	105.92	28.52 [14.08-60.57]	28.05 [13.96-58.8]	-0.07 [-0.08 to -0.06]
Suriname	Both	0.64 [0.31-1.32]	1.49 [0.73-3.1]	132.81	24.2 [11.82-50.01]	24.28 [11.8-49.85]	0.01 [0.01 to 0.01]
Sweden	Both	65.68 [32.45-137.46]	87.37 [42.86-181.4]	33.02	47.54 [23.29-99.49]	46.94 [22.94-97.96]	-0.26 [-0.67 to 0.15]
Switzerland	Both	82.99 [41.12-173.46]	133.01 [66.09-280.32]	60.27	85.17 [42.08-178.31]	84.61 [41.98-176.3]	-0.03 [-0.03 to -0.03]
Syrian Arab Republic	Both	15.51 [7.61-33.12]	38.22 [18.65-80.45]	146.42	28.54 [14.06-60.35]	28.49 [13.98-60.85]	0 [-0.02 to 0.01]
Tajikistan	Both	18.09 [8.81-37.66]	34.66 [16.94-72.99]	91.6	64.27 [31.41-134.27]	62.85 [30.67-132.68]	-0.08 [-0.1 to -0.06]
Thailand	Both	13.28 [5.9-28.31]	39.7 [17.57-84.74]	198.95	3.76 [1.7-7.94]	3.84 [1.7-8.17]	0.12 [0.1 to 0.14]
Timor-Leste	Both	0.1 [0.05-0.22]	0.29 [0.13-0.61]	190	3.64 [1.65-7.6]	3.65 [1.65-7.5]	0.06 [0.02 to 0.09]
Togo	Both	6.98 [3.41-14.52]	21.45 [10.41-44.45]	207.31	52.48 [25.9-109.68]	53.63 [26.56-112.04]	0.08 [0.07 to 0.09]
Tonga	Both	0.05 [0.02-0.1]	0.07 [0.03-0.15]	40	9.01 [4.28-18.54]	9.03 [4.37-18.99]	0.01 [0.01 to 0.02]

Trinidad and Tobago	Both	2.02 [0.98-4.15]	4.49 [2.23-9.27]	122.28	24.26 [11.83-49.45]	24.03 [11.99-49.36]	-0.02 [-0.03 to -0.02]
Tunisia	Both	14.85 [7.19-31.42]	38 [18.58-81.41]	155.89	28.92 [14-61.11]	29.34 [14.46-62.68]	0.05 [0.04 to 0.06]
Turkey	Both	29.29 [13.97-61.66]	72.72 [34.39-156.02]	148.28	8.14 [3.89-17.21]	8.15 [3.88-17.3]	-0.13 [-0.29 to 0.04]
Turkmenistan	Both	8.67 [4.23-18.05]	20.08 [9.8-42.5]	131.6	45.09 [22.19-94.11]	49.05 [24.19-102.49]	0.33 [0.23 to 0.42]
Uganda	Both	34.78 [17.03-72.36]	80.25 [38.96-166.34]	130.74	51.72 [25.63-107.46]	53.14 [25.79-110.09]	0.1 [0.09 to 0.11]
Ukraine	Both	409.21 [198.68-863.45]	431.09 [213.58-907.19]	5.35	57.7 [28.15-122.06]	57.95 [28.58-121.6]	0.28 [0.11 to 0.44]
United Arab Emirates	Both	1.45 [0.7-2.98]	14.48 [6.74-29.51]	898.62	25.8 [12.65-54.58]	23.57 [11.55-48.82]	-0.32 [-0.34 to -0.3]
United Kingdom	Both	662.83 [326.59-1388.88]	925.13 [457.84-1923.4]	39.57	78.54 [38.47-164.91]	81.32 [40.18-170.64]	-0.03 [-0.38 to 0.32]
United Republic of Tanzania	Both	58.74 [28.5-120.95]	137.48 [66.82-282.15]	134.05	51.9 [25.33-106.31]	52.3 [25.62-108.12]	0.03 [0.02 to 0.03]
United States of America	Both	4868.46 [2494.56-10176.54]	9300.81 [4667.04-19649.24]	91.04	163.53 [83.73-342.46]	176.31 [89.12-366.47]	0.27 [0.03 to 0.52]
United States Virgin Islands	Both	0.22 [0.11-0.46]	0.44 [0.22-0.92]	100	24.66 [12.12-51.52]	24.72 [11.98-50.98]	0 [0 to 0.01]
Uruguay	Both	39.94 [19.57-83.72]	52.71 [26.01-110.46]	31.97	105.55 [51.95-220.29]	105.68 [52.12-223.91]	0.01 [0.01 to 0.02]
Uzbekistan	Both	73.71 [36.18-154.56]	146.61 [72.6-307.34]	98.9	65.17 [31.79-136.78]	64.17 [31.44-134.29]	-0.04 [-0.05 to -0.04]
Vanuatu	Both	0.06 [0.03-0.12]	0.15 [0.07-0.31]	150	8.57 [4.12-17.84]	8.69 [4.17-18.22]	0.07 [0.05 to 0.08]
Venezuela (Bolivarian Republic of)	Both	10.87 [5.21-23.61]	33.42 [15.96-71.69]	207.45	11.27 [5.41-24.4]	11.32 [5.44-24.07]	-0.02 [-0.04 to 0]
VietNam	Both	15.54 [6.89-32.29]	36.84 [16.73-76.75]	137.07	4 [1.79-8.33]	3.96 [1.83-8.18]	-0.04 [-0.04 to -0.03]
Yemen	Both	14.76 [7.19-31.4]	40.54 [19.72-85.46]	174.66	29.55 [14.29-61.83]	29.12 [14.03-61.72]	-0.04 [-0.05 to -0.02]
Zambia	Both	15.55 [7.59-31.81]	37.79 [18.36-78.63]	143.02	51.13 [25.05-105.41]	52.13 [25.93-109.08]	0.09 [0.08 to 0.1]
Zimbabwe	Both	27.64 [13.64-57.13]	48.62 [24.2-101.6]	75.9	63.83 [31.25-130.67]	64.33 [32.01-132.59]	0.04 [0.04 to 0.04]
Afghanistan	Male	6.59 [3.22-14.02]	11.87 [5.63-24.32]	80.12	17.58 [8.62-37.16]	17.37 [8.43-36.42]	-0.03 [-0.04 to -0.03]
Albania	Male	3.04 [1.48-6.46]	5.82 [2.88-12.46]	91.45	28.8 [14.13-61.44]	28.94 [14.15-61.48]	0.02 [0.02 to 0.03]
Algeria	Male	11.01 [5.33-23.28]	32.05 [15.41-67.11]	191.1	17.89 [8.66-37.3]	17.85 [8.65-37.31]	-0.01 [-0.01 to 0]
American Samoa	Male	0.01 [0-0.02]	0.01 [0.01-0.03]	0	6.09 [2.79-12.63]	6.06 [2.77-12.52]	-0.01 [-0.02 to 0]
Andorra	Male	0.52 [0.26-1.1]	1.27 [0.63-2.67]	144.23	176.2 [88.63-371.92]	176.07 [88.18-371.89]	1.18 [0.79 to 1.57]
Angola	Male	8.72 [4.31-17.87]	23.43 [11.5-48.39]	168.69	40.46 [19.86-83.09]	40.53 [19.88-82.98]	0.01 [0 to 0.01]
Antigua and Barbuda	Male	0.04 [0.02-0.07]	0.08 [0.04-0.17]	100	15.88 [7.68-33.41]	15.81 [7.55-33.04]	-0.02 [-0.02 to -0.01]
Argentina	Male	73.62 [35.53-157.14]	119.33 [57.96-254.48]	62.09	50.52 [24.47-107.75]	50.51 [24.5-107.54]	0 [0 to 0]
Armenia	Male	4.91 [2.39-10.33]	7.21 [3.5-15.21]	46.84	40.05 [19.7-83.48]	40.3 [19.7-84.5]	0.02 [0.02 to 0.03]

Australia	Male	44.3 [21.65-94.08]	91.97 [44.92-193.44]	107.61	49.93 [24.44-105.85]	49.93 [24.2-104.95]	0 [-0.01 to 0]
Austria	Male	70.94 [35.62-148.54]	115.91 [57.75-248.42]	63.39	156.72 [78.56-327.29]	157.07 [78.73-331.61]	1.15 [0.78 to 1.53]
Azerbaijan	Male	7.69 [3.77-16.14]	16.37 [8.03-34.69]	112.87	35.57 [17.45-74.75]	35.45 [17.53-75.16]	0.35 [0.12 to 0.59]
Bahamas	Male	0.11 [0.05-0.24]	0.29 [0.14-0.63]	163.64	15.9 [7.59-33.49]	15.85 [7.68-33.62]	-0.02 [-0.02 to -0.01]
Bahrain	Male	0.22 [0.1-0.47]	1.56 [0.74-3.27]	609.09	17.73 [8.6-37.55]	17.6 [8.66-36.95]	-0.03 [-0.04 to -0.02]
Bangladesh	Male	43.16 [20.94-92.12]	113.73 [54.96-243.67]	163.51	16.67 [8.27-35.15]	16.73 [8.15-35.75]	0.02 [0.02 to 0.03]
Barbados	Male	0.18 [0.09-0.38]	0.35 [0.17-0.72]	94.44	15.96 [7.6-33.79]	15.87 [7.48-33.1]	-0.02 [-0.02 to -0.01]
Belarus	Male	23.82 [11.71-50.28]	29.44 [14.49-62.17]	23.59	47.77 [23.59-99.57]	47.92 [23.59-100.14]	0.01 [0 to 0.02]
Belgium	Male	75.3 [37.43-156.31]	110.37 [54.86-228.56]	46.57	117.32 [58.46-243.85]	117.18 [58.44-244.81]	0.72 [0.48 to 0.96]
Belize	Male	0.07 [0.04-0.16]	0.23 [0.11-0.48]	228.57	15.96 [7.49-33.79]	15.86 [7.63-33.01]	-0.01 [-0.02 to -0.01]
Benin	Male	3.95 [1.93-8.1]	10.26 [5.03-20.79]	159.75	40.58 [19.94-83.73]	40.86 [20.15-83.08]	0.02 [0.02 to 0.02]
Bermuda	Male	0.04 [0.02-0.1]	0.09 [0.04-0.19]	125	15.94 [7.71-33.97]	15.85 [7.64-33.36]	-0.01 [-0.02 to -0.01]
Bhutan	Male	0.21 [0.1-0.45]	0.49 [0.23-1.05]	133.33	16.73 [8.14-34.83]	16.69 [7.98-34.82]	0 [0 to 0.01]
Bolivia(Plurinational State of)	Male	2.96 [1.42-6.23]	8.35 [4.01-17.23]	182.09	19.19 [9.25-39.78]	19.23 [9.3-39.55]	0.01 [0.01 to 0.02]
Bosnia and Herzegovina	Male	5.47 [2.66-11.35]	7.54 [3.7-15.82]	37.84	28.72 [13.88-59.41]	28.61 [14.08-59.62]	-0.02 [-0.03 to -0.02]
Botswana	Male	1.52 [0.75-3.18]	3.83 [1.89-7.97]	151.97	54.81 [27.04-115.23]	54.26 [26.6-113.18]	-0.02 [-0.03 to -0.02]
Brazil	Male	73.12 [35.35-151.32]	188.65 [90.94-388.45]	158	17.2 [8.35-34.97]	17.32 [8.37-35.45]	0.01 [0.01 to 0.02]
Brunei Darussalam	Male	0.15 [0.07-0.32]	0.47 [0.23-0.98]	213.33	29.42 [14.46-61.06]	29.5 [14.68-61.24]	0.02 [0.01 to 0.02]
Bulgaria	Male	16.49 [8.16-34.22]	16.98 [8.34-35.52]	2.97	28.65 [14.12-59.17]	28.77 [14.26-59.83]	0.01 [0.01 to 0.01]
Burkina Faso	Male	8.73 [4.26-17.73]	18.47 [8.92-37.53]	111.57	40.45 [19.85-82.09]	40.85 [19.89-82.92]	0.04 [0.03 to 0.04]
Burundi	Male	4.43 [2.19-9.04]	10.68 [5.23-21.82]	141.08	40.54 [19.86-83.07]	40.43 [19.74-82.93]	0 [-0.01 to 0]
Cabo Verde	Male	0.37 [0.18-0.77]	0.83 [0.41-1.72]	124.32	41.05 [20.13-83.83]	40.92 [20.01-84.16]	-0.01 [-0.02 to -0.01]
Cambodia	Male	0.37 [0.16-0.83]	0.99 [0.41-2.18]	167.57	1.97 [0.84-4.37]	1.98 [0.83-4.2]	0.01 [0 to 0.01]
Cameroon	Male	9.46 [4.62-19.39]	25.98 [12.86-52.4]	174.63	40.44 [19.89-83.37]	40.59 [19.96-83.31]	0.02 [0.01 to 0.03]
Canada	Male	148.8 [73.02-310.85]	310.34 [154.73-649.01]	108.56	102.66 [50.44-213.99]	102.59 [51.25-214.68]	0 [-0.01 to 0]
Central African Republic	Male	2.34 [1.16-4.78]	4.58 [2.25-9.41]	95.73	40.1 [19.63-81.88]	40.25 [19.81-82.55]	0.02 [0.01 to 0.02]
Chad	Male	5.58 [2.73-11.56]	13.1 [6.42-27.03]	134.77	40.7 [20.01-84.69]	40.64 [20.14-84.09]	0 [-0.01 to 0]
Chile	Male	23.28 [11.26-49.35]	55.44 [26.95-116.05]	138.14	50.41 [24.37-106.5]	50.31 [24.44-105.65]	0 [-0.01 to 0]
China	Male	1062 [517.1-2258.51]	2995.58 [1446.69-6308.99]	182.07	25.56 [12.64-53.7]	29.95 [14.62-63.73]	-0.93 [-1.57 to -0.28]
Colombia	Male	18.26 [8.89-38.43]	50.77 [24.87-106.22]	178.04	20.78 [10.13-43.17]	20.89 [10.19-43.67]	0.02 [0.02 to 0.03]

Comoros	Male	0.45 [0.22-0.93]	0.97 [0.48-1.96]	115.56	40.61 [19.8-83.34]	40.74 [19.94-82.65]	0.01 [0.01 to 0.02]
Congo	Male	2.03 [1-4.14]	5.97 [2.92-12.09]	194.09	40.45 [19.97-82.99]	40.67 [19.86-83.45]	0.02 [0.02 to 0.02]
Costa Rica	Male	1.79 [0.86-3.67]	5.02 [2.46-10.66]	180.45	20.86 [10.12-43.05]	20.8 [10.25-43.97]	-0.01 [-0.02 to -0.01]
Cote d'Ivoire	Male	9.83 [4.8-20.2]	25.98 [12.63-53.63]	164.29	40.3 [19.7-82.9]	40.67 [19.91-83.52]	0.02 [0.02 to 0.03]
Croatia	Male	8.79 [4.17-18.35]	10.18 [5.11-21.46]	15.81	32.14 [15.35-67.02]	28.57 [14.2-59.75]	-0.45 [-0.58 to -0.32]
Cuba	Male	7.96 [3.75-16.74]	13.98 [6.73-29.98]	75.63	15.87 [7.51-33.38]	15.78 [7.52-33.65]	-0.02 [-0.02 to -0.01]
Cyprus	Male	5.29 [2.63-11.2]	12.34 [6.15-26.05]	133.27	137.07 [68.15-289.71]	136.99 [68.54-289.4]	0.85 [0.57 to 1.13]
Czechia	Male	16.21 [7.96-34.09]	24.95 [12.25-52.67]	53.92	28.49 [14.03-59.96]	28.41 [13.98-59.8]	-0.02 [-0.02 to -0.01]
Democratic People's Republic of Korea	Male	17.25 [8.3-35.91]	36.76 [17.96-77.92]	113.1	27.15 [13.41-56.6]	27.17 [13.29-56.78]	0 [0 to 0]
Democratic Republic of the Congo	Male	31.48 [15.31-64.36]	74.47 [36.76-152.79]	136.56	40.22 [19.79-81.99]	40.41 [19.98-84.62]	0.03 [0.02 to 0.03]
Denmark	Male	37.35 [18.24-77.5]	48.27 [23.48-102.29]	29.24	111.57 [54.63-231.31]	98.22 [47.87-207.43]	0.29 [0.08 to 0.5]
Djibouti	Male	0.35 [0.17-0.7]	1.55 [0.75-3.17]	342.86	40.87 [19.89-83.89]	40.83 [19.85-82.97]	0 [-0.01 to 0]
Dominica	Male	0.04 [0.02-0.09]	0.07 [0.03-0.15]	75	15.87 [7.64-33.07]	15.7 [7.52-32.99]	-0.04 [-0.04 to -0.03]
Dominican Republic	Male	2.96 [1.42-6.34]	7.24 [3.44-15.33]	144.59	15.89 [7.61-34.11]	15.87 [7.55-33.47]	-0.01 [-0.01 to 0]
Ecuador	Male	6.15 [2.96-13.03]	18.46 [9.01-37.7]	200.16	22.94 [11.12-48.84]	25 [12.18-51.27]	0.42 [0.33 to 0.52]
Egypt	Male	27.63 [13.23-58.17]	65.16 [31.44-136.35]	135.83	17.99 [8.74-38.03]	17.86 [8.7-37.42]	0 [-0.01 to 0]
El Salvador	Male	2.87 [1.4-6.09]	5.12 [2.52-10.51]	78.4	20.75 [10.13-43.76]	20.65 [10.14-42.55]	-0.02 [-0.03 to -0.02]
Equatorial Guinea	Male	0.37 [0.18-0.77]	0.93 [0.45-1.92]	151.35	40.08 [19.65-81.83]	40.52 [19.94-83.36]	0.04 [0.04 to 0.05]
Eritrea	Male	1.96 [0.96-4.01]	5.47 [2.69-11.14]	179.08	40.33 [19.83-82.86]	40.21 [20.01-81.12]	0 [-0.01 to 0]
Estonia	Male	3.68 [1.82-7.68]	4.52 [2.22-9.44]	22.83	47.53 [23.62-98.37]	48.02 [23.6-99.57]	0.04 [0.04 to 0.05]
Eswatini	Male	0.81 [0.4-1.69]	1.44 [0.73-3.01]	77.78	54.86 [27.26-115.09]	54 [27.22-114.24]	-0.06 [-0.07 to -0.05]
Ethiopia	Male	46.7 [23.07-96.65]	96.86 [47.68-200.42]	107.41	42.66 [21.2-88.54]	43.01 [21.24-90.49]	0.04 [0.03 to 0.04]
Fiji	Male	0.11 [0.05-0.23]	0.21 [0.1-0.46]	90.91	6.09 [2.77-12.61]	6.04 [2.77-12.79]	-0.03 [-0.04 to -0.02]
Finland	Male	7.03 [3.5-14.92]	12.48 [6.19-26.24]	77.52	24.76 [12.31-52.38]	24.86 [12.21-52.76]	0.01 [0 to 0.02]
France	Male	191.06 [94.17-398.59]	306.61 [152.61-644.78]	60.48	56.22 [27.68-117.2]	56.35 [28.15-118.24]	0.06 [0.04 to 0.08]
Gabon	Male	1.05 [0.52-2.17]	2.21 [1.08-4.57]	110.48	40.54 [20.39-84.15]	40.51 [19.95-83]	0 [-0.01 to 0]
Gambia	Male	0.84 [0.41-1.71]	2.04 [1-4.2]	142.86	40.9 [20.24-84.16]	40.91 [20.31-83.6]	-0.01 [-0.01 to -0.01]
Germany	Male	137.73 [69.15-288.58]	219.97 [107.94-461.95]	59.71	27.9 [13.9-58.16]	27.85 [13.69-58.78]	-0.01 [-0.01 to 0]

Ghana	Male	13.38 [6.54-27.88]	32.09 [15.67-65.97]	139.84	40.8 [20.01-84.02]	40.83 [20.16-84.15]	0 [0 to 0]
Greece	Male	13.78 [6.71-29.5]	19.36 [9.53-40.69]	40.49	19.94 [9.77-42.91]	19.91 [9.78-42.05]	1.31 [0.77 to 1.86]
Greenland	Male	0.2 [0.1-0.41]	0.41 [0.2-0.86]	105	100.81 [50.06-210.87]	101.47 [50.42-210.61]	0.03 [0.02 to 0.03]
Grenada	Male	0.05 [0.02-0.1]	0.09 [0.04-0.19]	80	15.84 [7.57-33.02]	15.72 [7.59-33.05]	-0.03 [-0.03 to -0.02]
Guam	Male	0.02 [0.01-0.05]	0.06 [0.03-0.12]	200	6.19 [2.82-12.92]	6.17 [2.88-12.72]	-0.01 [-0.02 to 0]
Guatemala	Male	3.9 [1.89-8.19]	10.33 [5.1-21.47]	164.87	20.72 [10.09-43.1]	20.63 [10.21-43.14]	-0.02 [-0.02 to -0.01]
Guinea	Male	6.89 [3.38-14]	11.77 [5.67-24.41]	70.83	40.8 [20.2-83.19]	40.81 [20.04-85.09]	0 [0 to 0]
Guinea-Bissau	Male	0.82 [0.4-1.7]	1.48 [0.72-3.02]	80.49	40.52 [19.88-83.26]	40.76 [19.92-83.23]	0.02 [0.01 to 0.02]
Guyana	Male	0.29 [0.14-0.62]	0.48 [0.23-1.01]	65.52	15.65 [7.51-33.41]	15.58 [7.37-32.89]	-0.01 [-0.01 to 0]
Haiti	Male	2.53 [1.21-5.44]	5.34 [2.52-11.25]	111.07	15.73 [7.63-33.59]	15.71 [7.5-32.99]	-0.01 [-0.01 to 0]
Honduras	Male	2.14 [1.04-4.51]	6.06 [2.95-12.57]	183.18	20.81 [10.22-43.47]	20.77 [10.08-43.15]	-0.01 [-0.01 to 0]
Hungary	Male	17.36 [8.51-36.66]	21.57 [10.54-45.66]	24.25	28.47 [13.99-59.39]	28.59 [14.06-61.01]	0.03 [0.02 to 0.03]
Iceland	Male	2.18 [1.1-4.53]	4.05 [1.98-8.52]	85.78	171.06 [85.66-355.24]	160.01 [78.32-335.5]	-0.26 [-1.38 to 0.88]
India	Male	430.52 [207.21-903.67]	1014.07 [488.97-2113.32]	135.55	17.92 [8.78-37.19]	18.01 [8.81-37.14]	0.02 [0.02 to 0.02]
Indonesia	Male	12.15 [5.06-26.66]	27.42 [11.39-59.78]	125.68	2.58 [1.12-5.64]	2.59 [1.11-5.58]	0.01 [0.01 to 0.02]
Iran (Islamic Republic of)	Male	33.35 [16.12-71.15]	91.08 [44.08-191.41]	173.1	23.16 [11.32-48.45]	23.66 [11.5-49.32]	0.04 [-0.06 to 0.13]
Iraq	Male	6.94 [3.4-14.62]	21.77 [10.58-45.14]	213.69	17.56 [8.57-37.35]	17.68 [8.72-36.08]	0.02 [0.01 to 0.03]
Ireland	Male	12.4 [6.19-26.32]	23.47 [11.64-49.57]	89.27	68.24 [34.07-144.91]	68.1 [33.97-143.32]	0 [0 to 0]
Israel	Male	22.08 [10.9-46.18]	52.27 [25.54-110.07]	136.73	102.53 [50.75-215.34]	102.37 [50.15-214.7]	-0.67 [-0.97 to -0.36]
Italy	Male	202.97 [99.9-426.89]	292.73 [143.06-614.72]	44.22	54.07 [26.46-114.96]	51.38 [25.27-109.94]	0.13 [-0.16 to 0.42]
Jamaica	Male	1.28 [0.61-2.72]	2.28 [1.1-4.72]	78.12	15.98 [7.57-34.16]	15.8 [7.55-32.62]	-0.03 [-0.04 to -0.03]
Japan	Male	222.22 [107.85-474.19]	411.14 [203.39-868.5]	85.01	29.37 [14.31-62.08]	29.52 [14.4-62.16]	-0.09 [-0.14 to -0.05]
Jordan	Male	1.34 [0.65-2.82]	6.99 [3.37-14.51]	421.64	17.87 [8.83-37.84]	17.86 [8.79-37.54]	0 [-0.01 to 0]
Kazakhstan	Male	20.42 [9.99-42.95]	30.42 [14.91-63.28]	48.97	40.13 [19.63-83.95]	40.11 [19.77-84.24]	-0.01 [-0.01 to 0]
Kenya	Male	18.08 [8.8-37.33]	49.79 [24.32-103.56]	175.39	42.7 [21.18-89.11]	42.77 [21.24-89.51]	0.02 [0.01 to 0.03]
Kuwait	Male	0.84 [0.39-1.76]	3.41 [1.6-7.21]	305.95	17.88 [8.72-37.88]	17.78 [8.56-37.42]	-0.01 [-0.02 to -0.01]
Kyrgyzstan	Male	4.99 [2.41-10.53]	8.69 [4.24-18.2]	74.15	40.27 [19.66-84.91]	40.58 [19.83-85.45]	0.02 [0.02 to 0.03]
Lao People's Democratic Republic	Male	0.19 [0.08-0.4]	0.42 [0.17-0.93]	121.05	1.98 [0.86-4.2]	1.98 [0.83-4.38]	0 [-0.01 to 0.01]
Latvia	Male	6.39 [3.09-13.31]	6.65 [3.27-13.89]	4.07	47.32 [23.09-98.64]	47.89 [23.34-99.65]	0.04 [0.04 to 0.05]
Lebanon	Male	2.08 [1.01-4.47]	4.13 [2.03-8.6]	98.56	17.79 [8.71-37.89]	17.7 [8.68-36.76]	-0.03 [-0.03 to -0.02]

Lesotho	Male	2.56 [1.28-5.35]	3.16 [1.55-6.58]	23.44	55.03 [27.28-114.57]	54.24 [26.87-114.52]	-0.05 [-0.06 to -0.05]
Liberia	Male	2.44 [1.21-5.04]	4.86 [2.37-9.65]	99.18	40.35 [20.05-83.58]	40.27 [19.71-81.3]	-0.01 [-0.01 to 0]
Libya	Male	1.88 [0.91-3.91]	5.07 [2.46-10.66]	169.68	17.86 [8.76-36.97]	17.72 [8.77-37.54]	-0.03 [-0.03 to -0.03]
Lithuania	Male	4.38 [2.17-9.1]	5.09 [2.5-10.62]	16.21	24.56 [12.24-50.76]	24.82 [12.19-51.95]	0.25 [0.11 to 0.38]
Luxembourg	Male	1.56 [0.78-3.31]	3.13 [1.55-6.61]	100.64	67.98 [33.87-143.24]	67.86 [33.82-143.01]	0 [-0.01 to 0]
Madagascar	Male	10.93 [5.28-22.48]	24.62 [11.98-50.84]	125.25	40.57 [19.77-84.06]	40.8 [19.82-82.77]	0.02 [0.02 to 0.03]
Malawi	Male	7.81 [3.8-15.96]	14.46 [7.12-29.65]	85.15	40.41 [19.76-83.27]	40.51 [20.07-83.56]	0.02 [0.01 to 0.03]
Malaysia	Male	0.9 [0.37-1.99]	2.66 [1.09-5.75]	195.56	1.97 [0.84-4.28]	1.98 [0.82-4.25]	0.02 [0.01 to 0.03]
Maldives	Male	0.01 [0-0.02]	0.04 [0.02-0.08]	300	1.99 [0.83-4.37]	2 [0.86-4.34]	0.02 [0.01 to 0.03]
Mali	Male	8.78 [4.28-18.32]	19.34 [9.45-39.31]	120.27	40.57 [19.86-84.34]	40.9 [19.8-83.25]	0.04 [0.03 to 0.04]
Malta	Male	1.31 [0.64-2.78]	2.69 [1.34-5.67]	105.34	68.13 [33.47-143.96]	68.15 [33.74-145.63]	0 [0 to 0]
Marshall Islands	Male	0 [0-0.01]	0.01 [0-0.02]	Inf	6.12 [2.84-13]	6.06 [2.72-12.7]	-0.03 [-0.03 to -0.02]
Mauritania	Male	2.02 [0.99-4.15]	4.47 [2.22-9.19]	121.29	40.94 [20.39-83.91]	41.08 [20.36-84.55]	0.01 [0.01 to 0.01]
Mauritius	Male	0.07 [0.03-0.14]	0.16 [0.07-0.34]	128.57	1.97 [0.85-4.18]	1.96 [0.87-4.26]	-0.02 [-0.03 to -0.01]
Mexico	Male	133.78 [66.27-275.15]	422.35 [211.03-866.2]	215.7	62.57 [31.06-130.04]	74.67 [37.21-153.47]	0.85 [0.67 to 1.02]
Micronesia (Federated States of)	Male	0.01 [0.01-0.03]	0.02 [0.01-0.04]	100	6.13 [2.85-12.73]	6.12 [2.79-12.58]	0 [-0.01 to 0]
Mongolia	Male	1.97 [0.95-4.18]	4.59 [2.23-9.54]	132.99	40.26 [19.49-84.26]	40.25 [19.89-84.34]	0 [-0.01 to 0]
Montenegro	Male	0.81 [0.4-1.71]	1.27 [0.62-2.68]	56.79	28.73 [13.99-60.19]	28.77 [14.01-60.1]	0 [0 to 0]
Morocco	Male	12.42 [6.04-25.96]	29.15 [14.15-61.08]	134.7	17.89 [8.83-37.45]	17.81 [8.8-37.59]	-0.02 [-0.02 to -0.01]
Mozambique	Male	12.39 [6.11-25.68]	21.63 [10.51-44.71]	74.58	40.18 [19.67-82.6]	40.14 [19.43-82.83]	0 [-0.01 to 0.01]
Myanmar	Male	2.07 [0.86-4.68]	4.03 [1.65-9]	94.69	1.97 [0.84-4.3]	1.98 [0.84-4.37]	0.02 [0.02 to 0.03]
Namibia	Male	1.89 [0.93-4.01]	3.63 [1.77-7.55]	92.06	54.78 [26.75-115.43]	54.58 [27.13-113.92]	-0.01 [-0.02 to -0.01]
Nepal	Male	8.15 [3.88-17.18]	17.79 [8.51-37.34]	118.28	16.52 [7.98-34.37]	16.62 [8.06-34.58]	0.03 [0.02 to 0.03]
Netherlands	Male	39.84 [19.97-80.33]	72.31 [35.59-154.84]	81.5	46.97 [23.49-94.88]	48.56 [23.84-104.26]	-0.08 [-0.22 to 0.06]
New Zealand	Male	9.22 [4.58-19.3]	18.33 [9.05-38.29]	98.81	52.86 [26.25-110.97]	53.01 [26.21-110.92]	0.02 [0.01 to 0.02]
Nicaragua	Male	1.53 [0.74-3.21]	4.32 [2.11-9.11]	182.35	20.79 [10.22-43.48]	20.77 [10.26-43.47]	-0.01 [-0.01 to -0.01]
Niger	Male	6.55 [3.21-13.53]	16.72 [8.21-34.58]	155.27	40.84 [20.01-84.55]	40.98 [20.11-84.03]	0.02 [0.01 to 0.02]
Nigeria	Male	106.28 [51.84-221.48]	191.59 [93.92-398.22]	80.27	42.85 [21.28-89.94]	43.07 [21.37-90.23]	0.02 [0.02 to 0.03]
North Macedonia	Male	2.64 [1.28-5.59]	4.46 [2.19-9.46]	68.94	28.81 [14.03-60.68]	28.73 [14.26-61.19]	0 [0 to 0.01]
Northern Mariana Islands	Male	0.01 [0-0.02]	0.02 [0.01-0.04]	100	6.16 [2.8-12.75]	6.12 [2.8-12.72]	-0.01 [-0.02 to -0.01]

Norway	Male	21.56 [10.7-45.01]	33.55 [16.65-69.81]	55.61	78.62 [39.02-166.05]	78.96 [39.43-165.64]	0.16 [0.09 to 0.24]
Oman	Male	0.78 [0.37-1.65]	2.52 [1.15-5.34]	223.08	17.73 [8.82-37.51]	17.73 [8.59-37.33]	-0.01 [-0.01 to 0]
Pakistan	Male	56.94 [27.56-119.68]	106.61 [51.13-226.52]	87.23	18.26 [8.86-38.1]	18.2 [8.84-37.97]	-0.01 [-0.01 to 0]
Palestine	Male	0.7 [0.34-1.46]	2.27 [1.09-4.81]	224.29	17.77 [8.7-36.9]	17.62 [8.6-37.71]	-0.03 [-0.04 to -0.03]
Panama	Male	1.59 [0.79-3.32]	4.24 [2.08-8.87]	166.67	20.9 [10.31-43.58]	20.81 [10.13-43.69]	-0.01 [-0.02 to -0.01]
Papua New Guinea	Male	0.57 [0.26-1.2]	1.51 [0.65-3.18]	164.91	6.08 [2.79-12.57]	6.1 [2.82-12.45]	0 [0 to 0.01]
Paraguay	Male	1.71 [0.83-3.67]	4.32 [2.08-9.07]	152.63	15.87 [7.62-34.23]	15.84 [7.67-33.45]	-0.01 [-0.01 to 0]
Peru	Male	9.88 [4.76-20.74]	25.8 [12.61-54.76]	161.13	16.6 [8.04-34.73]	16.58 [8.09-34.91]	0.18 [0.06 to 0.29]
Philippines	Male	3.73 [1.58-8.17]	9.5 [3.97-20.86]	154.69	2.59 [1.14-5.58]	2.6 [1.11-5.59]	0.02 [0.01 to 0.02]
Poland	Male	55.72 [27.13-117.31]	88.68 [43.21-184.16]	59.15	30.58 [14.92-63.32]	30.83 [14.97-63.47]	0.03 [0.02 to 0.03]
Portugal	Male	27.79 [13.43-57.43]	43.89 [21.46-92.82]	57.93	46.2 [22.5-95.97]	46.41 [22.69-98.77]	-0.34 [-0.46 to -0.22]
Puerto Rico	Male	2.63 [1.26-5.5]	4.57 [2.2-9.66]	73.76	15.83 [7.63-33.12]	15.73 [7.53-33.26]	-0.02 [-0.03 to -0.02]
Qatar	Male	0.22 [0.1-0.46]	2.11 [0.98-4.43]	859.09	17.72 [8.66-38.08]	17.59 [8.65-36.42]	-0.03 [-0.04 to -0.02]
Republic of Korea	Male	44.57 [21.89-95.14]	144.72 [71.29-306]	224.7	33.91 [16.62-71.41]	34.17 [16.92-71.77]	0.07 [-0.19 to 0.34]
Republic of Moldova	Male	8.87 [4.28-18.74]	11.46 [5.6-23.83]	29.2	47.7 [23.23-99.94]	48.04 [23.54-99.3]	0.02 [0.02 to 0.03]
Romania	Male	36.45 [17.83-77.31]	44.2 [21.85-92.84]	21.26	28.61 [14.06-60.48]	28.82 [14.25-59.97]	0.03 [0.03 to 0.04]
Russian Federation	Male	1082.58 [541.38-2229.66]	1330.48 [668.14-2776.22]	22.9	157.18 [78.86-324.27]	145.11 [73.18-301.91]	-1.63 [-2.14 to -1.12]
Rwanda	Male	5.44 [2.67-11.2]	11.5 [5.61-23.55]	111.4	40.43 [19.98-83.33]	40.51 [19.78-83.78]	0.02 [0.01 to 0.03]
Saint Lucia	Male	0.06 [0.03-0.13]	0.16 [0.08-0.35]	166.67	15.79 [7.61-33.51]	15.74 [7.5-33.41]	-0.01 [-0.02 to -0.01]
Saint Vincent and the Grenadines	Male	0.05 [0.02-0.1]	0.11 [0.05-0.23]	120	15.86 [7.68-33.6]	15.76 [7.65-32.73]	-0.02 [-0.02 to -0.02]
Samoa	Male	0.03 [0.01-0.05]	0.04 [0.02-0.09]	33.33	6.13 [2.77-12.66]	6.13 [2.75-12.78]	0 [0 to 0.01]
Sao Tome and Principe	Male	0.13 [0.06-0.26]	0.23 [0.11-0.47]	76.92	40.87 [20.08-83.68]	40.72 [19.92-83.66]	-0.01 [-0.01 to -0.01]
Saudi Arabia	Male	23.64 [11.53-48.09]	84.84 [41.5-171.31]	258.88	57.88 [28.57-117.35]	57.57 [28.75-118.08]	-0.83 [-1.15 to -0.51]
Senegal	Male	6.88 [3.37-14.18]	15.63 [7.71-32.47]	127.18	40.64 [19.98-83.92]	40.77 [20.1-84.14]	0.01 [0 to 0.01]
Serbia	Male	15.62 [7.6-32.88]	19.83 [9.7-41.94]	26.95	28.75 [13.98-60.32]	28.65 [14.05-60.57]	-0.01 [-0.01 to 0]
Seychelles	Male	0 [0-0.01]	0.01 [0-0.02]	Inf	1.98 [0.86-4.35]	1.97 [0.85-4.26]	-0.01 [-0.02 to 0]
Sierra Leone	Male	4.05 [1.98-8.26]	7.94 [3.87-16.35]	96.05	40.6 [20.01-83.51]	40.73 [19.79-84.27]	0.02 [0.01 to 0.02]
Singapore	Male	3.13 [1.56-6.5]	12.09 [5.96-25.67]	286.26	29.95 [14.84-62.74]	30.21 [15.03-63.43]	0.04 [0.03 to 0.04]
Slovakia	Male	7.26 [3.54-15.32]	11.25 [5.57-23.76]	54.96	28.56 [14.02-59.89]	28.61 [14.2-60.47]	0.01 [0 to 0.01]
Slovenia	Male	2.87 [1.41-6.04]	5.18 [2.53-10.73]	80.49	28.37 [13.88-59.96]	28.65 [14-58.72]	0.02 [0.01 to 0.02]

Solomon Islands	Male	0.04 [0.02-0.09]	0.09 [0.04-0.2]	125	6.17 [2.82-12.54]	6.13 [2.82-12.68]	-0.03 [-0.03 to -0.03]
Somalia	Male	5.54 [2.71-11.24]	13.29 [6.43-27.18]	139.89	40.62 [19.82-83.41]	40.55 [19.76-83.35]	-0.01 [-0.01 to -0.01]
South Africa	Male	53.81 [26.83-110.92]	116.11 [57.6-239.25]	115.78	56.83 [28.43-116.04]	56.56 [28.32-115.82]	-0.02 [-0.02 to -0.02]
South Sudan	Male	5.62 [2.72-11.48]	9.01 [4.35-18.19]	60.32	40.12 [19.29-81.68]	40.08 [19.79-81.97]	0 [0 to 0.01]
Spain	Male	116.25 [56.96-241.22]	245.72 [103.6-559.37]	111.37	49.64 [24.31-103.36]	62.27 [26.18-142.49]	1.86 [1.42 to 2.3]
Sri Lanka	Male	1.07 [0.43-2.35]	2.23 [0.92-5.07]	108.41	1.97 [0.83-4.26]	1.97 [0.82-4.41]	-0.02 [-0.03 to -0.01]
Sudan	Male	8.85 [4.31-18.37]	18.88 [9.14-39.12]	113.33	17.85 [8.75-37.06]	17.81 [8.78-36.61]	-0.01 [-0.01 to 0]
Suriname	Male	0.2 [0.1-0.43]	0.45 [0.22-0.97]	125	15.86 [7.51-33.82]	15.72 [7.57-33.28]	-0.03 [-0.04 to -0.03]
Sweden	Male	24.1 [11.86-50.18]	34.39 [16.73-71.84]	42.7	38.95 [18.97-81.46]	38.7 [18.89-80.88]	-0.27 [-0.74 to 0.2]
Switzerland	Male	28.88 [14.35-61.31]	50.53 [24.99-107.07]	74.97	67.57 [33.45-143.82]	68.17 [33.59-144.12]	0.03 [0.03 to 0.03]
Syrian Arab Republic	Male	5.1 [2.49-10.77]	12.07 [5.89-25.44]	136.67	17.91 [8.87-37.55]	17.75 [8.77-37.13]	-0.02 [-0.03 to -0.01]
Tajikistan	Male	5.17 [2.52-10.67]	10.83 [5.2-22.59]	109.48	40.44 [19.94-84.66]	40.35 [19.92-84.4]	0 [-0.01 to 0]
Thailand	Male	3.32 [1.34-7.51]	9.38 [3.94-20.67]	182.53	1.99 [0.84-4.43]	1.99 [0.85-4.3]	0.03 [0.02 to 0.05]
Timor-Leste	Male	0.03 [0.01-0.07]	0.08 [0.03-0.17]	166.67	1.98 [0.85-4.33]	1.96 [0.84-4.32]	0.01 [-0.02 to 0.03]
Togo	Male	2.58 [1.27-5.31]	7.48 [3.61-15]	189.92	40.72 [20.16-84.27]	40.84 [19.94-83.51]	0.01 [0 to 0.01]
Tonga	Male	0.02 [0.01-0.03]	0.02 [0.01-0.05]	0	6.17 [2.8-12.59]	6.15 [2.79-12.89]	-0.01 [-0.01 to 0]
Trinidad and Tobago	Male	0.63 [0.31-1.35]	1.42 [0.69-3.04]	125.4	15.81 [7.73-33.4]	15.74 [7.65-33.51]	-0.02 [-0.02 to -0.01]
Tunisia	Male	4.73 [2.29-10]	11.28 [5.45-23.55]	138.48	17.87 [8.63-37.46]	17.77 [8.64-36.9]	-0.03 [-0.03 to -0.02]
Turkey	Male	7.49 [3.28-16.07]	18.19 [7.93-39.87]	142.86	4.29 [1.87-9.29]	4.28 [1.88-9.4]	-0.23 [-0.51 to 0.04]
Turkmenistan	Male	3.89 [1.88-8.05]	8.64 [4.22-18.69]	122.11	46.38 [22.66-96.01]	46.04 [22.5-98.52]	-0.03 [-0.04 to -0.02]
Uganda	Male	13.4 [6.47-27.29]	28.1 [13.71-57.34]	109.7	40.3 [19.64-81.84]	40.5 [19.71-82.11]	0.03 [0.02 to 0.03]
Ukraine	Male	91.8 [44.63-190.85]	100.74 [49.25-210.74]	9.74	34.63 [17.13-71.24]	35.24 [17.28-73.19]	0.32 [0.16 to 0.48]
United Arab Emirates	Male	0.81 [0.37-1.7]	9.06 [4.18-19.39]	1018.52	17.74 [8.67-37.59]	17.63 [8.61-36.88]	-0.02 [-0.02 to -0.01]
United Kingdom	Male	256.86 [124.85-543.19]	352.69 [172.61-739.77]	37.31	69.56 [33.72-147.23]	65.71 [32.13-138.57]	-0.41 [-0.59 to -0.22]
United Republic of Tanzania	Male	22.5 [11-46.26]	52.09 [25.49-104.64]	131.51	40.49 [19.82-82.61]	40.73 [19.88-83.16]	0.03 [0.02 to 0.03]
United States of America	Male	1671.27 [840.12-3498.74]	3031.1 [1524.19-6316.37]	81.37	126.3 [63.21-265.95]	122.87 [61.52-257.8]	0.05 [-0.4 to 0.5]
United States Virgin Islands	Male	0.07 [0.03-0.14]	0.13 [0.06-0.27]	85.71	15.88 [7.56-33.79]	15.79 [7.48-33.25]	-0.03 [-0.03 to -0.03]
Uruguay	Male	8.56 [4.12-18.06]	10.99 [5.38-23.63]	28.39	50.57 [24.35-107.38]	50.42 [24.55-107.97]	0 [-0.01 to 0]
Uzbekistan	Male	19.98 [9.6-41.5]	42.86 [20.46-89.7]	114.51	40.53 [19.75-84.9]	40.37 [19.71-84.49]	-0.01 [-0.01 to 0]
Vanuatu	Male	0.02 [0.01-0.05]	0.05 [0.02-0.11]	150	6.15 [2.79-12.85]	6.13 [2.79-12.77]	0 [-0.01 to 0.01]

Venezuela (Bolivarian Republic of)	Male	3.26 [1.49-7.15]	9.8 [4.43-21.25]	200.61	7.09 [3.28-15.32]	7.08 [3.27-15.45]	-0.08 [-0.12 to -0.04]
VietNam	Male	3.25 [1.32-7.2]	8.19 [3.37-18.17]	152	1.99 [0.82-4.25]	1.98 [0.85-4.29]	-0.01 [-0.02 to 0]
Yemen	Male	4.54 [2.2-9.41]	12.51 [5.94-26.07]	175.55	17.83 [8.8-37.29]	17.83 [8.66-37.41]	0 [0 to 0.01]
Zambia	Male	6.38 [3.07-13.05]	14.71 [7.21-29.89]	130.56	40.54 [19.56-82.08]	40.52 [19.73-82.82]	0 [0 to 0.01]
Zimbabwe	Male	11.89 [5.83-25.24]	18.26 [9.09-38.38]	53.57	55.14 [26.92-116.66]	54.74 [27.08-114.84]	-0.02 [-0.02 to -0.01]
Afghanistan	Female	13.96 [6.79-29.5]	26.92 [13.32-56.32]	92.84	39.37 [19.24-82.2]	28.79 [14.25-60.53]	-0.01 [-0.02 to -0.01]
Albania	Female	3.65 [1.76-7.78]	7.54 [3.71-16.11]	106.58	34.08 [16.48-72.33]	31.66 [15.37-66.95]	0.01 [0 to 0.01]
Algeria	Female	25.83 [12.47-53.74]	69.5 [33.94-145.91]	169.07	40.36 [19.66-83.83]	28.77 [14.12-60.19]	-0.01 [-0.01 to -0.01]
American Samoa	Female	0.01 [0.01-0.03]	0.03 [0.01-0.06]	200	11.54 [5.47-24.49]	8.84 [4.27-18.42]	-0.04 [-0.04 to -0.03]
Andorra	Female	0.27 [0.14-0.56]	0.69 [0.34-1.45]	155.56	100.32 [49.82-207.99]	139.19 [69.86-293.69]	-0.01 [-0.01 to 0]
Angola	Female	13.06 [6.46-27.29]	42.06 [20.72-87.84]	222.05	62.73 [31.28-129.77]	52.94 [26.08-109.49]	0.01 [0.01 to 0.02]
Antigua and Barbuda	Female	0.09 [0.05-0.19]	0.18 [0.09-0.36]	100	32.05 [15.71-67.69]	24.31 [11.78-49.85]	-0.02 [-0.02 to -0.02]
Argentina	Female	266.09 [132.48-551.56]	430.45 [215.28-892.48]	61.77	150.85 [75.56-313.54]	104.86 [52.01-218.47]	0 [0 to 0]
Armenia	Female	12.96 [6.37-27.41]	19.55 [9.68-41.1]	50.85	82.91 [40.78-174.87]	64.49 [31.75-134.35]	0 [0 to 0]
Australia	Female	156.11 [77.91-329.54]	326.96 [163.84-686.27]	109.44	154.61 [77.44-325.82]	109.07 [54.94-227.69]	-0.34 [-0.55 to -0.13]
Austria	Female	65.4 [32.32-135.85]	86.93 [43.08-182.89]	32.92	99.91 [49.76-207.43]	127.1 [63.29-267.09]	0.01 [0.01 to 0.01]
Azerbaijan	Female	30.3 [15.03-63.57]	56.85 [28.2-119.41]	87.62	102.6 [50.7-214.48]	72.32 [35.63-150.62]	0.3 [0.1 to 0.51]
Bahamas	Female	0.28 [0.14-0.58]	0.7 [0.34-1.48]	150	32.03 [15.94-67.42]	24.67 [11.96-51.66]	-0.01 [-0.01 to -0.01]
Bahrain	Female	0.34 [0.17-0.72]	1.82 [0.88-3.87]	435.29	39.91 [19.37-83.95]	26.26 [12.91-55.22]	-0.02 [-0.02 to -0.02]
Bangladesh	Female	67.81 [32.88-142.34]	206.59 [100.75-431.25]	204.66	32.12 [15.78-66.98]	24.23 [11.77-49.91]	0.02 [0.01 to 0.02]
Barbados	Female	0.5 [0.25-1.04]	0.84 [0.42-1.75]	68	32.08 [15.76-67.45]	24.59 [12.09-50.75]	-0.02 [-0.02 to -0.01]
Belarus	Female	70.11 [34.69-149.35]	82.69 [41.18-174.47]	17.94	87.32 [43.48-185.29]	71.86 [35.94-149.68]	0.02 [0.01 to 0.02]
Belgium	Female	81.39 [40.28-170.53]	108.02 [53.4-223.96]	32.72	99.92 [49.38-207.72]	108.04 [53.65-225]	-0.01 [-0.02 to -0.01]
Belize	Female	0.15 [0.07-0.3]	0.45 [0.22-0.94]	200	32.08 [15.88-66.71]	23.76 [11.62-48.74]	-0.02 [-0.02 to -0.02]
Benin	Female	6.61 [3.23-13.74]	17.18 [8.42-35.15]	159.91	62.57 [30.79-131.17]	52.64 [25.71-109.36]	0.02 [0.01 to 0.02]
Bermuda	Female	0.11 [0.06-0.23]	0.22 [0.11-0.46]	100	32.17 [16-67.53]	24.66 [12.1-51.44]	0 [0 to 0]
Bhutan	Female	0.4 [0.2-0.84]	0.89 [0.44-1.86]	122.5	32.18 [15.78-66.24]	24.3 [11.97-50.79]	0.02 [0.02 to 0.02]
Bolivia(Plurinational State of)	Female	8.75 [4.27-18.32]	23.84 [11.62-48.5]	172.46	50.63 [24.82-105.76]	35.68 [17.43-73.72]	0.01 [0.01 to 0.01]
Bosnia and Herzegovina	Female	7.9 [3.82-16.97]	10.76 [5.28-22.63]	36.2	33.85 [16.45-72.09]	31.43 [15.29-65.52]	-0.02 [-0.02 to -0.01]

Botswana	Female	2.36 [1.17-4.93]	5.92 [2.92-12.15]	150.85	71.56 [35.88-148.71]	63.74 [31.7-131.63]	-0.04 [-0.04 to -0.04]
Brazil	Female	162 [78.45-336.34]	449.65 [218.43-930.17]	177.56	34.01 [16.58-69.63]	26.64 [12.95-54.83]	0.02 [0.02 to 0.02]
Brunei Darussalam	Female	0.96 [0.48-2.02]	3.34 [1.68-7]	247.92	183.9 [92.33-380.54]	111.05 [55.79-230.76]	0.02 [0.02 to 0.02]
Bulgaria	Female	22.5 [11.03-47.3]	26.05 [12.8-55.28]	15.78	33.9 [16.55-70.94]	31.67 [15.69-66.64]	-0.01 [-0.01 to 0]
Burkina Faso	Female	14.92 [7.37-30.84]	33.56 [16.34-69.23]	124.93	62.51 [30.65-128.99]	53.06 [26.11-109.74]	0.03 [0.03 to 0.04]
Burundi	Female	8.12 [4.03-16.91]	14.73 [7.26-30.49]	81.4	62.7 [31.16-130.78]	51.32 [25.17-107.19]	0.02 [0.02 to 0.03]
Cabo Verde	Female	0.8 [0.39-1.67]	1.53 [0.76-3.27]	91.25	63.5 [31.18-132.5]	53.39 [26.29-111.65]	-0.01 [-0.02 to -0.01]
Cambodia	Female	1.32 [0.58-2.83]	3.64 [1.6-7.92]	175.76	5.31 [2.4-11.16]	3.96 [1.8-8.35]	0.02 [0.02 to 0.03]
Cameroon	Female	15.04 [7.41-31.11]	41.07 [20.23-86.05]	173.07	62.56 [31-130.73]	52.1 [25.79-108.9]	0.01 [0.01 to 0.02]
Canada	Female	323.2 [159.39-673.8]	631.89 [315.93-1330.6]	95.51	191.22 [94.28-398.84]	148.46 [74-310.61]	-0.01 [-0.01 to 0]
Central African Republic	Female	4.11 [2.04-8.56]	7.86 [3.91-16.35]	91.24	62.27 [30.98-129.08]	52.8 [26.2-109.12]	0.02 [0.02 to 0.02]
Chad	Female	9.29 [4.57-19.28]	17.46 [8.59-36.52]	87.94	62.82 [30.88-129.43]	50.84 [24.93-106.09]	-0.01 [-0.01 to 0]
Chile	Female	82.72 [41.19-171.66]	194.23 [96.81-410.43]	134.8	150.08 [74.84-310.83]	103.87 [51.52-215.21]	0 [0 to 0.01]
China	Female	1160.55 [567.01-2398.61]	3235.09 [1582.55-6848]	178.75	26.79 [13.22-55.21]	30.12 [14.75-62.79]	0.56 [0.33 to 0.79]
Colombia	Female	30.48 [14.51-65]	96.99 [46.79-204.19]	218.21	33.3 [16.17-70.35]	27.74 [13.35-58.42]	0.03 [0.02 to 0.03]
Comoros	Female	0.73 [0.35-1.5]	1.7 [0.83-3.52]	132.88	62.92 [30.9-130.89]	52.87 [26.04-109.48]	0.02 [0.02 to 0.02]
Congo	Female	3.82 [1.88-8.07]	9.14 [4.45-18.93]	139.27	62.57 [30.73-131.17]	52.06 [25.73-107.7]	0.01 [0 to 0.01]
Costa Rica	Female	2.99 [1.44-6.29]	9.36 [4.47-19.96]	213.04	33.34 [15.94-70.06]	27.58 [13.37-58.23]	0.01 [0 to 0.01]
Cote d'Ivoire	Female	12.66 [6.24-26.27]	34.51 [17.01-72.66]	172.59	62.01 [30.71-130]	51.27 [25.12-106.08]	0.03 [0.02 to 0.04]
Croatia	Female	13.33 [6.49-27.63]	15.38 [7.57-32.4]	15.38	35.64 [17.16-73.15]	31.42 [15.63-66.05]	-0.22 [-0.28 to -0.16]
Cuba	Female	16.41 [8.09-34.13]	31.54 [15.6-65.45]	92.2	31.83 [15.67-66.39]	24.21 [11.77-50.45]	0 [-0.01 to 0]
Cyprus	Female	4.32 [2.15-8.96]	9.81 [4.87-20.54]	127.08	99.79 [49.23-207.86]	117.57 [58.46-247.33]	0 [0 to 0.01]
Czechia	Female	26.22 [12.82-55.38]	36.1 [18.1-75.41]	37.68	33.55 [16.36-71.49]	31.24 [15.41-66.64]	-0.01 [-0.01 to 0]
Democratic People's Republic of Korea	Female	27.93 [13.38-59.36]	52.01 [25.55-108.27]	86.22	27.92 [13.62-59.49]	27.27 [13.29-57.28]	0 [0 to 0]
Democratic Republic of the Congo	Female	56.03 [27.78-115.94]	128.56 [63.22-269.76]	129.45	62.04 [30.82-127.95]	52.72 [26.18-110.37]	0.03 [0.03 to 0.03]
Denmark	Female	60.77 [30.71-125.25]	72.65 [35.59-153.7]	19.55	148.79 [74.9-307.66]	117.35 [57.9-246.68]	-0.26 [-0.33 to -0.19]
Djibouti	Female	0.48 [0.24-0.98]	1.94 [0.94-3.96]	304.17	63.02 [31.21-129.92]	51.14 [25.09-106.37]	0.01 [0.01 to 0.01]
Dominica	Female	0.13 [0.06-0.26]	0.14 [0.07-0.3]	7.69	32.01 [15.7-67.28]	23.8 [11.62-50.07]	-0.02 [-0.02 to -0.02]

Dominican Republic	Female	6.1 [3.02-12.8]	15.48 [7.67-31.99]	153.77	32.11 [15.92-66.66]	24.2 [11.86-49.43]	-0.02 [-0.02 to -0.01]
Ecuador	Female	21.03 [10.34-43.69]	71.09 [35.89-146.41]	238.04	76.54 [37.83-159.22]	58.25 [29.14-120.27]	0.72 [0.57 to 0.87]
Egypt	Female	60.58 [29.4-127.65]	125.09 [60.99-265.23]	106.49	40.24 [19.89-84.49]	27.9 [13.71-59.19]	-0.01 [-0.01 to -0.01]
El Salvador	Female	5.29 [2.56-11.09]	11.3 [5.53-23.73]	113.61	33.41 [16.06-70.45]	27.96 [13.6-59.22]	-0.01 [-0.01 to 0]
Equatorial Guinea	Female	0.71 [0.36-1.49]	1.86 [0.91-3.91]	161.97	61.96 [31.02-130.04]	53.39 [26.44-111.39]	0.04 [0.03 to 0.04]
Eritrea	Female	3.81 [1.87-7.91]	10.18 [5.06-21.41]	167.19	62.57 [31.26-129.36]	53.44 [26.59-110.27]	0.02 [0.01 to 0.02]
Estonia	Female	11.03 [5.47-23.18]	12.47 [6.21-26.77]	13.06	87.36 [43.51-182.34]	71.17 [34.94-148.99]	0.02 [0.02 to 0.02]
Eswatini	Female	1.24 [0.62-2.57]	2.51 [1.26-5.16]	102.42	71.54 [35.92-147.77]	64.17 [32.17-132.73]	-0.05 [-0.05 to -0.04]
Ethiopia	Female	64.59 [32.14-134.33]	139.92 [69.47-292.43]	116.63	65.24 [32.56-135.91]	54.18 [26.84-113.45]	0.05 [0.04 to 0.05]
Fiji	Female	0.21 [0.1-0.45]	0.45 [0.22-0.97]	114.29	11.52 [5.52-24.15]	8.93 [4.27-18.88]	-0.02 [-0.03 to -0.02]
Finland	Female	38.58 [19.35-80.18]	55.54 [27.47-116.11]	43.96	97.09 [48.4-204.62]	62.76 [30.89-130.99]	-0.02 [-0.04 to 0.01]
France	Female	437.81 [217.7-920.62]	652.58 [322.67-1367.3]	49.06	100.09 [50.29-209.68]	79.81 [39.96-168.54]	0.01 [0.01 to 0.01]
Gabon	Female	1.95 [0.96-4.09]	3.68 [1.81-7.68]	88.72	62.68 [30.95-130.85]	52.43 [26.13-108.76]	0 [0 to 0]
Gambia	Female	1.07 [0.53-2.26]	3.17 [1.58-6.57]	196.26	62.77 [30.94-132.02]	52.04 [25.9-106.96]	-0.01 [-0.01 to -0.01]
Germany	Female	550.85 [272.54-1163.13]	695.02 [351.69-1449.63]	26.17	77.5 [38.02-163.71]	53.64 [27.05-110.65]	-0.02 [-0.03 to -0.02]
Ghana	Female	21.44 [10.53-45.17]	59.99 [29.41-126.76]	179.8	62.84 [31.05-131.43]	53.38 [26.43-111.01]	0.01 [0 to 0.01]
Greece	Female	36.93 [18.31-76.01]	51.74 [25.85-106.31]	40.1	46.23 [22.87-95.25]	33.82 [16.8-69.61]	0.7 [0.42 to 0.99]
Greenland	Female	0.34 [0.17-0.7]	0.65 [0.32-1.37]	91.18	187.32 [93.48-390.62]	141.6 [70.74-293.47]	0.03 [0.02 to 0.03]
Grenada	Female	0.13 [0.06-0.26]	0.19 [0.09-0.39]	46.15	31.97 [15.78-66.68]	24.18 [11.85-50.41]	-0.02 [-0.02 to -0.01]
Guam	Female	0.04 [0.02-0.1]	0.11 [0.05-0.24]	175	11.63 [5.69-24.32]	8.95 [4.31-18.63]	0 [-0.01 to 0]
Guatemala	Female	6.25 [3.05-13.06]	20.2 [9.81-43.09]	223.2	33.09 [16.17-68.75]	27.53 [13.43-57.55]	0 [0 to 0]
Guinea	Female	10.6 [5.19-22.11]	17.92 [8.88-36.94]	69.06	62.81 [30.59-130.59]	51.75 [25.58-107.68]	0 [0 to 0.01]
Guinea-Bissau	Female	1.36 [0.67-2.83]	2.61 [1.27-5.43]	91.91	62.65 [30.91-130.14]	52.93 [25.95-109.02]	0 [0 to 0.01]
Guyana	Female	0.62 [0.31-1.31]	1.07 [0.53-2.25]	72.58	31.51 [15.65-65.47]	24.07 [11.82-49.86]	0 [0 to 0]
Haiti	Female	5.36 [2.63-11.13]	11.98 [5.84-24.94]	123.51	31.72 [15.58-66.02]	24.07 [11.8-49.81]	-0.02 [-0.02 to -0.01]
Honduras	Female	3.53 [1.7-7.49]	10.81 [5.2-22.7]	206.23	33.31 [15.95-70.17]	27.34 [13.24-56.93]	-0.01 [-0.01 to -0.01]
Hungary	Female	28.11 [13.76-59.44]	35.6 [17.6-74.12]	26.65	33.52 [16.32-70.98]	31.59 [15.5-66.84]	0.02 [0.02 to 0.02]
Iceland	Female	2.72 [1.36-5.56]	5.25 [2.61-10.94]	93.01	192.82 [95.1-386.55]	179.59 [88.5-373.71]	0.23 [-0.7 to 1.16]
India	Female	750.15 [371.59-1581.53]	2010.36 [996.11-4157.97]	167.99	34.09 [17.08-70.87]	26.41 [13.17-55.04]	0.03 [0.03 to 0.03]
Indonesia	Female	31.39 [14.02-67.57]	71.17 [32.09-151.71]	126.73	6.26 [2.9-13.28]	4.57 [2.09-9.42]	0.03 [0.03 to 0.03]

Iran (Islamic Republic of)	Female	87.88 [43.06-187.01]	262.57 [129.32-550.33]	198.78	67.58 [33.44-141.4]	46.17 [22.73-96.78]	0.06 [-0.11 to 0.23]
Iraq	Female	15.87 [7.68-33.78]	48.93 [23.7-104.46]	208.32	39.74 [19.51-84.51]	29.07 [14.2-60.89]	0.02 [0.02 to 0.03]
Ireland	Female	20.9 [10.4-43.95]	37.03 [18.19-77.83]	77.18	99.97 [49.77-208.95]	84.5 [41.9-177.88]	0 [0 to 0]
Israel	Female	33.91 [16.87-71.26]	78.29 [38.9-163.06]	130.88	135.16 [67.74-284.32]	119.73 [59.09-252.38]	-0.44 [-0.65 to -0.24]
Italy	Female	416.39 [207.58-864.85]	612.81 [310.23-1266.39]	47.17	87.16 [43.31-181.81]	71.95 [35.69-151.16]	0.32 [0.1 to 0.54]
Jamaica	Female	2.93 [1.43-6.1]	4.95 [2.47-10.33]	68.94	32.03 [15.65-66.91]	24.07 [11.9-50.43]	-0.02 [-0.03 to -0.02]
Japan	Female	1130.77 [553.15-2339.74]	2316.7 [1153.02-4892.98]	104.88	119.8 [58.43-247.75]	93.39 [46.08-197.07]	2.13 [1.67 to 2.6]
Jordan	Female	2.77 [1.34-5.85]	13.54 [6.51-28.84]	388.81	40.15 [19.47-84.61]	28.44 [13.82-60.14]	0.02 [0.01 to 0.02]
Kazakhstan	Female	63.84 [31.61-135.11]	85.61 [42.94-180.91]	34.1	82.52 [40.76-174.97]	65.11 [31.91-137.41]	-0.01 [-0.01 to 0]
Kenya	Female	28.39 [14.2-59.09]	80.27 [39.82-167.09]	182.74	65.5 [32.79-135.8]	55.04 [27.34-115.33]	0.02 [0.02 to 0.03]
Kuwait	Female	1.02 [0.49-2.15]	5.3 [2.57-10.78]	419.61	40.32 [19.74-85.67]	26.92 [13.18-57.2]	0 [0 to 0.01]
Kyrgyzstan	Female	14.91 [7.32-31.51]	22.37 [11.15-47.11]	50.03	83.01 [40.8-173.82]	64.81 [31.95-136.44]	0.02 [0.01 to 0.02]
Lao People's Democratic Republic	Female	0.56 [0.25-1.19]	1.21 [0.54-2.56]	116.07	5.34 [2.4-11.22]	3.75 [1.7-7.88]	0.02 [0.01 to 0.03]
Latvia	Female	19.27 [9.59-40.63]	19.3 [9.56-41.01]	0.16	86.99 [43.77-183.15]	71.49 [35.1-150.86]	0.02 [0.02 to 0.03]
Lebanon	Female	4.75 [2.31-10.18]	11.34 [5.49-23.62]	138.74	40.17 [19.53-85.38]	29.97 [14.79-63.21]	-0.02 [-0.02 to -0.01]
Lesotho	Female	3.99 [1.99-8.28]	5.39 [2.71-11.2]	35.09	71.58 [35.81-148.2]	64.07 [32.18-132.47]	-0.06 [-0.06 to -0.05]
Liberia	Female	3.22 [1.57-6.7]	6.75 [3.36-13.88]	109.63	61.91 [30.63-128.64]	50.83 [25.12-104.06]	0.01 [0.01 to 0.01]
Libya	Female	3.56 [1.74-7.47]	10.67 [5.23-22.56]	199.72	40.28 [19.83-85.23]	28.65 [14.09-60.84]	-0.03 [-0.03 to -0.02]
Lithuania	Female	14.18 [7.1-29.85]	16.94 [8.37-34.9]	19.46	52.03 [26.13-108.85]	41.19 [20.4-85.6]	0.26 [0.11 to 0.42]
Luxembourg	Female	2.99 [1.48-6.29]	4.93 [2.44-10.32]	64.88	99.92 [49.94-204.73]	83.96 [41.47-177.29]	-0.01 [-0.01 to -0.01]
Madagascar	Female	16.48 [8.2-34.38]	38.83 [19.04-80.64]	135.62	62.73 [31.06-129.84]	52.27 [25.71-108.35]	0.02 [0.01 to 0.02]
Malawi	Female	13.29 [6.48-27.92]	25.99 [12.72-54.39]	95.56	62.55 [30.64-131.37]	52.99 [26.19-109.8]	0.02 [0.02 to 0.03]
Malaysia	Female	2.5 [1.11-5.37]	7.12 [3.14-15.35]	184.8	5.33 [2.4-11.32]	3.66 [1.64-7.68]	0 [0 to 0.01]
Maldives	Female	0.02 [0.01-0.04]	0.08 [0.03-0.16]	300	5.35 [2.46-11.48]	3.52 [1.61-7.46]	0.03 [0.02 to 0.04]
Mali	Female	13.51 [6.58-28.25]	27.65 [13.68-57.55]	104.66	62.54 [30.9-130.65]	51.5 [25.21-107]	0.03 [0.02 to 0.03]
Malta	Female	2.34 [1.16-4.87]	4.36 [2.15-9.08]	86.32	100.01 [49.61-207.73]	84.55 [41.78-177.66]	0 [0 to 0]
Marshall Islands	Female	0.01 [0-0.02]	0.02 [0.01-0.04]	100	11.53 [5.49-24.38]	8.65 [4.13-18.1]	-0.03 [-0.04 to -0.03]
Mauritania	Female	3.34 [1.66-7.04]	6.91 [3.39-14.32]	106.89	63.1 [31.53-131.86]	52.14 [25.85-109.12]	0.01 [0.01 to 0.01]
Mauritius	Female	0.21 [0.09-0.45]	0.5 [0.23-1.09]	138.1	5.33 [2.39-11.45]	3.8 [1.73-8.04]	-0.02 [-0.03 to -0.01]
Mexico	Female	176.91 [87.29-367.89]	594.72 [298.04-1232.19]	236.17	77.97 [38.34-162.07]	84.34 [42.02-174.2]	0.84 [0.67 to 1.02]

Micronesia (Federated States of)	Female	0.03 [0.01-0.06]	0.04 [0.02-0.09]	33.33	11.58 [5.55-24.53]	8.99 [4.35-18.55]	-0.03 [-0.03 to -0.02]
Mongolia	Female	4.68 [2.32-9.89]	11.61 [5.76-24.31]	148.08	83.13 [41.22-175.63]	64.81 [31.65-135.56]	0.01 [0.01 to 0.02]
Montenegro	Female	1.18 [0.58-2.51]	1.78 [0.87-3.75]	50.85	33.91 [16.71-71.77]	31.54 [15.51-66.15]	-0.01 [-0.02 to -0.01]
Morocco	Female	28.4 [13.94-58.75]	66.15 [31.97-138.71]	132.92	40.26 [19.83-84.44]	29.08 [14.31-61.08]	-0.01 [-0.02 to -0.01]
Mozambique	Female	20.77 [10.15-42.88]	39.98 [19.77-83.26]	92.49	62.25 [30.64-127.83]	52.75 [26.44-108.4]	0.02 [0.01 to 0.03]
Myanmar	Female	6.4 [2.84-13.44]	13.95 [6.05-30.05]	117.97	5.34 [2.43-11.25]	3.9 [1.75-8.21]	0.01 [0 to 0.02]
Namibia	Female	2.81 [1.4-5.79]	5.95 [2.99-12.36]	111.74	71.39 [35.81-146.52]	64.17 [31.75-131.99]	-0.01 [-0.01 to 0]
Nepal	Female	14.85 [7.23-30.99]	38.05 [18.59-80.22]	156.23	31.95 [15.72-66.66]	24.78 [11.93-51.92]	0.02 [0.02 to 0.02]
Netherlands	Female	83.06 [42.61-168.24]	115.78 [58.46-243.83]	39.39	77.86 [39.7-159.6]	60.04 [29.79-127.79]	-0.45 [-0.71 to -0.18]
New Zealand	Female	33.44 [16.66-68.5]	63.82 [31.65-131.46]	90.85	165.09 [82.75-346.54]	112.58 [56.1-236.6]	0.02 [0.01 to 0.03]
Nicaragua	Female	2.73 [1.33-5.81]	8.31 [4.01-17.59]	204.4	33.32 [16.25-70.12]	27.72 [13.46-59.17]	0.01 [0 to 0.01]
Niger	Female	8.95 [4.41-18.68]	27.06 [13.32-57.04]	202.35	62.95 [31.16-132.19]	52.36 [26.02-109.38]	0.01 [0.01 to 0.01]
Nigeria	Female	136.24 [67.66-284.81]	329.16 [162.69-687.25]	141.6	65.48 [32.72-136.48]	55.05 [27.36-115.11]	0.02 [0.02 to 0.02]
North Macedonia	Female	3.34 [1.62-7.05]	5.59 [2.73-11.83]	67.37	33.73 [16.46-71.09]	31.36 [15.38-66.77]	0 [-0.01 to 0]
Northern Mariana Islands	Female	0.01 [0-0.02]	0.03 [0.01-0.07]	200	11.61 [5.59-24.58]	8.79 [4.2-18.6]	-0.02 [-0.03 to -0.02]
Norway	Female	53.07 [26.72-110.62]	71.61 [36.14-149.09]	34.93	159.57 [80.1-328.78]	119.7 [59.91-249.15]	0.22 [0.11 to 0.34]
Oman	Female	1.22 [0.6-2.56]	3.22 [1.57-6.78]	163.93	40.03 [19.68-84.81]	27.28 [13.2-57.78]	0 [-0.01 to 0]
Pakistan	Female	89.64 [44.91-190.93]	189.12 [93.63-397.16]	110.98	34.74 [17.29-72.52]	26.2 [12.8-55.13]	-0.01 [-0.01 to 0]
Palestine	Female	1.93 [0.95-4.11]	5.16 [2.51-10.85]	167.36	40.11 [19.73-85.32]	29.26 [14.33-61.86]	-0.01 [-0.02 to -0.01]
Panama	Female	2.47 [1.21-5.24]	7.13 [3.51-14.92]	188.66	33.41 [16.35-70.72]	27.25 [13.24-57.43]	0 [-0.01 to 0]
Papua New Guinea	Female	1.03 [0.48-2.16]	2.64 [1.26-5.69]	156.31	11.49 [5.44-23.9]	8.64 [4.2-17.89]	0 [-0.01 to 0.01]
Paraguay	Female	3.69 [1.81-7.71]	9.29 [4.55-19.09]	151.76	31.85 [15.68-66.65]	24.16 [11.91-49.9]	0 [-0.01 to 0]
Peru	Female	21.43 [10.56-45.06]	58.31 [28.93-123.92]	172.1	34.84 [17.24-73.14]	26.06 [12.95-54.36]	0.21 [0.08 to 0.35]
Philippines	Female	9.54 [4.33-20.14]	26.12 [11.97-55.51]	173.79	6.31 [2.91-13.39]	4.64 [2.15-9.51]	0.02 [0.01 to 0.02]
Poland	Female	91.05 [46.11-192.63]	139.3 [70.08-290.99]	52.99	36.22 [18.2-76.27]	34.13 [16.93-71.64]	0.03 [0.03 to 0.04]
Portugal	Female	99.05 [49.49-204.85]	151.61 [75.67-315.96]	53.06	130.66 [65.31-268.66]	92.43 [45.55-191.6]	0.36 [0.24 to 0.47]
Puerto Rico	Female	6.16 [3.04-12.94]	11.58 [5.75-24.07]	87.99	31.93 [15.73-66.92]	24.56 [11.98-51.54]	-0.01 [-0.02 to -0.01]
Qatar	Female	0.17 [0.08-0.36]	1.32 [0.64-2.76]	676.47	39.83 [19.4-83.7]	22.78 [11.21-47.38]	-0.03 [-0.04 to -0.02]
Republic of Korea	Female	406.95 [205.63-841.82]	1083.93 [540.63-2270.21]	166.35	226.2 [114.28-468.34]	135.2 [68.07-281.23]	0.73 [0.46 to 0.99]
Republic of Moldova	Female	22.75 [11.27-48.26]	29.1 [14.42-61.43]	27.91	87.04 [43.19-183.49]	70.96 [35.12-147.66]	0.01 [0.01 to 0.01]

Romania	Female	52.3 [25.67-111.32]	67.38 [33.14-143.22]	28.83	33.91 [16.77-72.38]	31.75 [15.54-67.4]	0.01 [0.01 to 0.02]
Russian Federation	Female	2437.93 [1225.85-4981.64]	2755.17 [1373.98-5734.23]	13.01	216.83 [109.97-440.63]	178 [89.5-370.48]	-0.8 [-1.01 to -0.59]
Rwanda	Female	10.27 [5.07-21.23]	22.96 [11.18-48.4]	123.56	62.63 [30.84-130.22]	53.54 [26.15-111.61]	0.03 [0.02 to 0.04]
Saint Lucia	Female	0.15 [0.07-0.32]	0.36 [0.18-0.75]	140	31.69 [15.68-66.96]	24.05 [11.77-49.19]	0 [-0.01 to 0]
Saint Vincent and the Grenadines	Female	0.12 [0.06-0.26]	0.21 [0.1-0.44]	75	31.91 [15.92-66.56]	23.54 [11.47-48.86]	-0.01 [-0.01 to -0.01]
Samoa	Female	0.05 [0.02-0.11]	0.09 [0.04-0.18]	80	11.6 [5.57-24.72]	8.86 [4.22-18.46]	-0.02 [-0.03 to -0.02]
Sao Tome and Principe	Female	0.21 [0.11-0.44]	0.37 [0.18-0.77]	76.19	63.11 [31.5-130.36]	52.24 [25.75-108.66]	-0.01 [-0.01 to 0]
Saudi Arabia	Female	22.36 [11.38-46.06]	77.24 [38.07-155.91]	245.44	87.51 [44.19-180.92]	69.58 [35.23-144.03]	-0.53 [-0.75 to -0.32]
Senegal	Female	10.4 [5.07-21.72]	25.63 [12.57-53.24]	146.44	62.78 [30.93-130.49]	52.23 [25.59-109.1]	0 [0 to 0]
Serbia	Female	21.07 [10.21-45.03]	27.54 [13.45-58.28]	30.71	33.81 [16.39-72.29]	31.38 [15.26-66.5]	-0.01 [-0.02 to -0.01]
Seychelles	Female	0.02 [0.01-0.04]	0.03 [0.01-0.07]	50	5.39 [2.39-11.61]	3.76 [1.73-7.85]	-0.04 [-0.05 to -0.03]
Sierra Leone	Female	5.93 [2.93-12.26]	11.7 [5.75-24.34]	97.3	62.84 [31.11-131.98]	51.72 [25.41-107.09]	0 [0 to 0.01]
Singapore	Female	23.31 [11.65-48.94]	75.89 [37.87-159.57]	225.57	185.48 [93.37-388.65]	108.11 [53.74-226.75]	0.4 [0.28 to 0.52]
Slovakia	Female	11.35 [5.61-24.2]	17.14 [8.37-36.4]	51.01	33.78 [16.67-72.2]	31.63 [15.38-66.77]	0.01 [0 to 0.01]
Slovenia	Female	4.82 [2.34-10.31]	7.39 [3.66-15.64]	53.32	33.57 [16.28-71.82]	31.43 [15.42-65.34]	0.01 [0.01 to 0.02]
Solomon Islands	Female	0.07 [0.03-0.15]	0.18 [0.08-0.37]	157.14	11.57 [5.45-23.87]	8.76 [4.22-18.03]	-0.02 [-0.02 to -0.02]
Somalia	Female	9.1 [4.49-18.8]	24.78 [12.21-51.92]	172.31	62.54 [30.95-129.96]	53.43 [26.58-111.58]	0.01 [0.01 to 0.01]
South Africa	Female	89.15 [44.97-187.21]	193.67 [97.47-404.66]	117.24	73.95 [37.31-154.62]	66.45 [33.21-137.17]	-0.02 [-0.03 to -0.02]
South Sudan	Female	6.59 [3.26-13.56]	12.66 [6.13-25.83]	92.11	61.5 [30.74-126.61]	50.3 [24.83-103.29]	0.01 [0.01 to 0.02]
Spain	Female	309.57 [154.34-653.57]	672.49 [267.12-1708.76]	117.23	106.6 [53.38-220.6]	106.59 [42.69-258.3]	1.97 [1.56 to 2.38]
Sri Lanka	Female	2.86 [1.26-5.99]	7.5 [3.34-16.25]	162.24	5.35 [2.38-11.05]	3.85 [1.74-8.23]	-0.02 [-0.03 to -0.01]
Sudan	Female	18 [8.77-38.16]	36.4 [17.72-76.49]	102.22	40.23 [19.68-85.12]	28.05 [13.96-58.8]	0 [-0.01 to 0]
Suriname	Female	0.44 [0.22-0.9]	1.04 [0.51-2.15]	136.36	31.83 [15.72-66.15]	24.28 [11.8-49.85]	-0.03 [-0.04 to -0.03]
Sweden	Female	41.58 [20.33-87.43]	52.98 [26.27-112.07]	27.42	54.88 [26.76-115.8]	46.94 [22.94-97.96]	-0.2 [-0.57 to 0.17]
Switzerland	Female	54.11 [27.01-112.14]	82.48 [40.8-173.25]	52.43	99.64 [49.23-207.65]	84.61 [41.98-176.3]	0.01 [0.01 to 0.01]
Syrian Arab Republic	Female	10.41 [5.12-22.03]	26.15 [12.7-55.19]	151.2	40.23 [19.9-84.49]	28.49 [13.98-60.85]	-0.01 [-0.02 to -0.01]
Tajikistan	Female	12.92 [6.35-27.09]	23.83 [11.75-50.56]	84.44	83.33 [40.95-174.57]	62.85 [30.67-132.68]	-0.01 [-0.01 to -0.01]
Thailand	Female	9.96 [4.42-21.29]	30.32 [13.57-65.42]	204.42	5.24 [2.35-11.31]	3.84 [1.7-8.17]	0.15 [0.13 to 0.18]
Timor-Leste	Female	0.08 [0.03-0.16]	0.21 [0.1-0.45]	162.5	5.32 [2.39-10.96]	3.65 [1.65-7.5]	0.03 [0.01 to 0.05]
Togo	Female	4.4 [2.16-9.24]	13.98 [6.84-29.39]	217.73	62.92 [31-131.46]	53.63 [26.56-112.04]	0.01 [0.01 to 0.01]

Tonga	Female	0.03 [0.02-0.07]	0.05 [0.02-0.1]	66.67	11.62 [5.51-24.18]	9.03 [4.37-18.99]	-0.01 [-0.02 to -0.01]
Trinidad and Tobago	Female	1.39 [0.67-2.86]	3.07 [1.52-6.36]	120.86	31.82 [15.46-65.62]	24.03 [11.99-49.36]	0 [0 to 0]
Tunisia	Female	10.12 [4.9-21.22]	26.72 [12.95-56.98]	164.03	40.48 [19.71-84.55]	29.34 [14.46-62.68]	-0.01 [-0.01 to -0.01]
Turkey	Female	21.8 [10.47-46.22]	54.53 [25.77-118.33]	150.14	11.6 [5.57-24.64]	8.15 [3.88-17.3]	-0.12 [-0.26 to 0.02]
Turkmenistan	Female	4.79 [2.34-9.99]	11.44 [5.63-24.16]	138.83	43.73 [21.54-91.14]	49.05 [24.19-102.49]	0.61 [0.43 to 0.78]
Uganda	Female	21.38 [10.55-44.88]	52.16 [25.74-109.16]	143.97	62.39 [30.98-130.37]	53.14 [25.79-110.09]	0.03 [0.03 to 0.04]
Ukraine	Female	317.41 [154.91-663.15]	330.35 [161.51-690.09]	4.08	71.36 [34.66-152.97]	57.95 [28.58-121.6]	0.31 [0.15 to 0.48]
United Arab Emirates	Female	0.64 [0.31-1.32]	5.42 [2.56-11.44]	746.88	39.87 [19.35-83.6]	23.57 [11.55-48.82]	-0.02 [-0.02 to -0.01]
United Kingdom	Female	405.97 [200.13-843.91]	572.44 [287.07-1189.65]	41.01	85.76 [42.41-178.14]	81.32 [40.18-170.64]	0.27 [-0.19 to 0.74]
United Republic of Tanzania	Female	36.24 [17.78-74.56]	85.39 [41.87-178.08]	135.62	62.46 [30.65-128.29]	52.3 [25.62-108.12]	0.02 [0.02 to 0.03]
United States of America	Female	3197.19 [1643.14-6641.13]	6269.71 [3133.83-13118.81]	96.1	195.17 [100.48-406.6]	176.31 [89.12-366.47]	0.44 [0.28 to 0.6]
United States Virgin Islands	Female	0.15 [0.07-0.32]	0.32 [0.15-0.66]	113.33	32.09 [15.92-66.77]	24.72 [11.98-50.98]	-0.01 [-0.02 to -0.01]
Uruguay	Female	31.37 [15.57-65.02]	41.72 [20.66-87.37]	32.99	151.13 [75.22-314.78]	105.68 [52.12-223.91]	0 [-0.01 to 0]
Uzbekistan	Female	53.73 [26.49-112.63]	103.76 [52.04-219.18]	93.11	83.14 [41.18-174.13]	64.17 [31.44-134.29]	-0.01 [-0.01 to 0]
Vanuatu	Female	0.03 [0.02-0.07]	0.09 [0.05-0.2]	200	11.56 [5.47-24.36]	8.69 [4.17-18.22]	-0.02 [-0.02 to -0.02]
Venezuela (Bolivarian Republic of)	Female	7.61 [3.73-16.11]	23.62 [11.46-50.07]	210.38	14.99 [7.39-32.02]	11.32 [5.44-24.07]	-0.01 [-0.02 to 0]
VietNam	Female	12.29 [5.53-25.45]	28.65 [12.6-58.69]	133.12	5.39 [2.44-11.16]	3.96 [1.83-8.18]	0.01 [0 to 0.01]
Yemen	Female	10.23 [4.89-21.15]	28.03 [13.61-59.08]	174	40.03 [19.44-83.6]	29.12 [14.03-61.72]	0.01 [0.01 to 0.01]
Zambia	Female	9.17 [4.54-18.86]	23.07 [11.33-49.05]	151.58	62.66 [31.12-129.49]	52.13 [25.93-109.08]	0.01 [0.01 to 0.02]
Zimbabwe	Female	15.75 [7.81-32.32]	30.36 [15.13-62.9]	92.76	71.66 [35.71-146.85]	64.33 [32.01-132.59]	-0.02 [-0.03 to -0.02]

Supplementary Table S6. Age distribution of DALYs rate (per 100,000) for hand osteoarthritis in different countries in 2019.

Supplementary Table S6. Age distribution of DALYs rate (per 100,000) for Osteoarthritis-hand in different countries in 2019.

country	sex	<5	5 to 9	10 to 14	15 to 19	20 to 24	25 to 29	30 to 34	35 to 39	40 to 44	45 to 49	50 to 54	55 to 59	60 to 64	65 to 69	70 to 74	75 to 79	80 to 84	85 to 89	90 to 94	95+
Afghanistan	Male	0	0	0	0	0	0	0.78	5.08	13.72	26.35	41.39	55.68	68.1	77.34	83.56	89.74	95.46	101.62	108.83	116.2
Afghanistan	Female	0	0	0	0	0	0	1.14	7.82	24.24	53.76	92.3	130.6	162.57	184.06	197.31	207.02	216.17	225.52	236.84	249.89
Albania	Male	0	0	0	0	0	0	0.91	6.33	18.95	39.68	65.64	93.33	117.59	135.34	147.8	156.76	166.5	174.57	184.76	194.65
Albania	Female	0	0	0	0	0	0	0.97	6.74	20.15	42.69	73.19	106.19	137.78	162.34	181.26	196.54	210.28	223.79	238.62	255.33
Algeria	Male	0	0	0	0	0	0	0.78	5.15	14.21	26.99	42.75	57.02	70.17	79.46	86.07	92.17	97.72	103.67	112.23	119.81
Algeria	Female	0	0	0	0	0	0	1.15	8.09	24.76	54.95	94.35	134.15	166.63	189.27	203.22	213.77	222.19	232.35	244.44	258.51
American Samoa	Male	0	0	0	0	0	0	0.26	1.62	3.74	6.6	10.96	16.65	22.87	28.25	33.34	38.41	44.56	50.91	59.13	68.21
American Samoa	Female	0	0	0	0	0	0	0.46	2.98	7.53	14.63	23.36	33.62	43.56	52.28	59.31	67.15	75.19	84.95	96.57	111.06
Andorra	Male	0	0	0	0	0	0	7.05	47.5	126.05	248.01	427.07	627.56	761.24	821.04	841.85	844.72	842.66	839.48	839.19	835.54
Andorra	Female	0	0	0	0	0	0	3.03	21.04	61.42	133.55	235.61	342.97	428.06	478.77	506.77	521.84	528.22	528.86	530.06	531.29
Angola	Male	0	0	0	0	0	0	1.7	11.9	35.11	68.65	103.21	134.77	158.68	174.4	185.38	194.64	203.39	212.49	221.7	231.77
Angola	Female	0	0	0	0	0	0	2.14	15.9	49.06	99.59	155.57	208.42	250.71	280.2	300.44	314.87	328.64	342.04	356.3	371.3
Antigua and Barbuda	Male	0	0	0	0	0	0	0.57	3.94	10.89	21.68	35.26	49.41	62.32	71.81	79.54	87.3	95.39	104.77	115.68	126.98
Antigua and Barbuda	Female	0	0	0	0	0	0	0.89	6.37	19.64	41.96	70.93	101.17	128	149.12	164.55	178.08	192.52	206.46	223.49	242.26
Argentina	Male	0	0	0	0	0	0	1.66	11.12	31.94	65.68	110.42	162.33	210.68	243.86	265.34	277.52	286.8	294.24	303.31	310.39
Argentina	Female	0	0	0	0	0	0	3.91	29.82	99.32	227.01	390.08	541.88	640.04	687.92	711.26	721.95	728.04	730.9	736.14	739.51
Armenia	Male	0	0	0	0	0	0	1.32	9.41	28.4	58.23	93.65	130.08	161.82	186.17	202.83	214.72	225.36	233.66	242.5	250.81
Armenia	Female	0	0	0	0	0	0	1.96	14.84	50.13	113.71	196.35	279.45	348.06	393.63	420.77	438.08	449.19	459.48	471.67	484.27
Australia	Male	0	0	0	0	0	0	1.65	11.23	31.8	65.13	109.47	161.16	209.07	241.3	260.72	272.32	281.53	288.14	293.21	298.18
Australia	Female	0	0	0	0	0	0	3.72	29.29	101.89	243.07	428.22	600.08	708.71	756.29	773.13	777.15	776.35	770.43	763.96	753.51
Austria	Male	0	0	0	0	0	0	6.25	42.59	112.24	220.64	376.61	553.52	678.78	734.89	755.1	761.1	762.65	761.21	761.96	759.68
Austria	Female	0	0	0	0	0	0	3.03	20.99	61.39	133.38	235.13	342.26	425.94	476.84	504.38	520.32	529.83	534	538.26	544.3
Azerbaijan	Male	0	0	0	0	0	0	1.18	8.39	24.71	50.52	81.3	113.35	141.62	164.23	179.42	191.99	201.38	210.81	218.94	227.92
Azerbaijan	Female	0	0	0	0	0	0	2.36	18.29	62	142.68	247.42	350.4	431.65	482.01	511.08	528.15	539.35	547.42	559.09	569.65
Bahamas	Male	0	0	0	0	0	0	0.57	3.92	10.9	21.65	35.13	49.77	62.26	71.75	79.93	87.75	96.25	105.32	116.04	128.25
Bahamas	Female	0	0	0	0	0	0	0.89	6.36	19.53	42.22	70.84	101.26	128.65	149.27	165.73	178.76	193.17	207.35	224.25	243.81
Bahrain	Male	0	0	0	0	0	0	0.78	5.25	14.19	27.18	42.06	57	68.84	77.58	83.93	89.64	95.69	101.71	108.81	117.24

Bahrain	Female	0	0	0	0	0	0	1.14	8.09	24.58	54.63	93.85	132.87	164.38	185.48	200.25	209.79	218.51	227.93	239.99	254.35
Bangladesh	Male	0	0	0	0	0	0	0.61	3.98	11.39	22.45	36.33	51.59	65.86	76.91	85.54	94.04	103.36	112.32	123.62	134.69
Bangladesh	Female	0	0	0	0	0	0	0.97	6.59	20.26	42.29	70.5	100.72	128.74	150.91	167.57	183.66	198.46	213.57	231.35	251.05
Barbados	Male	0	0	0	0	0	0	0.57	3.94	10.93	21.73	34.96	49.44	62.34	72.53	80.46	88.2	95.68	105.55	116.4	128.63
Barbados	Female	0	0	0	0	0	0	0.89	6.35	19.47	42.08	70.8	101.42	128.26	149.18	164.87	179.16	192.56	206.99	225.37	243.93
Belarus	Male	0	0	0	0	0	0	1.87	12.96	37.22	74.34	116.93	158.69	192.11	215.15	229.85	240.6	249.63	258.7	267.17	275.67
Belarus	Female	0	0	0	0	0	0	2.57	18.74	59.05	125.6	209.62	293.63	362.28	408.87	437.09	454.27	464.22	475.34	489.33	502.8
Belgium	Male	0	0	0	0	0	0	4.71	31.2	81.21	157.69	268.52	400.45	505.81	560.52	583.18	591.29	595.02	595.38	596.16	595.82
Belgium	Female	0	0	0	0	0	0	3.04	21.05	61.46	132.75	234.2	341.86	424.84	475.39	502.52	517.46	524.37	527.66	529.96	533.27
Belize	Male	0	0	0	0	0	0	0.57	3.92	10.91	21.52	34.99	49.58	62.2	72.36	80.58	87.9	96.6	105.49	116.5	127.91
Belize	Female	0	0	0	0	0	0	0.89	6.27	19.43	42.14	70.92	100.84	127.83	149.4	164.8	179.25	192.38	206.18	224.33	243.4
Benin	Male	0	0	0	0	0	0	1.71	12.13	35.52	68.88	104.57	136.08	160.39	176.04	186.34	195.73	203.94	211.99	221.38	231.11
Benin	Female	0	0	0	0	0	0	2.16	15.98	49.19	99.73	156.24	208.75	250.43	279.26	299.53	315.43	329.27	342.74	357.79	372.79
Bermuda	Male	0	0	0	0	0	0	0.57	3.91	10.94	21.52	35.24	49.61	62.62	72.38	80.18	87	95.71	104.93	115.21	126.76
Bermuda	Female	0	0	0	0	0	0	0.89	6.37	19.61	42.27	71.13	102.19	129.14	150.5	166.63	180.58	193.82	207.57	224.41	242.77
Bhutan	Male	0	0	0	0	0	0	0.61	4.09	11.22	22.46	36.62	51.64	65.44	76.28	85.47	93.96	102.67	111.92	122.72	134.13
Bhutan	Female	0	0	0	0	0	0	0.97	6.75	20.44	42.63	70.69	100.53	129.3	151.4	168.68	183.12	197.66	211.51	228.68	246.89
Bolivia (Plurinational State of)	Male	0	0	0	0	0	0	0.64	4.4	12.84	26.61	43.79	61.5	76.65	88.25	97.09	104.49	113.18	121.24	131.23	143.36
Bolivia (Plurinational State of)	Female	0	0	0	0	0	0	1.28	9.32	30.4	68.2	118.29	169.05	210.65	238.23	258.81	273.92	286.42	298.07	311.98	330.73
Bosnia and Herzegovina	Male	0	0	0	0	0	0	0.91	6.37	19.09	39.24	65.5	92.37	116.37	133.1	145.23	153.86	163.57	172.55	182.74	193.11
Bosnia and Herzegovina	Female	0	0	0	0	0	0	0.97	6.73	20.18	42.71	72.35	105.78	135.8	159.92	177.83	192.7	205.91	219.59	234.42	251.39
Botswana	Male	0	0	0	0	0	0	2.86	19.9	56.02	102.13	146.04	181.33	207.15	222.52	231.81	239.58	245.83	252.31	259.85	268.53
Botswana	Female	0	0	0	0	0	0	3.13	22.69	65.69	124.1	182.49	233.32	272.34	299.15	318.68	333.04	346.04	357.16	370.59	385.5
Brazil	Male	0	0	0	0	0	0	0.62	4.15	11.66	23.13	37.33	52.4	66.48	78.22	88.34	99.21	111.6	124.78	140.15	156.67
Brazil	Female	0	0	0	0	0	0	0.94	6.67	20.77	44.53	74.34	105.19	134.41	159.05	179.92	199.68	219.95	240.46	263.39	288.74
Brunei Darussalam	Male	0	0	0	0	0	0	0.84	5.66	15.35	31.74	56.4	88.2	121.92	149.02	169.03	181.28	190.97	198.6	208.44	217.87
Brunei Darussalam	Female	0	0	0	0	0	0	4.41	35.23	124.04	290.27	497.52	676.98	785.71	832.82	850.81	855.69	854.13	851.16	853.45	855.41
Bulgaria	Male	0	0	0	0	0	0	0.91	6.33	19.16	39.5	65.47	92.65	116.64	134.11	145.88	156.13	165.26	174.72	183.77	194.15

Bulgaria	Female	0	0	0	0	0	0	0.97	6.68	20.06	42.55	72.46	106.17	136.95	161.27	179.55	194.75	208.38	221.98	237.2	253.63
Burkina Faso	Male	0	0	0	0	0	0	1.7	12.11	35.6	69.09	104.16	135.8	160.31	176.13	186.61	195.49	204.3	212.33	222.14	231.71
Burkina Faso	Female	0	0	0	0	0	0	2.16	16.04	49.2	99.93	156.04	208.71	250.83	280.58	300.6	315.96	329.59	342.71	358.35	372.47
Burundi	Male	0	0	0	0	0	0	1.69	11.97	34.55	67.53	102.59	134.57	157.94	175.49	186.56	194.8	203.58	212.22	222.01	231.06
Burundi	Female	0	0	0	0	0	0	2.16	16	48.85	99.93	155.65	208.08	250.7	279.15	301.04	316.93	330.55	344.31	360.07	376.25
Cabo Verde	Male	0	0	0	0	0	0	1.71	12.16	35.69	69.19	104.32	136.27	160.11	176.55	186.8	195.95	205.15	212.74	222.56	231.19
Cabo Verde	Female	0	0	0	0	0	0	2.18	16	49.49	100.12	156.8	209.25	252.16	281.1	300.83	316.65	330.53	342.66	358.23	372.24
Cambodia	Male	0	0	0	0	0	0	0.11	0.68	1.5	2.5	3.8	5.21	6.85	8.48	10.11	12.07	14.49	17.54	22.22	27.99
Cambodia	Female	0	0	0	0	0	0	0.24	1.5	3.52	6	9.96	14.48	19.52	24.14	28.58	33.47	39.21	46.42	55.94	68.18
Cameroon	Male	0	0	0	0	0	0	1.71	12.21	34.91	68.51	103.98	135.39	158.94	174.85	185.56	194.11	202.93	210.89	220.08	229.75
Cameroon	Female	0	0	0	0	0	0	2.16	15.97	48.8	99.79	155.22	207.61	250	278.73	298.96	314.34	328.79	341.19	356.77	372.32
Canada	Male	0	0	0	0	0	0	4.77	29.56	69.18	130.81	227.19	347.29	449.93	500.28	518.38	524.39	523.3	520.6	519.38	516.22
Canada	Female	0	0	0	0	0	0	6.75	46.02	128.11	282	505.97	710.15	825.67	866.13	874.95	874.11	868.03	857.44	847.36	831.76
Central African Republic	Male	0	0	0	0	0	0	1.69	11.96	34.84	67.98	102.7	133.57	157.36	173.08	184.17	193.46	203.21	211.08	220.76	230.6
Central African Republic	Female	0	0	0	0	0	0	2.19	16.01	48.68	98.9	154.53	207.17	248.92	278.37	298.85	312.83	327.59	340.41	355.69	370.99
Chad	Male	0	0	0	0	0	0	1.7	11.93	35.31	68.75	103.75	135.19	159.35	175.21	186.25	194.26	203.44	211.26	220.08	230.21
Chad	Female	0	0	0	0	0	0	2.18	15.86	49.13	100.1	155.51	207.91	250.17	278.09	298.09	313.68	327.62	340.48	355.96	370.9
Chile	Male	0	0	0	0	0	0	1.67	11.4	32.34	65.13	110.65	161.77	209.51	243.25	263.18	274.81	284.74	291.82	300.39	307.79
Chile	Female	0	0	0	0	0	0	3.9	29.81	99.58	227.09	389.36	539.84	637.93	686.39	707.56	716.7	722.19	722.33	723.1	722.41
China	Male	0	0	0	0	0	0	0.75	5.25	15.42	32.88	57.89	89.62	122.24	148.72	168.48	184.31	199.25	212.64	226.21	238.19
China	Female	0	0	0	0	0	0	0.99	6.96	20.94	43.37	69.54	96.03	119.32	137.04	152.35	166.6	181.78	197.22	214.55	232.8
Colombia	Male	0	0	0	0	0	0	0.64	4.58	13.79	28.61	47.88	67.79	84.07	95.99	105.21	113.16	121.72	129.98	139.74	151.16
Colombia	Female	0	0	0	0	0	0	0.82	5.84	18.8	42.38	74.66	108.83	136.9	159.08	175.16	188.4	200.47	212.71	228.26	245.49
Comoros	Male	0	0	0	0	0	0	1.7	12.11	35.11	68.97	104.03	135.02	160.01	175.59	186.69	195.33	203.94	212.46	222.02	232.22
Comoros	Female	0	0	0	0	0	0	2.15	16.16	49.43	100.25	156.43	208.75	250.86	280.61	301.26	317.56	330.78	344.53	358.95	374.57
Congo	Male	0	0	0	0	0	0	1.7	12.03	35.22	68.61	103.94	134.97	159.03	175.65	185.87	195.44	204.1	212.47	222.24	232
Congo	Female	0	0	0	0	0	0	2.13	15.87	48.9	99.11	154.71	207.04	248.59	278.22	298.85	314.48	327.59	340.9	355.92	371.68
Costa Rica	Male	0	0	0	0	0	0	0.64	4.46	13.42	28.59	48.02	67.47	83.38	95.62	105.01	112.85	121.2	129.27	139.28	150.41
Costa Rica	Female	0	0	0	0	0	0	0.82	5.83	18.85	42.39	74.84	108.56	137.32	158.15	174.58	187.39	199.12	211.12	226.48	242.6
Côte d'Ivoire	Male	0	0	0	0	0	0	1.69	11.99	35.31	68.72	103.76	135.55	159.75	175.46	185.65	194.72	203.14	211.01	220.28	229.69

Côte d'Ivoire	Female	0	0	0	0	0	0	2.2	15.94	49.16	99.7	155.38	207.27	249.27	279.53	299.03	314.92	328.13	341.63	356.41	371.97
Croatia	Male	0	0	0	0	0	0	0.91	6.4	19.11	39.34	65.23	92.67	116.24	133.04	144.81	154.02	162.41	170.85	179.51	188.32
Croatia	Female	0	0	0	0	0	0	0.97	6.71	20.23	42.54	72.61	105.84	136.59	160.35	178.2	192.41	204.12	214.63	226.54	241.18
Cuba	Male	0	0	0	0	0	0	0.57	3.95	10.73	21.66	35.2	49.27	61.93	71.87	79.83	87.23	95.37	104.06	113.89	124.58
Cuba	Female	0	0	0	0	0	0	0.89	6.33	19.64	42.03	70.89	101.16	128.46	149.35	165.07	178.08	190.23	202.64	216.63	232.41
Cyprus	Male	0	0	0	0	0	0	5.54	36.8	95.9	187.41	320.71	476.89	591.66	649.7	670.65	677.95	678.68	678.7	681.16	680.36
Cyprus	Female	0	0	0	0	0	0	3.06	20.85	61.35	133.72	234.74	342.48	425.87	475.6	503.07	518.35	526.55	530.14	535.97	538.99
Czechia	Male	0	0	0	0	0	0	0.91	6.42	19.01	39.47	64.85	92.17	115.52	132.03	143.63	152.65	161.53	169.62	179.38	188.33
Czechia	Female	0	0	0	0	0	0	0.97	6.73	20.13	42.25	72.28	105.59	135.6	160.11	177.31	191.57	203.38	215.62	229.28	244.22
Democratic People's Republic of Korea	Male	0	0	0	0	0	0	0.71	4.77	13.43	27.89	50.63	81.38	114.66	140	156.43	166.94	175.8	184.69	194.14	203.98
Democratic People's Republic of Korea	Female	0	0	0	0	0	0	0.98	6.73	20.13	40.64	65.43	89.84	110.35	125.78	137.58	147.35	158.01	169.71	184.1	200.66
Democratic Republic of the Congo	Male	0	0	0	0	0	0	1.71	11.86	35	68.46	103.64	134.36	157.48	173.84	183.98	194.17	203.28	212.6	222.07	230.9
Democratic Republic of the Congo	Female	0	0	0	0	0	0	2.21	15.75	48.97	99.03	154.59	206.73	248.97	277.53	299.15	314.55	328.47	341.1	356.51	367.99
Denmark	Male	0	0	0	0	0	0	4.08	26.21	67.07	129.27	219.32	330.65	421.27	472.03	496.83	507.39	514.03	518.53	522.86	525.25
Denmark	Female	0	0	0	0	0	0	3.97	28.58	85.59	187.34	330.13	476.55	580.96	635.98	664.22	678.27	684.65	687.84	687.95	687.04
Djibouti	Male	0	0	0	0	0	0	1.7	12.11	35.18	68.8	104.1	136.06	160.02	176.35	187.09	196.04	204.21	212.49	221.45	231.8
Djibouti	Female	0	0	0	0	0	0	2.18	16.05	49.4	100.12	156.44	208.22	251.52	280.44	301.82	317.11	330.89	344.53	360.08	374.42
Dominica	Male	0	0	0	0	0	0	0.57	3.94	10.95	21.55	34.89	49.04	61.47	71.43	79.32	86.59	94.87	103.85	114.71	126.05
Dominica	Female	0	0	0	0	0	0	0.89	6.26	19.47	42.28	70.76	101.33	127.87	148.79	164.71	178.19	192.03	206.32	223.18	243.75
Dominican Republic	Male	0	0	0	0	0	0	0.57	3.9	10.9	21.64	35.07	49.38	62.25	72.48	80.48	88.1	96.64	105.64	116.38	127.73
Dominican Republic	Female	0	0	0	0	0	0	0.89	6.26	19.41	41.91	71.25	101.31	128.43	149.58	166.21	179.87	193.41	207.4	224.44	243.89
Ecuador	Male	0	0	0	0	0	0	0.91	6.03	17.23	35.74	59.38	82.55	100.89	113.58	123.06	130.8	138.71	146.18	156.62	167.54
Ecuador	Female	0	0	0	0	0	0	2.56	17.65	53.3	123.7	222.47	317.12	379.71	412.56	431.81	444.55	455.38	463.64	474.06	487.65
Egypt	Male	0	0	0	0	0	0	0.78	5.18	14.01	27.11	42.65	57.39	69.76	79.29	86.12	92.02	98.49	104.91	112.59	120.57
Egypt	Female	0	0	0	0	0	0	1.15	8.13	24.99	54.93	93.91	134.26	166.49	188.87	203.31	212.99	222.58	232.54	245.53	259.88
El Salvador	Male	0	0	0	0	0	0	0.64	4.5	13.44	28.7	47.6	66.56	82.65	94.91	104.04	112.27	120.91	128.57	137.82	149.27

El Salvador	Female	0	0	0	0	0	0	0.82	5.88	18.8	42.25	74.46	108.17	136.7	157.59	174.3	186.71	199.11	211.21	226.91	245.33
Equatorial Guinea	Male	0	0	0	0	0	0	1.71	12.24	35.41	68.38	103.76	134.68	158.38	174.26	185.19	193.8	202.66	210.82	220.26	228.82
Equatorial Guinea	Female	0	0	0	0	0	0	2.15	15.94	48.69	99.16	154.75	207.36	249.7	278.32	298.42	312.93	325.28	337.9	351.84	366.63
Eritrea	Male	0	0	0	0	0	0	1.7	11.98	35.07	67.98	102.89	132.76	156.84	173.58	184.41	193.28	201.51	210.03	219.49	229.58
Eritrea	Female	0	0	0	0	0	0	2.15	15.93	49.09	99.33	155.02	207.17	250.16	279.15	299.54	316.03	329.73	342.1	356.62	371.3
Estonia	Male	0	0	0	0	0	0	1.85	12.77	37.44	74.44	117.3	158.81	192.81	215.91	230.17	241.07	250.03	258.38	267.38	276.83
Estonia	Female	0	0	0	0	0	0	2.52	18.68	59.14	126.26	209.71	293.29	361.76	409.59	438.46	454.76	466.62	477.11	489.63	503.58
Eswatini	Male	0	0	0	0	0	0	2.87	19.83	55.14	101.33	144.71	181.48	206.44	221.26	230.48	238.46	245.85	253.23	262.8	271.4
Eswatini	Female	0	0	0	0	0	0	3.2	22.53	65.35	124.46	182.47	233.26	273.5	299.55	318.83	333.3	345.37	358.23	372.89	387.38
Ethiopia	Male	0	0	0	0	0	0	1.55	11.22	34.53	69.31	106.3	140.09	167.73	188.19	203.77	217.4	230.69	243.89	258.12	272.94
Ethiopia	Female	0	0	0	0	0	0	1.95	14.58	47.11	99.54	158.36	214.33	260.85	296.51	324.46	347.17	367.91	388.4	410.38	432.49
Fiji	Male	0	0	0	0	0	0	0.26	1.6	3.72	6.61	11.07	16.54	22.65	28.47	32.95	38.17	43.93	50.65	58.71	68.12
Fiji	Female	0	0	0	0	0	0	0.46	2.98	7.49	14.57	23.48	33.8	43.58	52.35	59.42	66.81	75.62	85.62	98.67	113.85
Finland	Male	0	0	0	0	0	0	1.02	6.22	15.11	28.73	49.17	75.66	102.52	122.99	136.64	145.4	152.67	159.61	167.39	175.81
Finland	Female	0	0	0	0	0	0	2.98	20.49	59.59	129.34	226.83	331.69	413.8	463.03	491.45	506.18	515.03	519.7	525.63	532.17
France	Male	0	0	0	0	0	0	2.3	14.59	36.77	70.65	118.9	179.62	237.51	276.36	297.79	309.71	318.23	322.67	328.08	333.48
France	Female	0	0	0	0	0	0	3.03	20.94	61.4	132.87	235.25	343.58	428.25	479.72	507.68	524.94	534.62	537.45	541	540.57
Gabon	Male	0	0	0	0	0	0	1.69	12.1	35.1	68.33	103.53	134.46	158.98	174.28	185.51	193.72	203.27	211.75	221.16	231.26
Gabon	Female	0	0	0	0	0	0	2.17	15.82	48.79	98.85	154.47	206.76	249.52	279.51	299.87	315.92	329.02	342	357.23	372.81
Gambia	Male	0	0	0	0	0	0	1.71	12.24	35.21	68.95	104.71	136.07	159.96	177.06	187.17	195.38	205.25	212.93	221.8	231.75
Gambia	Female	0	0	0	0	0	0	2.16	15.97	48.57	98.96	155.11	206.65	248.95	279.21	298.99	313.77	327.63	341.04	356.33	370.91
Germany	Male	0	0	0	0	0	0	1.12	7.13	17.37	32.44	55.18	84.72	114.65	137.33	152.31	162.61	170.93	177.84	185.68	193.36
Germany	Female	0	0	0	0	0	0	2.4	16.35	47.17	99.92	175.07	258.03	326.45	371.01	397.24	412.59	422.95	429.32	435.38	443.51
Ghana	Male	0	0	0	0	0	0	1.69	12.06	35.14	69.14	104.42	135.94	159.61	175.84	187.26	195.45	204.62	212.63	222.41	232.26
Ghana	Female	0	0	0	0	0	0	2.17	16.1	49.22	100.43	156.15	208.19	251.01	280.33	301.27	317.18	330.92	345.55	360.89	375.65
Greece	Male	0	0	0	0	0	0	0.8	4.89	11.74	22.46	38.51	59.16	80.67	99.09	110.65	119.46	127.12	133.78	141.79	151.13
Greece	Female	0	0	0	0	0	0	1.46	9.8	27.29	57.02	99.54	148.04	191.71	223.39	245.51	260.78	272.84	282.6	294.05	307.43
Greenland	Male	0	0	0	0	0	0	4.74	29.73	69.05	130.26	225.22	344.2	444.72	495.06	511.13	514.09	514.14	512.93	511.45	505.93
Greenland	Female	0	0	0	0	0	0	6.74	45.54	127.05	279.98	501.01	705.24	815.67	855.67	860.99	857.02	848.04	837.37	825.7	807.06
Grenada	Male	0	0	0	0	0	0	0.57	3.92	10.92	21.39	35.1	49.45	61.9	71.2	79.24	86.43	95.17	103.91	114.26	126.07

Grenada	Female	0	0	0	0	0	0	0.89	6.38	19.68	41.9	70.39	101.35	127.45	148.6	164.76	177.72	191.64	205.72	222.65	242.12
Guam	Male	0	0	0	0	0	0	0.26	1.61	3.73	6.75	10.99	16.79	23.17	29.06	34.19	39.27	45.4	52.15	59.82	69.25
Guam	Female	0	0	0	0	0	0	0.46	2.98	7.54	14.65	23.75	34.38	44.38	53.27	60.91	68.41	77.47	86.74	99.56	113.92
Guatemala	Male	0	0	0	0	0	0	0.64	4.47	13.51	28.49	47.49	66.67	82.88	94.9	103.93	111.77	120.42	128.12	138.01	149.32
Guatemala	Female	0	0	0	0	0	0	0.82	5.82	18.57	42.39	73.72	107.9	135.67	156.48	173.2	185.18	197.12	209.27	224.98	241.81
Guinea	Male	0	0	0	0	0	0	1.7	12.04	35.32	69.17	104.35	135.89	159.87	175.86	186.61	195.44	204.04	211.78	222.14	230.75
Guinea	Female	0	0	0	0	0	0	2.14	16.07	48.84	99.75	155.59	207.84	249.87	279.31	299.36	314.86	328.3	342.3	357.77	373.61
Guinea-Bissau	Male	0	0	0	0	0	0	1.71	12.06	35.3	68.66	104.14	135.75	159.72	175.63	186.51	195.49	203.96	212.26	222.31	231.75
Guinea-Bissau	Female	0	0	0	0	0	0	2.19	16.16	48.9	99.43	155.33	208.18	250.46	279.16	298.78	314.67	329.3	342.22	357.75	373.32
Guyana	Male	0	0	0	0	0	0	0.57	3.91	10.82	21.36	34.6	48.56	61.09	70.64	78.39	86.08	94.69	103.96	114.48	126.1
Guyana	Female	0	0	0	0	0	0	0.89	6.28	19.47	41.77	69.87	100.01	126.31	147.1	162.32	176.88	189.84	204.22	221.72	240.63
Haiti	Male	0	0	0	0	0	0	0.57	3.9	10.84	21.46	34.63	48.95	61.86	71.46	79.44	87.04	95.48	104.7	114.58	126.4
Haiti	Female	0	0	0	0	0	0	0.89	6.26	19.47	41.36	70.21	99.81	126.97	147.54	163.34	176.18	190.39	204.55	222.15	241.73
Honduras	Male	0	0	0	0	0	0	0.64	4.49	13.56	28.76	47.83	67.1	83.01	95.72	104.72	112.93	121.23	129.03	139.53	151.37
Honduras	Female	0	0	0	0	0	0	0.82	5.92	18.78	42.38	74.32	107.63	135.85	157.27	172.75	186.26	198.43	210.66	225.72	243.87
Hungary	Male	0	0	0	0	0	0	0.91	6.41	19.02	39.51	65.5	92.45	116.01	133.25	144.46	154.12	162.67	171.84	180.87	190.3
Hungary	Female	0	0	0	0	0	0	0.97	6.62	20.26	42.56	72.41	105.55	136.75	160.67	178.99	192.77	205.53	217.93	231.15	247.55
Iceland	Male	0	0	0	0	0	0	6.64	41.71	98.45	182.89	339.97	565.14	724.8	797.38	817.81	823.03	822.13	819.5	818.32	813.7
Iceland	Female	0	0	0	0	0	0	6.3	46.11	131.93	272.36	488.4	720.25	864.66	930.14	955.94	965.13	963.83	957.09	950.42	940.39
India	Male	0	0	0	0	0	0	0.66	4.41	12.22	24.07	38.76	54.34	69.09	81.42	92.16	103.23	115.82	129.2	145.43	162.38
India	Female	0	0	0	0	0	0	0.98	6.88	21.1	44.74	74.06	105.18	134.21	158.41	179.53	199.06	219.95	241.12	265.72	292.1
Indonesia	Male	0	0	0	0	0	0	0.15	0.93	2.01	3.33	4.84	6.86	8.85	10.8	12.96	15.73	19.53	24.3	30.63	38.69
Indonesia	Female	0	0	0	0	0	0	0.28	1.81	4.11	7.31	11.57	16.85	22.39	27.85	33.28	39.64	48.11	58.23	71.39	87.13
Iran (Islamic Republic of)	Male	0	0	0	0	0	0	0.93	6.46	18.36	35.92	55.9	75.64	91.5	104.36	114.68	123.32	134.11	144.19	157.88	181.14
Iran (Islamic Republic of)	Female	0	0	0	0	0	0	1.7	12.89	43.5	98.17	167.09	232.74	281.32	316.02	340.42	357.38	372.12	384.44	405.11	435.68
Irap	Male	0	0	0	0	0	0	0.78	5.08	13.93	26.89	42.22	56.8	68.97	78.48	85.41	91.27	97.67	103.74	110.99	119.08
Irap	Female	0	0	0	0	0	0	1.15	8.04	24.68	54.45	93.54	133.23	165.11	187.79	201.69	212.49	222.22	231.86	244.29	258.18
Ireland	Male	0	0	0	0	0	0	2.81	17.93	45.52	86.82	146.96	221.43	288.99	332.32	354.13	364.56	371.97	376.73	384.84	390.41
Ireland	Female	0	0	0	0	0	0	3.06	20.93	61.52	133.19	234.8	342.69	426.16	475.52	502.97	518.64	529.27	534.91	540.82	548.35
Israel	Male	0	0	0	0	0	0	4.13	27.2	69.95	134.86	228.87	344.66	438.47	493.24	518.06	528.9	534.32	538.56	542.94	547.1

Israel	Female	0	0	0	0	0	0	4.02	28.13	85.06	186.26	330.04	476.95	580.12	636.93	665.29	679.03	685.68	687.93	692.76	694.42
Italy	Male	0	0	0	0	0	0	1.62	11.3	33.07	67.51	112.1	162.17	208.78	244.87	270.42	288.58	301.67	312.88	324.61	336
Italy	Female	0	0	0	0	0	0	2.16	16.27	53.51	120.8	208.45	298.35	373.22	427.84	465.11	490.87	506.46	517.55	531.01	545.76
Jamaica	Male	0	0	0	0	0	0	0.57	3.97	10.89	21.66	35.05	49.36	61.82	72.12	79.93	87.18	95.54	104.99	115.86	128
Jamaica	Female	0	0	0	0	0	0	0.89	6.4	19.47	41.98	70.49	101.3	127.98	148.81	164.35	178.31	192.8	207.15	224.3	244.33
Japan	Male	0	0	0	0	0	0	0.74	5.14	15.1	32.07	55.86	85.59	117.29	145.72	169.13	188	204.3	217.94	232.96	247.54
Japan	Female	0	0	0	0	0	0	3.04	25.41	94.47	226.52	388.92	537.11	642.77	705.2	738.79	757.49	768.02	774.86	782.19	788.4
Jordan	Male	0	0	0	0	0	0	0.78	5.15	14.15	27.27	42.33	57.25	70.13	79.15	86.57	91.99	97.81	104.35	111.75	120.03
Jordan	Female	0	0	0	0	0	0	1.15	8.01	24.92	54.7	94.15	134.12	166.46	189.71	204.1	214.68	223.59	233.18	246.53	260.88
Kazakhstan	Male	0	0	0	0	0	0	1.32	9.47	28.32	58.04	93.75	129.13	160.77	184.96	201.34	213.72	223.9	232.93	241.7	249.63
Kazakhstan	Female	0	0	0	0	0	0	1.97	14.8	49.85	113.63	195.24	277.23	345.8	390	418.49	436.09	448.68	459.36	471.24	484.92
Kenya	Male	0	0	0	0	0	0	1.55	11.27	34.52	69.33	106.08	139.51	166.88	186.72	201.82	214.99	228.35	241.41	256.09	270.72
Kenya	Female	0	0	0	0	0	0	1.95	14.54	46.89	99.03	157.64	213.33	260.28	296.24	323.83	347.02	368.7	389.41	411.81	434.35
Kuwait	Male	0	0	0	0	0	0	0.78	5.21	13.89	27.07	42.45	57.33	69.46	79	85.73	91.03	97.73	104.19	111.63	119.09
Kuwait	Female	0	0	0	0	0	0	1.15	7.95	24.74	54.56	94.44	134.03	166.66	188.87	204.29	214.73	224.34	233.78	245.84	260.56
Kyrgyzstan	Male	0	0	0	0	0	0	1.33	9.4	28.42	58.52	94.47	130.8	162.32	187.34	204.83	217.43	227.43	236.25	244.95	252.96
Kyrgyzstan	Female	0	0	0	0	0	0	1.96	14.9	49.68	114.15	195.75	279.7	349.62	395.57	423.6	442.32	453.78	465.1	478.48	492.03
Lao People's Democratic Republic	Male	0	0	0	0	0	0	0.11	0.68	1.5	2.52	3.78	5.21	6.86	8.48	10.1	12.08	14.64	17.9	22.53	28.7
Lao People's Democratic Republic	Female	0	0	0	0	0	0	0.24	1.51	3.5	6.15	9.84	14.37	19.53	24.28	28.64	33.35	39.73	47.2	57.01	69.53
Latvia	Male	0	0	0	0	0	0	1.85	12.91	37.15	74.11	117.08	158.65	192.22	214.91	229.7	240.42	249.81	258.06	267.42	275.15
Latvia	Female	0	0	0	0	0	0	2.55	18.82	59.58	125.42	209.37	292.65	361.39	407.6	435.83	453.59	464.99	473.95	487.76	501.79
Lebanon	Male	0	0	0	0	0	0	0.78	5.19	14.1	27.1	42.51	57.05	68.96	78.25	85.01	90.73	96.8	102.9	110.36	118.26
Lebanon	Female	0	0	0	0	0	0	1.15	8.04	24.85	54.29	93.86	133.49	166.24	188.49	202.23	212.96	221.01	230.14	242.42	255.08
Lesotho	Male	0	0	0	0	0	0	2.83	19.75	55.17	101.67	145.19	181.86	207.23	223.26	232.28	239.71	247.45	255.1	264.42	272.68
Lesotho	Female	0	0	0	0	0	0	3.2	22.62	65.26	123.96	181.69	233.22	272.01	299.28	318.51	333.18	345.95	357.49	371.38	386.44
Liberia	Male	0	0	0	0	0	0	1.7	11.94	34.99	68.2	102.67	133.74	157.59	172.96	184.24	192.84	202.46	211.13	220.49	230.05
Liberia	Female	0	0	0	0	0	0	2.17	15.73	48.86	98.64	153.62	205.46	246.71	275.81	295.65	310.83	324.76	338.15	352.87	366.96
Libya	Male	0	0	0	0	0	0	0.78	5.14	14.06	27.03	42.35	56.96	69.53	78.66	85.21	91.15	96.71	102.55	110.16	118.25

Libya	Female	0	0	0	0	0	0	1.15	8.01	24.95	54.34	93.57	133.12	165.93	187.89	202.09	212.35	221.51	231.2	243.4	257.26
Lithuania	Male	0	0	0	0	0	0	0.98	6.72	18.39	36.07	56.86	78.55	98.01	112.7	123.74	132.87	141.48	149.62	158.85	169.18
Lithuania	Female	0	0	0	0	0	0	1.64	11.43	34.41	71.35	117.76	167.44	212.43	246.35	270.33	286.72	298.46	310.16	325.75	340.9
Luxembourg	Male	0	0	0	0	0	0	2.78	17.96	45.7	86.98	146.85	221.52	287.67	330.25	351.99	363.15	368.98	374.79	377.58	381.85
Luxembourg	Female	0	0	0	0	0	0	3.04	20.75	61.26	132.78	234.21	341.22	424.67	473.65	501.56	515.69	524.59	528.15	532.68	535.91
Madagascar	Male	0	0	0	0	0	0	1.7	11.98	35.44	68.77	103.77	135.76	160.09	176.06	187.32	195.61	203.97	213.16	222.46	232.23
Madagascar	Female	0	0	0	0	0	0	2.17	16.04	49.45	99.74	155.62	207.77	250.38	279.26	300.02	315.81	330.02	343.54	358.67	373.53
Malawi	Male	0	0	0	0	0	0	1.7	11.98	34.93	68.42	103.25	134.98	158.75	174.9	185.8	194.18	202.13	211.26	219.73	229.79
Malawi	Female	0	0	0	0	0	0	2.15	15.94	48.96	98.78	154.48	207.13	251.03	279.6	300.4	315.68	330.13	343.3	358.47	372.36
Malaysia	Male	0	0	0	0	0	0	0.11	0.68	1.5	2.52	3.81	5.13	6.92	8.47	10.19	12.12	14.62	17.84	22.31	28.22
Malaysia	Female	0	0	0	0	0	0	0.24	1.5	3.49	6.08	10.06	14.51	19.58	24.04	28.61	33.35	39.4	46.58	56.37	68.41
Maldives	Male	0	0	0	0	0	0	0.11	0.68	1.51	2.53	3.83	5.28	6.92	8.51	10.27	12.21	14.63	17.98	22.5	28.35
Maldives	Female	0	0	0	0	0	0	0.24	1.51	3.5	6.07	9.98	14.55	19.38	24.31	28.71	33.73	39.99	46.53	56.26	68.61
Mali	Male	0	0	0	0	0	0	1.7	12.14	35.21	68.82	104.32	136.38	160.82	176.46	187.06	195.77	204.88	212.69	222.38	231.08
Mali	Female	0	0	0	0	0	0	2.2	15.95	48.94	99.89	155.6	207.93	250.61	279.26	299.79	314.75	328.94	341.26	355.52	371.07
Malta	Male	0	0	0	0	0	0	2.77	18.1	45.46	86.92	147.03	221.92	288.98	332.57	354.24	365.42	371.39	377.22	383.75	389.77
Malta	Female	0	0	0	0	0	0	3.05	20.88	61.81	133.3	234.62	341.77	425.37	475.82	501.89	518.46	528.59	534.4	541.44	548.67
Marshall Islands	Male	0	0	0	0	0	0	0.26	1.61	3.77	6.64	10.86	16.55	22.73	28.43	33.47	38.4	44.9	50.86	59.36	68.68
Marshall Islands	Female	0	0	0	0	0	0	0.46	2.93	7.5	14.36	23.66	33.48	43.39	51.89	59.53	66.96	75.64	85.71	97.8	112.74
Mauritania	Male	0	0	0	0	0	0	1.69	12.16	35.49	69.09	104.9	136.36	161.2	177.2	188.5	197.18	205.99	214.21	223.9	233.76
Mauritania	Female	0	0	0	0	0	0	2.16	16.04	49.45	100.48	156.44	209.11	251.68	280.73	302.02	316.85	330.56	343.9	359.64	375.06
Mauritius	Male	0	0	0	0	0	0	0.11	0.68	1.49	2.5	3.79	5.17	6.74	8.47	9.95	11.9	14.51	17.71	22.01	27.83
Mauritius	Female	0	0	0	0	0	0	0.24	1.5	3.47	6.09	10	14.52	19.15	23.85	28.17	33.34	39.49	46.8	55.85	68.17
Mexico	Male	0	0	0	0	0	0	1.68	13.56	49.11	117	197.71	265.24	308.41	333.36	349.1	361.21	372.66	383.62	396.83	407.79
Mexico	Female	0	0	0	0	0	0	1.69	13.74	52.19	131.32	233.72	325.77	389.44	428.68	455.39	475.33	493.12	509.77	529.22	548.12
Micronesia (Federated States of)	Male	0	0	0	0	0	0	0.26	1.62	3.76	6.63	11.16	16.68	22.94	28.52	33.94	38.88	45.07	51.57	59.96	69.64
Micronesia (Federated States of)	Female	0	0	0	0	0	0	0.46	3	7.53	14.54	23.59	33.83	43.72	52.29	60.02	67.7	76.51	86.35	98.65	113.6
Mongolia	Male	0	0	0	0	0	0	1.32	9.35	28.45	58.12	93.4	129.25	161.47	185.81	202.91	215.76	226.07	234.03	242.15	251

Mongolia	Female	0	0	0	0	0	0	1.96	14.86	50.01	113.87	196.03	281.13	350	395.64	423.96	442.64	452.76	464.45	476.83	488.57
Montenegro	Male	0	0	0	0	0	0	0.91	6.41	18.89	39.74	65.81	93.21	116.43	133.69	146.11	155.29	164.62	173.7	182.9	192.66
Montenegro	Female	0	0	0	0	0	0	0.97	6.67	20.12	42.73	72.62	105.57	136.77	161.23	178.81	193.25	206.8	220.66	235.04	251.4
Morocco	Male	0	0	0	0	0	0	0.78	5.22	14.04	27.23	42.26	57.28	69.72	79.02	85.85	91.95	97.46	104.74	111.97	119.67
Morocco	Female	0	0	0	0	0	0	1.14	8	24.83	54.79	93.53	133.43	166.34	188.31	203.15	214.05	223.4	233.08	244.97	258.56
Mozambique	Male	0	0	0	0	0	0	1.7	11.93	34.77	67.64	102.21	133.78	157.24	173.56	183.72	192.06	200.59	208.32	218.62	227.83
Mozambique	Female	0	0	0	0	0	0	2.14	15.68	48.94	98.71	154.35	206.67	248.7	277.65	298.44	314.55	327.3	340.87	355.94	370.07
Myanmar	Male	0	0	0	0	0	0	0.11	0.68	1.51	2.5	3.84	5.26	6.84	8.47	10.01	12.07	14.59	17.72	22.22	28.3
Myanmar	Female	0	0	0	0	0	0	0.24	1.5	3.55	6.08	9.87	14.62	19.56	24.11	28.67	32.93	39.38	46.24	55.62	67.9
Namibia	Male	0	0	0	0	0	0	2.86	20.18	56.01	102.28	147.29	182.37	208.64	223.29	233.05	240.99	248.2	255.49	264.32	272.96
Namibia	Female	0	0	0	0	0	0	3.21	22.71	65.98	124.78	183.87	234.45	274.56	302.06	321.32	335.83	348.37	359.83	373.85	388.09
Nepal	Male	0	0	0	0	0	0	0.61	4.11	11.21	22.26	36.34	50.98	65.24	76.51	85.2	93.36	102.76	111.89	122.89	133.93
Nepal	Female	0	0	0	0	0	0	0.97	6.75	20.36	42.49	70.73	100.51	128.1	150.18	166.91	181.82	196.41	210.13	229.51	247.32
Netherlands	Male	0	0	0	0	0	0	2.1	13.44	33.25	62.37	104.01	154.68	202.03	235.25	253.04	264.45	271.83	277.84	284.72	291.03
Netherlands	Female	0	0	0	0	0	0	2.23	15.46	43.86	92.9	161.01	236.22	297.2	339.12	362.32	378.72	389.43	395.87	401.01	407.27
New Zealand	Male	0	0	0	0	0	0	1.46	10.44	32.58	69.78	117.45	168.8	216.05	252.79	278.79	297.75	312.17	323.52	336.48	345.62
New Zealand	Female	0	0	0	0	0	0	3.15	26.37	100.18	245.58	427.58	594.38	707.59	769.81	803.51	818.14	824.08	823.15	822.22	819.02
Nicaragua	Male	0	0	0	0	0	0	0.64	4.5	13.46	28.72	47.69	66.93	83.47	95.35	104.85	113.17	121.69	129.34	139.84	151.12
Nicaragua	Female	0	0	0	0	0	0	0.82	5.92	18.75	42.46	74.51	107.78	136.85	158	174.2	187.26	199.24	211.39	227.01	244.26
Niger	Male	0	0	0	0	0	0	1.71	12.21	35.41	69.01	104.78	135.98	160.45	176.6	187.85	197.48	205.67	213.17	222.89	233.04
Niger	Female	0	0	0	0	0	0	2.15	15.9	49.13	100.22	155.82	208.61	251.24	280.06	300.82	316.27	330.07	343.81	358.3	372.9
Nigeria	Male	0	0	0	0	0	0	1.55	11.28	34.66	69.7	106.84	140.67	168.01	187.81	203.05	216.52	230.64	244.33	259.54	274.38
Nigeria	Female	0	0	0	0	0	0	1.95	14.54	46.94	99.1	157.89	213.45	259.79	295.3	322.78	345.86	368.02	389.89	413.89	437.64
North Macedonia	Male	0	0	0	0	0	0	0.91	6.39	19.09	39.57	65.76	92.93	116.93	134	144.89	154.81	164.03	172.8	182.4	191.46
North Macedonia	Female	0	0	0	0	0	0	0.97	6.79	20.13	42.79	72.8	105.84	136.17	160.25	178.12	192.33	205.29	218.75	233.63	250.09
Northern Mariana Islands	Male	0	0	0	0	0	0	0.26	1.6	3.72	6.63	11.04	16.71	23.06	28.55	33.99	39.2	45	51.66	59.53	68.49
Northern Mariana Islands	Female	0	0	0	0	0	0	0.46	3.02	7.55	14.58	23.68	34.18	44.13	52.51	60.57	67.68	76.68	86.22	97.77	111.79
Norway	Male	0	0	0	0	0	0	2.37	17.05	51.08	106.77	178.68	258.31	328.84	377.58	407.24	424.82	436.39	444.52	452.35	457.04
Norway	Female	0	0	0	0	0	0	3.58	28.38	99.19	231.28	397.93	556.84	672.83	742.54	782.23	803.35	813.06	813.06	811.06	806.22
Oman	Male	0	0	0	0	0	0	0.78	5.23	14.06	27.19	42.72	57.36	69.6	78.28	85.08	90.26	95.98	102.79	109.79	118.52

Oman	Female	0	0	0	0	0	0	1.15	8.13	24.77	54.79	94.19	133.94	166.28	187.42	201.69	211.73	220.21	230.13	242.01	256.07
Pakistan	Male	0	0	0	0	0	0	0.66	4.43	12.27	24.18	38.92	54.77	69.76	82.67	93.42	104.98	117.99	130.91	146.14	162.95
Pakistan	Female	0	0	0	0	0	0	0.98	6.84	21.19	44.96	74.4	106.04	135.47	160.6	181.91	202.51	223.19	245.98	270.77	296.16
Palestine	Male	0	0	0	0	0	0	0.78	5.07	13.85	27.03	42.07	56.59	68.93	78.05	84.74	90.88	96.68	103.66	111.07	119.7
Palestine	Female	0	0	0	0	0	0	1.15	8.04	24.85	54.46	93.54	133.23	165.71	187.92	202.15	212.76	221.69	232.76	245.06	259.17
Panama	Male	0	0	0	0	0	0	0.64	4.45	13.57	28.64	47.81	67.64	83.86	95.37	104.74	113.02	121.04	129.54	139.3	150.57
Panama	Female	0	0	0	0	0	0	0.82	5.86	18.83	42.75	74.49	107.81	137.1	158.03	174.32	187.46	199.69	211.81	227.6	244.77
Papua New Guinea	Male	0	0	0	0	0	0	0.26	1.61	3.76	6.44	10.94	16.7	22.91	28.67	33.78	38.96	44.96	51.63	59.97	69.45
Papua New Guinea	Female	0	0	0	0	0	0	0.46	2.97	7.58	14.45	23.61	33.74	43.89	52.18	59.65	67.13	75.83	84.81	96.84	111.31
Paraguay	Male	0	0	0	0	0	0	0.57	3.97	10.98	21.56	34.84	49.62	62.25	72.23	80.04	87.77	95.93	105.57	115.59	127.45
Paraguay	Female	0	0	0	0	0	0	0.89	6.3	19.44	41.82	71.01	101.19	128.58	148.73	165.11	178.4	191.45	204.91	221.6	240.86
Peru	Male	0	0	0	0	0	0	0.55	3.85	10.76	22.22	36.68	52.42	66.04	76.5	84.7	91.79	100.04	108.36	118.14	130.54
Peru	Female	0	0	0	0	0	0	0.91	6.48	20.27	44.81	77.95	112.74	142.4	165.32	181.86	195.48	208.04	220.01	234.8	253.32
Philippines	Male	0	0	0	0	0	0	0.15	0.93	2.01	3.31	4.86	6.82	8.83	10.82	13.05	15.87	19.78	24.65	31.09	39.25
Philippines	Female	0	0	0	0	0	0	0.28	1.8	4.08	7.28	11.56	16.8	22.48	27.86	33.47	40.1	48.85	59.33	72.92	89.13
Poland	Male	0	0	0	0	0	0	0.94	6.62	20.07	42.02	69.12	96.83	121.97	141.83	158.16	172.73	187.02	200.74	215.45	229.65
Poland	Female	0	0	0	0	0	0	0.97	6.9	21.42	46	77.1	111.34	143.86	171.97	195.33	216.5	236.65	256.07	277.52	300.31
Portugal	Male	0	0	0	0	0	0	1.94	12.14	30.02	57.11	96.19	145.92	194.32	227.75	247.7	259.32	267.68	274.21	282.33	290.29
Portugal	Female	0	0	0	0	0	0	3.93	27.51	82.66	180.79	319.94	460.9	561.53	617.64	643.9	657.57	664.31	666.41	671.49	675.46
Puerto Rico	Male	0	0	0	0	0	0	0.57	3.9	10.81	21.59	35.06	49.16	62.02	71.28	79.27	86.89	95.45	104.22	114.57	125.62
Puerto Rico	Female	0	0	0	0	0	0	0.89	6.35	19.71	42.01	70.9	100.91	127.97	148.89	164.56	178.05	191.68	205.49	222.09	240.15
Qatar	Male	0	0	0	0	0	0	0.78	5.23	14.08	27.16	42.44	56.75	68.71	77.27	84.28	89.89	95.44	101.62	108.68	116.49
Qatar	Female	0	0	0	0	0	0	1.14	7.93	25.1	54.37	93.53	131.71	163.26	184.77	198.69	208.43	216.81	225.42	238.42	251.7
Republic of Korea	Male	0	0	0	0	0	0	0.92	6.17	17.38	36.69	65.78	103.27	142.26	174.24	195.77	209.05	217.84	225.41	234.55	243.52
Republic of Korea	Female	0	0	0	0	0	0	5.4	43.78	155.3	366.47	626.27	843.99	966.23	1018.92	1033.18	1035.4	1028.89	1020.91	1013.39	1005.98
Republic of Moldova	Male	0	0	0	0	0	0	1.86	12.9	37.23	74.31	117.48	158.9	192.43	215.56	230.61	242.06	251.21	259.18	268.26	277.22
Republic of Moldova	Female	0	0	0	0	0	0	2.6	18.66	59.13	125.67	208.96	292.14	361.59	407.77	435.84	453.72	464.67	475.92	490.38	504.93
Romania	Male	0	0	0	0	0	0	0.91	6.39	18.97	39.72	65.54	93.1	116.88	134.2	146.32	155.82	165.71	175	185.02	195.1
Romania	Female	0	0	0	0	0	0	0.97	6.69	20.24	42.82	72.78	106.17	137.76	161.62	180.27	194.68	208.61	222.98	238.81	254.8
Russian Federation	Male	0	0	0	0	0	0	3.97	32.89	116.95	257.82	405.29	515.99	583.73	618.76	637.11	653.62	666.4	677.06	679.24	677.85

Russian Federation	Female	0	0	0	0	0	0	4.15	36.18	135.99	320.6	533.5	710.75	827.88	893.01	928.51	950.21	960.99	972.2	980.61	986.84
Rwanda	Male	0	0	0	0	0	0	1.7	12.02	34.73	68.34	103.14	134.78	158.83	174.94	185.98	194.49	203.28	211.21	220.86	230.9
Rwanda	Female	0	0	0	0	0	0	2.18	15.93	49.07	99.58	155.28	208.1	249.88	279.17	300.48	316.3	329.04	342.46	358	372.58
Saint Lucia	Male	0	0	0	0	0	0	0.57	3.94	10.93	21.54	34.76	49.51	61.83	71.79	79.26	86.7	95.08	103.76	114.46	125.97
Saint Lucia	Female	0	0	0	0	0	0	0.89	6.36	19.36	41.92	70.39	100.38	126.57	147.71	162.93	177.11	190.2	204.18	220.95	240.16
Saint Vincent and the Grenadines	Male	0	0	0	0	0	0	0.57	3.97	10.78	21.5	34.95	49.39	61.74	71.73	79.76	86.9	95.65	104.67	115.2	126.95
Saint Vincent and the Grenadines	Female	0	0	0	0	0	0	0.89	6.39	19.52	42	70.51	101.02	127.83	147.83	164.45	177.21	190.47	205.53	223.07	242.9
Samoa	Male	0	0	0	0	0	0	0.26	1.62	3.75	6.57	11.17	16.73	23.09	28.96	33.76	39.07	44.84	51.41	59.79	69.43
Samoa	Female	0	0	0	0	0	0	0.46	2.97	7.55	14.51	23.93	34.06	44.11	52.33	59.82	67.83	76.1	85.51	97.78	112.52
Sao Tome and Principe	Male	0	0	0	0	0	0	1.7	12.04	35.22	69.22	104.56	135.78	159.77	175.57	185.87	194.23	202.23	209.63	218.1	227.13
Sao Tome and Principe	Female	0	0	0	0	0	0	2.19	16.12	49.39	100.16	156.66	208.84	250.78	279.46	299.82	314.47	327.76	341.04	355.36	369.55
Saudi Arabia	Male	0	0	0	0	0	0	2.3	16.66	51.32	105.5	159.94	202.32	227.12	241.28	248.34	252.68	256.92	261.14	266.42	270.27
Saudi Arabia	Female	0	0	0	0	0	0	2.3	17.27	57.44	132.54	229.61	314.7	369.49	398.47	411.85	419.67	424.5	429.28	436.67	445.81
Senegal	Male	0	0	0	0	0	0	1.69	12.08	35.52	69.07	104.35	135.94	159.45	175.58	186.08	194.45	203.95	211.62	219.9	230.1
Senegal	Female	0	0	0	0	0	0	2.21	15.98	49.21	99.53	156.18	208.1	249.84	278.85	298.72	314.57	327.21	341.37	354.98	370.57
Serbia	Male	0	0	0	0	0	0	0.91	6.36	18.84	39.51	65.25	92.56	116.49	133.31	145.32	154.95	164.14	173.02	182.39	192.56
Serbia	Female	0	0	0	0	0	0	0.97	6.74	20.03	42.57	72.45	105.93	136.1	160.58	178.64	193.02	205.32	219.25	234.6	250.1
Seychelles	Male	0	0	0	0	0	0	0.11	0.68	1.51	2.48	3.82	5.27	6.81	8.4	10.05	11.81	14.48	17.52	22.01	28.11
Seychelles	Female	0	0	0	0	0	0	0.24	1.49	3.52	6.14	10.04	14.51	19.38	24.34	28.29	33.09	39.27	46.67	56.41	68.44
Sierra Leone	Male	0	0	0	0	0	0	1.7	11.95	35.29	68.43	103.97	135.57	159.53	175.78	186.24	195.83	204.59	212.38	221.98	231.78
Sierra Leone	Female	0	0	0	0	0	0	2.16	16.07	49.16	99.65	155.49	208.14	250.02	279.66	299.7	314.29	328.56	342.48	357.87	372.03
Singapore	Male	0	0	0	0	0	0	0.84	5.53	15.26	32.11	56.94	89.39	124.49	153.54	174.61	188	198.54	206.78	216.54	226.18
Singapore	Female	0	0	0	0	0	0	4.26	35.06	123.94	289.92	497.43	681.47	796.99	851.12	871.68	877.12	877.17	874.92	877.81	879.94
Slovakia	Male	0	0	0	0	0	0	0.91	6.44	19.14	39.26	65.34	92.67	115.84	133.52	145.03	153.98	163.22	172.46	182.06	192.33
Slovakia	Female	0	0	0	0	0	0	0.97	6.73	20.25	42.75	72.62	105.91	136.62	161	179.81	194.86	206.82	219.65	234.9	250.74
Slovenia	Male	0	0	0	0	0	0	0.91	6.31	18.94	39.58	65.54	92.89	116.63	133.71	144.85	154.75	163.41	171.16	179.55	188.1
Slovenia	Female	0	0	0	0	0	0	0.97	6.62	20.25	42.71	72.64	106.06	136.66	161.13	178.82	193.15	205.11	216.04	228.86	242.65
Solomon Islands	Male	0	0	0	0	0	0	0.26	1.62	3.72	6.66	11.07	16.72	23.1	28.66	34.1	38.98	44.99	51.68	59.74	69.29

Solomon Islands	Female	0	0	0	0	0	0	0.46	2.97	7.52	14.45	23.68	33.89	43.89	52.56	60.06	68.01	76.6	86.26	97.63	112.14
Somalia	Male	0	0	0	0	0	0	1.7	11.95	35.03	68.54	103.54	134.66	158.38	175.01	186.14	194.63	203.11	212.15	220.64	231.32
Somalia	Female	0	0	0	0	0	0	2.16	15.87	48.83	99.15	154.91	206.73	249.01	278.82	298.8	314.69	329.31	342.07	356.95	373.25
South Africa	Male	0	0	0	0	0	0	2.36	17.3	52.23	101.16	148.37	187.69	217.31	237.56	251.95	264.76	276.97	288.92	302.26	316.2
South Africa	Female	0	0	0	0	0	0	2.61	19.41	60.42	121.24	184.27	240.3	285.08	318.88	345.49	367.43	388.03	408.03	429.14	451.19
South Sudan	Male	0	0	0	0	0	0	1.7	11.87	35.07	67.88	102.51	133.52	157	172.93	182.74	190.92	200.07	207.48	217.26	226.89
South Sudan	Female	0	0	0	0	0	0	2.13	16.07	48.97	99.14	153.97	204.73	246.47	272.73	290.26	304.65	319.39	333.31	348.18	363.51
Spain	Male	0	0	0	0	0	0	2.13	13.89	36.4	71.89	127.6	201.89	269.53	313.23	336.64	347.25	354.25	360.88	366.8	374.28
Spain	Female	0	0	0	0	0	0	3.25	23.46	73.99	180.41	350.55	532.35	652.59	711.46	735.41	746.08	750.53	749.35	749.3	753.36
Sri Lanka	Male	0	0	0	0	0	0	0.11	0.68	1.5	2.49	3.84	5.2	6.95	8.39	10.01	11.89	14.37	17.56	22.08	27.63
Sri Lanka	Female	0	0	0	0	0	0	0.24	1.51	3.52	6.14	9.92	14.47	19.49	24.32	28.33	33.1	38.98	46.58	55.62	67.83
Sudan	Male	0	0	0	0	0	0	0.78	5.08	14.08	26.95	42.43	57.53	69.71	79.2	85.74	91.88	97.73	103.88	111.74	119.56
Sudan	Female	0	0	0	0	0	0	1.15	7.96	24.65	54.53	94.17	134.14	166.68	188.94	203.2	213.31	222.2	232.15	244.72	258.29
Suriname	Male	0	0	0	0	0	0	0.57	3.91	10.87	21.46	34.96	48.91	61.8	71.82	79.27	87.03	95.04	104.02	114.59	125.83
Suriname	Female	0	0	0	0	0	0	0.89	6.26	19.44	41.83	70.52	100.44	127.5	148.05	163.18	177.29	190.56	204.11	220.49	240.07
Sweden	Male	0	0	0	0	0	0	1.23	8.34	23.76	48.57	80.9	118.59	155.88	186.05	208.53	224.91	239.39	252.31	263.59	275.42
Sweden	Female	0	0	0	0	0	0	1.44	10.23	32.26	69.72	119.18	173.33	222.84	261.29	290.78	313.95	333.45	348.8	362.92	376.08
Switzerland	Male	0	0	0	0	0	0	2.79	18	45.65	87.01	146.93	222.63	288.82	332.16	354.23	365.81	372.39	376.18	380.78	385.12
Switzerland	Female	0	0	0	0	0	0	3.03	20.95	61.4	132.62	234.44	342.2	426.49	478.32	506.32	521.83	529.61	531.65	534.17	537.93
Syrian Arab Republic	Male	0	0	0	0	0	0	0.78	5.14	13.97	27.19	42.27	57.11	69.71	78.77	85.46	91.94	97.29	102.26	108.98	116.92
Syrian Arab Republic	Female	0	0	0	0	0	0	1.14	7.94	24.78	54.86	94.28	133.8	165.69	188.35	203.03	212.46	220.76	228.96	239.08	251.86
Tajikistan	Male	0	0	0	0	0	0	1.32	9.43	28.4	58.1	93.41	129.93	162.27	185.85	203.25	216.49	226.57	235.21	244.01	252.49
Tajikistan	Female	0	0	0	0	0	0	1.97	14.81	50.1	113.81	195.95	279.95	348.41	393.74	421.49	440.75	452.25	463.24	476.07	490.56
Thailand	Male	0	0	0	0	0	0	0.11	0.68	1.5	2.5	3.83	5.19	6.87	8.45	10.18	12.12	14.74	18	22.75	28.62
Thailand	Female	0	0	0	0	0	0	0.24	1.51	3.5	6.18	9.99	14.81	19.56	24.42	28.77	33.77	40.09	47.47	57.25	69.98
Timor-Leste	Male	0	0	0	0	0	0	0.11	0.68	1.51	2.5	3.81	5.1	6.75	8.35	10.04	12.04	14.35	17.45	22.11	28.15
Timor-Leste	Female	0	0	0	0	0	0	0.24	1.52	3.54	6.13	10.04	14.5	19.51	23.98	28.41	33.3	39.21	46.47	56.24	68.27
Togo	Male	0	0	0	0	0	0	1.7	12.13	35.45	69.14	104.05	135.83	159.88	175.69	186.96	195.79	205.35	212.86	222.16	232.09
Togo	Female	0	0	0	0	0	0	2.2	15.93	49.07	99.9	155.75	208.38	250.95	280.89	301	316.6	330.54	343.43	358.98	373.77
Tonga	Male	0	0	0	0	0	0	0.26	1.62	3.73	6.51	11.14	16.76	23.32	28.79	33.93	39.41	45.23	51.74	60.65	70.03

Tonga	Female	0	0	0	0	0	0	0.46	2.96	7.67	14.7	23.88	33.94	44.12	52.57	60.25	68.05	76.6	86.77	98.9	113.67
Trinidad and Tobago	Male	0	0	0	0	0	0	0.57	3.97	10.6	21.51	35.03	49.05	61.76	71.48	80.17	86.95	95.34	104.66	115.56	127.63
Trinidad and Tobago	Female	0	0	0	0	0	0	0.89	6.4	19.64	41.74	70.38	100.76	127.72	147.76	164.16	177.72	191.49	205.84	223.4	243.76
Tunisia	Male	0	0	0	0	0	0	0.78	5.2	14.01	27.17	42.57	57.58	69.41	78.71	85.45	91.17	96.83	103.01	111	118.32
Tunisia	Female	0	0	0	0	0	0	1.15	8.07	24.85	54.78	94.44	134.3	166.87	190.21	203.98	214.19	223.72	233.13	244.89	259.29
Turkey	Male	0	0	0	0	0	0	0.19	1.21	2.95	5.47	8.76	12.52	16.33	19.27	22.05	24.94	28.12	32.19	37.96	45.71
Turkey	Female	0	0	0	0	0	0	0.33	2.25	6.25	13.33	23.65	35.48	46.27	55.62	62.72	68.99	75.88	84.05	94.79	109.08
Turkmenistan	Male	0	0	0	0	0	0	1.51	10.92	33	67.67	109.15	149.23	185.06	211.1	229.41	242.5	253.16	261.37	270.1	278.88
Turkmenistan	Female	0	0	0	0	0	0	1.26	9.29	29.66	65.82	113.42	165.58	210.43	245.62	269.71	285.84	299.19	311.27	327.4	343.53
Uganda	Male	0	0	0	0	0	0	1.71	11.95	34.94	68.12	103.63	134.48	158.35	174.64	185.96	194.36	203.56	211.82	221.66	231.07
Uganda	Female	0	0	0	0	0	0	2.15	15.9	48.74	99.63	155.71	207.51	249.51	279.81	301.12	316.52	330.8	344.8	358.84	373.39
Ukraine	Male	0	0	0	0	0	0	1.21	8.59	25.44	51.07	81.08	111	138.33	159.39	176.81	191.13	205.67	218.4	233.81	247.09
Ukraine	Female	0	0	0	0	0	0	1.83	13.69	45.35	98.99	165.61	234.86	294.45	341.18	375.44	399.28	419.57	438.36	458.81	479.43
United Arab Emirates	Male	0	0	0	0	0	0	0.78	5.13	13.94	26.97	42.15	56.8	69.17	78.02	84.48	90.59	96.14	102.88	110.26	117.89
United Arab Emirates	Female	0	0	0	0	0	0	1.14	8.08	24.63	54.96	94.05	132.74	164.99	185.37	199.44	210.19	218.47	228.59	240.97	254.94
United Kingdom	Male	0	0	0	0	0	0	2.2	15.05	42.97	87.72	146.33	212.31	271.51	313.77	340.04	357.35	370.1	380.25	391.49	402.16
United Kingdom	Female	0	0	0	0	0	0	2.44	18.06	58.34	129.97	223.75	319.91	398.82	452.78	488.15	511.71	528.4	540.44	553.18	565.15
United Republic of Tanzania	Male	0	0	0	0	0	0	1.7	12.05	35.33	68.49	103.9	135.56	159.36	175.79	186.92	195.14	204.37	211.75	221.07	231.24
United Republic of Tanzania	Female	0	0	0	0	0	0	2.17	15.73	48.94	99.58	155.25	208.08	250.52	279	299.53	315.68	328.83	341.49	355.86	369.72
United States of America	Male	0	0	0	0	0	0	5.67	34.58	78.64	148.07	261.31	408.78	539.62	610.18	637.81	647.53	650.79	654.2	660.06	658.71
United States of America	Female	0	0	0	0	0	0	8.22	53.36	140.56	304.93	562.95	826.18	989.69	1052.42	1067.93	1066.62	1060.24	1057.07	1055.94	1044.92
United States Virgin Islands	Male	0	0	0	0	0	0	0.57	3.95	10.82	21.68	35.08	49.08	61.96	72.01	80.08	87.25	95.55	104.54	114.92	126.43
United States Virgin Islands	Female	0	0	0	0	0	0	0.89	6.32	19.6	42.15	70.72	101.16	128.55	149.01	165.57	179	192.5	206.55	223.55	242.33
Uruguay	Male	0	0	0	0	0	0	1.67	11.22	32.1	65.55	110.26	162.29	209.46	243.86	264.47	276.77	286.07	293.31	301.99	309.55
Uruguay	Female	0	0	0	0	0	0	3.91	29.76	99.09	227.39	390.43	542.69	641.03	689.65	711.45	722.79	726.97	729.46	734.26	736.48
Uzbekistan	Male	0	0	0	0	0	0	1.32	9.6	28.43	58.08	93.68	129.92	161.92	186.4	203.61	215.78	225.81	235.35	243.33	251.75
Uzbekistan	Female	0	0	0	0	0	0	1.97	14.87	49.57	113.12	195.34	278.65	347.38	393.73	422.91	440.01	452.23	464.49	476.31	491.27
Vanuatu	Male	0	0	0	0	0	0	0.26	1.6	3.74	6.6	11.02	16.64	23.15	28.71	33.71	39.27	45.36	51.94	60.03	69.41

Vanuatu	Female	0	0	0	0	0	0	0.46	2.97	7.65	14.47	23.6	33.86	44.08	52.3	60.28	67.71	76.62	86.24	98.05	113.66
Venezuela (Bolivarian Republic of)	Male	0	0	0	0	0	0	0.22	1.5	4.07	8.23	14.24	21.03	27.66	33.11	37.88	42.68	48.09	54.04	62.08	72.54
Venezuela (Bolivarian Republic of)	Female	0	0	0	0	0	0	0.37	2.64	7.76	17.13	30.24	45.48	59.75	71.83	81.79	91.11	101.2	112.18	126.46	144.07
Viet Nam	Male	0	0	0	0	0	0	0.11	0.68	1.51	2.5	3.78	5.21	6.86	8.49	10.09	12.14	14.74	17.87	22.63	28.53
Viet Nam	Female	0	0	0	0	0	0	0.24	1.51	3.54	6.26	10	14.69	19.63	24.66	28.77	33.8	39.77	47.09	56.35	68.63
Yemen	Male	0	0	0	0	0	0	0.78	5.23	14.11	27.08	42.4	57.34	69.9	79.31	85.78	91.87	97.82	103.9	111.19	119.33
Yemen	Female	0	0	0	0	0	0	1.14	7.92	24.71	53.88	93.47	133.31	166.27	188.8	203.18	213.38	221.45	231.22	243.83	256.79
Zambia	Male	0	0	0	0	0	0	1.7	11.84	35.07	67.55	103.09	133.97	159.23	175.83	186.67	195.13	203.57	211.88	221.64	231.27
Zambia	Female	0	0	0	0	0	0	2.15	15.84	48.94	99.13	153.46	206.34	249.63	280.04	300.54	316.11	331.26	344.55	360.22	374.21
Zimbabwe	Male	0	0	0	0	0	0	2.9	20.01	56.11	102.57	147.01	183.48	208.65	224.58	234.17	242.04	249.7	256.97	266.24	273.92
Zimbabwe	Female	0	0	0	0	0	0	3.2	22.92	66.02	125.34	183.63	235.42	274.04	301.12	320.29	334.41	346.54	358.99	373.29	388.91

Supplementary Figures

Supplementary figure 1.

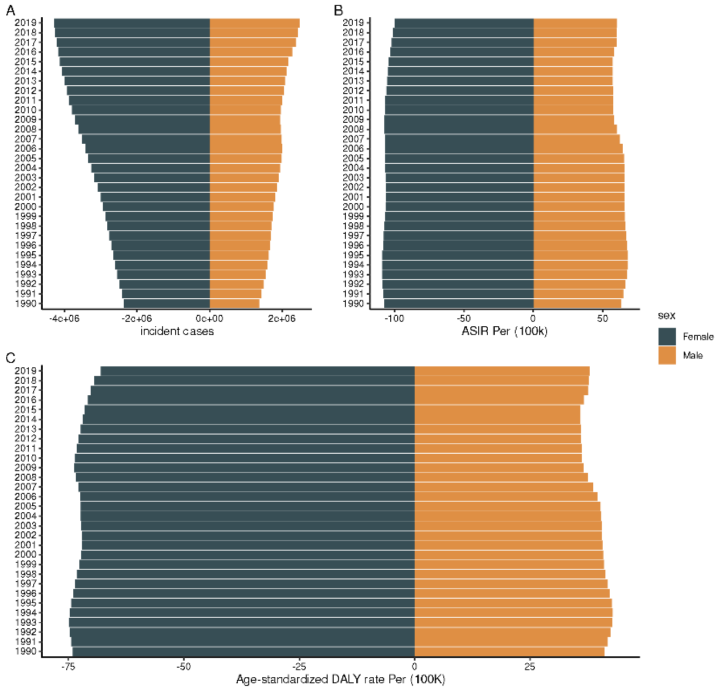

Supplementary figure 1. The incident cases (A), age standardized incidence (B), and age-standardized DALYs (C) rates of hand osteoarthritis between 1990 and 2019 both males and females.

Supplementary figure 2

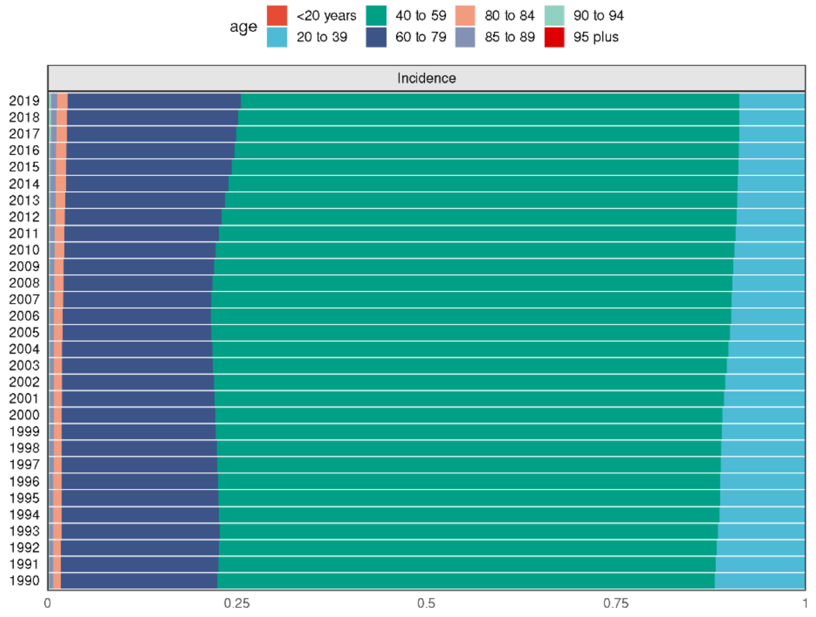

Supplementary figure 2. The proportion of different ages in incidence of hand osteoarthritis by years.

Supplementary figure 3.

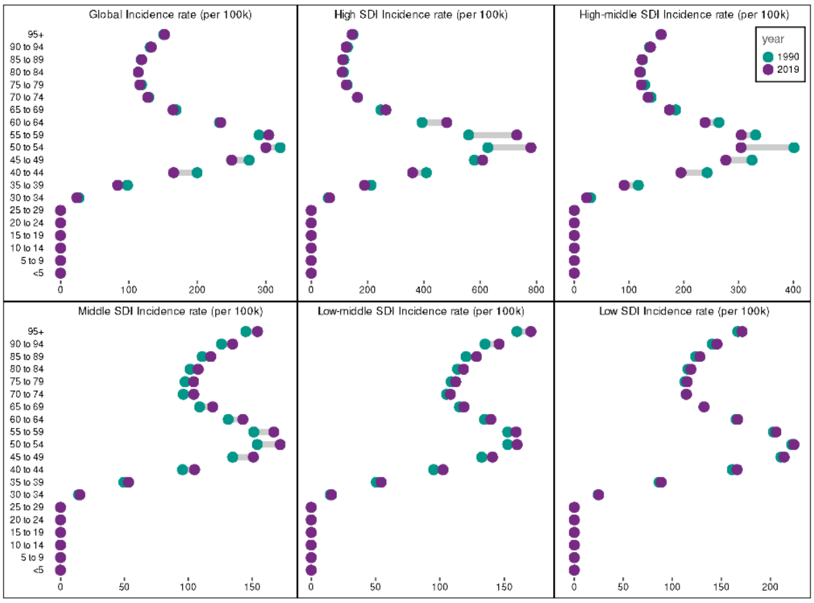

Supplementary figure 3. Distribution of different ages in incidence of hand osteoarthritis in global population.

Supplementary figure 4.

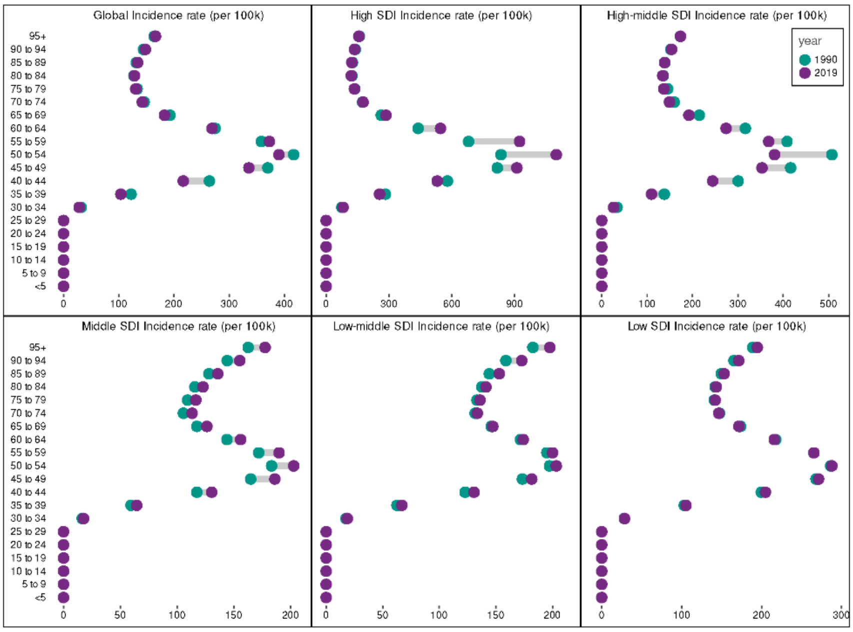

Supplementary figure 4. Distribution of different ages in incidence of hand osteoarthritis in female subjects.

Supplementary figure 5.

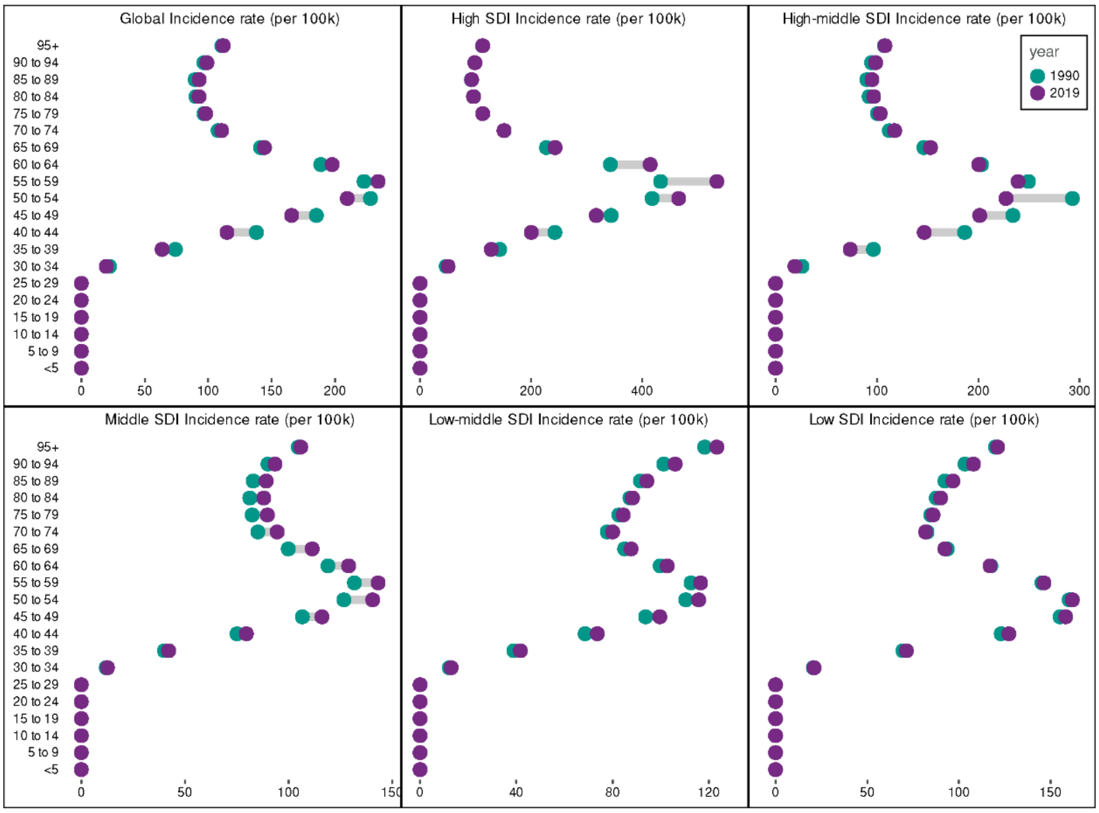

Supplementary figure 5. Distribution of different ages in incidence of hand osteoarthritis in male subjects.

Supplementary figure 6.

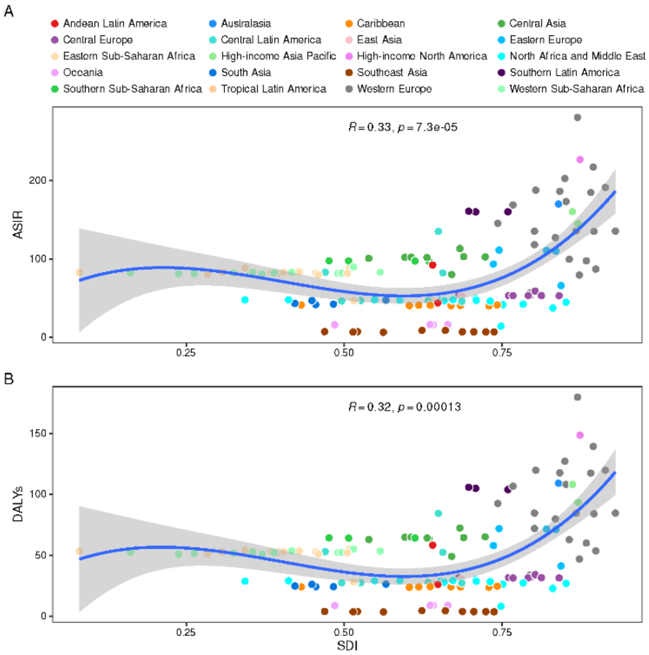

Supplementary figure 6. The age standardized incidence (A) and DALY (B) rates of

hand osteoarthritis per 100,000 population among regions based on SDI in 2019.

Supplementary figure 7

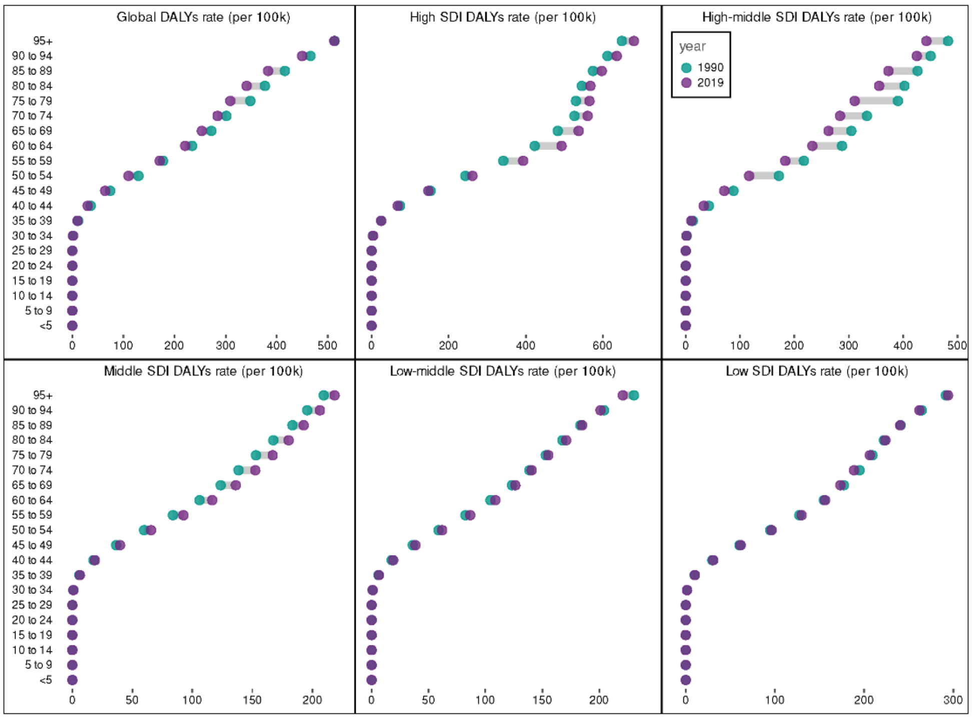

Supplementary figure 7. Distribution of different ages in DALYs of hand osteoarthritis in global level.

Supplementary figure 8.

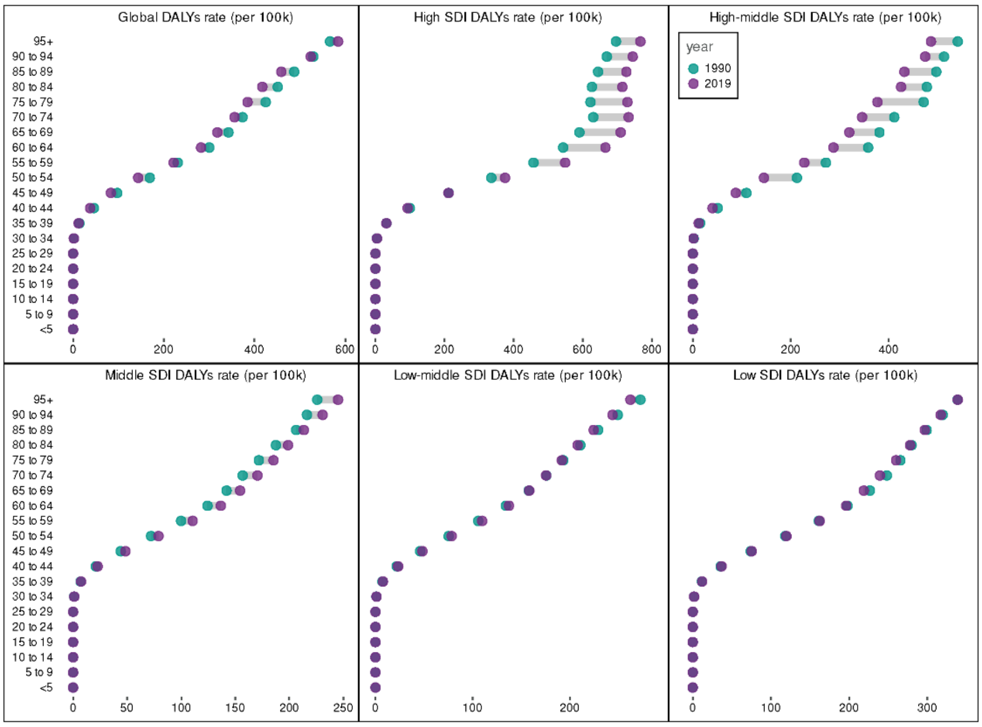

Supplementary figure 8. Distribution of different ages in DALYs of hand osteoarthritis in females.

Supplementary figure 9

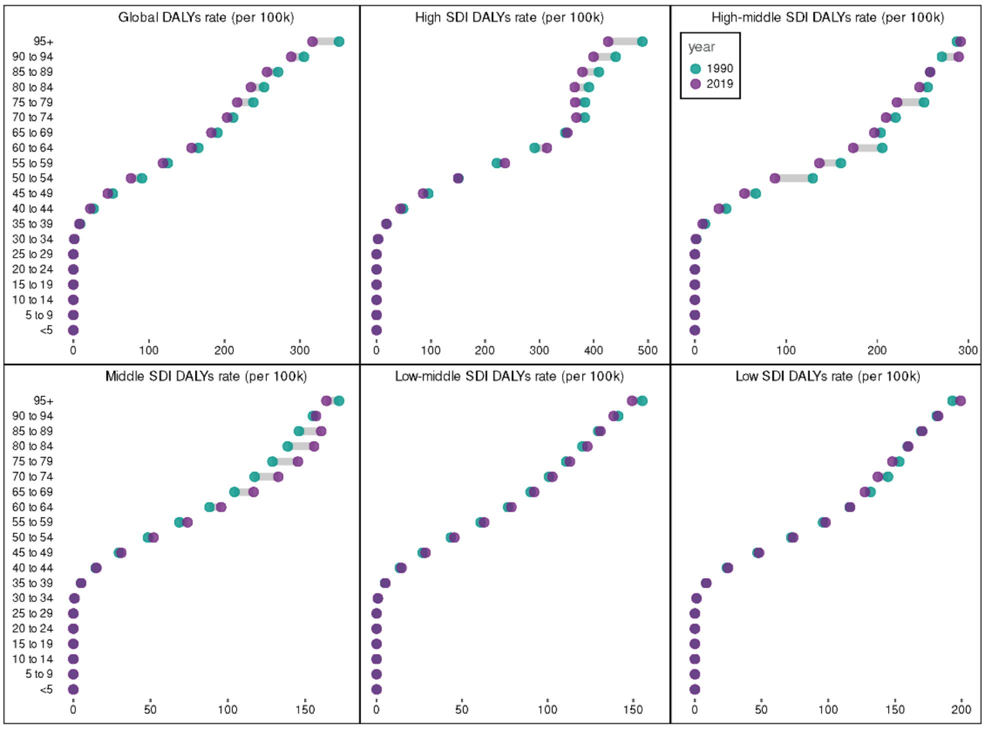

Supplementary figure 9. Distribution of different ages in DALYs of hand osteoarthritis in males.

Supplementary figure 10

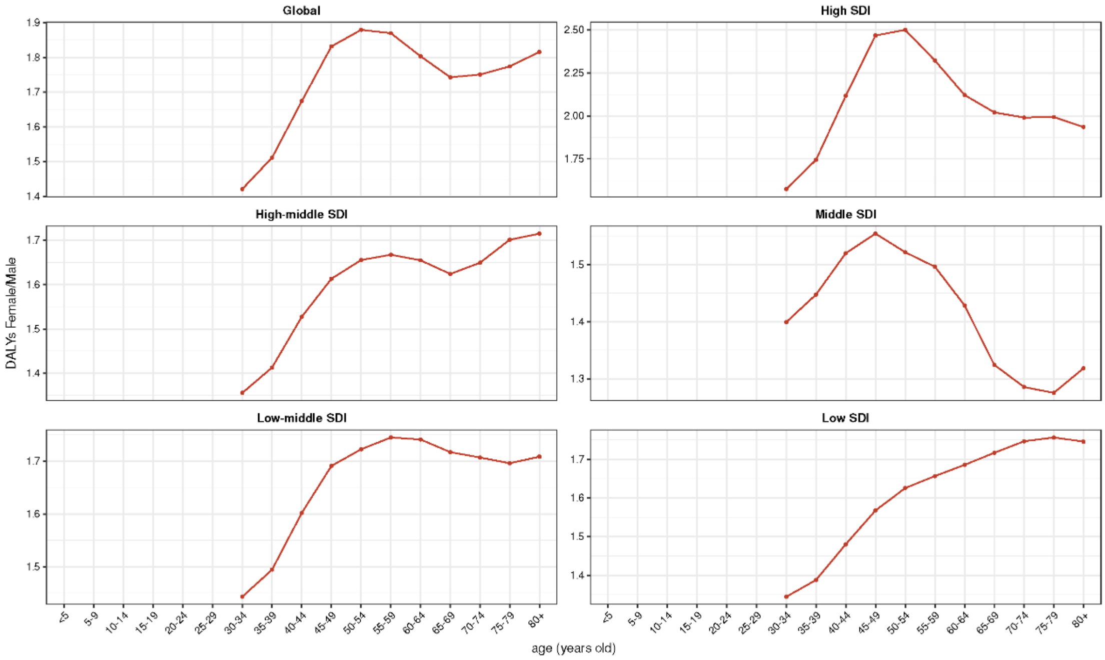

Supplementary figure 10. The ratio of female to male DALYs among different age groups in global. SDI, socio-demographic index.
